# Determinants of associations between codon and amino acid usage patterns of microbial communities and the environment inferred based on a cross-biome metagenomic analysis

**DOI:** 10.1038/s41522-023-00372-w

**Published:** 2023-01-24

**Authors:** Arup Panda, Tamir Tuller

**Affiliations:** Department of Biomedical Engineering, Tel Aviv University, Tel Aviv, 69978 Israel

**Keywords:** Molecular evolution, Metagenomics, Microbiome

## Abstract

Codon and amino acid usage were associated with almost every aspect of microbial life. However, how the environment may impact the codon and amino acid choice of microbial communities at the habitat level is not clearly understood. Therefore, in this study, we analyzed codon and amino acid usage patterns of a large number of environmental samples collected from diverse ecological niches. Our results suggested that samples derived from similar environmental niches, in general, show overall similar codon and amino acid distribution as compared to samples from other habitats. To substantiate the relative impact of the environment, we considered several factors, such as their similarity in GC content, or in functional or taxonomic abundance. Our analysis demonstrated that none of these factors can fully explain the trends that we observed at the codon or amino acid level implying a direct environmental influence on them. Further, our analysis demonstrated different levels of selection on codon bias in different microbial communities with the highest bias in host-associated environments such as the digestive system or oral samples and the lowest level of selection in soil and water samples. Considering a large number of metagenomic samples here we showed that microorganisms collected from similar environmental backgrounds exhibit similar patterns of codon and amino acid usage irrespective of the location or time from where the samples were collected. Thus our study suggested a direct impact of the environment on codon and amino usage of microorganisms that cannot be explained considering the influence of other factors.

## Introduction

Microbes are the most diverse and abundant organisms that can be found almost everywhere on earth but rarely alone. Microorganisms are being investigated over the last three centuries; however, understanding the attributes that characterize different microbial communities is still very challenging. Codons and amino acids (AAs) are basic community-level attributes of microorganisms that exhibit characteristic distributions at the level of community. Considering different direct and indirect evidence, previous studies suggested that the environment where microbes inhabit can influence their relative usage of codons and AAs^1–10^. However, till now, we have no clear understanding of how the codon and AA usage of microorganisms may vary across biomes and how the environment may influence this variation. Considering a large number of metagenomic samples from diverse ecological niches here we investigated the codon and AA usage patterns of microbial communities collected from various habitats.

Codons are the basic units of genetic code that encode AAs, the building blocks of proteins, in sets of three nucleotides. There are 61 codons (and 3 stop codons) encoding 20 AAs, thus most of the AAs are specified by multiple codons called synonymous codons^11–15^. Synonymous codons, although encode the same AA, are not used at equal frequencies, instead, each organism shows specific signatures in the choice and arrangement of these codons. This biasness in codon usage, called Codon Usage Bias (CUB), was observed in the protein-coding sequences of almost all organisms but most prominent in microorganisms^11–16^. CUB varies not only between organisms but also among the genes or along a gene of an organism; however, genes from the same organism show more uniform CUB than the genes from different organisms^12,15^. Because of its importance, CUB was regarded as a fundamental property of microorganisms that could influence their cellular fitness, phenotypic traits, and genome evolution^12,13,15,16^.

CUB was first traced several decades ago; however, the exact cause and consequences of it are still not clearly understood. The factors that were found to influence CUB can be grouped broadly into two categories (i) intrinsic factors such as gene expression, gene length, GC content, sequence composition, rate of recombination, mRNA secondary structure and stability, metabolism, protein folding, and the extent of horizontal gene transfer, etc., and (ii) extrinsic factors: mainly different environmental variables such as temperature, pH, salinity, humidity, etc.^10–16^. However, the intrinsic and extrinsic factors strongly correlate and overlap as the extrinsic factors can, directly and indirectly, affect the intrinsic ones.

Among the intrinsic factors, the gene expression level was considered as a strong determinant of CUB^13,15,16^. In most organisms, highly expressed genes are found to be encoded by the most frequent codons, which invoked the notion that selection for an efficient and accurate translation of highly expressed genes is a major cause of CUB^11,13,15,16^. CUB was also suggested to be an important determinant of mRNA folding and stability, which can affect translation initiation and elongation^13,15–17^. Moreover, codon usage was shown to regulate translation elongation rates to optimize co-translational protein folding processes^16,18^. According to the neutral theory, on the other hand, CUB exists mainly because of mutational bias, as some codons are more mutable than other codons^11,12,15,19^.

As codon usage, AA usage can also vary widely among prokaryotic and eukaryotic organisms and also among the proteins of the same organisms depending on their structure, functionality, or abundance^7,20,21^. These variations in AA frequencies have been related to several factors, such as their molecular weight, cost of synthesis, etc.^20–22^. However, the processes that shape AA usage at the molecular level are not clear. In some organisms, AA usage was found to correlate with their tRNA gene copy number supporting the translational optimization hypothesis^22^. Moreover, the extent of GC content or the intensity of CUB were also suggested to shape their distribution at the proteome level^5,7^, which may be an indirect consequence of environmental impacts as the environment can also modulate these factors (discussed later).

The environment where the microbes populate was suggested to influence every aspect of the microbial genome, including their codon and AA choice. Following this, previous studies speculated that codon or AA usage might be associated with the lifestyle of the organism^1–10,23,24^. For instance, considering microorganisms from several extreme habitats, it was suggested that microbes that could survive in a wide range of habitats exhibit high CUB^2^. An environment-specific trend has also been noticed in the relative usage of different AAs^5–7,23,24^. However, most of these studies were conducted at the species level considering genomes of cultured microorganisms precluding the community effect. In nature, microbes rarely survive and grow independently as individual species^25^. Thus, these species-level studies cannot provide the global scenario of the forces that microbes experience in their natural habitats.

The advent of metagenomics over the last decade has provided an unprecedented opportunity to extend earlier studies beyond the species boundaries and to explore the microbe world at the community level. Metagenomics (environmental genomics or community genomics) is a technique in which DNA sample(s) collected from different environments are analyzed directly without any need for culturing^26,27^. Thus, metagenomic studies can identify up to 99% of the total microbial population present in any sample and can provide information regarding the metabolic, functional, and taxonomic diversity of the whole population at a time. Metagenomic techniques are routinely used to characterize microorganisms and gene products that cannot be identified using common culturing techniques^27^. Moreover, shotgun metagenomic analyses are increasingly recognized as a powerful tool to explore the traits that microbes exploit to survive in different habitats and to understand their interactions with other environmental factors^27^.

Availing this opportunity, recent studies were directed to explore the codon or AA usage of microorganisms in a holistic approach considering the traits as a property of the community rather than of any species. For instance, a recent study of 11 environmental samples showed that genes from the same metagenome display comparable codon usage as compared to genes from any other metagenome^3^. An overall similarity in the codon usage pattern has also been noticed among some acidophilic bacteria in their natural consortium^4^. Considering the habitat information of 925 prokaryotic species retrieved from the fusionDB database^28^ we estimated how the codon and AA usage of these species may change with the environmental changes (details in materials and methods). Our study suggested that microbes from similar environments overall show a similar codon and AA usage pattern as compared to the microbes from other environmental biomes (Supplementary Table 1, Supplementary Fig. 1). However, to date, there is no integrated understanding of codon or AA usage of microorganisms at the community level. Particularly, it is not clear whether the environment has any direct impact on the codon or AA usage of microbe at the community level or not. If the environment has any direct impact, we would expect a similarity not only within a metagenome but also among the metagenomes from similar types of ecological niches.

Earlier, there has been a great interest to measure the extent of CUB in the genes of different prokaryotic and eukaryotic genomes^2,3,8,9^. To this end, several indices have been developed^14,15^. However, CUB of entire communities of microorganisms in their natural habitats has not been studied from the perspectives of their environmental features.

Considering all these aspects, we set the objectives for this study as following (i) to investigate whether there is any environment-specific trend in codon and AA usage of microorganisms at the community level, (ii) to understand the factors that may control such specificity (if any), (iii) to explore the extent of selection for CUB in different microbial communities using several codon usage indices. Considering over 400 metagenomic samples from 7 different environmental biomes, in this paper, we first studied their codon and AA usage specificities, and next, we made a detailed analysis of the forces that maintain the bias. We hope that our study of codon and AA usage at the community level will aid in the aggregation of more knowledge to explore the microbial world in a better context.

## Results

### General characteristics of the test samples

**Fig. 1:**
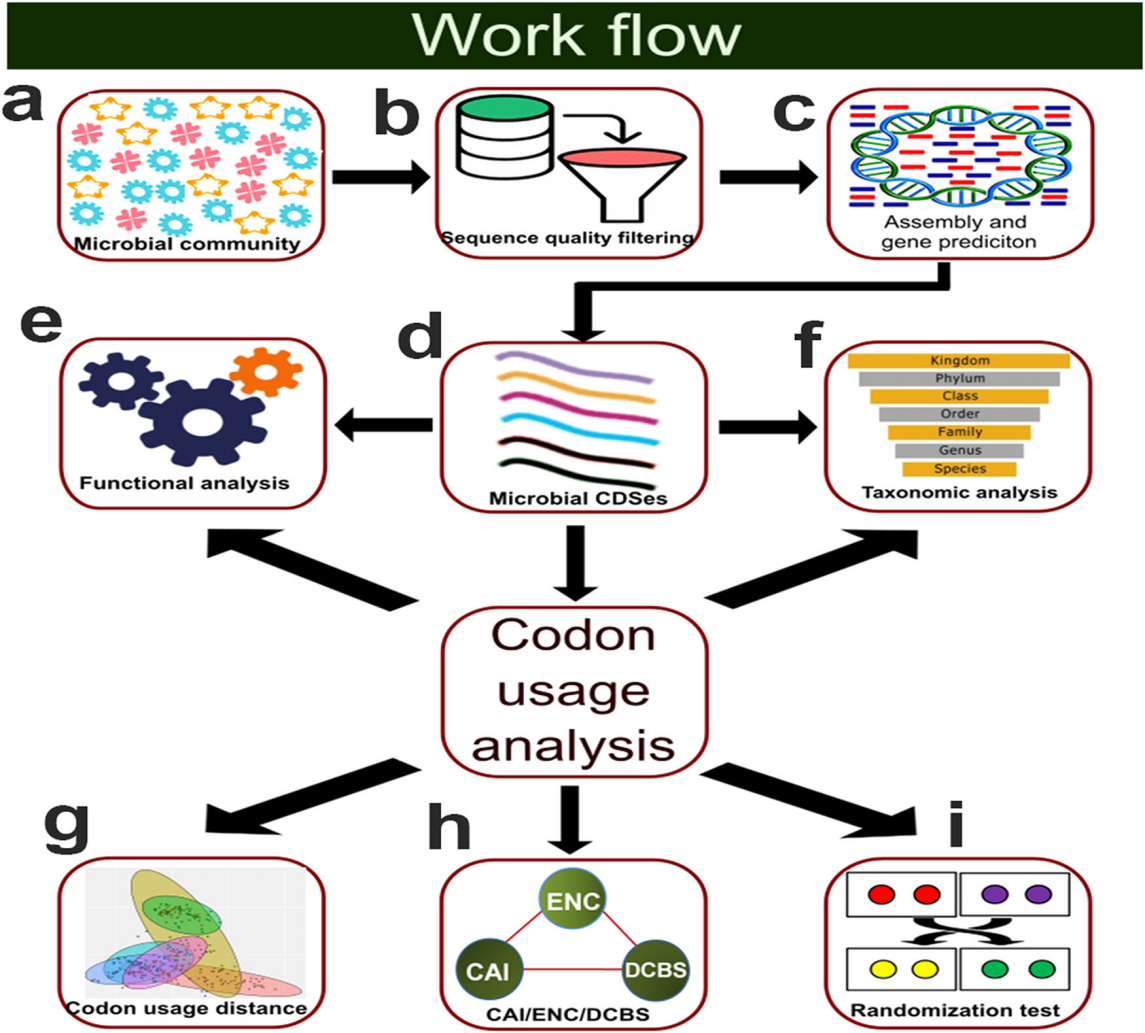
Metagenomic workflow. Here we described the steps that were employed in this analysis. **a** At the first step, raw metagenomic reads of each sample were collected from the Sequence Read Archive (SRA) database. **b** Reads were processed with a rigorous metagenomic pipeline and assembled. **c**, **d** From the assembled contigs, we predicted CDS (and protein) sequences that were employed for the codon usage analysis. **e**, **f** To understand how taxonomic and functional diversity influence the codon usage of the samples, we annotated the samples with the functional and taxonomic information and analyzed their impacts in detail. **g**. Analysis of codon usage similarity among different environmental samples. **h** To understand the codon usage specificity of the samples we calculated three indices of codon usage namely ENC, CAI, and DCBS values. **i** Next, we tested whether there is any evidence of selection on the codon usage of the samples by comparing the CAI/ENC/DCBS values of real sequences of the samples with their corresponding random sequences.

For this study, we considered 422 samples collected from seven environmental biomes: (i) freshwater, (ii) wastewater, (iii) digestive system, (iv) soil, (v) skin, (vi) sediment, and (vii) human oral samples. We considered these biomes because for each of these biomes we found at least 15 samples (to ensure adequate numbers of data points for reliable statistical tests) with more than 10,000 predicted CDS sequences. Collected shotgun metagenomic reads for each sample were processed through a rigorous metagenomic pipeline that does three steps sequentially (i) sequence quality check, (ii) assembly, and (iii) gene prediction (Fig. 1 and methods). The samples with the lowest (10,740) and highest (118,840,951) number of predicted CDSes are from wastewater and sediment biomes, respectively. We did not find any substantial bias in the number of predicted CDSes among the samples; however, sediment and soil samples, in general, showed slightly more CDSes than the other samples. To explore the impact of gene distribution on the codon and AA usage of the samples, we retrieved their functional and taxonomic information (see methods). On average, functional information could be found for 30-50% of the proteins in each sample. Considering the three ontologies biological process (BP), cellular component (CC), and molecular functions (MF) total 3702 Gene Ontology (GO) terms were found among the samples. A detailed discussion of their abundance is not the focus of this study, however, the relative occurrence of all these terms in each sample and the relative abundance values of different taxonomic ranks that were found in the samples can be found at Zenodo (10.5281/zenodo.7455261). To understand whether there is any environment-specific trend in the codon and AA usage of microorganisms at the community level, we conducted a series of analyses that are discussed in the next few sections.

### Distinct codon usage among the samples from similar environmental biome

**Fig. 2:**
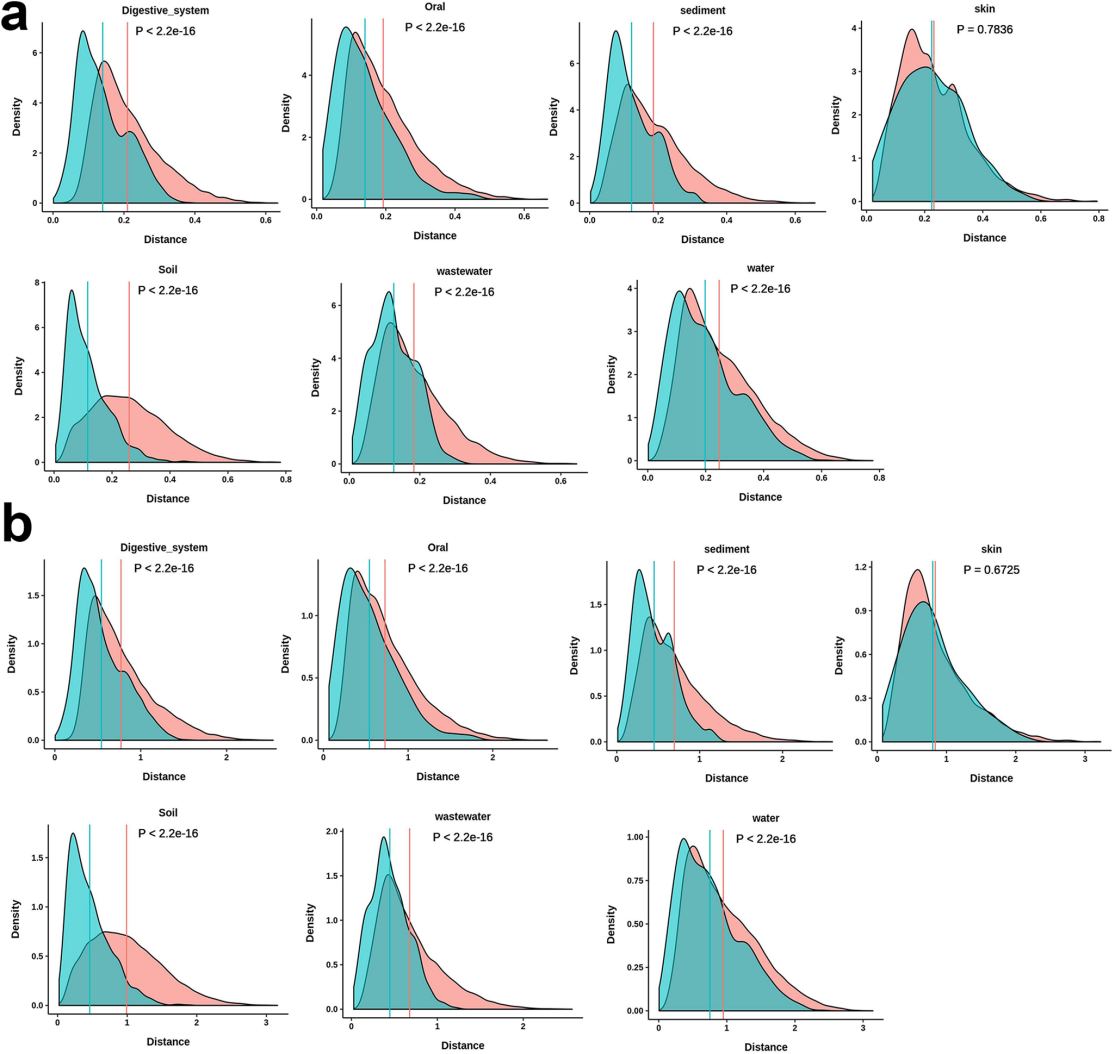
Density plots showing codon usage distances among the samples calculated by Endres–Schindelin method. This figure shows the density plots of codon usage distances among samples from seven selected ecological niches. **a** Codon frequencies were calculated as absCUFs. **b** Codon frequencies were calculated as synCUFs. For each sample in each selected habitat, we calculated all possible pairwise codon usage distances with all other samples either from the same (within-group comparison) or different habitats (between-groups comparison). Next, we compared the within-group distances of codon usage with that of between-group distances habitat-wise. Statistical significance of the differences for pairwise comparison of within to between-group distances was accessed by Mann-Whitney U test and the corresponding *P* values were shown in the respective panel. In each panel, vertical lines represent the average of within and between-group distances in codon usage frequencies, respectively. Blue line stands for within-group distances and pink for between-group distances, respectively.

At first, we studied the similarity/dissimilarity in codon usage frequencies among the test metagenomic samples grouped according to their habitats. Codon frequencies were calculated following two approaches: absolute codon usage frequency (absCUFs) and synonymous codon usage frequency (synCUFs). For each sample in these environmental groups, we calculated all possible pairwise codon usage distances with all other samples either from the same (within-group comparison) or different environmental groups (between-groups comparison) by three methods: Euclidean distance (abbreviate as EU distance), (ii) Bray-Curtis dissimilarity (abbreviated as BC dissimilarity), and (iii) Endres–Schindelin distance (abbreviated as ES distance) based on both absCUFs and synCUFs frequencies (details in materials and methods). Next, for each biome, we compared the average values of within-group distances with that of between-group distances. Figure 2 represents the density plots of codon usage distances estimated by ES method considering absCUFs (Fig. 2a) and synCUFs (Fig. 2b) codon frequencies. Similar plots based on EU and BC distances are given in Supplementary Figs. 2, 3. These figures suggest that for most of the environmental groups, average codon usage (absCUFs or synCUFs) distances calculated by EU, ES or BC method for within-group comparison is significantly (*P* < 0.01) lower than that for between-group comparison (Fig. 2 and Supplementary Figs. 2, 3). However, a notable exception was observed for skin samples where we did not find any significant difference (*P* > 0.5) between these two groups of distances (within versus between). This may indicate an overall functional and taxonomic similarity of skin microbes with the other groups of microorganisms (discussed in the discussion section). Overall, these results suggest that, in general, samples that share similar ecological features (i. e. collected from similar habitats) tend to show comparable codon frequencies (i.e. lower distance in their codon usage) as compared to samples from different habitats irrespective of the methods how the codon frequencies or the codon usage distances were calculated.

**Fig. 3:**
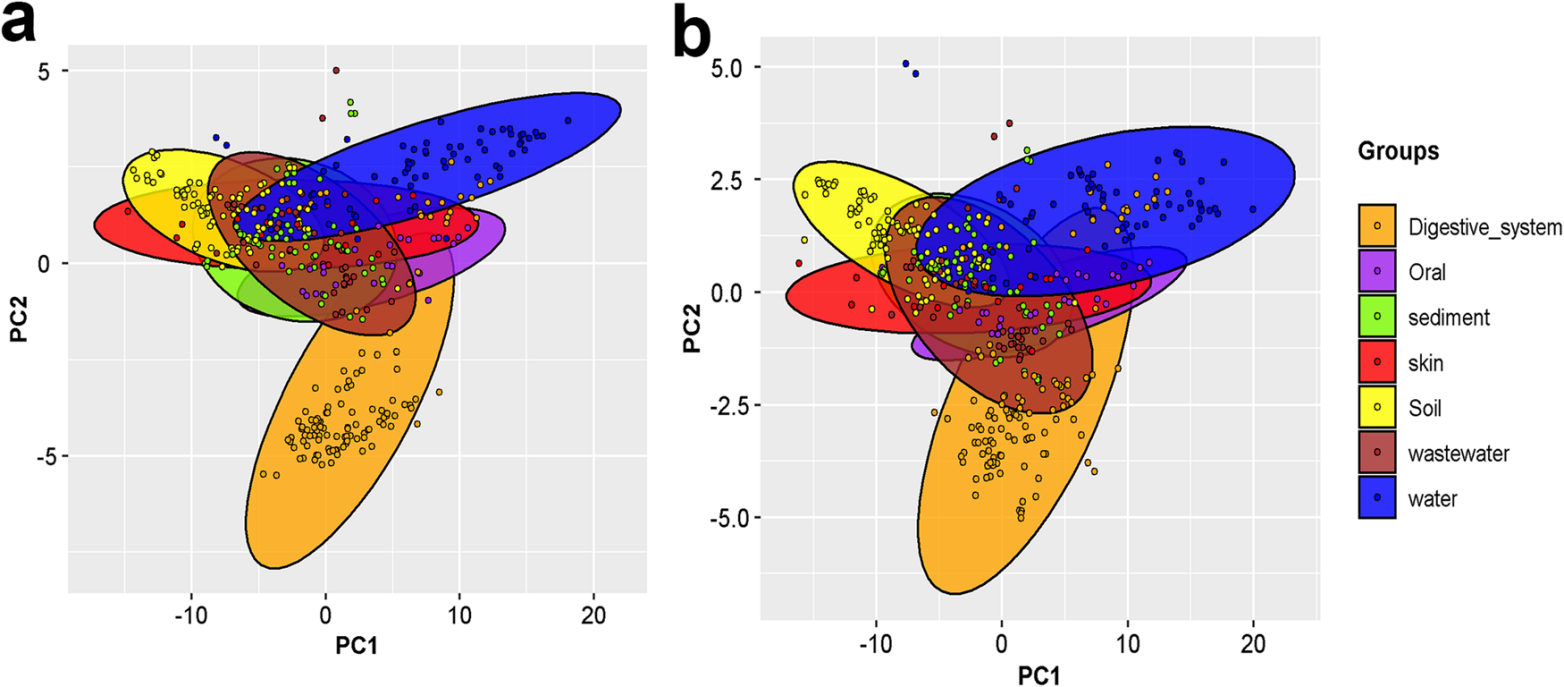
Principal component analysis (PCA) on the codon usage frequency of samples from seven environmental biomes. PCA analysis was applied on the codon usage frequencies of samples from seven selected biomes. For each sample, codon frequencies were calculated by two methods absCUFs and synCUFs (see main text). In panel **a** PCA was done based on absCUFs and in panel **b** PCA was done based on synCUFs. Each point in the figure represents one sample. Samples are colored according to the environmental feature from where those were collected. The color scheme is shown in the legend. The first two components (PC1 and PC2) of the PCA are presented here. The first two components (PC1 and PC2) explain more than 70% of the variance in codon frequencies in the dataset.

To further substantiate this observation, we applied principal component analysis (PCA) on the absCUFs and synCUFs frequencies of the samples from the seven selected biomes. This analysis suggested an overall separation of samples according to the environmental biome along the first two axes of the principal components (PC1 and PC2) for both absCUFs (Fig. 3a) and synCUFs (Fig. 3b) matrices. PC1 and PC2 together explained more than 70% of the variance of synCUFs or absCUFs among the samples. Some apparently visible trends that can be gleaned from these graphs are: (*i*) samples from most of the selected biomes formed visibly distinct clusters based on their codon frequencies (synCUFs or absCUFs) although there are substantial overlaps, (*ii*) most distinct separation was observed among the samples from the two largest communities (according to the number of samples) water and digestive system, and (*iii*) there is no well separation of sediment samples from the soil samples maybe because of the overall functional and taxonomic similarity among the microbes in these two communities^29^.

To access the statistical significance of this clustering, we applied ANOSIM (Analysis of Similarities) test to the EU distance and BC dissimilarity of codon frequencies (synCUFs and absCUFs) in a pairwise manner. ANOSIM test suggested that samples from most of the test habitats can be separated (significant *P* < 0.05 and positive R values) based on their codon frequencies, except for a few cases (Table 1). However, ANOSIM does not support the ES method; therefore, we performed another related test (permutation test) based on the ES distance method (see methods). This test implied that except for skin samples, within-group codon usage distances are indeed significantly (*P* < 0.05) smaller than between-group codon usage distances and this trend cannot be obtained just by random chance (Supplementary Table 2).

**Table 1:** ANOSIM results for codon usage distance comparisons among the samples from seven selected habitats.

Habitat1	N1	Habitat2	N2	R values for absCUFs based on Bray-Curtis dissimilarity	R values for absCUFs based on Euclidean distance	R values for synCUFs based on Bray-Curtis dissimilarity	R values for synCUFs based on Euclidean distance
Digestive system	122	Oral	27	R = 0.1858*;* *P* = 2.00E-03	R = 0.1912*;* *P* = 1.10E-03	R = 0.1561*;* P = 4.60E-03	R = 0.1883*;* P = 1.10E-03
Digestive system	122	Sediment	60	R = 0.3365*;* *P* = 1.00E-04	R = 0.3469*;* *P* = 1.00E-04	R = 0.2242*;* P = 1.00E-04	R = 0.2231*;* P = 1.00E-04
Digestive system	122	Skin	15	R = 0.5845*;* *P* = 1.00E-04	R = 0.6208*;* *P* = 1.00E-04	R = 0.4337*;* P = 1.00E-04	R = 0.4556*;* P = 1.00E-04
Digestive system	122	Soil	85	R = 0.7576*;* *P* = 1.00E-04	R = 0.7774*;* *P* = 1.00E-04	R = 0.6751*;* P = 1.00E-04	R = 0.6608*;* P = 1.00E-04
Digestive system	122	Wastewater	45	R = 0.2951*;* *P* = 1.00E-04	R = 0.3162*;* *P* = 1.00E-04	R = 0.1898*;* P = 2.00E-04	R = 0.2087*;* P = 1.00E-04
Digestive system	122	Water	68	R = 0.4165*;* *P* = 1.00E-04	R = 0.4193*;* *P* = 1.00E-04	R = 0.3597*;* P = 1.00E-04	R = 0.3842*;* P = 1.00E-04
Oral	27	Sediment	60	R = 0.3362*;* *P* = 1.00E-04	R = 0.3368*;* *P* = 1.00E-04	R = 0.3281*;* P = 1.00E-04	R = 0.3368*;* P = 1.00E-04
Oral	27	Skin	15	R = 0.3827*;* *P* = 1.00E-04	R = 0.4215*;* *P* = 1.00E-04	R = 0.2901*;* P = 2.00E-04	R = 0.3237*;* P = 1.00E-04
Oral	27	Soil	85	R = 0.7415*;* *P* = 1.00E-04	R = 0.7399*;* *P* = 1.00E-04	R = 0.7046*;* P = 1.00E-04	R = 0.6935*;* P = 1.00E-04
Oral	27	Wastewater	45	R = 0.2428*;* *P* = 1.00E-04	R = 0.2553*;* *P* = 1.00E-04	R = 0.2447*;* P = 1.00E-04	R = 0.253*;* P = 1.00E-04
Oral	27	Water	68	R = 0.0415*;* *P* = 1.38E-01	R = 0.0272*;* *P* = 2.21E-01	R = 0.0311*;* P = 1.85E-01	R = 0.0396*;* P = 1.50E-01
sediment	60	skin	15	R = 0.3615*;* *P* = 1.00E-04	R = 0.3929*;* *P* = 3.00E-04	R = 0.2888*;* P = 8.00E-04	R = 0.3367*;* P = 1.00E-04
sediment	60	Soil	85	R = 0.3135*;* *P* = 1.00E-04	R = 0.3134*;* *P* = 1.00E-04	R = 0.3013*;* P = 1.00E-04	R = 0.2941*;* P = 1.00E-04
sediment	60	Wastewater	45	R = 0.0738*;* *P* = 3.60E-03	R = 0.0827*;* *P* = 1.00E-03	R = 0.0624*;* P = 7.60E-03	R = 0.0717*;* P = 3.40E-03
sediment	60	Water	68	R = 0.3768*;* *P* = 1.00E-04	R = 0.3667*;* *P* = 1.00E-04	R = 0.3807*;* P = 1.00E-04	R = 0.3862*;* P = 1.00E-04
skin	15	Soil	85	R = 0.502*;* *P* = 1.00E-04	R = 0.5085*;* *P* = 1.00E-04	R = 0.4354*;* P = 1.00E-04	R = 0.4419*;* P = 1.00E-04
skin	15	Wastewater	45	R = 0.3093*;* *P* = 3.00E-04	R = 0.3341*;* *P* = 4.00E-04	R = 0.2612*;* P = 5.00E-04	R = 0.3117*;* P = 2.00E-04
skin	15	Water	68	R = 0.3738*;* *P* = 1.00E-04	R = 0.3997*;* *P* = 1.00E-04	R = 0.3266*;* P = 1.00E-04	R = 0.3516*;* P = 1.00E-04
Soil	85	Wastewater	45	R = 0.4369*;* *P* = 1.00E-04	R = 0.4381*;* *P* = 1.00E-04	R = 0.4162*;* P = 1.00E-04	R = 0.4127*;* P = 1.00E-04
Soil	85	water	68	R = 0.7046*;* *P* = 1.00E-04	R = 0.6968*;* *P* = 1.00E-04	R = 0.7013*;* P = 1.00E-04	R = 0.6997*;* P = 1.00E-04
wastewater	45	Water	68	R = 0.2847*;* *P* = 1.00E-04	R = 0.2854*;* *P* = 1.00E-04	R = 0.291*;* P = 1.00E-04	R = 0.2971*;* P = 1.00E-04

For each sample, codon usage frequencies were calculated by two methods absCUFs and synCUFs (see main text). ANOSIM test was carried out considering the codon usage frequencies (absCUFs and synCUFs) of samples from two habitats at a time using Euclidean distance and Bray-Curtis dissimilarity matrices separately with 10,000 permutation values. Therefore, there are two columns for the R-values (one for Euclidean distance and another for Bray-Curtis dissimilarity). Here N_1_ and N_2_ represent the number of samples in the first and second habitat, respectively. ANOSIM test statistics are shown with R values and significance levels are shown with *P*-values.

### Distinct amino acid usage among the samples from similar environmental biome

Considering their natural abundance from various environmental determinants earlier it was suggested that in each environment AAs are distributed at a specific ratio that is characteristic of the environment^5^. Therefore, next, we analyzed whether there is any environment-specific signature in the AA usage as we found for codon usage. When we compared relative AA usage distances among the samples from the same environmental group to that of samples from different environmental groups (within-group versus between-group comparisons), we found similar trends as we found in Fig. 2 for codon usage (Supplementary Fig. 4). Particularly, this test suggested that for most of the biomes distances in AA usage among the samples from the same habitat are significantly (*P* < 0.01) lower than that among the samples from other habitats (Supplementary Fig. 4). Consequently, PCA analysis based on AA frequencies also revealed distinct clustering of the samples separated according to the habitat along the first two principal components PC1 and PC2 (Supplementary Fig. 5). When we compared the AA frequencies among the samples from the seven environmental biomes in a pairwise manner, both the ANOSIM (for EU distance and BC dissimilarity) and permutation test (for ES distance) showed that the AA composition of samples from most of the biomes is significantly (R > 0 and *P* < 0.05) different from that of any other biome (Supplementary Table 3, 4). Taken together, it may be inferred from these results that the abundance of AA can create habitat-specific signatures similar to what we noticed earlier for codon frequencies.

### Observed codon/amino usage patterns are robust to the variation in the dataset

Gene prediction in metagenomic samples highly depends on the algorithms used for sequence processing and prediction. Specifically, the assembly step could drastically impact every aspect of predicted sequences, including their codon usage^30^. Therefore, for each sample, we collected CDS and protein sequences from the MGnify metagenomic database^31^ that are predicted from the same Fastq read file(s), however, by a completely different approach. The basic difference between the CDSes predicted by our approach with the CDSes predicted by this database is that we predicted CDSes from the assembled contigs whereas the database predicted CDSes from the merged reads without any assembly step. When we calculated codon/AA usage distances based on the sequences collected from the MGnify metagenomic database, we noticed similar trends as we found considering the sequences predicted by our pipeline. For instance, PCA and ANOSIM tests (for EU distance and BC dissimilarity) reflected similar trends as we noticed before considering sequences predicted by our metagenomic pipeline (Supplementary Fig. 6, Supplementary Table 5). These results altogether further substantiate that microbes from similar environmental biomes tend to show comparable codon and AA usage frequencies as opposed to the samples from different environmental biomes and this trend does not depend upon the methods of CDS prediction or upon the fact whether the sequences were predicted from assembled sequences or not.

Sequencing depth is another important aspect that can significantly impact the metagenomic read processing steps and hence their genetic contents^32^. To simulate how sequencing at different depths may impact our results, we considered different subsets of reads (0.1%, 1%, 5%, and 10% of reads) from each sample and checked the replicability of the trends considering CDS/AA sequences predicted from the assembly of those reads. Our results suggested consistent patterns of codon or AA usage among all the subsets, similar to the patterns we noticed in our main dataset (Supplementary Figs. 7-9 and Supplementary Table 6). Overall, these results imply that sequences identified at any resolution show similar patterns in their codon/AA usage, irrespective of the depth at which they were sequenced.

Since different sequencing technologies have different error patterns it is necessary to check whether samples sequenced by each method show similar patterns or not. However, most of the samples (>85 %) in this analysis were sequenced by the Illumina method and only a few samples were sequenced by other methods such as Ion Torrent or pyrosequencing (<20 samples for each method), which are not sufficient for reliable statistical tests. Considering this limitation of the dataset, we grouped the samples sequenced by all non-Illumina methods together (a total of 31 samples from three environmental biomes: digestive system, soil, and water) and categorized our dataset into two groups (i) those are sequenced by the Illumina method and, (ii) those are sequenced by non-Illumina methods. By repeating our analysis for both these two groups of samples separately, we noticed overall similar patterns in their codon and amino usage, which are highly consistent with the trends that we noticed for our main analysis. Results for pair-wise ANOSIM tests (Supplementary Table 7, 8), density plots (Supplementary Figs. 10-15), and PCA analyses (Supplementary Figs. 16, 17) for these two groups of samples are provided in the supplementary information.

Essential genes were suggested to be more conserved and to retain their functional characteristics in long-term evolution than the non-essential genes^33^. This may suggest that essential genes of microorganisms would show more distinct trends in their codon usage patterns than the non-essential genes. To test this hypothesis, we identified potential ribosomal protein-coding genes in the test metagenomic samples (see materials and methods) and conducted PCA and pairwise ANOSIM test considering the codon usage frequencies (absCUFs and synCUFs) of only these ribosomal protein-coding genes in each sample. Our study suggested that samples from the test biomes can be well separated into their habitats based on the codon usage of these genes only (Supplementary Fig. 18, Supplementary Table 9).

### Correlations between codon usage distances and distances in GC content, k-mer frequencies, functional and taxonomic abundance

GC content is regarded as one of the strongest predictors of microorganisms’ codon and AA usage^34,35^. Thus it is probable that the resemblance that we observed in the codon or AA usage of the samples from the same habitat may be due to their proximity in GC-content instead of direct selection for codon or AA usage. To verify its impact, we checked how codon and AA usage distances are correlated with the distances in GC content among the samples. Significant correlations (ρ = 0.695‒0.977; *P* < 1 × 10^−6^) were noticed considering samples habitat-wise (i.e. considering samples from each habitat separately) or taking all samples together, suggesting that GC content may be a contributing factor in shaping the codon usage distances among the samples (Supplementary Table 10).

k-mer frequency is another important variable that was shown to strongly correlate with codon usage frequencies^36^. To test how k-mer frequencies could influence the codon and AA usage distances of our test samples we estimated frequencies of different short k-mers starting from k = 2 to k = 10 (except k = 3) (details in material and methods) and correlated the distances in k-mer usage frequencies with our previously estimated codon/AA usage distances among the test samples. Our study suggested significant and strong correlations (ρ = 0.4828‒0.9759; *P* < 1 × 10^−6^) between these variables suggesting that k-mer frequencies are important correlates of codon/AA usage distances (Supplementary Table 11); such correlations are expected as there is (by definition) overlap between the definitions of k-mer and codon frequencies. However, the correlations suggest that not all the variability in the codon usage can be explained by the k-mers.

Based on small-scale studies of few metagenomic samples, previously it was proposed that microbes from related environments show overall similar functional profiles^37^. Besides, a universal correlation has also been proposed between functional specificity and codon usage of genes^38^. Thus, it is probable that the similarities we noticed in the codon usage profile may have been caused by the functional similarities among the communities. To test this probability, first, we checked whether functional resemblance among the similar environmental communities, as has been reported before in few selected samples, is also reflected in our test habitats and next tested if any correlation exists between the codon usage distances and functional distances among the samples. Considering the relative abundance of selected GO terms from each type of GO ontologies (BP, CC, and MF) we found that indeed there are overall similarities in their abundance among the samples from similar habitats. Our PCA analysis for samples from the seven habitats revealed visibly distinct clustering of samples from similar habitats based on GO MF or GO BP terms’ frequencies (Supplementary Fig. 19). Next, we calculated distances in the functional profile among the samples considering the relative abundances of selected GO terms from three types of ontologies (MF, BP, and CC) separately. Correlating the distances in functional profiles with the codon usage distances of the samples, strong and significant correlations were noticed, although the strengths of correlations (ρ = 0.172‒0.726; *P* < 0.05) were not uniform across all habitats (Supplementary Table 12). Overall, these results indicated codon usage distances among the samples may have been shaped by their functional similarities, although the extent of this relation is not the same in all communities. In this context, it may be relevant to ask whether the patterns that we observed in the codon usage are specific to proteins from any functional category or universal to all functional groups. To dig deeper into this aspect we classified the CDS sequences of each test sample into the 23 COG (Clusters of Orthologous Groups) functional categories (see materials and methods). Next, we conducted ANOSIM tests for codon usage frequencies (absCUFs and synCUFs) considering the sequences under each functional category independently. Results revealed similar trends in all the functional categories we tested here which suggested that the trends that we observed are universal to proteins from all functional groups; at least for the groups we tested here (Supplementary Table 13).

At the next step, we analyzed how the diversity in the taxonomic composition may impact the codon usage distances among the samples. For this, we calculated distances among the samples based on the relative abundance of different taxons under four taxonomic ranks (phylum, order, family, and genus) individually (see methods). When we correlated the taxonomic distances of our test samples with their codon usage (absCUFs and synCUFs) distances, codon usage distances were found to be significantly correlated (ρ = 0.112‒0.360; *P* < 0.05) with the taxonomic distances calculated based on the relative abundance of taxons under each of the four taxonomic ranks (Supplementary Table 14).

### Independent impacts of different variables on the AA and CUB distances

The results presented in the previous sections implied that several attributes, such as distances in AA usage frequencies, k-mer frequencies, GC content, functional abundance, and taxonomic abundance, could significantly regulate the codon usage distances among the samples. The pronounced effect of each of these variables prompted us to excavate their relative contribution in shaping codon usage distances. To this end, we performed partial correlation analyses between codon usage distances (absCUFs and synCUFs), and distances for each of these variables while controlling the distances for all other variables. The results delineated in Table 2 represent that each of these variables can independently control the codon usage distances; however, the strength of the correlations is weak either for the distances calculated by ES or BC method for most of the variables, except for the impact of GC content. Specifically, we did not find any strong independent influence of any variable related to distances in taxonomic abundance (partial correlation < 0.15; *P* < 1 × 10^−6^) or different k-mer frequencies (partial correlation < 0.31; *P* < 1 × 10^−2^) on the codon usage distances. Further, our observations suggested that although distances in GC content (partial correlation > 0.630; *P* = 0 for EU distance) is the most influencing variable, functional abundance specifically distances in GO BP or GO MF (partial correlation > 0.34; *P* < 1 × 10^−6^ for BC dissimilarity) abundance can significantly affect codon usage distances. We performed the same analysis to underscore the features that may regulate AA distances among the samples independently. In this case, our results suggested that, instead of distances in GC content (partial correlation < 0.26; *P* < 1 × 10^−6^), distances in codon usage (partial correlation > -0.30; *P* < 1 × 10^−6^), followed by distances in k5-mer frequency (partial correlation = 0.298; *P* < 1 × 10^−6^ for EU distance) has considerable impacts over AA distances (Supplementary Table 15). However, all these correlations are relatively weak, implying that there may be other factors that control the variations of AA distances among the samples.

**Table 2:** Partial correlation analyses between the distances in codon usage frequencies and distances of all other variables.

Test variable	Control variables	Partial correlation with distances in absCUFs frequency (distances calculated by Euclidean distance method)	Partial correlation with distances in absCUFs frequency (distances calculated by Bray-Curtis dissimilarity method)
AminoAcid frequency	GO-BP & GO-CC & GO-MF & family & genus & order & phylum & GC-distance & k2-mer & k4-mer & k5-mer & k6-mer & k7-mer & k8-mer & k9-mer & k10-mer	ρ = −0.3*;* *P* = 0.00E + 00	ρ = −0.394*;* *P* = 0.00E + 00
GO-BP	GO-CC & GO-MF & family & genus & order & phylum & GC-distance & k2-mer & k4-mer & k5-mer & k6-mer & k7-mer & k8-mer & k9-mer & k10-mer & AminoAcid	ρ = 0.088*;* *P* = 1.73E−153	ρ = 0.352*;* *P* = 0.00E + 00
GO-CC	GO-MF & family & genus & order & phylum & GC-distance & k2-mer & k4-mer & k5-mer & k6-mer & k7-mer & k8-mer & k9-mer & k10-mer & AminoAcid & GO-BP	ρ = 0.19*;* *P* = 0.00E + 00	--------
GO-MF	family & genus & order & phylum & GC-distance & k2-mer & k4-mer & k5-mer & k6-mer & k7-mer & k8-mer & k9-mer & k10-mer & AminoAcid & GO-BP & GO-CC	ρ = 0.037*;* *P* = 7.36E−28	ρ = −0.342*;* *P* = 0.00E + 00
Family	genus & order & phylum & GC-distance & k2-mer & k4-mer & k5-mer & k6-mer & k7-mer & k8-mer & k9-mer & k10-mer & AminoAcid & GO-BP & GO-CC & GO-MF	ρ = 0.017*;* *P* = 2.44E−07	ρ = 0.029*;* *P* = 1.06E−17
Genus	order & phylum & GC-distance & k2-mer & k4-mer & k5-mer & k6-mer & k7-mer & k8-mer & k9-mer & k10-mer & AminoAcid & GO-BP & GO-CC & GO-MF & family	ρ = 0.101*;* *P* = 4.12E−200	ρ = −0.001*;* *P* = 8.10E−01
Order	phylum & GC-distance & k2-mer & k4-mer & k5-mer & k6-mer & k7-mer & k8-mer & k9-mer & k10-mer & AminoAcid & GO-BP & GO-CC & GO-MF & family & genus	ρ = 0.101*;* *P* = 1.64E-198	ρ = 0.037*;* *P* = 5.10E−28
Phylum	GC-distance & k2-mer & k4-mer & k5-mer & k6-mer & k7-mer & k8-mer & k9-mer & k10-mer & AminoAcid & GO-BP & GO-CC & GO-MF & family & genus & order	ρ = −0.147*;* *P* = 0.00E + 00	ρ = −0.12*;* *P* = 1.57E−281
GC-distance	k2-mer & k4-mer & k5-mer & k6-mer & k7-mer & k8-mer & k9-mer & k10-mer & AminoAcid & GO-BP & GO-CC & GO-MF & family & genus & order & phylum	ρ = 0.613*;* *P* = 0.00E + 00	ρ = 0.306*;* *P* = 0.00E + 00
k2-mer	k4-mer & k5-mer & k6-mer & k7-mer & k8-mer & k9-mer & k10-mer & AminoAcid & GO-BP & GO-CC & GO-MF & family & genus & order & phylum & GC-distance	ρ = 0.09*;* *P* = 3.27E−158	ρ = 0.038*;* *P* = 1.71E−30
k4-mer	k5-mer & k6-mer & k7-mer & k8-mer & k9-mer & k10-mer & AminoAcid & GO-BP & GO-CC & GO-MF & family & genus & order & phylum & GC-distance & k2-mer	ρ = 0.1*;* *P* = 1.36E−196	ρ = 0.194*;* *P* = 0.00E + 00
k5-mer	k6-mer & k7-mer & k8-mer & k9-mer & k10-mer & AminoAcid & GO-BP & GO-CC & GO-MF & family & genus & order & phylum & GC-distance & k2-mer & k4-mer	ρ = 0.308*;* *P* = 0.00E + 00	ρ = 0.005*;* *P* = 1.03E−01
k6-mer	k7-mer & k8-mer & k9-mer & k10-mer & AminoAcid & GO-BP & GO-CC & GO-MF & family & genus & order & phylum & GC-distance & k2-mer & k4-mer & k5-mer	ρ = −0.197*;* *P* = 0.00E + 00	ρ = 0.097*;* *P* = 7.38E−184
k7-mer	k8-mer & k9-mer & k10-mer & AminoAcid & GO-BP & GO-CC & GO-MF & family & genus & order & phylum & GC-distance & k2-mer & k4-mer & k5-mer & k6-mer	ρ = −0.011*;* *P* = 8.20E−04	ρ = −0.143*;* *P* = 0.00E + 00
k8-mer	k9-mer & k10-mer & AminoAcid & GO-BP & GO-CC & GO-MF & family & genus & order & phylum & GC-distance & k2-mer & k4-mer & k5-mer & k6-mer & k7-mer	ρ = 0.08*;* *P* = 2.76E−126	ρ = 0.105*;* *P* = 2.55E−214
k9-mer	k10-mer & AminoAcid & GO-BP & GO-CC & GO-MF & family & genus & order & phylum & GC-distance & k2-mer & k4-mer & k5-mer & k6-mer & k7-mer & k8-mer	ρ = 0.021*;* *P* = 2.73E−10	ρ = 0.049*;* *P* = 1.18E−48
k10-mer	AminoAcid & GO-BP & GO-CC & GO-MF & family & genus & order & phylum & GC-distance & k2-mer & k4-mer & k5-mer & k6-mer & k7-mer & k8-mer & k9-mer	ρ = −0.074*;* *P* = 2.90E−108	ρ = −0.05*;* *P* = 4.94E−50

Partial correlations were conducted between two variables controlling the effects of all other variables where the one variable is distances in codon usage frequencies (absCUFs) and the other variable is either distances in GC content or amino acid usage or k-mer frequencies or distances in GO terms’ abundance or in taxonomic abundance. Distances in Gene Ontology (GO) terms’ frequencies among the samples were calculated considering the relative abundance of 500 GO biological process, 500 GO molecular function, and 100 GO cellular component terms separately. Taxonomic distances among the samples were calculated considering relative abundance of selected taxons under four taxonomic ranks (order, phylum, family, genus) separately (see main text). All the distances were calculated following two methods, (i) Euclidean, and (ii) BC dissimilarity methods. Correlation values were tested considering the distances calculated by each of these two methods separately. Therefore, there are two columns for correlation values (one for Euclidean distance and another for Bray-Curtis dissimilarity). Here ρ stands for correlation coefficient and significance levels were shown with *P*-values.

To better quantify the contribution of each of these variables in shaping codon/AA distances, we performed linear regression considering codon/AA usage distances as dependent variables and distances for all other variables as predictor variables. The selected variables based on the chosen (linear) model can explain more than 80% of the variation in the codon or AA usage distances (R^2^ > 0. 80). From the values of the regression coefficient (β), it could be inferred that distances in GC content can significantly explain codon usage distances (β = 0.494, 0.233; *P* < 1 × 10^−6^ for EU distance and BC dissimilarity respectively) (Supplementary Table 16). Further, it may be noted that although GC content distance can account for a significant fraction of the variance in codon usage distances, it has little impact (β = 0.419 and 0.127; *P* < 1 × 10^−6^ for EU distance and BC dissimilarity, respectively) as compared to the effect of codon usage distances (β = -0.609; and -0.854; *P* < 1 × 10^−6^ for EU distance and BC dissimilarity respectively) over AA usage distances (Supplementary Table 17). Here it may be noted that we also found some dispersed effect of different k-mer frequencies on the codon or AA distances, however, we think this is an artifact as we did not find any consistent trend between the β values obtained by the EU distance and BC dissimilarity methods. Overall, these results implied that the patterns that we observed in codon usage distances have been significantly shaped by the distances in GC content besides, the weak impact of AA usage distances. However, none of these parameters can completely explain the variation of AA distances and there is still unexplained variability related to CUB distance that can’t be explained by the other variables and may be related to direct selection.

### Further analysis on the impact of GC content on codon usage patterns

The results presented in the previous section suggested a strong influence of GC content on codon usage distances of microbial communities derived from different habitats. To delve deeper into this aspect, we compared the codon usage distances among the samples where the impact of GC content is expected to be comparable, *i.e*. having similar GC content. For this, we grouped the samples into GC bins based on their overall GC content, then in each such GC bin we compared the codon usage distances among samples from different habitats using the ANOSIM test in the same way as we did before without considering any GC bin. Thus, we noticed a similar trend in each GC bin that samples from similar habitats tend to show lower codon usage distances than the samples from different habitats (Supplementary Table 18). This suggests that although there may be significant correlations, the codon usage of samples can’t be explained simply from their GC content. To further underscore the impact of GC content, we generated random CDS sequences that preserve not only GC content but also other factors, such as AA encoding and dinucleotide frequencies of each real sequence that may modulate codon usage (see methods). If the trend is only because of GC content (or any of these factors) we would expect a comparable level of dissimilarity in the codon usage among the samples from different habitats for random datasets as of real dataset. When we accessed the dissimilarities using ANOSIM tests, for most of the habitats the R values obtained based on absCUFs or synCUFs frequencies of random datasets were lower than that we found for similar tests using absCUFs or synCUFs frequencies of the real dataset, respectively (Supplementary Table 19). This implies that GC content alone cannot explain the codon usage trend. However, this test also revealed that when GC content and dinucleotide frequencies remain unchanged, the codon usage of random sequences can discriminate the samples from different habitats, although with lower R values, suggesting these parameters may have non-negligible effects over the observed trends.

### Environmental samples show selection for codon usage bias

**Fig. 4:**
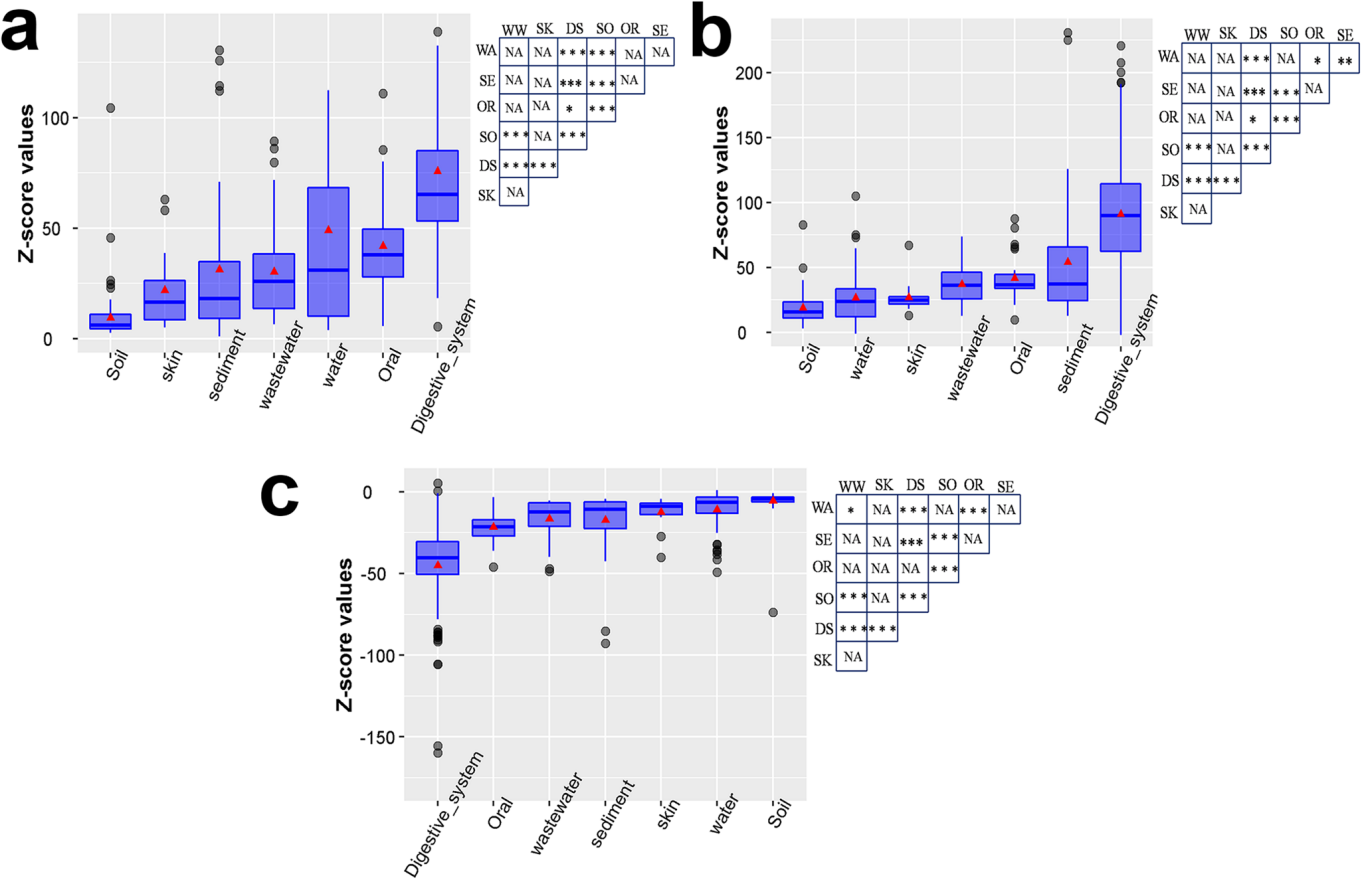
Average Z-score for CAI, ENC, and DCBS values of samples from seven selected habitats. This figure shows the Z-scores calculated for three codon usage indices (CAI, ENC, and DCBS) of samples from each selected biome in the form of box plots. Panels **a**, **b**, and **c** represent Z-scores for CAI, DCBS, and ENC values respectively. Z-score values were calculated comparing average CAI/EN/DCBS values of real and random sequences of each sample. Each box represents Z-scores of samples derived from a specific habitat. Horizontal line within a box represents the median value, whereas its upper and lower edges represent 25% and 75% of the data, respectively. The average of Z-scores of all samples from each habitat is shown with a red triangle. In each panel, niches were arranged from lower to higher average values of Z-scores. Statistical significance for pairwise comparison of average values (of codon usage indices) among the habitats was shown in adjacent panels. *P*-values were calculated by Wilcoxon signed-rank test and adjusted for multiple comparisons using Bonferroni correction. *P*-values for the pairwise comparisons are shown by abbreviating the group labels as WA-Water; SE-Sediment; OR-Oral; SO-Soil; DS-Digestive system; SK-Skin; WW-Wastewater. *P*-values *<0.05; **<0.01; ***< 1 ×10^−6^. In each panel, boxes are arranged according to the median values of Z-scores.

To get a relative estimate of selection that may shape CUB at the community level, we compared the average codon usage indices Codon Adaptation Index (CAI)^39^, Effective Number of Codons (ENC)^40^, and Directional Codon Bias Score (DCBS)^41,42^ of each sample with that of corresponding randomized sequences through Z-score approach (see methods). For indices such as CAI and DCBS a positive Z-scores and for ENC (where lower values suggest higher bias) negative Z-score indicates higher selection for CUB in real sequences than random sequences. Here we used the same sets of random sequences that preserve overall di-nucleotide frequencies (hence GC content) and protein-encoding of each real sequence thus any deviation from the random values are likely to be because of selection (or exclusion) for specific sets of codons among real sequences than because of selection for the controlled factors (GC content, di-nucleotide frequencies, and AA usage). We found a general trend in Z-scores when we ranked different habitats according to the average Z-scores of all samples. Specifically, samples from the host-associated environment such as the digestive system or oral samples consistently demonstrated a higher extent of selection for codon bias (*i.e*. higher Z-scores for DCBS, CAI values, and lower Z-scores for ENC values) than most of the environmental samples (Fig. 4). In contrast, soil and water samples showed a consistently lower extent of selection (lower Z-scores for DCBS, CAI values, and higher Z-scores for ENC values) as compared to samples from most of the other habitats (Fig. 4). Interestingly, skin samples, although host-associated, were found to exhibit an intermediate level of selection for codon bias, in between the above extreme cases (Fig. 4). Overall, these results indicated host-associated samples show higher extent selection for codon bias than the other samples although there are exceptions (discussed in the discussion section).

## Discussion

The genomic architecture of microorganisms bears telltale signs of their long evolutionary histories which are mainly driven towards adaptation to different environmental conditions^10^. Therefore, genome fabrics of microorganisms are thought to bear signatures specific to their lifestyle. Thus it is often found that microbes from distant lineages but dwelling in similar habitats exhibit related genomic/proteomic properties than the phylogenetically close microbes from different habitats^10^. Studies of codon or AA usage of the whole community can shed light on the evolutionary mechanisms that microbes acclimatized to adapt with the ever-changing environment. Therefore, in this study, we analyzed the impact of habitat and microhabitat on the codon and AA usage of different microbial communities and explored their interlink with various factors such as GC-content, k-mer frequencies, and taxonomic and functional similarities.

Considering a vast number of metagenomic samples from seven different environmental biomes here we first showed that there exist distinct trends in the codon usage among the microorganisms from different habitats. Particularly, our principal component analysis and related statistical tests based on codon usage frequencies of samples from seven environmental biomes (water, wastewater, soil, sediment, digestive system, and human oral microbiome) suggested that except few samples, microorganisms from each habitat bear specific signatures in their codon usage that can segregate them according to ecological niche from where they were collected. The exceptional cases where codon usage cannot discriminate samples from different biomes were mainly from skin samples. In this case, we found a high level of diversity in codon usage within a biome than between biomes perhaps because of their environmental relatedness with other samples. Except for these few cases, our analysis emphasized that metagenome from each habitat do have distinct signatures in their codon usage that is significantly different from that of other habitats. However, signs of environmental signatures on codon usage have already been reported in a number of previous studies^1–4,8,9^. Therefore, let us try to critically access the significance of the results that we presented here in light of earlier studies.

By analyzing the relative codon usage pattern of more than 700 prokaryotes from different habitats earlier Botzman and Margalit noted a correspondence between codon usage of microorganisms and their habitats^2^. Specifically, organisms that can live in multiple habitats were shown to exhibit higher CUB than the microbes that can populate in some specialized habitats^2^. Several other studies also ascertained that microbes from different niches show the characteristic distribution of codon usage by which it is possible to group them according to their habitats. However, all these speculations are based on the aggregated trait of species-level studies considering only available genome sequences. In their natural habitats microorganisms exist like a connatural soup of organisms intercommunicating with each other. Those studies not only ignored the influence of community structure but also overlooked the codon (or AA) usage of the majority of microorganisms that exist only in natural habitats. Thus species-level studies are not expected to provide the realistic trend of a trait that microorganisms inherit within their community. Further, the concept of microbial species has been questioned repeatedly leading to doubt if there is any microbe that ever exists as a distinct species. Therefore, it is necessary to inspect the codon usage pattern of microorganisms in a holistic manner throughout the community rather than of individual species. Considering this, previously few attempts have been taken to investigate codon usage of microbes at the community level^3,4^. However, these studies suffered from the paucity of datasets (that were available when the study was performed) calling the generality of their results into question. Therefore, in this study, we considered a large number of metagenomic samples from a diverse array of biomes which made it unique in the breadth and depth of the analysis.

Perhaps the most interesting and significant result that we found here is intra-biome similarity rather than inter-biome similarity. To understand whether there is any community-specific bias in the codon preference, previously Roller et al., compared the codon usage variability of genes from the same metagenome to that of genes from other metagenomes considering altogether 11 metagenomic samples^3^. Their study provided interesting insights that genes from a metagenome exhibit a common preference for codon usage than the genes from other metagenomes irrespective of their phylogenetic diversity. Within a community, microorganisms can share their genetic information; thus this inter-metagenome similarity is attributed due to the factors such as horizontal gene transfer or shared translational machineries^3^. If there is any direct impact of environment on the codon usage, we could expect similarity not only within a metagenome but also among the genes from different metagenomes but from similar environmental biomes. Therefore, in this study, we investigated how the codon usage of metagenomes varies between communities rather than within a community. Thus our study provided a novel insight that the similarity in the codon usage is not restricted only to the genes from a metagenome but genes from similar types of biomes in general show an overall similar codon usage pattern. In most of the cases, the samples that we considered under different environmental categories vary extensively in their host or geo-location, or collection time. Therefore, the hypotheses of shared translational machinery or extensive gene transfer within a community are not enough to explain the intra-biome similarities in the codon usage that we observed in this study (although those maybe a part of the process). Thus our results, on the first glimpse, point towards the direct influence of the environment on this pattern.

As codon usage, an environment-specific trend has also been deciphered in the relative frequencies of different AAs. Estimating their relative abundance from various environmental determinants, earlier it was demonstrated that in each habitat different AAs occur in a constant ratio which changes across environmental gradients^5^. Niche-specific variation in AA frequencies has been noted by a number of other studies highlighting its adaptive consequences^6,7^. However, to the best of our knowledge, no study has ever analyzed the AA usage of metagenomic samples at a large scale. Overall our study suggested that there is a substantial signal at AA distribution similar to the signal that we found considering codon usage at the community level. Specifically, the PCA analysis and ANOSIM test implied that AA frequencies of microorganisms can also create signatures that can predict their environmental origin.

Although our results primarily showed that codon and AA usage of microorganisms carries signals specific to each habitat, however, it is worth noting that several other factors can influence these trends. Here we first checked how the gene prediction steps could modulate our results. For this, we considered CDS and AA sequences predicted following a completely different approach, specifically from merged reads without any assembly step. Further, we also predicted sequences from different subsets of all reads in each sample. Our detailed analysis implied that sequences sampled at any depth or predicted by any approach show similar patterns in their codon/AA usage suggesting their minor impact on the observed trends if any. Further, our analysis suggested that samples sequenced by the Illumina method and the other nonIllumina based methods overall show similar patterns in their codon and AA usage implying that the sequencing platforms may not have significant impacts on the observed trends.

Strong and significant differences in the codon usage among the samples from different habitats were also observed considering the probable ribosomal-protein encoding genes in each sample. Ribosomal proteins, a class of conserved genes, are the primary component of ribosomes that translate genetic information from mRNAs into proteins^43^. Despite their functional universality, some ribosomal proteins were suggested to play role in the environmental adaption of different organisms by forming environment-specific modules with specialized functions^43^. An environment-specific trend in the codon usage of these proteins may suggest environmental impacts on their evolution.

It is generally accepted that metagenomes derived from similar habitats are related in terms of their functional and/or taxonomic distribution^44,45^. On the other hand, a general convergence has been noted in the codon usage of genes encoding similar functions suggesting a universal correlation between these two variables^38^. GC content is another crucial variable that can shape the codon usage pattern of microorganisms just like gene expression^7,34,35,46^. Recent studies suggested that despite their compositional diversity environmental samples show distinctive distributions in their oligonucleotide signature^36,47^. Consequently, k-mer-based comparison of sequence signatures gained wide popularity to evaluate relationships between different metagenomic samples^47^. This evidence of profound associations prompted us to test whether the signatures that we observed at the codon or AA level can be explained from independent or mutual interactions of any or all of these variables (GC content, k-mer frequencies, taxonomic and functional abundance). To obtain a representative and quantitative estimation of their relative contributions, we correlated codon usage distances among the samples with the distances calculated for each of these variables followed by linear regression analysis. Although strong and significant correlations were found between the distances in codon usage frequencies and that of each of these variables, by factoring out the impact of GC, the effects of other factors were found to be significantly diminished suggestive of their poor independent predictive power. Particularly, parameters related to distances in taxonomic abundance (i.e. distances calculated based on family, phylum, order, or genus abundance) also showed nearly negligible independent impacts. This may indicate that although the taxonomic composition of the test samples is merely a factor that can independently influence their codon usage distances. However, it may be relevant to emphasize that with the best of our efforts we can’t find reliable taxonomic information for a significant fraction of sequences in each test sample that may have set a caveat in these results. Here we found some dispersed effect of distribution of different k-mers, however, we think these results are due to artifacts since the regression coefficients are not always in the expected range. Overall, our regression analysis suggested that GC content alone can explain most of the codon usage variability among the samples. Thus our study supported earlier observations that GC is one of the strongest determinants of codon usage^7,34,35,46^. The key question then is whether the patterns that we observed at the codon level are purely a secondary effect of similarity in their GC content? When we controlled the impact of GC by grouping the samples into GC bins, we found similar results in almost all bins as we found without considering any GC bin suggesting there may be other factors inherent to the samples, which may be an environmental effect. To consider this aspect in more detail, we generated random sequences by keeping the GC content, dinucleotide frequencies, and AA encoding of the real sequences unchanged. Thus when the impact of GC (and of these factors) is comparable, any significant trend at the codon level is expected to be related to their codon frequencies directly rather than because of these factors. Note that the random sequences generated by this way are constrained by various requirements (as mentioned above) thus the codon frequencies of random sequences were highly correlated with that of their real counterpart. Therefore, we found an overall resemblance in the codon usage pattern of random sequences with that of real sequences. Despite this, for most of the habitats, we found higher segregation of samples (i.e. higher ANOSIM R values) according to their habitat in the real dataset as compared to random datasets implying the observed variations in codon usage among the samples cannot be solely explained from the differences in their GC or AA content. As these signatures cannot be entirely explained considering the variation in GC content or AA distribution, we conclude that there may be other inherent factor or factors related to their environment that drive the observed codon usage trend. Nevertheless, environmental has documented impacts on microbial GC content^46,48,49^. Although the exact mechanisms by which the environment may influence the GC content of inhabiting microorganisms are not clear, both mutational and selective processes were considered responsible^46,48,49^. GC content of microorganisms was shown to evolve in response to various environmental stimuli such as temperature, atmospheric oxygen, or exogenous entities for adaptive benefits^46,48,49^. Although no direct relationship was found, it was proposed that the environment can trigger certain kinds of mutations that can alter the GC content of microorganisms in a specific direction^46^. The impact of GC on the codon usage that we found here may be because of subtler effects of environment. It may also be possible that the effects of the environment on GC may have been driven, at least by part, by it’s downstream effect on codon usage. We currently cannot resolve how much the effect of GC on codon usage has been driven by environmental factors; however, our study provided an important quantitative baseline for further assessment.

Since we found similar patterns at the nucleotide and AA levels it is important to delineate the forces acting at these two levels. The trends at the codon level could be a byproduct of selection at the amino acid level or vice-versa. Previously we discussed that the observed variations in the codon usage cannot be explained from their amino acid distribution only (if so, we would find comparable ANOSIM R-values for real and random sequences). Therefore, codon usage attributes cannot be solely linked with the constraints acting at the amino acid level. However, here we don’t have any clear answer to what extent selection at the codon level can modify amino acid choice. Bias at the nucleotide level was considered to incur strong impacts at the AA level rather than vice-versa^50^. A third alternative possibility is that a third variable may strongly modulate both codon and AA choice resulting in a strong correlation between the first two. Development of approaches that can delineate forces acting on strongly related variables is challenging and sometimes impossible. Considering the consistent trends after controlling for several potential constraints we believe that the co-incidence in the codon and amino acid usage pattern is related to the influence of environment.

Previous studies have discussed the possible ways how within a community microbes can have similar codon usage patterns^3,4^. Here we discuss some probable (not mutually exclusive) biological explanations of how microbes collected from distant locations but from similar types of habitats can show similar trends in their codon or AA usage.

### Shared evolution

Microorganisms derived from similar environmental backgrounds may show a common preference for codon/AA usage because of their shared evolutionary history. Let us consider an example of the gut microbiome. Gut microbes of humans and most of the other apes are speculated to be inherited from their common ancestor(s) rather than have acquired from the environment^51^. According to this estimation, the symbiosis of gut microbiomes in hominids may have originated at least 15 million years ago at any time between the common ancestors of all apes to the common ancestors of all vertebrates and co-evolved for millions of years with the host genomes^51^. Now if codon or AA preference of gut microbiome have evolved at any of the ancestor lineages and selected over evolution, decedents of that ancestor lineage may show a common pattern of codon or AA usage irrespective of their physical distances.

### Parallel evolution

The similarities in codon and AA usage may be due to parallel evolution where species evolve similar traits (phenotypic or genotypic) independently because of their environmental similarities^52,53^. Parallel evolution of similar traits is considered as a common phenomenon among microorganisms^53^. For example, previous studies showed that the availability of resources (such as nutrients or water) and the type of co-habitant could potentially change the genetic make-up of microorganisms towards genetic parallelism^53^. Microorganisms from similar types of ecological niches may evolve under common physical and chemical constraints hence may experience comparable selection pressure irrespective of their physical distances. For instance, microbes in a desert soil sample are more likely to experience similar environmental (such as similar types of changes in temperature, pressure, humidity, or oxygen levels) and chemical constraints (such as pH, salinity, etc.) as of any other desert soil sample than any water sample or sample from any host-associated habitats. These types of common constraints may drive parallel evolution of codon or AA usage towards a common pattern independently (at least by part) among the population evolving under similar environmental conditions.

### Baas Becking hypothesis

The similarities in the codon and AA usage across habitats may also be explained in the light of the “Baas Becking hypothesis”. This hypothesis is famously quoted as “everything is everywhere but the environment selects” which is based on the proposition that microorganisms are present almost everywhere however environment can decide which microorganisms can thrive and proliferate in that environment^54^. According to this hypothesis, the spatial distribution of microorganisms that we observe in different habitats has been shaped mainly by ecological factors rather than by geographical distances. Recently, extending this hypothesis, it was proposed that the dispersal potential of genes is also shaped by environmental factors rather than by geographical barriers^54^. If this is true, it is likely that environmental similarities may favor the dispersion of similar types of gene pools with overall similarities in their codon and AA usage. This hypothesis is apparently similar to the parallel evolution hypothesis, however, as far as we understand the parallel evolution hypothesis focuses on the development of new traits under the constraints imposed by environmental factors. On the other hand, the Baas Becking hypothesis mainly considers the effect of the environment on the manifestation (dispersion potential) of traits (microorganisms) that are already present. In other words, Bass Becking hypothesis suggests environment acts as an assessor of whether a particular (existing) trait could exist or not.

Finally, we analyzed whether the samples derived from different habitats show any specific pattern in the extent of codon bias. Previously, numerous studies have been initiated to measure the degree of CUB in different microorganisms at the species level^1,2,8,9^. However, until now, no large-scale initiative has been undertaken to measure the extent of selection that may operate on the CUB of microorganisms in their natural habitats. Natural sequences evolve under various evolutionary constraints. Randomly generated sequences being free of such constraints were widely used as null models to access the strength of selection acting on different genomic and proteomic traits^55,56^. Therefore, here we compared the extent of CUB in each sample to what can be expected considering the random distribution of codons. To measure CUB, we considered three indices namely CAI, ENC, and DCBS values.CAI and ENC are the two most commonly used matrices of CUB^14^, while we particularly considered DCBS because this index can take into account mutational biases^41^. Our results suggested that samples from host-associated habitats such as the digestive system or oral environment show a higher extent of selection for codon bias than other samples. In contrast, soil and water samples were found to show the least extent of selection. Most of the host-associated microorganisms follow a biphasic lifestyle in which part of their life they live in the external environment and another part in the host organisms^57,58^. Moreover, in absence of a specific host, they may colonize in other related hosts. This provides host-associated microbes with an alternative habitat, where growth conditions are potentially different from environmental habitats. Overall these results support the earlier observation suggesting microbes that thrive in variable environments show a higher extent of CUB to adapt with the constraints enforced by different environments^2^.

In addition, to the results above we believe that the usage of CUB indices at the level of the entire community as suggested here is an approach that can be used in the future for characterizing and comparing communities and not only single genes or single organisms. The advantage of these indices is relevant also in the case of communities and includes their simplicity on one hand in addition to the important information they provide on the other hand^14^. Based on the observed results and above discussion our conclusions are following:

1. Microorganisms show community-specific trends in their codon and AA usage in their natural habitat.
2. These trends cannot be fully explained considering the potential effect of GC content or functional or taxonomic similarities.
3. There is evidence of selection on the codon usage bias in each sample irrespective of their environmental origin.
4. Consistent with earlier studies, our study implied that microorganisms that can live in a wide range of habitats such as some host-associated microorganisms show higher selection for codon usage bias compared to those that live in specialized habitats.
5. CUB indices can be generalized for the analysis of communities of organisms and not only single genes or organisms.

## Methods

### Data collection

Metagenomic project information was collected from the MGnify metagenomic database^31^. Currently (September 2021), microbiome data (sequence, taxonomic, and functional information, etc.) of 325,323 environmental samples can be found in this database. Often, microbes from similar ecological communities have been studied by different groups at different times and locations. Therefore, multiple projects were deposited for most of the microbial habitats. Here we considered the projects for which at least 5 samples are deposited (for reliable statistical tests) in this database and for any habitat type if we found multiple projects were deposited by the same group of investigators we considered one project. We start our analysis by considering 5 samples randomly from 90 metagenomic projects. The final classification of the selected samples was made considering sample information from various sources such as BioSamples database^59^, Sequence Read Archive (SRA) database^60^, and MGnify database^31^. Samples were classified broadly according to the ecological features of the sample material; if no such information is available from BioSamples^59^ or SRA database^60^, we considered the classification as suggested in MGnify database^31^. Details of the selected projects and associated sample information can be found at Zenodo (10.5281/zenodo.7455261).

### Processing of metagenomic reads, assembly, and prediction of coding sequences

Raw sequence read files of each selected sample were retrieved from the SRA database^60^. These reads are then processed with a rigorous quality check pipeline (Fig. 1). Given the run id, this pipeline automatically retrieves all read files associated with a sample from the SRA database^60^ using fastq-dump (https://trace.ncbi.nlm.nih.gov/Traces/sra/sra.cgi?view=toolkit_doc&f=fastq-dump), processes the reads with different quality check algorithms, assembles the high-quality reads, and provides CDS sequences in the final step. Depending on the read file type, this pipeline processes the samples either in the single-end or in paired-end mode; any unpaired reads in paired-end samples are processed in single-end mode and are included in the assembly step. The steps involved in this pipeline are briefly described here. Details of the commands used for each step can be found in our supplementary software file. At the first step, sequences containing bar code, adopter, or bad-quality nucleotides are trimmed as well as low-quality sequences (quality score below 10) and short sequences (length below 50 nucleotides) are removed using the BBDuk algorithm of BBTools. BBTools is a suite of bioinformatics tools designed for the analysis of DNA and RNA next-generation sequence data developed by the Joint Genome Institute (https://jgi.doe.gov/data-and-tools/bbtools/). Filtered reads were subjected to quality check again using the Trimmomatic sequence quality filtering tool^61^. All samples are checked for possible host sequence contamination by aligning the reads against reference genomes using the Bowtie 2 aligner^62^. As a general step, reads from all samples are aligned against the human reference genome. Additionally, samples from other hosts such as mouse, cow, pig, etc. are aligned again against the reference genome of the respective organism. Bowtie2 index files of the most recent versions (as of October 2020) of all the reference host genomes (build NCBI) were downloaded from the Bowtie 2 website^62^. The final quality of the processed reads (single-end or paired-end) was accessed through the FastQC algorithm (https://www.bioinformatics.babraham.ac.uk/projects/fastqc). At the next step, the samples that passed the FastQC quality check were assembled using MEGAHIT^63^ with the version optimized for metagenomic samples. MEGAHIT is a de Bruijn graph-based assembler that does not need any reference genome and was considered to be one of the best algorithms for assembling metagenomic reads^63^. MEGAHIT was run either in the single-end or paired-end mode (depending on the read file type) by adjusting the “presets” option as “sensitive” (slower but more rigorous search) or “large” (recommended for large metagenomes) option depending on the size of fastq read files. The minimum length of the contigs to be reported was chosen to be 60 nucleotides. Potential protein-coding regions were predicted from the contigs using the default settings of MetaProdigal^64^, an updated version of the well-known gene prediction tool Prodigal^65^ specifically designed for the metagenomic reads. MetaProdigal was shown to identify genes in short, anonymous coding sequences with a high degree of accuracy^65^. Sample for which FastQC reported problems in the read quality or less than 10,000 CDS were predicted in the gene prediction step was discarded and an alternative sample was chosen from the same project and processed in a similar way. This process was repeated until we got 5 samples for each project after processing. For a few projects (13 projects) we couldn’t find 5 samples that passed the FastQC quality check step and for which at least 10,000 CDSes were predicted. For these projects, we considered less than 5 samples. For comparison of results, predicted CDS sequences of each sample were also collected from the MGnify metagenomic database^31^ and analyzed separately. This database used a generic pipeline (details in reference^31^) to process the raw reads of each sample and predicted CDS/protein sequences from the processed reads along with their functional and taxonomical analysis. The database provides predicted protein sequences of each sample but does not provide the predicted CDS sequences directly. To get the CDS sequences, we predicted CDS from the filtered reads (retrieved from the database) of each sample using the same algorithms by which the database predicted protein-coding sequences and then matched the translated protein products of the CDSes with the protein sequences provided in the database. In this way, we could retrieve protein-coding gene sequences for 407 of 422 test samples.

### Calculation of codon/AA usage distances among metagenomic samples

To calculate similarity/dissimilarity among the metagenomic samples in terms of their codon usage we calculated the frequencies of 61 codons (excluding stop codons) considering all the genes in each sample. Codon frequencies were calculated following two approaches: (i) count of each codon in the sample was normalized by the total count of all the 61 codons in the sample (referred as absolute codon usage frequency or absCUFs), and (ii) count of each codon in the sample was normalized by the sum of all its synonymous codons in the sample (following Diament et al., we referred this as synonymous codon usage frequency or synCUFs^66^). The basic difference between these two approaches is that in the synCUFs approach normalization was done for each group of codons coding the same AA separately considering only the frequencies of synonymous codons rather than all other codons as in absCUFs. Being independent of AA usage and the abundance of synonymous codons, synCUFs can directly reflect synonymous codon usage bias. For each sample, AA frequencies were calculated by dividing the total count of each AA by the total length of all the proteins in the sample.

To estimate the similarity/dissimilarity in codon/AA usage among the selected samples we considered three well-known measures of distances (i) EU distance, (ii) ES distance^66^, and (iii) BC dissimilarity. The EU distance in codon usage between any two samples was calculated as follows

$$d_{pq} = \sqrt {\mathop {\sum}\limits_{i = 1}^{61} {\left( {q_i - p_i} \right)^2} }$$

Where *q*_*i*_ and *p*_*i*_ are codon frequency vectors (absCUFs or synCUFs) of the two samples, respectively.

ES distance method is based on Kullback–Leibler divergence for information gain, a measure widely used for comparing probability distributions in the field of information theory. Diament et al.^66^, first introduced this method to estimate codon usage distances between genes. Given the frequency vectors (absCUFs or synCUFs) of a pair of samples p and q, the ES distance between the samples was calculated as:

$$d_{_{KL\left( {p,q} \right)}} = \mathop {\sum}\limits_i^{61} {{{{\mathrm{log}}}}\frac{{p_i}}{{q_i}}p_i}$$

$${{{\mathrm{m}}}} = \frac{1}{2}\left( {{{{\mathrm{p}}}} + {{{\mathrm{q}}}}} \right)$$

$${{{\mathrm{d}}}}_{{{{\mathrm{ES}}}}}({{{\mathrm{p}}}},{{{\mathrm{q}}}}) = \sqrt {d_{KL}\left( {p,m} \right) + d_{KL}\left( {q,m} \right)}$$

Where *q*_*i*_ and *p*_*i*_ are codon frequency vectors (absCUFs or synCUFs) of the two samples respectively and d_KL_ is the Kullback–Leibler divergence.

BC dissimilarity is a well-known dissimilarity function, widely applied in ecological studies to compare species abundances^67^. BC dissimilarity in codon usage between any two samples was calculated as:

$${{{\mathrm{d}}}}_{{{{\mathrm{BC}}}}} = 1 - \frac{{2Cpg}}{{Sp + Sq}}$$

Where *Cpq* is sum of minimum values of codon frequencies for each codon between the two samples and S_p_ and S_q_ are the sum of codon frequencies for all codons in the two samples respectively. BC dissimilarity ranges from 0 to 1, where higher values suggest more dissimilar codon usage.

Distances in AA usage frequencies among the samples were calculated using the same distance methods (EU, ES and BC dissimilarity).

### Principal Component Analysis and ANOSIM test

Principal component analysis (PCA) helps visualization of complex multi-dimensional data into lower dimensions without losing much information^68^. We applied PCA to cluster samples based on their codon and AA usage and also on their functional and taxonomic abundances considering the respective frequency vectors. To access the statistical significance of the clustering, we performed ANOSIM test, a non-parametric test widely used in ecological studies to compare distances between two or more groups. As a measure of the extent of differences, ANOSIM provides “R” co-efficient (and associated *P*-value) which is the ratio of between groups distances to within groups distances. The R coefficient ranges from -1 to 1, where positive and higher R-values suggest greater separation between the test groups than within the test groups, while negative R-value suggests the reverse. *P*-value, in the test, is determined by permuting the grouping vectors considering empirical distributions of R under the null model. PCA was performed using the “prcomp” function and visualized using the “ggbiplot” library of statistical package R (version 3. 2. 1)^69^ and the ANOSIM test was executed using its vegan package considering the EU distance and BC dissimilarity method separately with 10,000 permutation values.

### Permutation test for comparing codon/ AA usage distances

For each group of samples under different environmental categories, we calculated an index value we called as Clustering Index (CI) which is the ratio of average distance in codon or AA usage among the samples from the same environmental group to that of samples from all other environmental groups. Here we explain this method by considering an environmental group “digestive system” as an example. We first calculated the average pairwise distances in codon/ AA usage considering all possible combinations of samples where both the samples belong to the “digestive system” group. Next, we calculated average pairwise distances considering all possible combinations of samples where one sample is from the “digestive system” group and the other sample is from any other environmental group. The index is the ratio of these two average distances. CI value < 1 suggests that samples from the same environmental origin are close in their codon/AA usage relative to that of samples from different environmental origins and vice-versa.

To test the significance level, for each environmental group, we generated 100 random datasets by randomly assigning the samples in different environmental groups keeping the number of samples in each group unchanged. CI values were calculated for the random datasets following the same approach as the original dataset. *P*-value was defined as the number of times the CI values calculated from the random datasets were lower than that calculated from the corresponding real dataset divided by the number of random datasets (100).

### Calculation of CUB indices

To estimate the extent of bias in different metagenomic samples, we considered three commonly used metrics of CUB namely CAI^39^, ENC^40^, and DCBS^41,42^. These indices try to capture differences in observed codon usage frequencies from uniform distribution (ENC, DCBS) or from distribution of reference set of highly expressed genes (CAI)^14^. However, they employ different scales to quantify the deviation. The scales of CAI and ENC values range from 0-1 and 20-61 respectively, while DCBS scores can be any positive value^14^. Presence of strong CUB reflected in higher CAI or DCBS values but lower ENC values. These indices were calculated for each sample separately considering all predicted CDS sequences for the sample as follows:

### Calculation of CAI values

CAI values of each gene (g) of each sample was calculated using the equation as described in^39^.

$${{{\mathrm{CAI}}}}_g = \left( {\mathop {\prod}\limits_{i = 1}^L {w_i} } \right)^{\!\!{1/L}}$$

Where “L” is the length of the gene in the number of codons. For a codon “i”, *w*_*i*_ represents its codon weightage which is calculated based on the observed frequency of that codon relative to the frequencies of all its synonymous codons from a reference set of highly expressed genes.

$$w_i = \frac{{f_i}}{{max\left( {f_i} \right)}}$$

Where *f*_*i*_ is the observed frequency of the codon and max(*f*_*i*_) is the maximum observed frequency among all its synonymous codons in the reference set.

The reference set of highly expressed genes was generated for each sample separately, measuring the transcript abundance of the predicted CDS sequences using Kallisto algorithm^70^. Kallisto is a sequence-alignment algorithm widely used for the quantification of RNA sequences in ribo-seq data analysis^70^. It aligns sequences against the reference contig or CDS sequences and calculates transcript abundance as the number of reads successfully mapped to the reference sequence normalized by their length and other parameters. We ran Kallisto using predicted CDS sequences of each sample as input and the corresponding contig files as reference sequences following default parameters. For the reference set of highly expressed genes, we considered top 5% CDS sequences of each sample sorted according to their abundance values. To get CAI values sample-wise, we calculated average CAI values of all genes in each sample.

### Calculation of ENC values

ENC value of each sample was calculated considering all the genes in the sample together following the equation described in the reference^40^.

$$N_c = 2 + \frac{9}{{F_2}} + \frac{1}{{F_5}} + \frac{5}{{F_4}} + \frac{3}{{F_6}}$$

Where F is called homozygosity index and is calculated for a group of AAs having the same number of codons. For instance, there are 9 AAs with 2 codons. F value is calculated for all these 9 AAs together and represented as F_2_. Homozygosity index F for a group of AA is calculated as follows

$${{{\mathrm{F}}}} = \frac{{{\sum} {n_{i{\sum} {\left( {p_i^2 - 1} \right)} }} }}{{{\sum} {n_i - 1} }}$$

For an AA with k number of synonymous codons, each with counts n_1_, n_2_,…, n_k_

$${{{\mathrm{N}}}} = \mathop {\sum}\limits_{i = 1}^k {n_i} \,{{{\mathrm{and}}}}\,p_i = n_i/{{{\mathrm{n}}}}{{{\mathrm{.}}}}$$

### Calculation of DCBS values

DCBS value of each gene (g) in each sample was calculated following^41,42^. Briefly, for each codon in a sample, we calculated a score *d*_*xyz*_ using the following equation

$$d_{xyz} = \frac{{f\left( {x,y,z} \right) - f_1\left( x \right){\cdot}f_2\left( y \right){\cdot}f_3\left( z \right)}}{{f_1\left( x \right){\cdot}f_2\left( y \right){\cdot}f_3\left( z \right)}}$$

Where *f*(*x,y,z*) are the frequencies of the codon xyz, and *f*_1_(*x*), *f*_2_(*y*), and *f*_3_(*z*) are the observed frequencies of nucleotides x, y, and z at the first, second, and the third codon position, respectively. These frequencies were calculated for each sample separately considering all the predicted CDS sequences in the sample. DCBS value of each gene (g) in a sample was calculated as geometric mean of *d*_*xyz*_ values over all its codons (except stop codons)

$${{{\mathrm{DCBS}}}} = \frac{{\mathop {\sum}\limits_{i = 1}^L {d_{xyz}} }}{L}$$

Where ‘L’ is the length of the gene in the number of codons. Finally, the DCBS value of each sample was estimated as the average DCBS values of all genes in the sample.

### Generation of random sequences and calculation of Z-scores

To test whether there is any kind of selection on the codon usage of the samples, we compared the codon usage bias of real sequences with that of their random sequences. To generate random sequences, coding sequences of a sample were randomly permuted 20 times using the SPARCS algorithm^71^. Thus, 20 sets of random sequences were generated from the real sequences of each sample. We specifically chose this algorithm to generate random sequences because this algorithm preserves the encoded protein sequence and the dinucleotide frequencies hence effects of other parameters such as mRNA secondary structure, and GC content, etc. are expected to be minimum^71^. Because of high computational cost, random sequences were generated for 417 samples (out of 422 samples in our dataset) excluding 5 samples with a very large number of predicted CDS sequences. We compared the codon usage scores (average CAI, DCBS, and ENC values) of real sequences of each test sample with that of their randomized version through the Z-score approach following previous studies^55,56^. For each sample, Z-score was calculated as:

$$Z_{score} = \frac{{Average\,codon\,usage\,score\left( {CAIorDCBSorENC} \right)ofreal\,sequences - Average\,codo\,nusage\,score\,of\,20\,randomizedgenomes}}{{standarddeviation\,of\,codonusagescore\,of\,the\,20\,randomizedgenomes}}$$

For the parameters like CAI and DCBS where higher scores indicate more bias, positive Z-scores denote that real sequences are more biased than their corresponding random sequences, while for ENC (lower value indicates higher bias) a negative Z-score denotes more bias in the real sequences.

### Taxonomic analysis of the metagenomic samples

For the taxonomic identification, we considered a well-known taxonomic abundance estimation tool for metagenomic samples namely Kraken^72^. Kraken is an ultrafast algorithm specifically designed to predict taxonomic classifications based on the exact alignment of k-mers^72^. Thus Kraken was shown to provide more accurate results (higher sensitivity and precision) than other commonly used taxonomic annotators at a much faster rate and using much lesser memory^72^. Here we estimated abundance values of different taxonomic ranks in our test samples by employing all the predicted CDS sequences in each sample against the standard Kraken databases. CDS sequences of each sample were annotated at six taxonomic ranks: (i) phylum, (ii) class, (iii) order, (iv) family, (v) genus, and (vi) species levels. However, for the calculation of abundance values, we considered annotation up to the genus level (species-level annotations were discarded for low confidence identification). Further, we also discarded annotation at the class level due to the relatively lower number of annotated taxons under this rank. To account for the variation in the sample sizes we calculated their relative abundance in each sample. Relative abundance values were calculated by normalizing their count in each sample by the number of sequences with identified taxonomy in the sample. Distances in taxonomic abundance among the samples were calculated for each taxonomic rank separately using the EU and BC methods and considering the frequencies of taxons that were detected at least once in the majority (300 out of 422) of the test samples.

### Functional analysis

For functional information, proteins predicted for each sample were subjected to InterProScan functional annotation tool (version-v83.0)^73^. InterProScan provides comprehensive information about protein families, domains, and functional sites by integrating signatures from several protein annotation servers including Pfam, CDD, PRINTS, PROSITE, SMART, ProDom, SUPERFAMILY, PANTHER, Gene3D, TIGRFAMs and HAMAP, etc.^73^. Functional annotations retrieved from these databases are highly redundant. Considering a large number of protein sequences in most of the samples, InterProScan was run against a subset of these databases namely Pfam, TIGRFAM, PROSITE, CDD, and GENE3D. Similar to taxonomic abundance, here we calculated the relative abundance of three GO categories: BP, CC, and MF in each sample. The relative abundance of each GO term in a sample was calculated by dividing the total count of the term in the sample by the total number of proteins in the sample for which GO annotations can be found. Altogether, 3702 different GO terms were found in all the test samples, most of which appeared only in few samples. Therefore, to calculate distances in GO term frequencies among the samples we considered abundance matrices of 500 GO BP, 500 GO MF and 100 GO CC terms respectively sorted according to the number of samples in which they were found. All of these terms were found at least once in more than 50% of test samples. Distances in GO term frequencies among the samples were calculated for each GO category (BP, MF, and CC) separately following the EU and BC dissimilarity methods.

Clusters of Orthologous Groups (COGs) are another important functional classification scheme for prokaryotic sequences^74^. The most recent update of the COG database includes 4877 different COGs definitions, which are categorized into 23 broad functional categories^74^. For our analysis, we retrieved the COG definition of the predicted protein sequences of each test sample by utilizing the standalone RPS-BLAST algorithm from NCBI CD-Search utilities^75^ against the pre-compiled Conserved Domain Database (CDD) database using *P*-value cutoff 10^−2^ and default settings for all other parameters. The results of the RPS-BLAST search were processed through the rpsbproc algorithm. RPS-BLAST is a variant of the PSI-BLAST algorithm that provides domain-level annotations imported from several external sources, including COG annotations^75^. To group sequences into the COG functional categories, we discarded the sequences that were annotated with multiple COGs from different functional categories and considered sequences annotated with COGs specific to that category only.

### Analysis of codon/amino acid usage patterns of microorganisms collected from the fusionDB database

To study the codon and amino acid usage pattern of microorganisms at the species level we collected habitat information of 1,374 microorganisms from the fusionDB database^28^. The fusionDB database provides metadata such as their habitat information, preferred temperature, oxygen requirements, etc., for 1,374 taxonomically distinct bacteria. Protein coding CDS sequences and amino acid sequences of these microorganisms were retrieved from the NCBI GenBank database^76^ following their taxonomic identifiers and species names. Thus we could find CDS and amino acid sequences for 1270 out of 1374 bacteria in this database. For the environmental classifications of these bacteria, we broadly followed the habitat information as provided in this database. Briefly, when specific information regarding the habitat preference of a bacterium was available we consider the bacterium into that specific environmental category otherwise we broadly followed the habitat classification as mentioned in the fusionDB database. For reliable statistical tests, in this analysis, we mainly considered codon/amino acid usage frequencies of 925 bacteria from 7 environmental biomes namely: “Nasopharyngeal microflora” (24 bacteria), “Multi” (198 bacteria), “Fresh water” (110 bacteria), “Soil” (107 bacteria), “Intestinal microflora” (66 bacteria), “Marine” (85 bacteria), and “nonspecific host” (335 bacteria). There are at least 20 bacteria under each of these 7 biomes. Codon usage frequencies were calculated following absCUFs and synCUFs methods as described in our main text for the metagenomic dataset. Distances in codon/amino acid usage frequencies were calculated and compared within and between the groups following similar approaches as described in our main text for the metagenomic dataset.

### Identification of ribosomal protein-coding genes in test samples

To identify potential ribosomal protein-coding genes we considered three approaches: (i) from their COG annotations: any sequence belonging to COGs defined as “ribosomal proteins” (Supplementary Table 20) were considered as potential ribosomal protein-coding genes, (ii) from InterPro^73^ annotation: for this, we considered a list 478 different InterPro identifiers (Supplementary Table 20) that were defined as ribosomal proteins. Any proteins with these annotations were considered as probable ribosomal proteins (iii) through blast: we collected known ribosomal protein sequences of 13 prokaryotic organisms from “Ribosomal protein Dataverse” (https://dataverse.harvard.edu/dataverse/Ribosomal_protein_database;jsessionid=e498052c86fa6e8eff1fc0d0ef23) and also known ribosomal protein sequences from Ribosomal Protein Gene Database^77^. Sequences showing significant (*P* < 10^−4^) blast hit with any of these sequences through the bi-directional blast approach (interchanging query and target) were considered as potential ribosomal protein-coding sequences. Finally, in each sample, we considered a non-redundant set of sequences that are identified as ribosomal proteins by any one of the three approaches.

### Estimation of k-mer frequencies

k-mer frequencies in the CDS sequences of the test samples were estimated using the Jellyfish algorithm^78^. Jellyfish provides very fast and accurate k-mer counting of DNA sequences using an order of magnitude less memory as compared to other algorithms^78^. Estimation of k-mer frequencies for higher k is very computationally intensive^47^, specifically for large datasets like metagenomic samples. Therefore, here we considered mainly short k-mers for lengths of k ranging from k = 2 to k = 10 except k = 3 (which are equivalent to codons). In each sample, we estimated the frequencies of all possible k-mers for a given k by normalizing their count by the total occurrence of all the possible k-mers for that k in the sample. Divergence in the k-mer frequencies among the samples (within or between the environmental groups) was estimated using the distance matrices as described in the main text. For shorter k (k < = 5), we considered the frequencies of all possible k-mers in the calculation of distance matrices. However, for longer k (k > = 6), where the number of possible k-mers is very high, we first selected 2000 highly abundant (based on their frequencies in the test samples) k-mers for each k (k = 6,..,10). Next, we calculated distance matrices based on the frequencies of these selected k-mers in each sample.

### Statistical analysis and visualizations

All statistical tests were performed using R (The R Project for Statistical Computing)^69^. For correlation analysis, we calculated the non-parametric Spearman’s Rank correlation coefficient ρ, where significant correlations were denoted by *P*-values. To compare the distribution of test variables (such as codon usage distances or CUB indices) among different groups of samples we considered the Mann-Whitney U test or the Kruskal-Wallis H test (an extended version of Mann-Whitney U tests) depending upon the number of groups to compare. *P* values were adjusted for multiple comparisons using the Bonferroni post hoc test when samples from more than two environmental groups were considered for the test. For regression analysis, we considered the multiple linear regression model where the dependent and explanatory variables are assumed to be related linearly. Plots are generated using the ‘ggplot2” library of R.

### Reporting summary

Further information on research design is available in the Nature Research Reporting Summary linked to this article.

## Supplementary information

Supplementary information

Reporting Summary

Supplementary software

## Data Availability

Quantitative data underpinning the results reported in this article are deposited in the online scientific data repository Zenodo (10.5281/zenodo.7455261) and all supplementary tables and figures can be found in supplementary information supplied with this manuscript. All data related to this paper can also be requested from the corresponding author.

## Code availability

All the codes used to generate results that are reported in this paper and central to its main claims are provided in supplementary software file associated with this manuscript.
