# Supplementary material for: Determinants of associations between codon and amino acid usage patterns of microbial communities and the environment inferred based on a cross-biome metagenomic analysis
Source: NPJ Biofilms Microbiomes. 2023 Jan 24;9:5. doi: 10.1038/s41522-023-00372-w (PMC9873608; doi:10.1038/s41522-023-00372-w)
Supplement: Supplementary file 1 — Supplementary information [file 41522_2023_372_MOESM1_ESM.pdf]

Supplementary information:

Determinants of associations between codon and amino acid usage patterns of microbial communities and the environment inferred based on a cross-biome metagenomic analysis

Arup Panda[1], Tamir Tuller[1]*

[1]Department of Biomedical Engineering, Tel Aviv University, Tel Aviv 69978, Israel

* Corresponding author

e-mail: tamirtul@post.tau.ac.il (Tamir Tuller)

This PDF file includes:

Supplementary figures 1-19

Supplementary tables 1-20

Supplementary references

Supplementary figures:

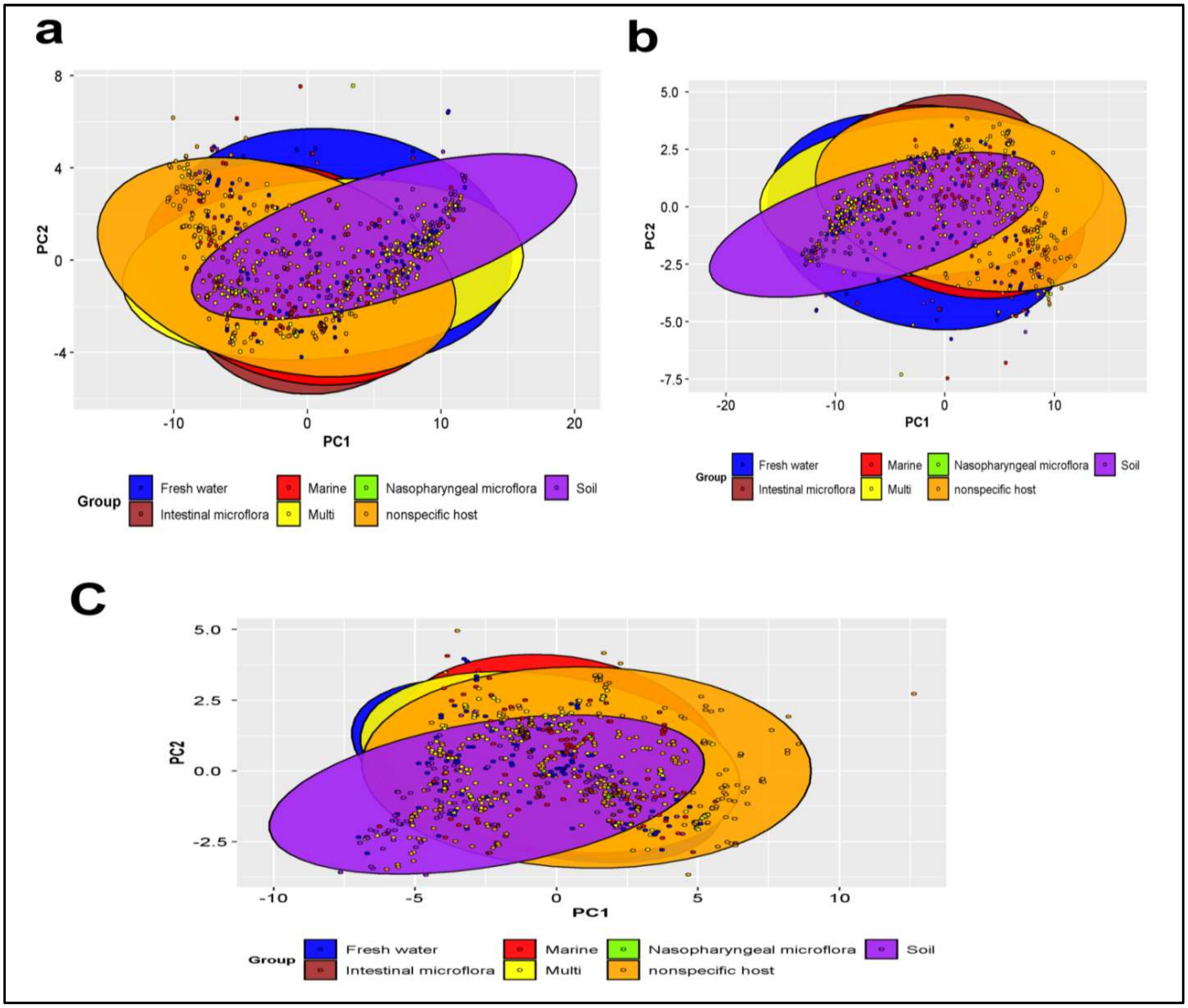

Supplementary Figure 1. Principal component analysis (PCA) on codon/amino acid usage frequencies of microorganisms collected from the fusionDB database. PCA analysis was applied on **a.** absCUFs, **b.** synCUFs, and **c.** amino acid usage frequencies of 925 microorganism collected from the fusionDB database. Each point in this figure represents one sample. Samples are grouped broadly following the habitat information collected from the fusionDB database (see main text). The color scheme is shown in the legend. The first two components of the PCA are presented here.

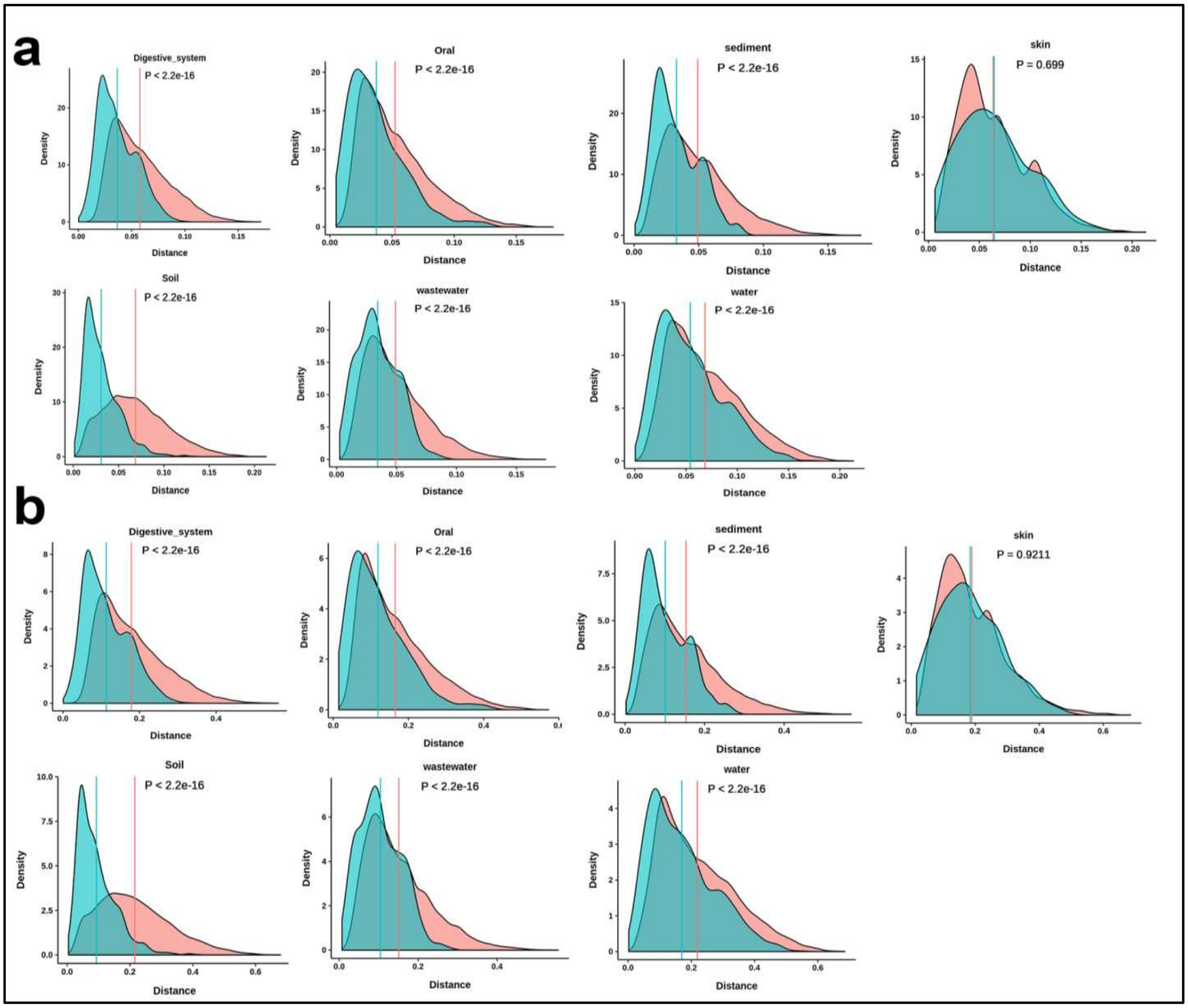

Supplementary Figure 2. Density plots showing absCUFs codon usage distances among the samples. This figure shows the density plots of absCUFs codon usage distances among the test metagenomic samples from 7 environmental biomes. **a.** Distances in the codon frequencies were calculated by Euclidean distance method. **b.** Distances in codon frequencies were calculated as Bray-Curtis dissimilarity method. For each sample in each selected habitat, we calculated all possible pair-wise codon usage distances (by these two methods separately) with all other samples either from the same (within-group comparison) or different habitats (between groups comparison). Next, we compared the within-group distances of codon usage with that of between-group distances habitat-wise. Statistical significance

of the differences for pair-wise comparison of within to between-group distances was accessed by Mann-Whitney U test and the corresponding *P*-values were shown in the respective panel. In each panel, vertical lines represent the average of within and between-groups distances in codon usage frequencies, respectively. Blue lines stand for within-group distances and pink for between-group distances, respectively.

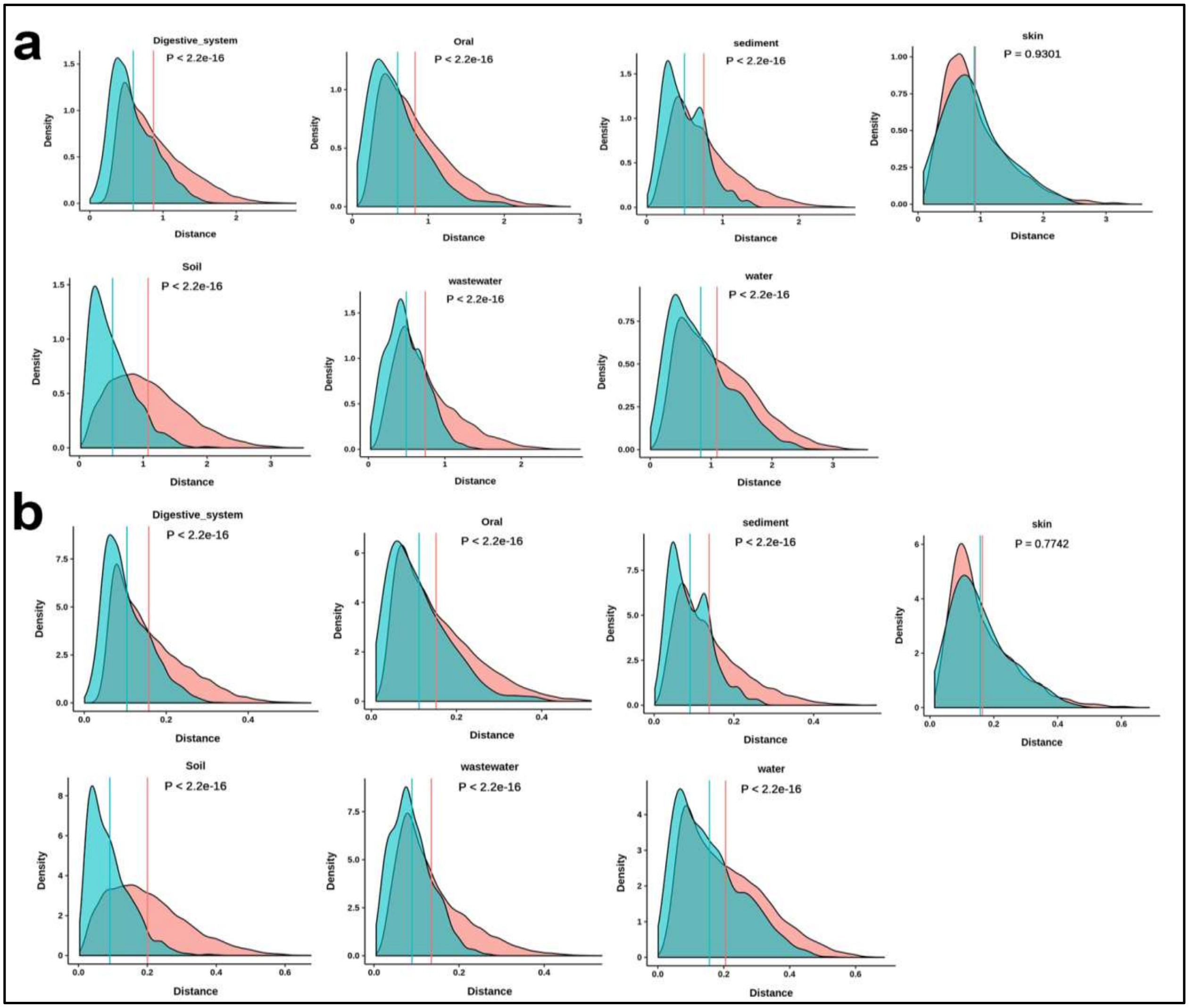

Supplementary Figure 3. Density plots showing synCUFs codon usage distances among the samples.

This figure shows the density plots of synCUFs codon usage distances among the test metagenomic

samples from 7 environmental biomes. **a.** Distances in the codon frequencies were calculated by Euclidean distance method. **b.** Distances in codon frequencies were calculated as Bray-Curtis dissimilarity method. For each sample in each selected habitat, we calculated all possible pair-wise codon usage distances (by these two methods separately) with all other samples either from the same (within-group comparison) or different habitats (between groups comparison). Next, we compared the within-group distances of codon usage with that of between-group distances habitat-wise. Statistical significance of the differences for pair-wise comparison of within to between-group distances was accessed by Mann-Whitney U test and the corresponding *P*-values were shown in the respective panel. In each panel, vertical lines represent the average of within and between-groups distances in codon usage frequencies, respectively. Blue lines stand for within-group distances and pink for between-group distances, respectively.

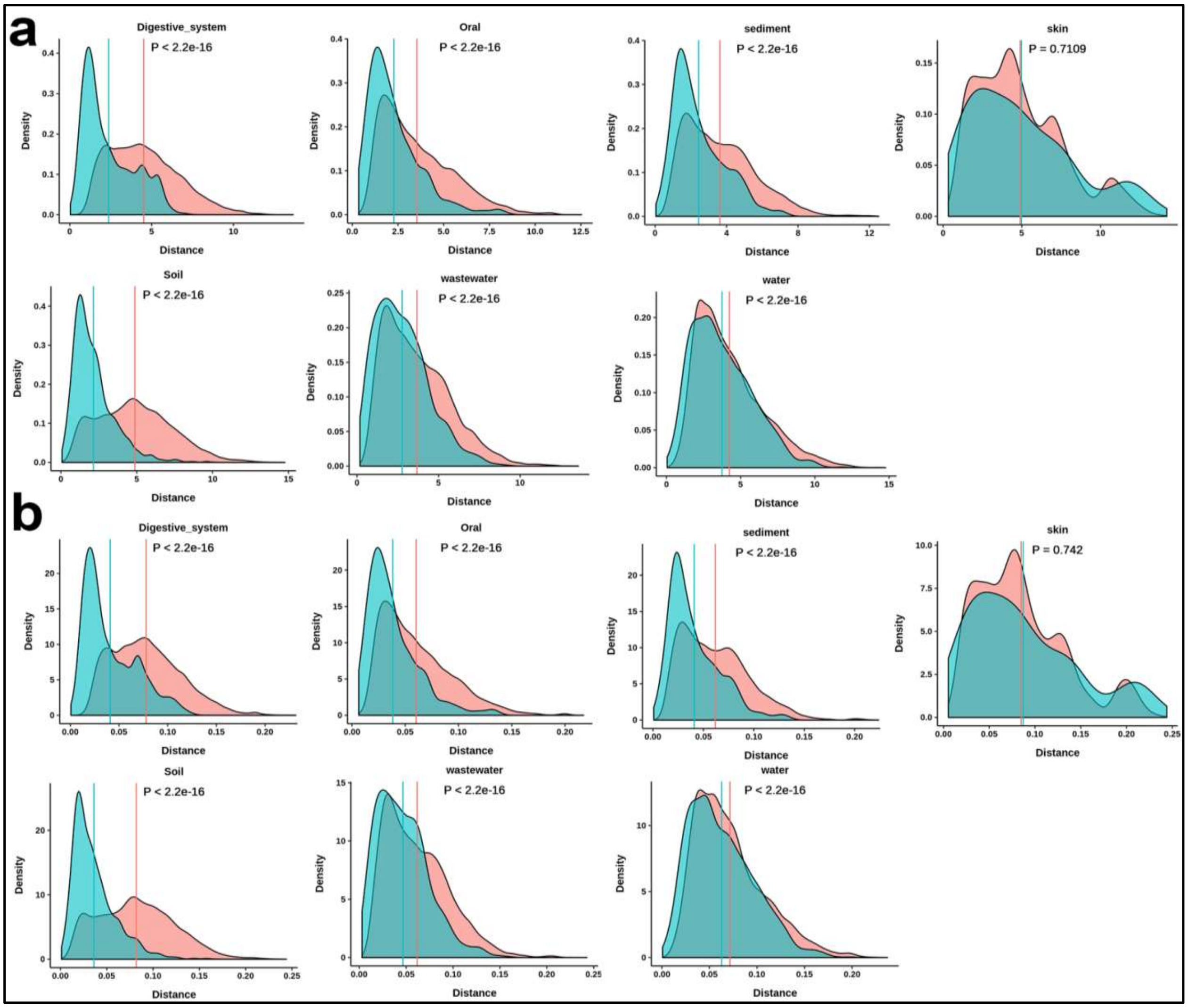

Supplementary Figure 4. Density plots showing amino acid usage distances among the samples from seven test biomes. This figure shows the density plots of amino acid usage distances among samples from seven ecological niches. Panel **a.** amino acid usage distances were calculated by the Euclidean method. Panel **b.** amino acid distances were calculated by Bray-Curtis dissimilarity method. For each sample in each selected habitat, we calculated all possible pair-wise amino acid usage distances with all other samples either from the same (within-group comparison) or different habitats (between-groups comparison). Next, we compared the within-group distances of amino acid usage with that of between-group distances habitat-wise. Statistical significance of the differences for pair-wise comparison

of within to between-group distances was accessed by Mann-Whitney U test and the corresponding *P*-values were shown in the respective panel. In each panel, vertical lines represent the average of within and between-group distances in codon usage frequencies, respectively. Blue line stands for within-group distances and pink for between-group distances, respectively.

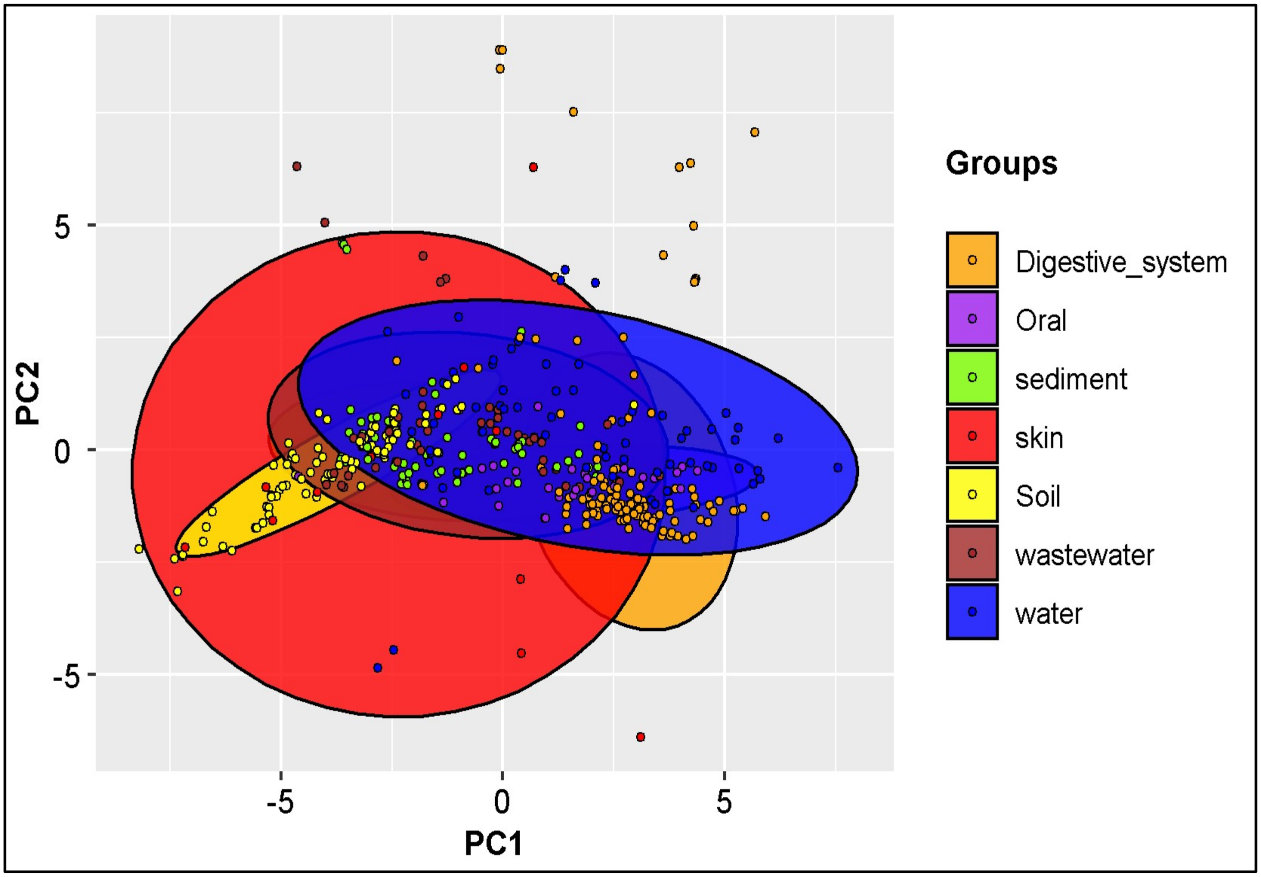

Supplementary Figure 5. Principal component analysis (PCA) based on the amino acid usage frequencies of samples from seven environmental biomes. Each point in the figure represents one sample. Samples are colored according to the environmental feature from where those were collected. The color scheme is shown in the legend. The first two components of the PCA are presented here. The first

two components (PC1 and PC2) can explain more than 70% of the variance in amino acid frequencies in the dataset**.**

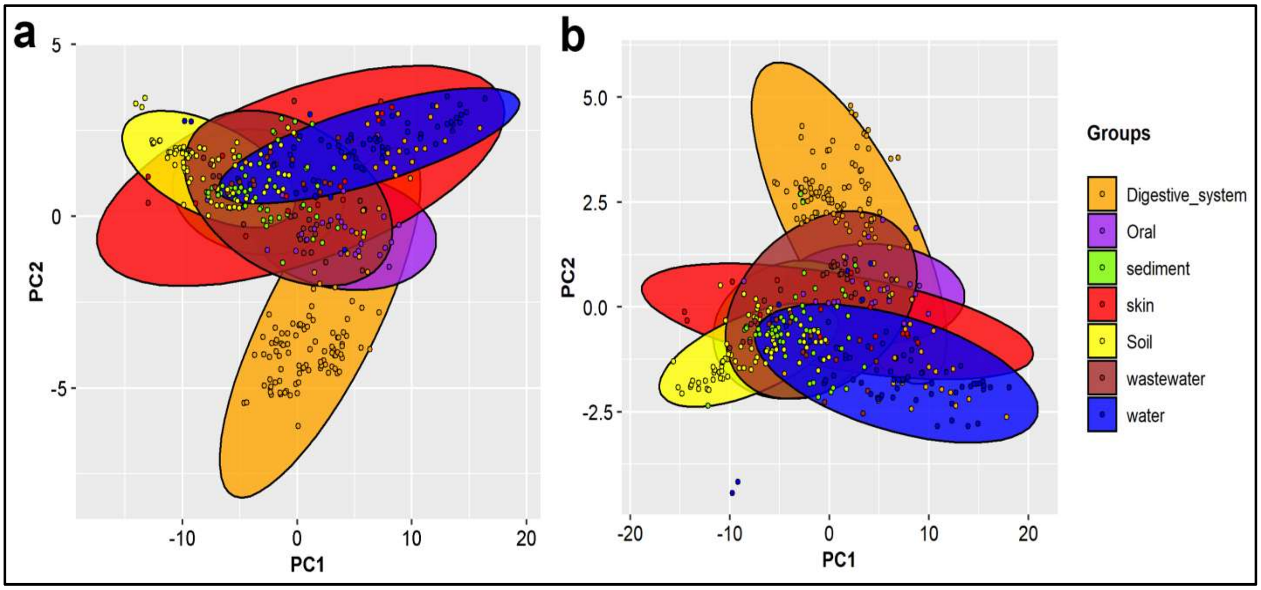

Supplementary Figure 6. Principal component analysis (PCA) on the codon usage frequency of samples from seven selected niches. CDS **s**equences of the samples were collected from MGnify metagenomic database. This figure shows the results of PCA analysis applied on the absCUFs frequencies (panel **a**) and synCUFs (panel **b**) frequencies of samples from seven selected biomes. Each point in the figure represents one sample. Samples are colored according to the environmental feature from where those were collected. The color scheme is shown in the legend. The first two components of the PCA are presented here. The first two components (PC1 and PC2) can explain more than 70% of the variance of codon frequencies in the dataset.

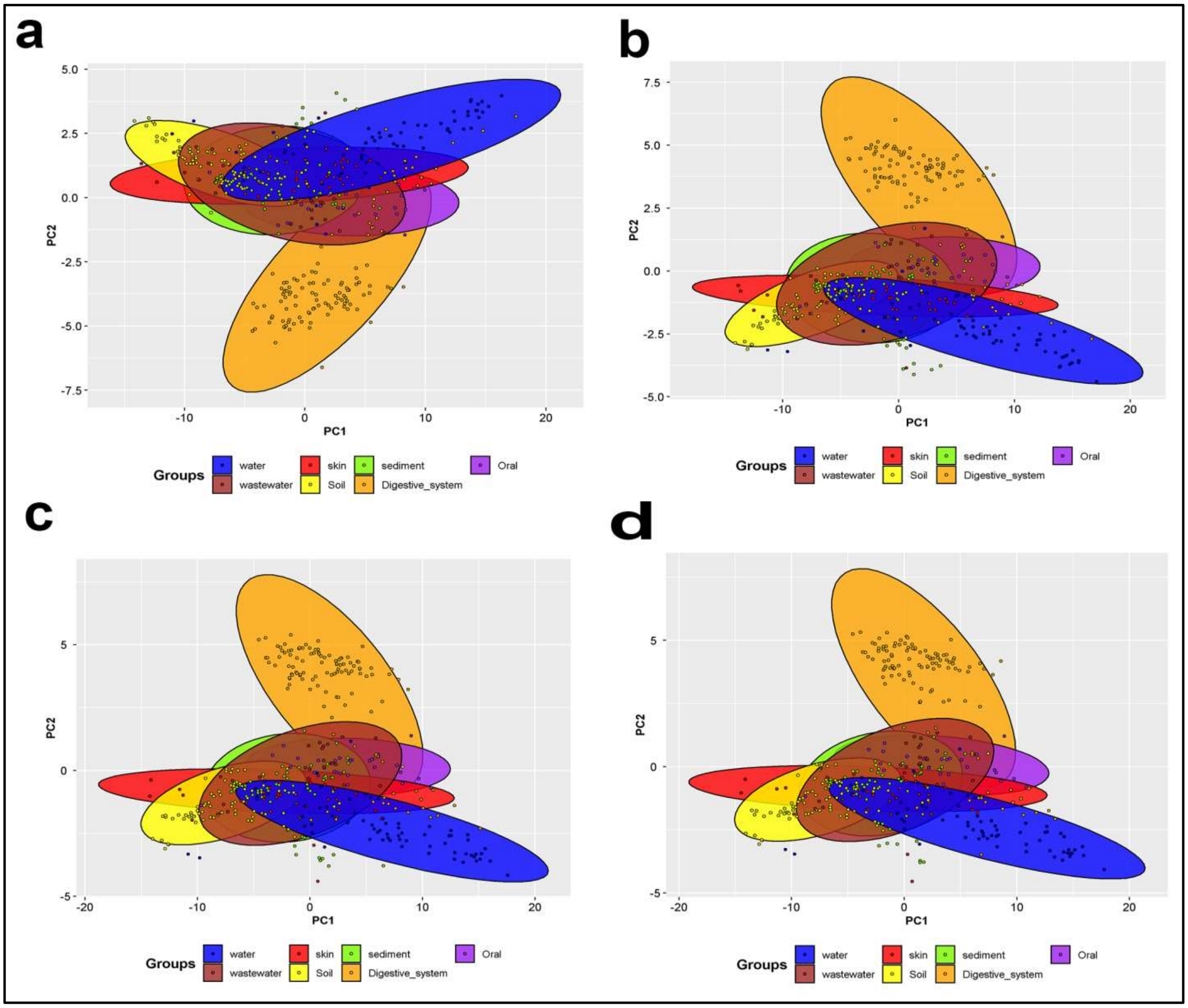

Supplementary Figure 7. Principal component analysis (PCA) on the absCUFs frequencies of CDSes predicted from subsampled reads. This figure shows the results of PCA analysis applied on the absCUFs frequencies of the test samples from seven environmental biomes. CDS sequences were predicted considering **(a)** 0.1%, **(b)** 1%, **(c)** 5%, and **(d)** 10% of all reads randomly in each sample following similar steps as described for CDS prediction from all reads without any subsampling. Each point in the figure represents one sample. Samples are colored according to the environmental feature from where those were collected. The color scheme is shown in the legend. The first two components of the PCA are presented here.

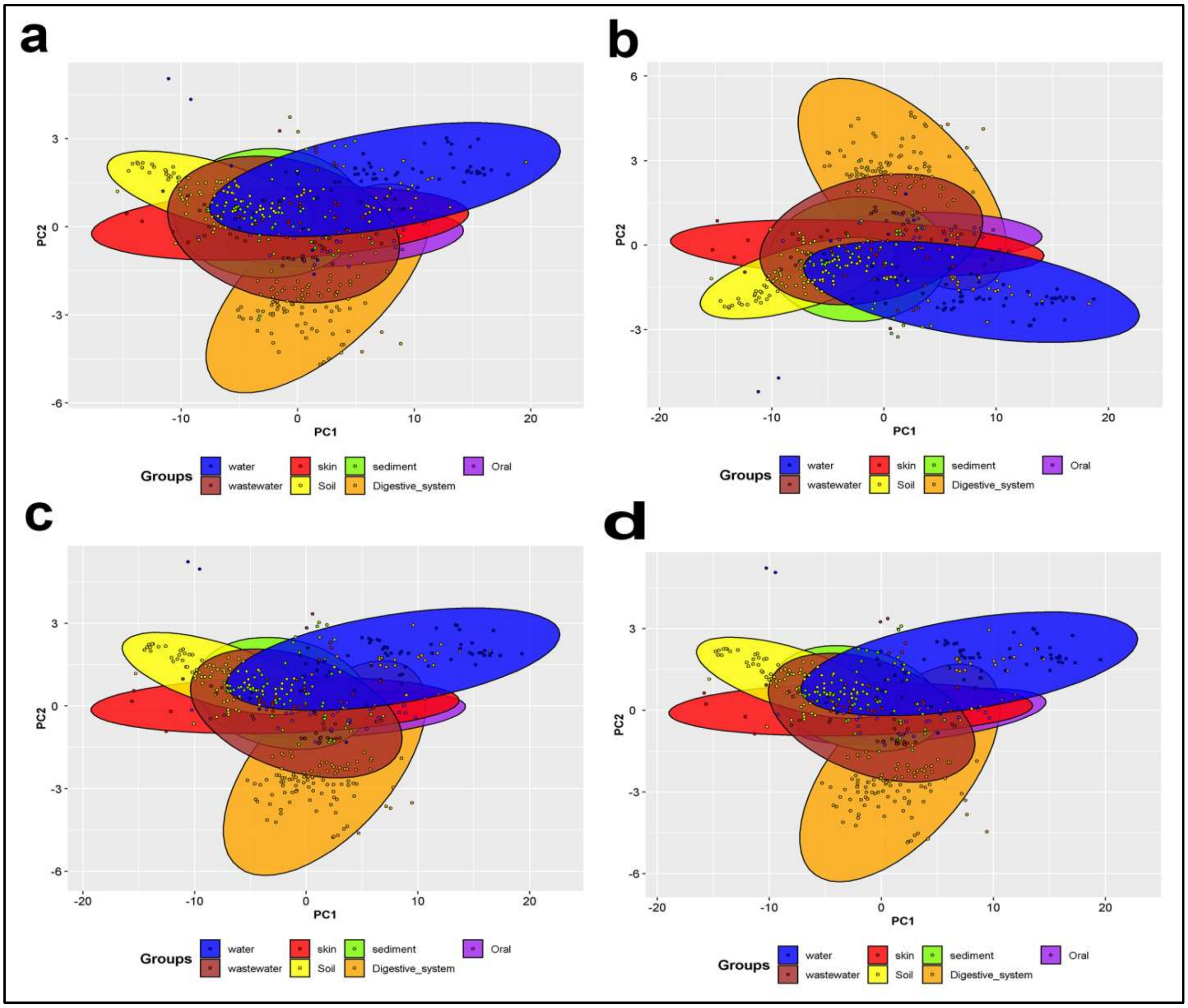

Supplementary Figure 8. Principal component analysis (PCA) on the synCUFs frequencies of CDSes predicted from subsampled reads. This figure shows the results of PCA analysis applied on the synCUFs frequencies of the test samples from seven environmental biomes. CDS sequences were predicted considering **(a)** 0.1%, **(b)** 1%, **(c)** 5%, and **(d)** 10% of all reads randomly in each sample following similar steps as described for CDS prediction from all reads without any subsampling. Each point in the figure represents one sample. Samples are colored according to the environmental feature from where those were collected. The color scheme is shown in the legend. The first two components of the PCA are presented here.

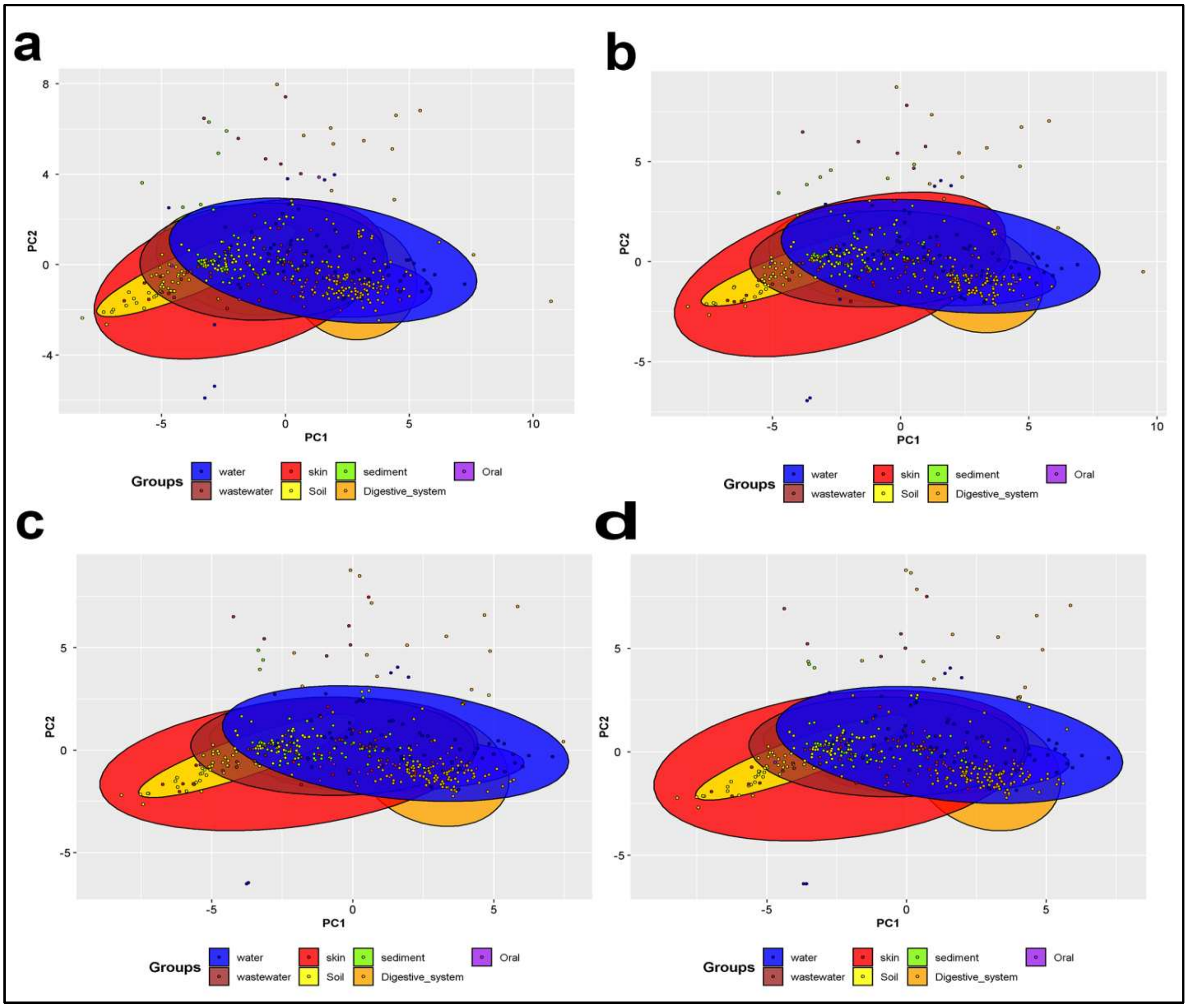

Supplementary Figure 9. Principal component analysis (PCA) on the amino acid usage frequencies predicted from subsampled reads. This figure shows the results of PCA analysis applied on the amino acid usage frequencies of the test samples from seven environmental biomes. Protein sequences were predicted considering **(a)** 0.1%, **(b)** 1%, **(c)** 5%, and **(d)** 10% of all reads randomly in each sample following similar steps as described for CDS prediction from all reads without any subsampling. Each point in the figure represents one sample. Samples are colored according to the environmental feature from where those were collected. The color scheme is shown in the legend. The first two components of the PCA are presented here.

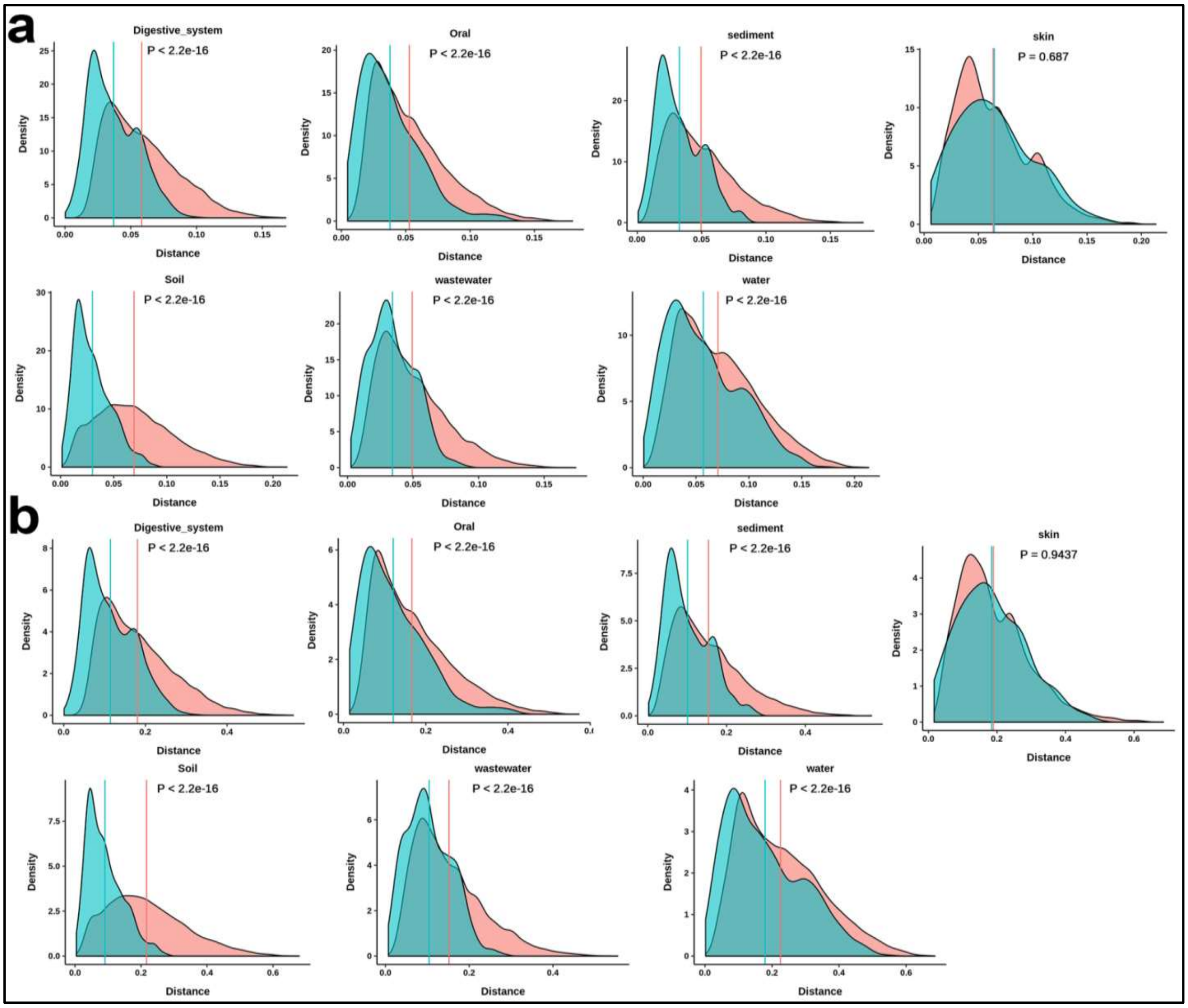

Supplementary Figure 10. Density plots showing absCUFs codon usage distances among the samples sequenced by the Illumina method. This figure shows the density plots of absCUFs codon usage distances among the test metagenomic samples sequenced by the Illumina method. **a.** Distances in the codon frequencies were calculated by Euclidean distance method. **b.** Distances in codon frequencies were calculated as Bray-Curtis dissimilarity method. For each sample in each selected habitat, we calculated all possible pair-wise codon usage distances (by these two methods separately) with all other samples either from the same (within-group comparison) or different habitats (between groups comparison). Next, we compared the within-group distances of codon usage with that of between-group

distances habitat-wise. Statistical significance of the differences for pair-wise comparison of within to between-group distances was accessed by Mann-Whitney U test and the corresponding *P*-values were shown in the respective panel. In each panel, vertical lines represent the average of within and between-groups distances in codon usage frequencies, respectively. Blue lines stand for within-group distances and pink for between-group distances, respectively.

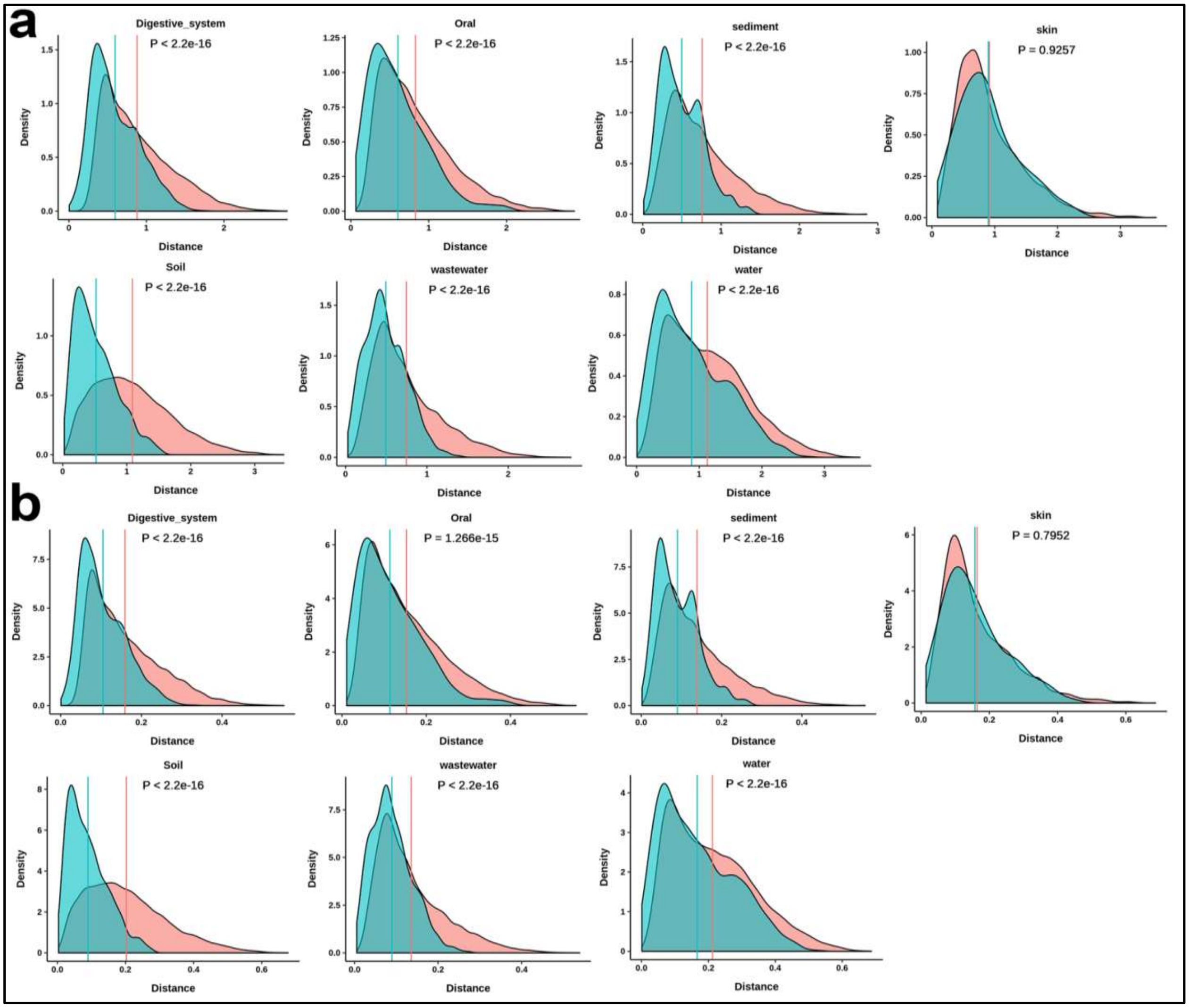

Supplementary Figure 11. Density plots showing synCUFs usage distances among the samples sequenced by the Illumina method. This figure shows the density plots of synCUFs codon usage distances among the test metagenomic samples sequenced by the Illumina method. **a.** Distances in the

codon frequencies were calculated by Euclidean distance method. **b.** Distances in codon frequencies were calculated as Bray-Curtis dissimilarity method. For each sample in each selected habitat, we calculated all possible pair-wise codon usage distances (by these two methods separately) with all other samples either from the same (within-group comparison) or different habitats (between groups comparison). Next, we compared the within-group distances of codon usage with that of between-group distances habitat-wise. Statistical significance of the differences for pair-wise comparison of within to between-group distances was accessed by Mann-Whitney U test and the corresponding *P*-values were shown in the respective panel. In each panel, vertical lines represent the average of within and between-groups distances in codon usage frequencies, respectively. Blue lines stand for within-group distances and pink for between-group distances, respectively.

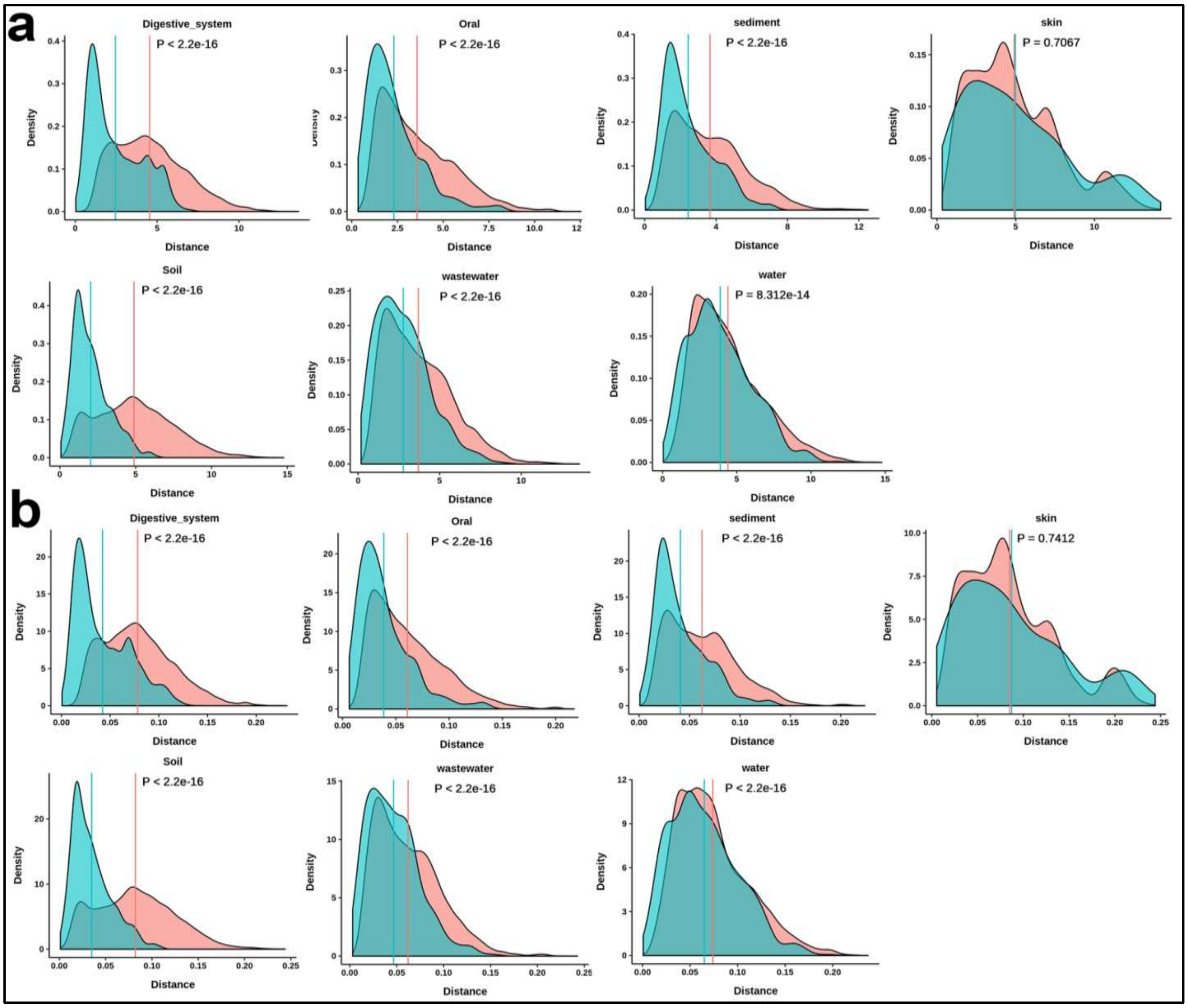

Supplementary Figure 12. Density plots showing amino acid usage distances among the samples sequenced by the Illumina method. This figure shows the density plots of amino acid usage distances among the test metagenomic samples sequenced by the Illumina method. **a.** Distances in the codon frequencies were calculated by Euclidean distance method. **b.** Distances in codon frequencies were calculated as Bray-Curtis dissimilarity method. For each sample in each selected habitat, we calculated all possible pair-wise amino acid usage distances (by these two methods separately) with all other samples either from the same (within-group comparison) or different habitats (between groups comparison). Next, we compared the within-group distances of codon usage with that of between-group distances habitat-wise. Statistical significance of the differences for pair-wise comparison of within to between-group

distances was accessed by Mann-Whitney U test and the corresponding *P*-values were shown in the respective panel. In each panel, vertical lines represent the average of within and between-groups distances in codon usage frequencies, respectively. Blue lines stand for within-group distances and pink for between-group distances, respectively.

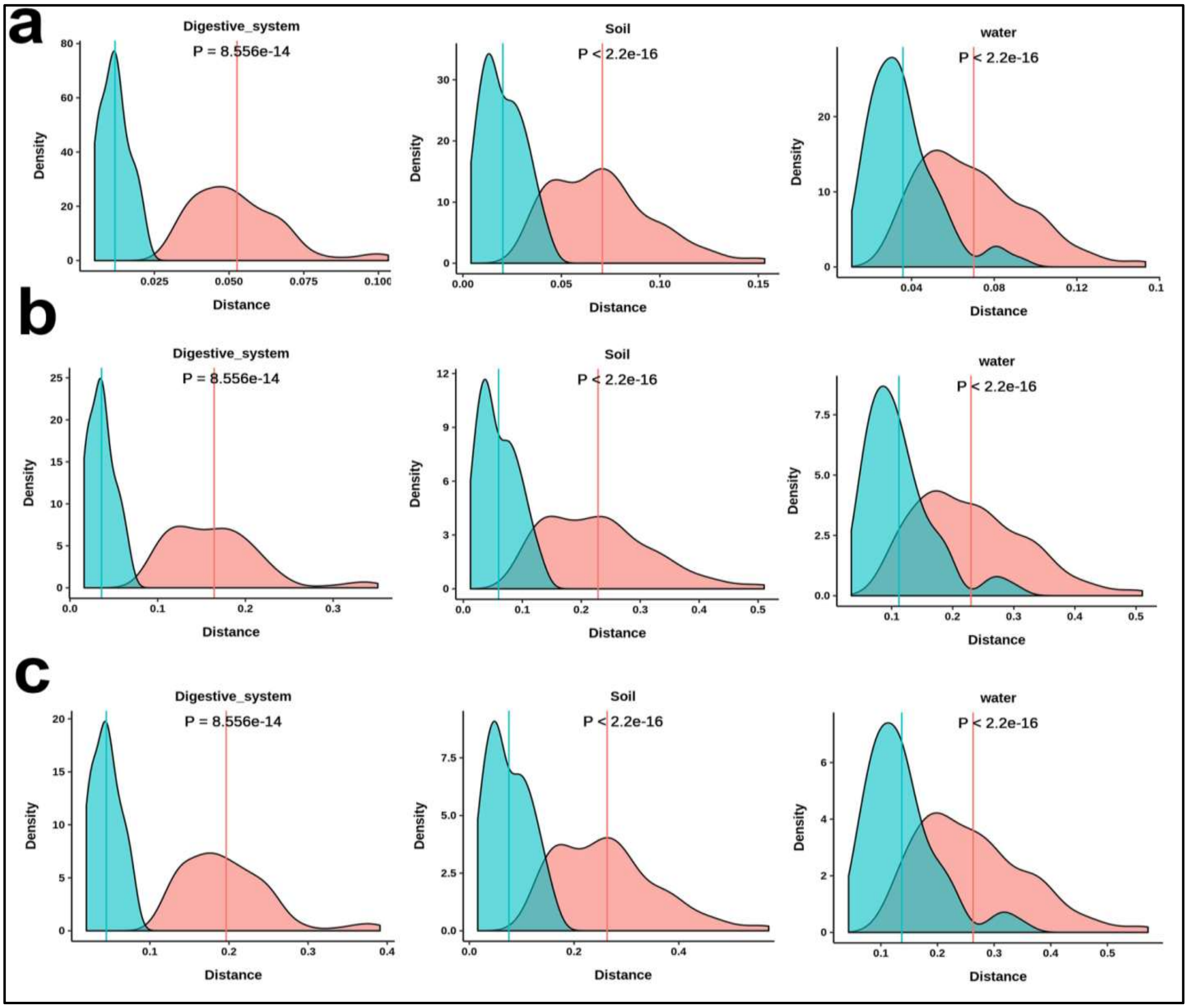

Supplementary Figure 13. Density plots showing absCUFs codon usage distances among the samples sequenced by the non-Illumina method. This figure shows the density plots of absCUFs codon usage distances among the test metagenomic samples sequenced by non-Illumina methods. **a.** Distances

in the codon frequencies were calculated by Euclidean distance method. **b.** Distances in codon frequencies were calculated as Bray-Curtis dissimilarity method, and **c**. Distances calculated by Endres–Schindelin distance method. For each sample in each selected habitat, we calculated all possible pair-wise codon usage distances (by these two methods separately) with all other samples either from the same (within-group comparison) or different habitats (between groups comparison). Next, we compared the within-group distances of codon usage with that of between-group distances habitat-wise. Statistical significance of the differences for pair-wise comparison of within to between-group distances was accessed by Mann-Whitney U test and the corresponding *P*-values were shown in the respective panel. In each panel, vertical lines represent the average of within and between-groups distances in codon usage frequencies, respectively. Blue lines stand for within-group distances and pink for between-group distances, respectively.

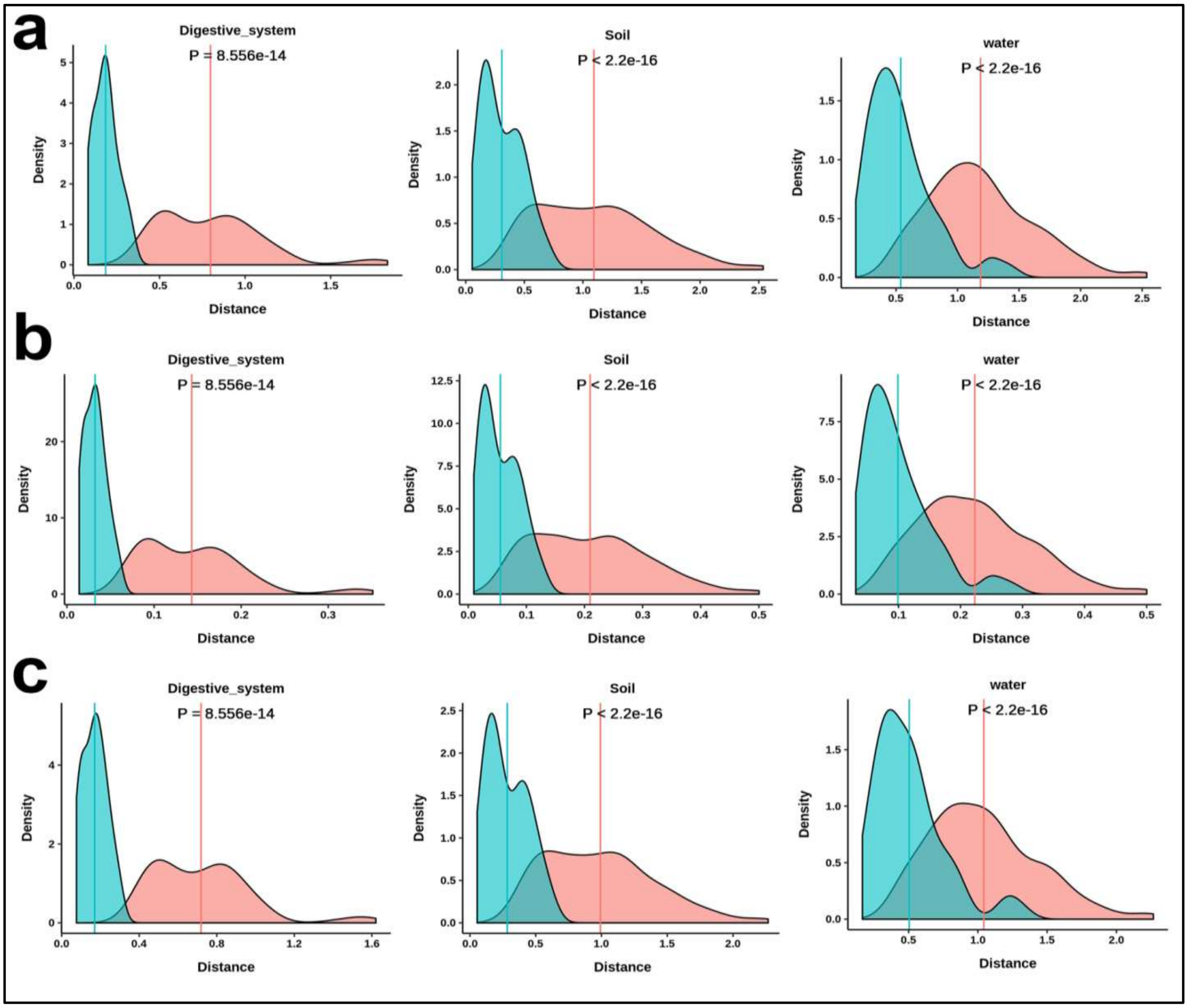

Supplementary Figure 14. Density plots showing synCUFs codon usage distances among the samples sequenced by the non-Illumina method. This figure shows the density plots of synCUFs codon usage distances among the test metagenomic samples sequenced by non-Illumina methods. **a.** Distances in the codon frequencies were calculated by Euclidean distance method. **b.** Distances in codon frequencies were calculated as Bray-Curtis dissimilarity method. **c**. Distances calculated by Endres–Schindelin distance method. For each sample in each selected habitat, we calculated all possible pair-wise codon usage distances (by these two methods separately) with all other samples either from the same (within-group comparison) or different habitats (between groups comparison). Next, we compared the within-group distances of codon usage with that of between-group distances habitat-wise. Statistical

significance of the differences for pair-wise comparison of within to between-group distances was accessed by Mann-Whitney U test and the corresponding P-values were shown in the respective panel. In each panel, vertical lines represent the average of within and between-groups distances in codon usage frequencies, respectively. Blue lines stand for within-group distances and pink for between-group distances, respectively.

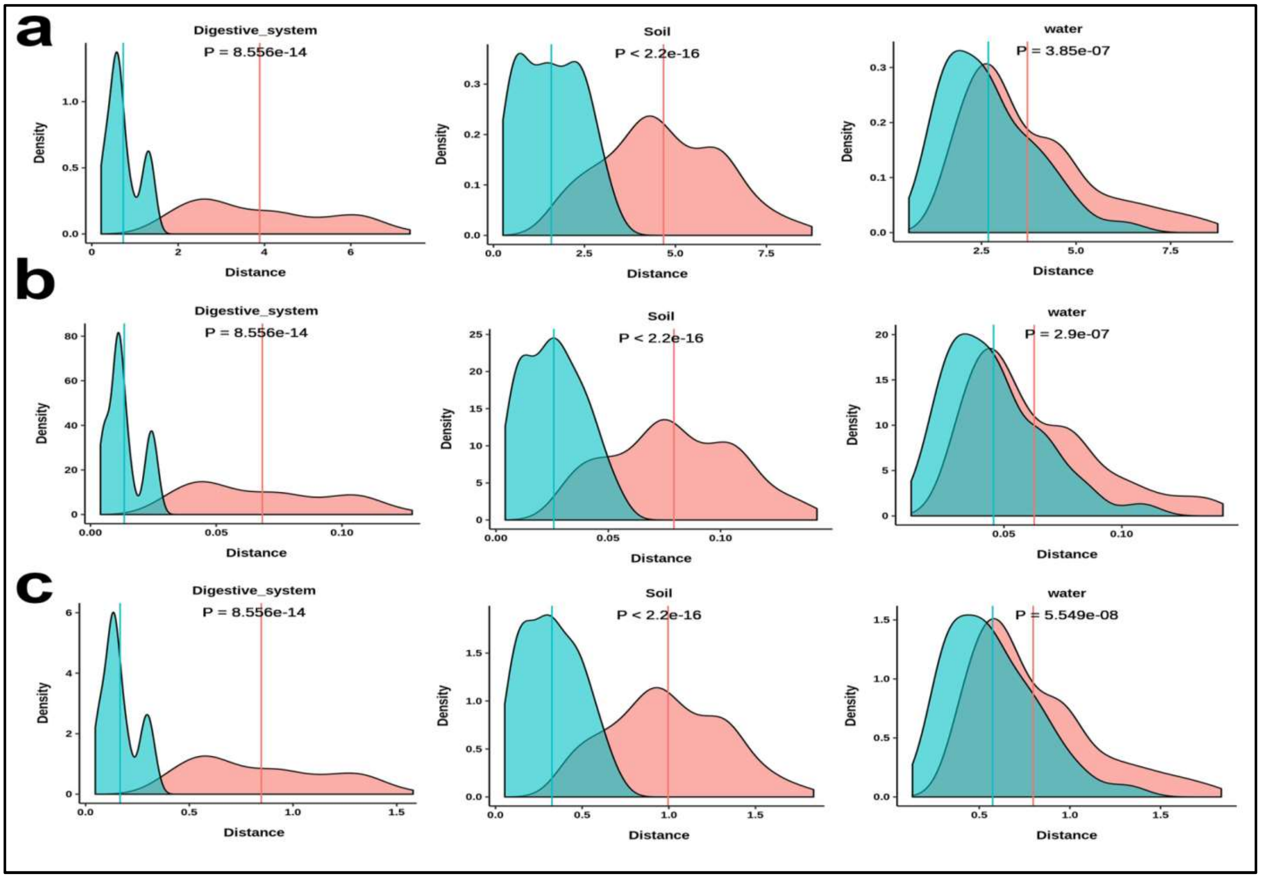

Supplementary Figure 15. Density plots showing amino acid usage distances among the samples sequenced by the non-Illumina method. This figure shows the density plots of amino acid usage distances among the test metagenomic samples sequenced by non-Illumina methods. **a.** Distances in the codon frequencies were calculated by Euclidean distance method. **b.** Distances in codon frequencies were

calculated as Bray-Curtis dissimilarity method **c**. Distances calculated by Endres–Schindelin distance method. For each sample in each selected habitat, we calculated all possible pair-wise codon usage distances (by these two methods separately) with all other samples either from the same (within-group comparison) or different habitats (between groups comparison). Next, we compared the within-group distances of codon usage with that of between-group distances habitat-wise. Statistical significance of the differences for pair-wise comparison of within to between-group distances was accessed by Mann-Whitney U test and the corresponding *P*-values were shown in the respective panel. In each panel, vertical lines represent the average of within and between-groups distances in codon usage frequencies, respectively. Blue lines stand for within-group distances and pink for between-group distances, respectively.

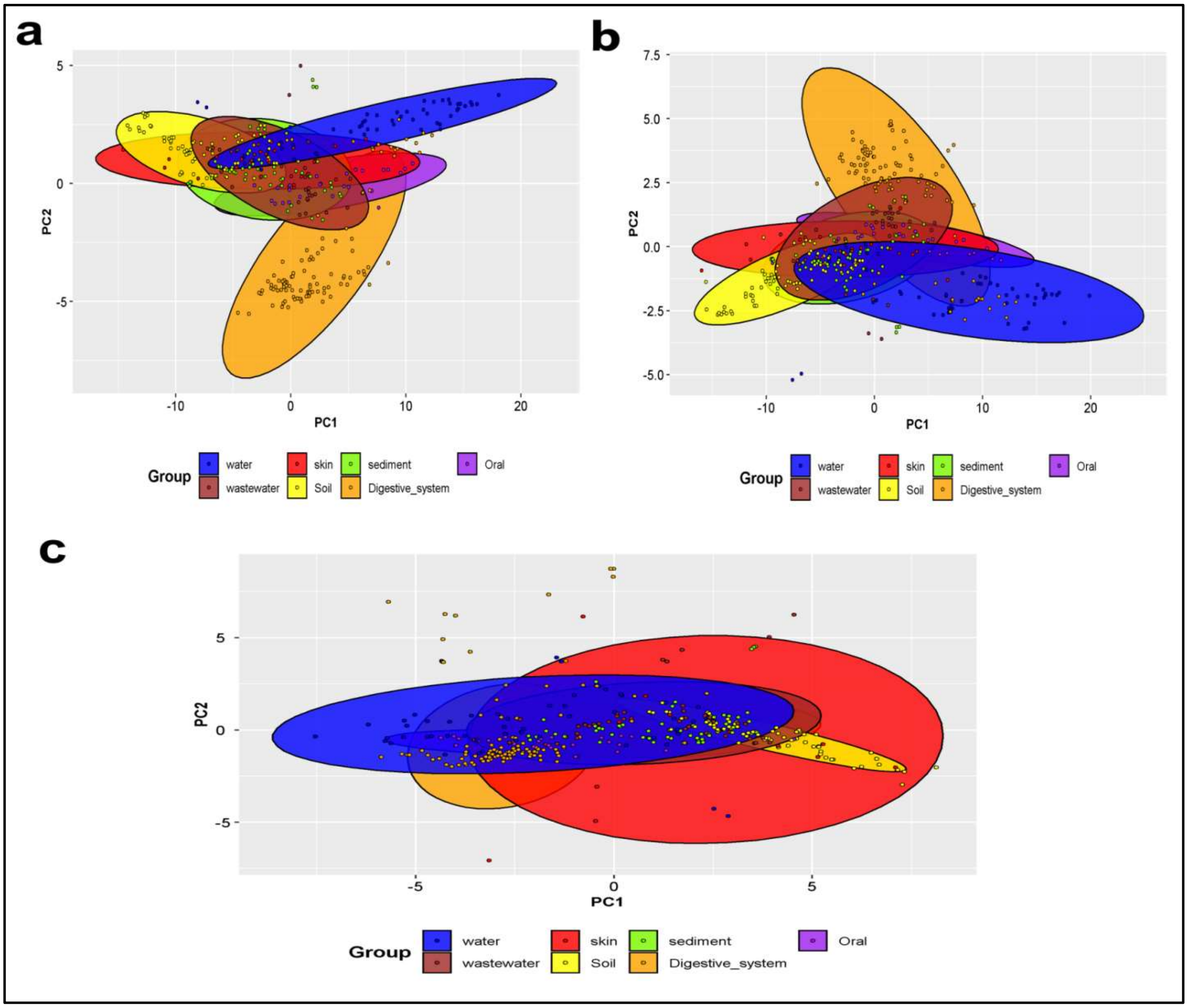

Supplementary Figure 16. Principal component analysis (PCA) on the codon and amino acid usage frequencies of samples sequenced by the Illumina method. PCA analysis was applied on the **a.** absCUFs **b.** synCUFs frequencies, and **c.** amino acid frequencies of samples sequenced by the Illumina method. Each point in the figure represents one sample. The color scheme is shown in the legend. The first two components of the PCA are presented here. The first two components (PC1 and PC2) can explain more than 70% of the variance in the respective datasets.

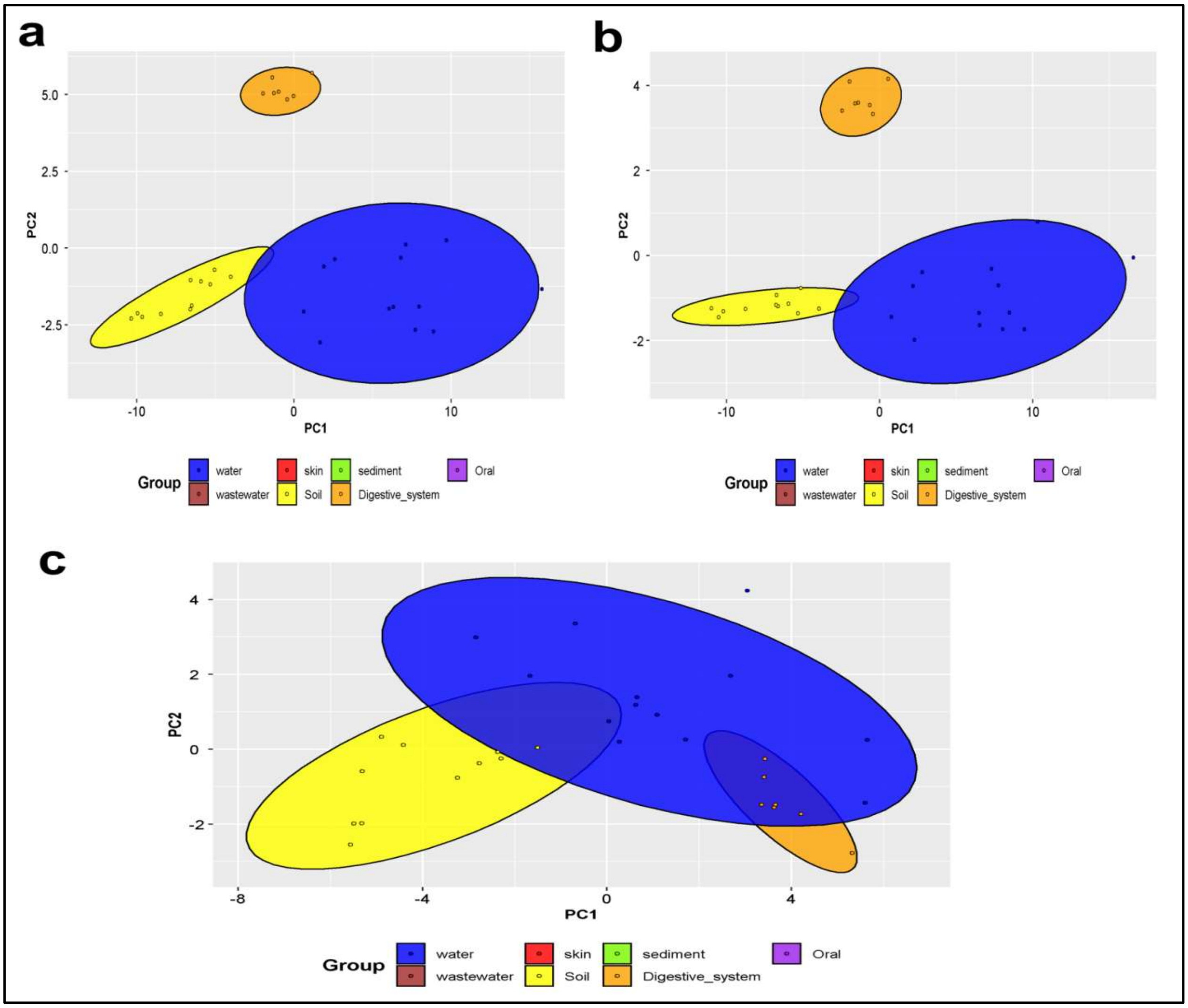

Supplementary Figure 17. Principal component analysis (PCA) on the codon and amino acid usage frequencies of samples sequenced by the non-Illumina method. PCA analysis was applied on the **a.** absCUFs **b.** synCUFs and **c.** amino acid frequencies of samples sequenced by the non-Illumina method. Each point in the figure represents one sample. The color scheme is shown in the legend. The first two components of the PCA are presented here. The first two components (PC1 and PC2) can explain more than 70% of the variance in the respective datasets.

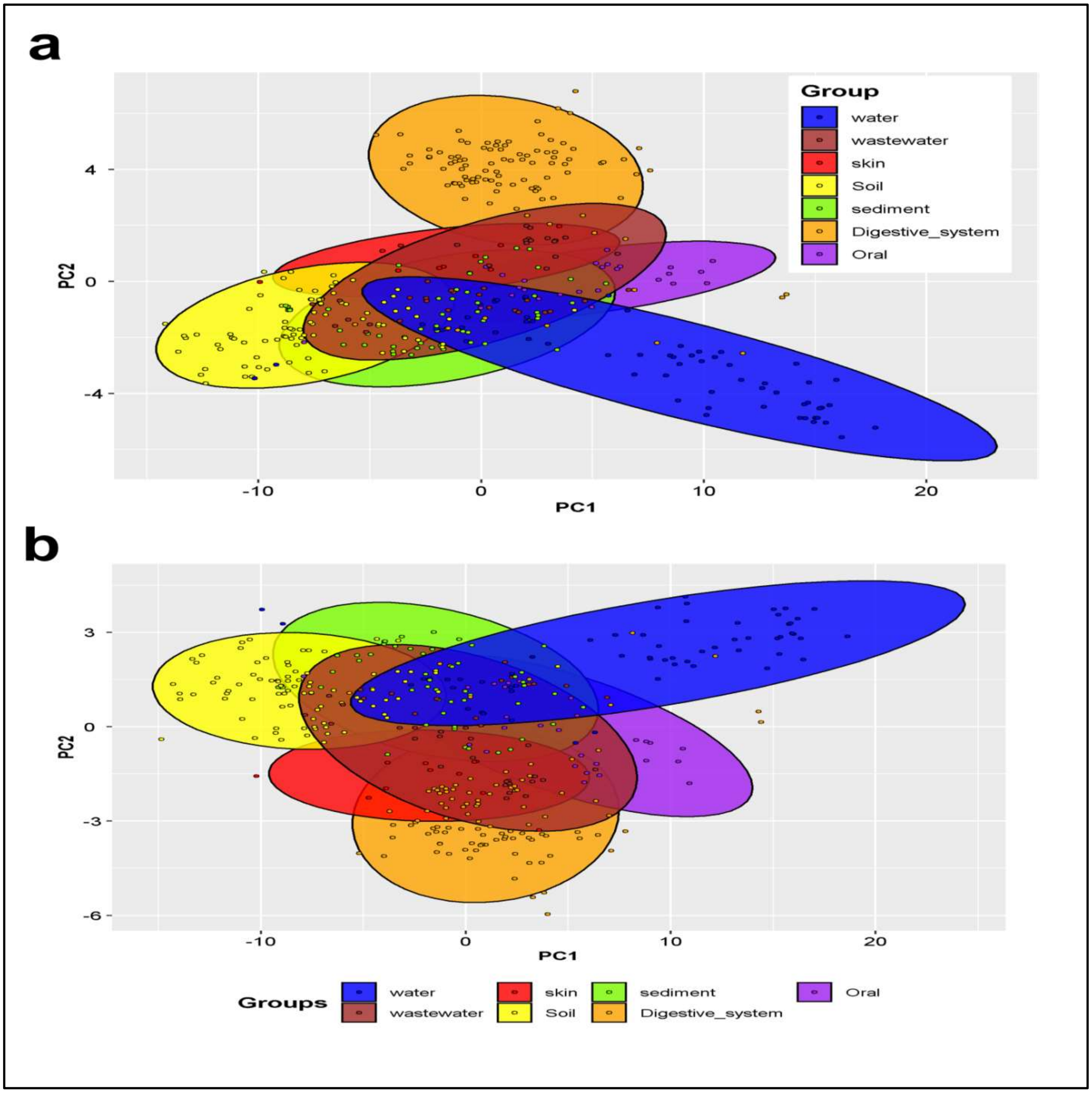

Supplementary Figure 18. Principal component analysis (PCA) on the codon usage frequencies of probable ribosomal protein coding genes in the test metagenomic samples. PCA analysis was applied on the **a.** absCUFs **b.** synCUFs frequencies of probable ribosomal protein coding genes of our test metagenomic samples. Each point in the figure represents one sample. The color scheme is shown in the legend. The first two components of the PCA are presented here.

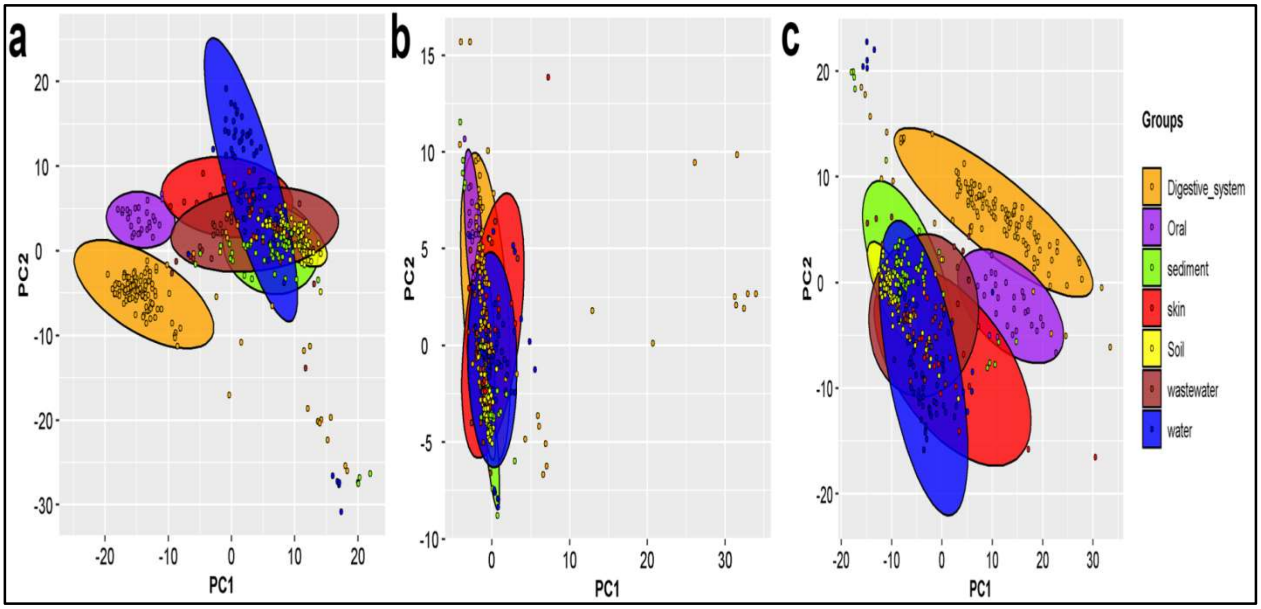

Supplementary Figure 19. Principal component analysis (PCA) based on the functional abundance of samples from seven environmental biomes. PCA analysis was applied on the frequencies of selected Gene Ontology (GO) terms of samples from seven biomes. **a.** PCA was done based on the relative abundance of 500 GO biological process terms, **b.** 100 GO cellular component terms, and **c.** 500 GO molecular function terms. Each point in the figure represents one sample. Samples are colored according to the environmental feature from where those were collected. The color scheme is shown in the legend. The first two components of the PCA are presented here. The first two components (PC1 and PC2) can explain more than 70% of the variance in GO term's frequencies in the dataset.

Supplementary tables:

Supplementary Table 1. ANOSIM results for codon/amino acid usage distances of microorganisms collected from the fusionDB database. Here we compared codon/amino usage distances among 925 microorganisms collected from the fusionDB database. These microorganisms were grouped according to their ecological niches following the classification offered in the database broadly, then their codon usage frequencies were compared within and between the groups using ANOSIM test. In each microorganism, codon usage frequencies were calculated by two methods (i) absCUFs, and (ii) synCUFs frequencies (see main text). ANOSIM test was carried out considering 10,000 permutation values and two distance matrices separately (i) Euclidean, and (ii) Bray-Curtis dissimilarity matrices. Therefore, there are two columns for each type of frequency (absCUFs/synCUFs/amino acid). N1 and N2 represent the number of samples in the first and second habitat, respectively. ANOSIM test statistics are shown with R-values and significance levels are shown with *P*-values.

Habitat1	N1	Habitat2	N2	R-values for absCUFs based on Bray-Curtis dissimilarity	R-values for absCUFs based on Euclidean distance	R-values for synCUFs based on Bray-Curtis dissimilarity	R-values for synCUFs based on Euclidean distance	R-values for amino acid based on Bray-Curtis dissimilarity	R-values for amino acid based on Euclidean distance

Fresh water	110	Intestinal microflora	66	R = 0.0155; *P* = 0.1708	R = 0.0088; *P* = 0.2728	R = 0.0125; *P* = 0.1995	R = 0.0133; *P* = 0.2025	R = 0.026; *P* = 0.103	R = 0.0214; *P* = 0.1436
Fresh water	110	Marine	85	R = 0.0325; *P* = 0.0116	R = 0.0253; *P* = 0.024	R = 0.0326; *P* = 0.0099	R = 0.0299; *P* = 0.0138	R = 0.0215; *P* = 0.0427	R = 0.0195; *P* = 0.0558
Fresh water	110	Multi	198	R = 0.0159; *P* = 0.0639	R = 0.0178; *P* = 0.057	R = 0.0137; *P* = 0.0882	R = 0.0163; *P* = 0.0694	R = 0.036; *P* = 0.01	R = 0.0362; *P* = 0.01
Fresh water	110	Nasopharyngeal microflora	24	R = 0.0443; *P* = 0.0926	R = 0.0254; *P* = 0.2336	R = 0.0687; *P* = 0.0262	R = 0.0573; *P* = 0.0649	R = -0.0326; *P* = 0.7562	R = -0.0475; *P* = 0.861
Fresh water	110	nonspecific host	335	R = 0.0872; *P* = 0.0001	R = 0.0821; *P* = 0.0001	R = 0.0976; *P* = 0.0001	R = 0.1038; *P* = 0.0001	R = 0.0382; *P* = 0.0337	R = 0.0439; *P* = 0.0168
Fresh water	110	Soil	107	R = 0.0655; *P* = 0.0003	R = 0.0655; *P* = 0.0001	R = 0.0759; *P* = 0.0001	R = 0.0821; *P* = 0.0001	R = 0.0659; *P* = 0.0002	R = 0.0669; *P* = 0.0003
Intestinal microflora	66	Marine	85	R = 0.0263; *P* = 0.0443	R = 0.0262; *P* = 0.047	R = 0.0244; *P* = 0.0572	R = 0.0362; *P* = 0.0204	R = 0.0203; *P* = 0.0869	R = 0.0146; *P* = 0.1385
Intestinal microflora	66	Multi	198	R = -0.0385; *P* = 0.9939	R = -0.0462; *P* = 0.9949	R = -0.0395; *P* = 0.9959	R = -0.0386; *P* = 0.9913	R = -0.0633; *P* = 0.9989	R = -0.0681; *P* = 0.9996

Intestinal microflora	66	Nasopharyngeal microflora	24	R = 0.0944; *P* = 0.0229	R = 0.0842; *P* = 0.0438	R = 0.0815; *P* = 0.0498	R = 0.0593; *P* = 0.1143	R = 0.0961; *P* = 0.0213	R = 0.0937; *P* = 0.0214
Intestinal microflora	66	nonspecific host	335	R = -0.0265; *P* = 0.8678	R = -0.0378; *P* = 0.9314	R = -0.0099; *P* = 0.6377	R = -0.0026; *P* = 0.5108	R = -0.1236; *P* = 1	R = -0.1289; *P* = 1
Intestinal microflora	66	Soil	107	R = 0.1776; *P* = 0.0001	R = 0.1586; *P* = 0.0001	R = 0.1726; *P* = 0.0001	R = 0.172; *P* = 0.0001	R = 0.183; *P* = 0.0001	R = 0.1809; *P* = 0.0001
Marine	85	Multi	198	R = -0.0059; *P* = 0.6447	R = -0.0145; *P* = 0.8144	R = -0.012; *P* = 0.8149	R = -0.0185; *P* = 0.8984	R = 0.0198; *P* = 0.15	R = 0.0152; *P* = 0.1937
Marine	85	Nasopharyngeal microflora	24	R = -0.068; *P* = 0.9324	R = -0.0726; *P* = 0.9268	R = -0.0595; *P* = 0.8939	R = -0.0613; *P* = 0.887	R = -0.0923; *P* = 0.9744	R = -0.1079; *P* = 0.9906
Marine	85	nonspecific host	335	R = -0.021; *P* = 0.8365	R = -0.0323; *P* = 0.9291	R = -0.0159; *P* = 0.7565	R = -0.02; *P* = 0.805	R = -0.0275; *P* = 0.8788	R = -0.0251; *P* = 0.8514
Marine	85	Soil	107	R = 0.1827; *P* = 0.0001	R = 0.1679; *P* = 0.0001	R = 0.1929; *P* = 0.0001	R = 0.1931; *P* = 0.0001	R = 0.138; *P* = 0.0001	R = 0.1387; *P* = 0.0001

Multi	198	Nasopharyngeal microflora	24	R = -0.0774; *P* = 0.9987	R = -0.0934; *P* = 0.9983	R = -0.0705; *P* = 0.9979	R = -0.0862; *P* = 0.9991	R = -0.1061; *P* = 0.9991	R = -0.1142; *P* = 0.9997
Multi	198	nonspecific host	335	R = 0.0366; *P* = 0.0006	R = 0.0341; *P* = 0.0014	R = 0.0424; *P* = 0.0002	R = 0.0442; *P* = 0.0002	R = -0.0047; *P* = 0.6732	R = -0.0023; *P* = 0.5734
Multi	198	Soil	107	R = 0.0822; *P* = 0.0001	R = 0.0796; *P* = 0.0001	R = 0.0924; *P* = 0.0001	R = 0.0995; *P* = 0.0001	R = 0.1056; *P* = 0.0001	R = 0.0952; *P* = 0.0001
Nasopharyngeal microflora	24	nonspecific host	335	R = -0.1928; *P* = 1	R = -0.1999; *P* = 1	R = -0.1779; *P* = 1	R = -0.1883; *P* = 1	R = -0.2241; *P* = 1	R = -0.2179; *P* = 1
Nasopharyngeal microflora	24	Soil	107	R = 0.2338; *P* = 0.0001	R = 0.2004; *P* = 0.0001	R = 0.252; *P* = 0.0001	R = 0.2366; *P* = 0.0001	R = 0.1346; *P* = 0.0001	R = 0.1272; *P* = 0.0001
nonspecific host	335	Soil	107	R = 0.2006; *P* = 0.0001	R = 0.1913; *P* = 0.0001	R = 0.2267; *P* = 0.0001	R = 0.2346; *P* = 0.0001	R = 0.1197; *P* = 0.0001	R = 0.1188; *P* = 0.0001

Supplementary Table 2. Results of permutation test for codon usage frequencies. For each group of the test samples, we calculated an index value CI (Clustering Index) which is the ratio of average distance in codon usage frequencies among the samples from the same environmental group to that of samples from all other environmental groups. Codon usage frequencies among the samples were calculated by two approaches absCUFs and synCUFs method. Therefore, there are two columns for CI values corresponding to distances calculated for each of these methods. Distances in codon usage frequencies among the samples were calculated by Endres–Schindelin method. To test the significance level, we generated 1,000 random datasets by randomly assigning the samples in different environmental groups keeping the number of samples in each group unchanged. For samples from each test environmental group, CI values were calculated from the random datasets following the same approach as the original dataset. For significance levels, we considered empirical *P*-values, defined as the number of times the CI calculated from the random dataset was higher than that calculated from the real dataset divided by the number of random datasets (1000).

**Environmental group**	**Number of samples**	**CI index based on absCUFs**	**Associated *P*-values**	**CI index based on synCUFs**	**Associated *P*-values**
Water	67	0.811	$P = 0$	0.788	$P = 0$
Skin	15	0.999	$P = 0.48$	0.965	$P = 0.33$
Digestive system	122	0.651	$P = 0$	0.702	$P = 0$
Soil	78	0.439	$P = 0$	0.470	$P = 0$

Wastewater	43	0.690	$P = 0$	0.660	$P = 0$
Oral	26	0.721	$P = 0$	0.740	$P = 0$
Sediment	56	0.655	$P = 0$	0.650	$P = 0$

Supplementary Table 3. ANOSIM results for amino acid usage distance comparisons among the samples from seven test habitats. ANOSIM test was carried out considering the amino acid usage frequencies of samples from two habitats at a time using Euclidean distance and Bray-Curtis dissimilarity matrix separately with 10,000 permutation values. Here N_1 and N_2 represent the number of samples in the first and second habitat, respectively. ANOSIM test statistics are shown with R-values and significance levels are shown with *P*-values.

**Habitat1**	**N1**	**Habitat2**	**N2**	**ANOSIM R-values for amino acid frequencies based on Euclidean distance**	**ANOSIM R-values for amino acid frequencies based on Bray-Curtis dissimilarity**

Digestive system	122	Oral	27	R = 0.1829; *P* = 5.60E-03	R = 0.1965; *P* = 2.90E-03
Digestive system	122	Sediment	60	R = 0.5535; *P* = 1.00E-04	R = 0.5836; *P* = 1.00E-04
Digestive system	122	Skin	15	R = 0.7706; *P* = 1.00E-04	R = 0.7905; *P* = 1.00E-04
Digestive system	122	Soil	85	R = 0.8701; *P* = 1.00E-04	R = 0.8713; *P* = 1.00E-04
Digestive system	122	wastewater	45	R = 0.5172; *P* = 1.00E-04	R = 0.5205; *P* = 1.00E-04
Digestive system	122	Water	68	R = 0.3634; *P* = 1.00E-04	R = 0.3938; *P* = 1.00E-04
Oral	27	Sediment	60	R = 0.3094; *P* = 1.00E-04	R = 0.3477; *P* = 1.00E-04
Oral	27	Skin	15	R = 0.5498; *P* = 1.00E-04	R = 0.5649; *P* = 1.00E-04
Oral	27	Soil	85	R = 0.7596; *P* = 1.00E-04	R = 0.7602; *P* = 1.00E-04

Oral	27	wastewater	45	R = 0.2043; P = 1.00E-04	R = 0.2139; P = 3.00E-04
Oral	27	Water	68	R = -0.0165; P = 6.17E-01	R = 0.0017; P = 4.45E-01
Sediment	60	Skin	15	R = 0.3653; P = 2.00E-04	R = 0.3825; P = 1.00E-04
Sediment	60	Soil	85	R = 0.295; P = 1.00E-04	R = 0.2715; P = 1.00E-04
Sediment	60	wastewater	45	R = 0.0826; P = 1.10E-03	R = 0.0788; P = 2.30E-03
Sediment	60	water	68	R = 0.2269; P = 1.00E-04	R = 0.2316; P = 1.00E-04
Skin	15	Soil	85	R = 0.4118; P = 2.00E-04	R = 0.4335; P = 2.00E-04
Skin	15	wastewater	45	R = 0.2792; P = 9.00E-04	R = 0.3034; P = 6.00E-04
Skin	15	Water	68	R = 0.3589; P = 1.00E-04	R = 0.3645; P = 1.00E-04

Soil	85	wastewater	45	R = 0.3782; *P* = 1.00E-04	R = 0.3663; *P* = 1.00E-04
Soil	85	Water	68	R = 0.5963; *P* = 1.00E-04	R = 0.5853; *P* = 1.00E-04
wastewater	45	Water	68	R = 0.1522; *P* = 2.00E-04	R = 0.1359; *P* = 2.00E-04

Supplementary Table 4. Results of permutation test for amino acid usage frequencies among the test samples. For this test, samples are first grouped according to their environmental biomes. For each group of the test samples, we calculated an index value CI (clustering index) which is the ratio of average distance in amino acid usage frequencies among the samples from the same environmental group to that of samples from all other environmental groups (details in the main text). Distances in amino acid usage frequencies among the samples were calculated by Endres–Schindelin method. To test the significance level, we generated 1,000 random datasets by randomly assigning the samples in different environmental groups keeping the number of samples in each group unchanged. For samples from each test environmental group, CI values were calculated from the random datasets following the same approach as the original dataset. For significance levels, we considered empirical *P*-values, defined as the number of times the CI calculated from the random dataset was higher than that calculated from the real dataset divided by the number of random datasets (1000).

Environmental group	Number of samples	CI index based on amino acid frequencies	Associated *P*-values
Water	67	0.885	$P = 0$
Skin	15	1.004	$P = 0.33$
Digestive system	122	0.541	$P = 0$
Soil	78	0.437	$P = 0$
Wastewater	43	0.747	$P = 0$
Oral	26	0.641	$P = 0$
Sediment	56	0.666	$P = 0$

Supplementary Table 5. ANOSIM results for codon/amino acid usage distances based on collected CDS sequences from MGnify metagenomic database. Here we compared codon and amino acid usage distances among the test samples from seven ecological niches using ANOSIM test. Predicted CDS and protein sequences of each sample were collected from MGnify metagenomic database. Codon usage frequencies were calculated by two approaches absCUFs and synCUFs method (see main text). ANOSIM test was carried out considering the codon or amino acid frequencies of samples from two habitats at a time using Euclidean distance and Bray-Curtis dissimilarity measures separately with 10,000 permutation values. Therefore, there are two columns for the test results (one for Euclidean distance another for Bray-

Curtis dissimilarity) for codon (absCUFs or synCUFs) and amino acid usage frequencies. Here N_1 and N_2 represent the number of samples in the first and second habitat, respectively. ANOSIM test statistics are shown with R-values and significance levels are shown with *P*-values.

Habitat-1	N_1	Habitat-2	N_2	ANOSIM R-values for absCUFs based on Euclidean distance	ANOSIM *R*-values for synCUFs based on Euclidean distance	ANOSIM R-values for absCUFs based on Bray-Curtis dissimilarity	ANOSIM *R*-values for synCUFs based on Bray-Curtis dissimilarity	ANOSIM R-values for amino acid frequencies based on Euclidean distance	ANOSIM R -values for amino acid frequencies based on Bray-Curtis dissimilarity
Digestive system	122	Oral	26	R = 0.1273; *P* = 2.27E-02	R = 0.1321; *P* =1.14E-02	R = 0.1347; *P* = 0.0139	R = 0.1074; *P* = 0.0246	R = 0.1064; *P* = 6.78E-2	R = 0.1218; *P* = 4.51E-2
Digestive system	122	Sediment	56	R = 0.3319; *P* = 1.00E-04	R = 0.2252; *P* = 1.00E-04	R = 0.3307; *P* = 0.0001	R = 0.2343; *P* = 0.0001	R = 0.5077; *P* = 1.00E-4	R = 0.5304; *P* = 1.00E-4
Digestive system	122	Skin	15	R = 0.4422; *P* = 1.00E-04	R = 0.3788; *P* = 2.00E-04	R = 0.4476; *P* = 0.0001	R = 0.3547; *P* = 0.0001	R = 0.5576; *P* = 1.00E-4	R = 0.5849; *P* = 1.00E-4
Digestive system	122	Soil	78	R = 0.6935; *P* = 1.00E-04	R = 0.5875; *P* = 1.00E-04	R = 0.6834; *P* = 0.0001	R = 0.6037; *P* = 0.0001	R = 0.7817; *P* = 1.00E-4	R = 0.7858; *P* = 1.00E-4

Digestive system	122	Wastewater	43	R = 0.2359; *P* = 1.00E-04	R = 0.1423; *P* = 1.60E-03	R = 0.2248; *P* = 0.0001	R = 0.1302; *P* = 0.0032	R = 0.4221; *P* = 1.00E-4	R = 0.4188; *P* = 1.00E-4
Digestive system	122	Water	67	R = 0.306; *P* = 1.00E-04	R = 0.2649; *P* = 1.00E-04	R = 0.3076; *P* = 0.0001	R = 0.2441; *P* = 0.0001	R = 0.3001; *P* = 1.00E-4	R = 0.3215; *P* = 1.00E-4
Oral	26	Sediment	56	R = 0.4749; *P* = 1.00E-04	R = 0.4711; *P* = 1.00E-04	R = 0.4809; *P* = 0.0001	R = 0.4692; *P* = 0.0001	R = 0.4513; *P* = 1.00E-4	R = 0.4742; *P* = 1.00E-4
Oral	26	Skin	15	R = 0.313; *P* = 3.00E-04	R = 0.2597; *P* = 8.00E-04	R = 0.2949; *P* = 0.0004	R = 0.2388; *P* = 0.0018	R = 0.3672; *P* = 2.00E-4	R = 0.3751; *P* = 1.00E-4
Oral	26	Soil	78	R = 0.7983; *P* = 1.00E-04	R = 0.7517; *P* = 1.00E-04	R = 0.7992; *P* = 0.0001	R = 0.7628; *P* = 0.0001	R = 0.7672; *P* = 1.00E-4	R = 0.7633; *P* = 1.00E-4
Oral	26	Wastewater	43	R = 0.2337; *P* = 1.00E-04	R = 0.2408; *P* = 1.00E-04	R = 0.2158; *P* = 0.0002	R = 0.2297; *P* = 0.0001	R = 0.1675; *P* = 1.20E-3	R = 0.159; *P* = 9.00E-4
Oral	26	Water	67	R = -0.0210; *P* = 6.36E-01	R = -0.0163; *P* = 6.04E-01	R = -0.0091; *P* = 0.5476	R = -0.0219; *P* = 0.6498	R = -0.0389; *P* = 7.65E-1	R = -0.0191; *P* = 6.26E-1
Sediment	56	Skin	15	R = 0.4844; *P* = 1.00E-04	R = 0.4909; *P* = 1.00E-04	R = 0.4898; *P* = 0.0001	R = 0.4761; *P* = 0.0001	R = 0.4223; *P* = 1.00E-4	R = 0.4708; *P* = 1.00E-4
Sediment	56	Soil	78	R = 0.246; *P* = 1.00E-04	R = 0.2249; *P* = 1.00E-04	R = 0.2437; *P* = 0.0001	R = 0.2283; *P* = 0.0001	R = 0.2273; *P* = 1.00E-4	R = 0.2152; *P* = 1.00E-4

Sediment	56	Wastewater	43	R = 0.1116; *P* = 3.00E-04	R = 0.1039; *P* = 1.00E-03	R = 0.1071; *P* = 0.0003	R = 0.1014; *P* = 0.0005	R = 0.1134; *P* = 1.00E-4	R = 0.1114; *P* = 6.00E-4
Sediment	56	Water	67	R = 0.3305; *P* = 1.00E-04	R = 0.3591; *P* = 1.00E-04	R = 0.3563; *P* = 0.0001	R = 0.3631; *P* = 0.0001	R = 0.1956; *P* = 1.00E-4	R = 0.198; *P* = 1.00E-4
Skin	15	Soil	78	R = 0.6334; *P* = 1.00E-04	R = 0.5883; *P* = 1.00E-04	R = 0.6359; *P* = 0.0001	R = 0.5922; *P* = 0.0001	R = 0.529; *P* = 1.00E-4	R = 0.5505; *P* = 1.00E-4
Skin	15	Wastewater	43	R = 0.3067; *P* = 2.00E-04	R = 0.3453; *P* = 1.00E-04	R = 0.2997; *P* = 0.0005	R = 0.3161; *P* = 0.0001	R = 0.2281; *P* = 5.00E-3	R = 0.2444; *P* = 4.40E-3
Skin	15	Water	67	R = 0.2039; *P* = 6.40E-03	R = 0.2021; *P* = 6.50E-03	R = 0.2244; *P* = 0.003	R = 0.1875; *P* = 0.0073	R = 0.2184; *P* = 4.70E-3	R = 0.2444; *P* = 2.80E-3
Soil	78	Wastewater	43	R = 0.4273; *P* = 1.00E-04	R = 0.3995; *P* = 1.00E-04	R = 0.4256; *P* = 0.0001	R = 0.4052; *P* = 0.0001	R = 0.3536; *P* = 1.00E-4	R = 0.3429; *P* = 1.00E-4
Soil	78	Water	67	R = 0.6433; *P* = 1.00E-04	R = 0.6536; *P* = 1.00E-04	R = 0.6655; *P* = 0.0001	R = 0.6601; *P* = 0.0001	R = 0.5241; *P* = 1.00E-4	R = 0.5125; *P* = 1.00E-4
Wastewater	43	Water	67	R = 0.1878; *P* = 2.00E-04	R = 0.2023; *P* = 1.00E-04	R = 0.1992; *P* = 0.0001	R = 0.2011; *P* = 0.0001	R = 0.0992; *P* = 1.70E-3	R = 0.0854; *P* = 4.50E-3

Supplementary Table 6. ANOSIM results for codon/amino acid usage distances considering sequences predicted from subsampled reads. Here we compared codon and amino acid usage distances among the test samples from seven ecological niches using ANOSIM test. CDS/protein sequences were predicted considering 0.1%, 1%, 5%, and 10% of all reads randomly in each sample following similar steps as described for CDS prediction from all reads without any subsampling. Codon usage frequencies were calculated by two approaches absCUFs and synCUFs method (see main text). ANOSIM test was carried out considering the codon or amino acid frequencies of samples from two habitats at a time using Euclidean distance matrix and Bray-Curtis dissimilarity measure separately with 10,000 permutation values. Therefore, there are two columns for the test results (one for Euclidean distance another for Bray-Curtis dissimilarity) for codon (absCUFs or synCUFs) and amino acid usage frequencies. Here N1 and N2 represent the number of samples in the first and second habitat, respectively. ANOSIM test statistics are shown with R-values and significance levels are shown with *P*-values.

**SUBSAMPLE set**	**Habitat1**	**N1**	**Habitat2**	**N2**	**ANOSIM R-values for Amino acid frequencies based on Bray-Curtis dissimilarity**	**ANOSIM R-values for Amino acid frequencies based on Euclidean distance**	**ANOSIM R-values for absCUFs frequencies based on Bray-Curtis dissimilarity**	**ANOSIM R-values for absCUFs frequencies based on Euclidean distance**	**ANOSIM R-values for synCUFs frequencies based on Bray-Curtis dissimilarity**	**ANOSIM R-values for synCUFs frequencies based on Euclidean distance**
SET=0.1	Digestive	122	Oral	27	R = 0.2121;	R = 0.19;	R = 0.1518;	R = 0.1557;	R = 0.1201;	R = 0.1477;

%	system				*P* = 2.30E-03	*P* = 5.30E-03	*P* = 7.50E-03	*P* = 6.10E-03	*P* = 1.58E-02	*P* = 5.20E-03
SET=0.1 %	Digestive system	122	sediment	60	R = 0.5743; *P* = 1.00E-04	R = 0.5507; *P* = 1.00E-04	R = 0.282; *P* = 1.00E-04	R = 0.2953; *P* = 1.00E-04	R = 0.1611; *P* = 1.00E-04	R = 0.1553; *P* = 1.00E-04
SET=0.1 %	Digestive system	122	skin	15	R = 0.6571; *P* = 1.00E-04	R = 0.6316; *P* = 1.00E-04	R = 0.4013; *P* = 1.00E-04	R = 0.4223; *P* = 2.00E-04	R = 0.2798; *P* = 8.00E-04	R = 0.3035; *P* = 6.00E-04
SET=0.1 %	Digestive system	122	Soil	85	R = 0.8277; *P* = 1.00E-04	R = 0.8202; *P* = 1.00E-04	R = 0.6526; *P* = 1.00E-04	R = 0.6697; *P* = 1.00E-04	R = 0.5519; *P* = 1.00E-04	R = 0.5327; *P* = 1.00E-04
SET=0.1 %	Digestive system	122	wastewate r	45	R = 0.492; *P* = 1.00E-04	R = 0.4909; *P* = 1.00E-04	R = 0.2301; *P* = 1.00E-04	R = 0.2463; *P* = 1.00E-04	R = 0.1205; *P* = 3.40E-03	R = 0.1288; *P* = 3.10E-03
SET=0.1 %	Digestive system	122	water	68	R = 0.3808; *P* = 1.00E-04	R = 0.3556; *P* = 1.00E-04	R = 0.3536; *P* = 1.00E-04	R = 0.3559; *P* = 1.00E-04	R = 0.2906; *P* = 1.00E-04	R = 0.3047; *P* = 1.00E-04
SET=0.1 %	Oral	27	sediment	60	R = 0.3599; *P* = 1.00E-04	R = 0.332; *P* = 1.00E-04	R = 0.357; *P* = 1.00E-04	R = 0.351; *P* = 1.00E-04	R = 0.3433; *P* = 1.00E-04	R = 0.3501; *P* = 1.00E-04
SET=0.1 %	Oral	27	skin	15	R = 0.3625; *P* = 2.00E-04	R = 0.3495; *P* = 3.00E-04	R = 0.2677; *P* = 7.00E-04	R = 0.2891; *P* = 5.00E-04	R = 0.2036; *P* = 5.80E-03	R = 0.2131; *P* = 5.10E-03
SET=0.1 %	Oral	27	Soil	85	R = 0.7225; *P* = 1.00E-04	R = 0.7131; *P* = 1.00E-04	R = 0.7371; *P* = 1.00E-04	R = 0.7365; *P* = 1.00E-04	R = 0.6998; *P* = 1.00E-04	R = 0.6935; *P* = 1.00E-04

SET=0.1%	Oral	27	wastewater	45	R = 0.1933; *P* = 4.00E-04	R = 0.1899; *P* = 2.00E-04	R = 0.2054; *P* = 4.00E-04	R = 0.2179; *P* = 1.00E-04	R = 0.2072; *P* = 2.00E-04	R = 0.2197; *P* = 1.00E-04
SET=0.1%	Oral	27	water	68	R = -0.02705; *P* = 6.99E-01	R = -0.04541; *P* = 8.39E-01	R = 0.008457; *P* = 3.81E-01	R = -0.005265; *P* = 5.02E-01	R = 0.004209; *P* = 4.14E-01	R = 0.01321; *P* = 3.47E-01
SET=0.1%	Sediment	60	skin	15	R = 0.3161; *P* = 1.00E-03	R = 0.2822; *P* = 2.00E-03	R = 0.3165; *P* = 3.00E-04	R = 0.3211; *P* = 4.00E-04	R = 0.2922; *P* = 3.00E-04	R = 0.3078; *P* = 4.00E-04
SET=0.1%	Sediment	60	Soil	85	R = 0.2219; *P* = 1.00E-04	R = 0.2372; *P* = 1.00E-04	R = 0.2672; *P* = 1.00E-04	R = 0.2676; *P* = 1.00E-04	R = 0.2573; *P* = 1.00E-04	R = 0.2499; *P* = 1.00E-04
SET=0.1%	Sediment	60	wastewater	45	R = 0.073; *P* = 2.40E-03	R = 0.07821; *P* = 2.40E-03	R = 0.06058; *P* = 8.80E-03	R = 0.0653; *P* = 4.60E-03	R = 0.0539; *P* = 1.02E-02	R = 0.05684; *P* = 1.14E-02
SET=0.1%	Sediment	60	water	68	R = 0.2037; *P* = 1.00E-04	R = 0.2008; *P* = 1.00E-04	R = 0.3453; *P* = 1.00E-04	R = 0.3319; *P* = 1.00E-04	R = 0.3447; *P* = 1.00E-04	R = 0.3456; *P* = 1.00E-04
SET=0.1%	Skin	15	Soil	85	R = 0.4619; *P* = 1.00E-04	R = 0.4379; *P* = 1.00E-04	R = 0.5526; *P* = 1.00E-04	R = 0.5556; *P* = 1.00E-04	R = 0.4961; *P* = 1.00E-04	R = 0.4923; *P* = 1.00E-04
SET=0.1%	Skin	15	wastewater	45	R = 0.1843; *P* = 1.28E-02	R = 0.1665; *P* = 2.06E-02	R = 0.1941; *P* = 8.10E-03	R = 0.2144; *P* = 6.30E-03	R = 0.1897; *P* = 8.10E-03	R = 0.2173; *P* = 5.10E-03

SET=0.1 %	Skin	15	water	68	R = 0.1927; *P* = 6.00E-03	R = 0.1763; *P* = 8.70E-03	R = 0.2426; *P* = 7.00E-04	R = 0.242; *P* = 9.00E-04	R = 0.2283; *P* = 9.00E-04	R = 0.2406; *P* = 9.00E-04
SET=0.1 %	Soil	85	wastewater	45	R = 0.3134; *P* = 1.00E-04	R = 0.3197; *P* = 1.00E-04	R = 0.3786; *P* = 1.00E-04	R = 0.3873; *P* = 1.00E-04	R = 0.3546; *P* = 1.00E-04	R = 0.3499; *P* = 1.00E-04
SET=0.1 %	Soil	85	water	68	R = 0.5199; *P* = 1.00E-04	R = 0.5261; *P* = 1.00E-04	R = 0.6493; *P* = 1.00E-04	R = 0.6401; *P* = 1.00E-04	R = 0.6422; *P* = 1.00E-04	R = 0.6395; *P* = 1.00E-04
SET=0.1 %	Wastewater	45	water	68	R = 0.09953; *P* = 1.10E-03	R = 0.1133; *P* = 6.00E-04	R = 0.2339; *P* = 1.00E-04	R = 0.2303; *P* = 1.00E-04	R = 0.2411; *P* = 1.00E-04	R = 0.2431; *P* = 1.00E-04
SET=1%	Digestive system	122	Oral	27	R = 0.205; *P* = 3.00E-03	R = 0.1842; *P* = 4.90E-03	R = 0.1728; *P* = 2.20E-03	R = 0.1769; *P* = 3.50E-03	R = 0.1405; *P* = 6.60E-03	R = 0.1724; *P* = 2.80E-03
SET=1%	Digestive system	122	sediment	60	R = 0.5959; *P* = 1.00E-04	R = 0.5723; *P* = 1.00E-04	R = 0.2949; *P* = 1.00E-04	R = 0.3098; *P* = 1.00E-04	R = 0.1695; *P* = 1.00E-04	R = 0.1675; *P* = 1.00E-04
SET=1%	Digestive system	122	skin	15	R = 0.7212; *P* = 1.00E-04	R = 0.7029; *P* = 1.00E-04	R = 0.4585; *P* = 1.00E-04	R = 0.4805; *P* = 1.00E-04	R = 0.3303; *P* = 1.00E-04	R = 0.3532; *P* = 2.00E-04
SET=1%	Digestive system	122	Soil	85	R = 0.8455; *P* = 1.00E-04	R = 0.8369; *P* = 1.00E-04	R = 0.6794; *P* = 1.00E-04	R = 0.6988; *P* = 1.00E-04	R = 0.5789; *P* = 1.00E-04	R = 0.5634; *P* = 1.00E-04
SET=1%	Digestive system	122	wastewater	45	R = 0.5087; *P* = 1.00E-04	R = 0.5062; *P* = 1.00E-04	R = 0.2494; *P* = 1.00E-04	R = 0.2713; *P* = 1.00E-04	R = 0.1427; *P* = 8.00E-04	R = 0.159; *P* = 2.00E-04

SET=1%	Digestive system	122	water	68	R = 0.3865; *P* = 1.00E-04	R = 0.3623; *P* = 1.00E-04	R = 0.3801; *P* = 1.00E-04	R = 0.3855; *P* = 1.00E-04	R = 0.3167; *P* = 1.00E-04	R = 0.3391; *P* = 1.00E-04
SET=1%	Oral	27	sediment	60	R = 0.3979; *P* = 1.00E-04	R = 0.375; *P* = 1.00E-04	R = 0.3761; *P* = 1.00E-04	R = 0.3738; *P* = 1.00E-04	R = 0.3554; *P* = 1.00E-04	R = 0.3614; *P* = 1.00E-04
SET=1%	Oral	27	skin	15	R = 0.4533; *P* = 1.00E-04	R = 0.4427; *P* = 1.00E-04	R = 0.3395; *P* = 2.00E-04	R = 0.3594; *P* = 1.00E-04	R = 0.2665; *P* = 8.00E-04	R = 0.2806; *P* = 7.00E-04
SET=1%	Oral	27	Soil	85	R = 0.728; *P* = 1.00E-04	R = 0.7179; *P* = 1.00E-04	R = 0.754; *P* = 1.00E-04	R = 0.751; *P* = 1.00E-04	R = 0.7172; *P* = 1.00E-04	R = 0.7084; *P* = 1.00E-04
SET=1%	Oral	27	wastewater	45	R = 0.1827; *P* = 3.00E-04	R = 0.178; *P* = 4.00E-04	R = 0.1943; *P* = 2.00E-04	R = 0.2064; *P* = 2.00E-04	R = 0.1927; *P* = 4.00E-04	R = 0.2019; *P* = 1.00E-04
SET=1%	Oral	27	water	68	R = -0.01571; *P* = 6.14E-01	R = -0.03576; *P* = 7.69E-01	R = 0.02214; *P* = 2.71E-01	R = 0.008232; *P* = 3.90E-01	R = 0.01397; *P* = 3.35E-01	R = 0.02444; *P* = 2.54E-01
SET=1%	Sediment	60	skin	15	R = 0.3405; *P* = 6.00E-04	R = 0.3104; *P* = 7.00E-04	R = 0.3318; *P* = 2.00E-04	R = 0.3377; *P* = 4.00E-04	R = 0.3087; *P* = 3.00E-04	R = 0.3245; *P* = 3.00E-04
SET=1%	Sediment	60	Soil	85	R = 0.2402; *P* = 1.00E-04	R = 0.2541; *P* = 1.00E-04	R = 0.2998; *P* = 1.00E-04	R = 0.2991; *P* = 1.00E-04	R = 0.2886; *P* = 1.00E-04	R = 0.2818; *P* = 1.00E-04

SET=1%	Sediment	60	wastewater	45	R = 0.08761; *P* = 1.00E-03	R = 0.09796; *P* = 5.00E-04	R = 0.07087; *P* = 2.90E-03	R = 0.07848; *P* = 2.20E-03	R = 0.06178; *P* = 7.20E-03	R = 0.06575; *P* = 5.00E-03
SET=1%	Sediment	60	water	68	R = 0.2197; *P* = 1.00E-04	R = 0.2142; *P* = 1.00E-04	R = 0.3611; *P* = 1.00E-04	R = 0.3487; *P* = 1.00E-04	R = 0.3638; *P* = 1.00E-04	R = 0.3658; *P* = 1.00E-04
SET=1%	Skin	15	Soil	85	R = 0.4349; *P* = 1.00E-04	R = 0.3976; *P* = 1.00E-04	R = 0.5494; *P* = 1.00E-04	R = 0.5458; *P* = 1.00E-04	R = 0.5048; *P* = 1.00E-04	R = 0.5006; *P* = 1.00E-04
SET=1%	Skin	15	wastewater	45	R = 0.2038; *P* = 8.20E-03	R = 0.1767; *P* = 1.74E-02	R = 0.2028; *P* = 7.20E-03	R = 0.2209; *P* = 6.20E-03	R = 0.2026; *P* = 7.90E-03	R = 0.2267; *P* = 4.50E-03
SET=1%	Skin	15	water	68	R = 0.2503; *P* = 1.90E-03	R = 0.2335; *P* = 8.00E-04	R = 0.3019; *P* = 1.00E-04	R = 0.3058; *P* = 1.00E-04	R = 0.2875; *P* = 4.00E-04	R = 0.3027; *P* = 2.00E-04
SET=1%	Soil	85	wastewater	45	R = 0.327; *P* = 1.00E-04	R = 0.3342; *P* = 1.00E-04	R = 0.4049; *P* = 1.00E-04	R = 0.4128; *P* = 1.00E-04	R = 0.3808; *P* = 1.00E-04	R = 0.3784; *P* = 1.00E-04
SET=1%	Soil	85	water	68	R = 0.5308; *P* = 1.00E-04	R = 0.5332; *P* = 1.00E-04	R = 0.6716; *P* = 1.00E-04	R = 0.6621; *P* = 1.00E-04	R = 0.6684; *P* = 1.00E-04	R = 0.6669; *P* = 1.00E-04
SET=1%	Wastewater	45	water	68	R = 0.1109; *P* = 8.00E-04	R = 0.1216; *P* = 3.00E-04	R = 0.2452; *P* = 1.00E-04	R = 0.2435; *P* = 1.00E-04	R = 0.2528; *P* = 1.00E-04	R = 0.2582; *P* = 1.00E-04
SET=5%	Digestive system	122	Oral	27	R = 0.2203; *P* = 5.00E-04	R = 0.1986; *P* = 1.60E-03	R = 0.1799; *P* = 2.40E-03	R = 0.1863; *P* = 1.70E-03	R = 0.149; *P* = 5.30E-03	R = 0.1846; *P* = 1.20E-03

SET=5%	Digestive system	122	sediment	60	R = 0.5642; *P* = 1.00E-04	R = 0.5406; *P* = 1.00E-04	R = 0.2826; *P* = 1.00E-04	R = 0.296; *P* = 1.00E-04	R = 0.1645; *P* = 1.00E-04	R = 0.1644; *P* = 1.00E-04
SET=5%	Digestive system	122	skin	15	R = 0.7501; *P* = 1.00E-04	R = 0.7377; *P* = 1.00E-04	R = 0.5266; *P* = 1.00E-04	R = 0.5659; *P* = 1.00E-04	R = 0.3874; *P* = 1.00E-04	R = 0.4137; *P* = 1.00E-04
SET=5%	Digestive system	122	Soil	85	R = 0.8522; *P* = 1.00E-04	R = 0.8498; *P* = 1.00E-04	R = 0.7088; *P* = 1.00E-04	R = 0.7293; *P* = 1.00E-04	R = 0.6162; *P* = 1.00E-04	R = 0.602; *P* = 1.00E-04
SET=5%	Digestive system	122	wastewater	45	R = 0.4933; *P* = 1.00E-04	R = 0.4917; *P* = 1.00E-04	R = 0.2529; *P* = 1.00E-04	R = 0.2751; *P* = 1.00E-04	R = 0.1481; *P* = 1.10E-03	R = 0.1683; *P* = 1.00E-04
SET=5%	Digestive system	122	water	68	R = 0.3728; *P* = 1.00E-04	R = 0.3473; *P* = 1.00E-04	R = 0.376; *P* = 1.00E-04	R = 0.3804; *P* = 1.00E-04	R = 0.3197; *P* = 1.00E-04	R = 0.346; *P* = 1.00E-04
SET=5%	Oral	27	sediment	60	R = 0.352; *P* = 1.00E-04	R = 0.3266; *P* = 1.00E-04	R = 0.3558; *P* = 1.00E-04	R = 0.357; *P* = 1.00E-04	R = 0.3353; *P* = 1.00E-04	R = 0.3449; *P* = 1.00E-04
SET=5%	Oral	27	skin	15	R = 0.472; *P* = 1.00E-04	R = 0.4657; *P* = 1.00E-04	R = 0.3476; *P* = 1.00E-04	R = 0.3822; *P* = 1.00E-04	R = 0.2683; *P* = 6.00E-04	R = 0.2924; *P* = 4.00E-04
SET=5%	Oral	27	Soil	85	R = 0.734; *P* = 1.00E-04	R = 0.727; *P* = 1.00E-04	R = 0.7379; *P* = 1.00E-04	R = 0.7366; *P* = 1.00E-04	R = 0.7; *P* = 1.00E-04	R = 0.6899; *P* = 1.00E-04
SET=5%	Oral	27	wastewater	45	R = 0.1565; *P* = 1.20E-03	R = 0.1448; *P* = 2.40E-03	R = 0.1735; *P* = 1.00E-04	R = 0.1863; *P* = 6.00E-04	R = 0.1751; *P* = 4.00E-04	R = 0.1865; *P* = 3.00E-04

SET=5%	Oral	27	water	68	R = -0.01154; *P* = 5.67E-01	R = -0.03037; *P* = 7.36E-01	R = 0.03484; *P* = 1.93E-01	R = 0.0214; *P* = 2.81E-01	R = 0.0282; *P* = 2.21E-01	R = 0.03899; *P* = 1.64E-01
SET=5%	Sediment	60	skin	15	R = 0.4051; *P* = 2.00E-04	R = 0.3931; *P* = 1.00E-04	R = 0.4114; *P* = 1.00E-04	R = 0.4427; *P* = 1.00E-04	R = 0.3515; *P* = 2.00E-04	R = 0.3837; *P* = 1.00E-04
SET=5%	Sediment	60	Soil	85	R = 0.2821; *P* = 1.00E-04	R = 0.3006; *P* = 1.00E-04	R = 0.3529; *P* = 1.00E-04	R = 0.3505; *P* = 1.00E-04	R = 0.3418; *P* = 1.00E-04	R = 0.3314; *P* = 1.00E-04
SET=5%	Sediment	60	wastewater	45	R = 0.09205; *P* = 7.00E-04	R = 0.1035; *P* = 4.00E-04	R = 0.08982; *P* = 8.00E-04	R = 0.09872; *P* = 5.00E-04	R = 0.07793; *P* = 2.90E-03	R = 0.08212; *P* = 2.10E-03
SET=5%	Sediment	60	water	68	R = 0.2088; *P* = 1.00E-04	R = 0.2041; *P* = 1.00E-04	R = 0.358; *P* = 1.00E-04	R = 0.3452; *P* = 1.00E-04	R = 0.3616; *P* = 1.00E-04	R = 0.3632; *P* = 1.00E-04
SET=5%	Skin	15	Soil	85	R = 0.4598; *P* = 1.00E-04	R = 0.4348; *P* = 1.00E-04	R = 0.5416; *P* = 1.00E-04	R = 0.5475; *P* = 1.00E-04	R = 0.4908; *P* = 1.00E-04	R = 0.4923; *P* = 1.00E-04
SET=5%	Skin	15	wastewater	45	R = 0.2695; *P* = 1.50E-03	R = 0.2497; *P* = 2.00E-03	R = 0.27; *P* = 1.50E-03	R = 0.3052; *P* = 3.00E-04	R = 0.25; *P* = 2.30E-03	R = 0.288; *P* = 1.40E-03
SET=5%	Skin	15	water	68	R = 0.3363; *P* = 2.00E-04	R = 0.3234; *P* = 1.00E-04	R = 0.3508; *P* = 1.00E-04	R = 0.3683; *P* = 1.00E-04	R = 0.3186; *P* = 1.00E-04	R = 0.3413; *P* = 1.00E-04

SET=5%	Soil	85	wastewater	45	R = 0.3613; P = 1.00E-04	R = 0.371; P = 1.00E-04	R = 0.437; P = 1.00E-04	R = 0.4435; P = 1.00E-04	R = 0.4155; P = 1.00E-04	R = 0.4122; P = 1.00E-04
SET=5%	Soil	85	water	68	R = 0.559; P = 1.00E-04	R = 0.5658; P = 1.00E-04	R = 0.6867; P = 1.00E-04	R = 0.6778; P = 1.00E-04	R = 0.6834; P = 1.00E-04	R = 0.6815; P = 1.00E-04
SET=5%	Wastewater	45	water	68	R = 0.1144; P = 6.00E-04	R = 0.1265; P = 2.00E-04	R = 0.2429; P = 1.00E-04	R = 0.2426; P = 1.00E-04	R = 0.2508; P = 1.00E-04	R = 0.2565; P = 1.00E-04
SET=10%	Digestive system	122	Oral	27	R = 0.2269; P = 1.10E-03	R = 0.2055; P = 2.30E-03	R = 0.1855; P = 2.20E-03	R = 0.1897; P = 2.30E-03	R = 0.1515; P = 5.10E-03	R = 0.1846; P = 1.40E-03
SET=10%	Digestive system	122	sediment	60	R = 0.5691; P = 1.00E-04	R = 0.5427; P = 1.00E-04	R = 0.2916; P = 1.00E-04	R = 0.3039; P = 1.00E-04	R = 0.1731; P = 1.00E-04	R = 0.1728; P = 1.00E-04
SET=10%	Digestive system	122	skin	15	R = 0.7574; P = 1.00E-04	R = 0.7432; P = 1.00E-04	R = 0.5416; P = 1.00E-04	R = 0.581; P = 1.00E-04	R = 0.4031; P = 1.00E-04	R = 0.428; P = 1.00E-04
SET=10%	Digestive system	122	Soil	85	R = 0.8603; P = 1.00E-04	R = 0.8577; P = 1.00E-04	R = 0.7194; P = 1.00E-04	R = 0.7398; P = 1.00E-04	R = 0.6282; P = 1.00E-04	R = 0.6136; P = 1.00E-04
SET=10%	Digestive system	122	wastewater	45	R = 0.4986; P = 1.00E-04	R = 0.4955; P = 1.00E-04	R = 0.2592; P = 1.00E-04	R = 0.2809; P = 1.00E-04	R = 0.1558; P = 6.00E-04	R = 0.1767; P = 2.00E-04
SET=10%	Digestive system	122	water	68	R = 0.3767; P = 1.00E-04	R = 0.3489; P = 1.00E-04	R = 0.3854; P = 1.00E-04	R = 0.3892; P = 1.00E-04	R = 0.3303; P = 1.00E-04	R = 0.3567; P = 1.00E-04

SET=10%	Oral	27	sediment	60	R = 0.3433; *P* = 1.00E-04	R = 0.3146; *P* = 1.00E-04	R = 0.3554; *P* = 1.00E-04	R = 0.3554; *P* = 1.00E-04	R = 0.3399; *P* = 1.00E-04	R = 0.3489; *P* = 1.00E-04
SET=10%	Oral	27	skin	15	R = 0.4829; *P* = 1.00E-04	R = 0.4735; *P* = 1.00E-04	R = 0.3485; *P* = 1.00E-04	R = 0.3812; *P* = 1.00E-04	R = 0.2681; *P* = 8.00E-04	R = 0.2931; *P* = 2.00E-04
SET=10%	Oral	27	Soil	85	R = 0.7334; *P* = 1.00E-04	R = 0.7279; *P* = 1.00E-04	R = 0.7303; *P* = 1.00E-04	R = 0.7284; *P* = 1.00E-04	R = 0.693; *P* = 1.00E-04	R = 0.6829; *P* = 1.00E-04
SET=10%	Oral	27	wastewater	45	R = 0.1498; *P* = 1.40E-03	R = 0.1391; *P* = 2.20E-03	R = 0.1731; *P* = 3.00E-04	R = 0.1852; *P* = 3.00E-04	R = 0.1752; *P* = 4.00E-04	R = 0.1858; *P* = 6.00E-04
SET=10%	Oral	27	water	68	R = -0.0162; *P* = 6.17E-01	R = -0.03378; *P* = 7.73E-01	R = 0.03355; *P* = 1.92E-01	R = 0.02032; *P* = 2.83E-01	R = 0.02802; *P* = 2.27E-01	R = 0.03771; *P* = 1.67E-01
SET=10%	Sediment	60	skin	15	R = 0.4062; *P* = 3.00E-04	R = 0.3935; *P* = 1.00E-04	R = 0.4224; *P* = 1.00E-04	R = 0.4503; *P* = 1.00E-04	R = 0.3619; *P* = 3.00E-04	R = 0.3971; *P* = 1.00E-04
SET=10%	Sediment	60	Soil	85	R = 0.2826; *P* = 1.00E-04	R = 0.3018; *P* = 1.00E-04	R = 0.3548; *P* = 1.00E-04	R = 0.3516; *P* = 1.00E-04	R = 0.3449; *P* = 1.00E-04	R = 0.3343; *P* = 1.00E-04
SET=10%	Sediment	60	wastewater	45	R = 0.09535; *P* = 1.00E-03	R = 0.1059; *P* = 1.00E-04	R = 0.0997; *P* = 6.00E-04	R = 0.1088; *P* = 2.00E-04	R = 0.08855; *P* = 1.20E-03	R = 0.09377; *P* = 9.00E-04

SET=10%	Sediment	60	water	68	R = 0.2082; *P* = 1.00E-04	R = 0.2046; *P* = 1.00E-04	R = 0.3645; *P* = 1.00E-04	R = 0.3514; *P* = 1.00E-04	R = 0.3693; *P* = 1.00E-04	R = 0.3717; *P* = 1.00E-04
SET=10%	Skin	15	Soil	85	R = 0.4558; *P* = 1.00E-04	R = 0.4314; *P* = 1.00E-04	R = 0.5311; *P* = 1.00E-04	R = 0.5367; *P* = 1.00E-04	R = 0.4783; *P* = 1.00E-04	R = 0.4808; *P* = 1.00E-04
SET=10%	Skin	15	wastewater	45	R = 0.2736; *P* = 1.30E-03	R = 0.253; *P* = 1.60E-03	R = 0.2817; *P* = 1.00E-03	R = 0.3151; *P* = 5.00E-04	R = 0.2557; *P* = 1.20E-03	R = 0.2955; *P* = 7.00E-04
SET=10%	Skin	15	water	68	R = 0.3389; *P* = 2.00E-04	R = 0.3284; *P* = 1.00E-04	R = 0.3563; *P* = 1.00E-04	R = 0.3747; *P* = 1.00E-04	R = 0.3227; *P* = 1.00E-04	R = 0.3453; *P* = 1.00E-04
SET=10%	Soil	85	wastewater	45	R = 0.3705; *P* = 1.00E-04	R = 0.3804; *P* = 1.00E-04	R = 0.4441; *P* = 1.00E-04	R = 0.4494; *P* = 1.00E-04	R = 0.423; *P* = 1.00E-04	R = 0.4195; *P* = 1.00E-04
SET=10%	Soil	85	water	68	R = 0.5637; *P* = 1.00E-04	R = 0.5718; *P* = 1.00E-04	R = 0.6915; *P* = 1.00E-04	R = 0.6826; *P* = 1.00E-04	R = 0.6884; *P* = 1.00E-04	R = 0.6864; *P* = 1.00E-04
SET=10%	Wastewater	45	water	68	R = 0.1135; *P* = 6.00E-04	R = 0.126; *P* = 2.00E-04	R = 0.2451; *P* = 1.00E-04	R = 0.2453; *P* = 1.00E-04	R = 0.2532; *P* = 1.00E-04	R = 0.2591; *P* = 1.00E-04

Supplementary Table 7. ANOSIM results for codon/amino acid usage distances considering samples sequenced by the Illumina method.

Here we compared codon and amino acid usage distances among the metagenomic samples from seven ecological niches using ANOSIM test. All

the samples used in this analysis were sequenced by Illumina method. Codon usage frequencies were calculated by two approaches absCUFs and synCUFs method (see main text). ANOSIM test was carried out considering the codon or amino acid frequencies of samples from two habitats at a time using Euclidean distance matrix and Bray-Curtis dissimilarity measure separately with 10,000 permutation values. Therefore, there are two columns for the test results (one for Euclidean distance another for Bray-Curtis dissimilarity) for codon (absCUFs or synCUFs) and amino acid usage frequencies. Here N1 and N2 represent the number of samples in the first and second habitat, respectively. ANOSIM test statistics are shown with R-values and significance levels are shown with P-values.

**Sample 1**	**Sample _count1**	**Sample 2**	**Sample _count2**	**Anosim R values for absCUFs frequencies based on Eucledean distance**	**Anosim R values for synCUFs frequencies based on Eucledean distance**	**Anosim R values for amino acid frequencies based on Eucledean distance**	**Anosim R values for absCUFs frequencies based on Bray-Curtis dissimilarity**	**Anosim R values for synCUFs frequencies based on Bray-Curtis dissimilarity**	**Anosim R values for amino acid frequencies based on Bray-Curtis dissimilarity**
Digestive system	110	Oral	26	R = 0.1786; *P* = 3.80E-03	R = 0.1777; *P* = 2.60E-03	R = 0.1613; *P* = 1.31E-02	R = 0.1752; *P* = 2.70E-03	R = 0.1494; *P* = 7.70E-03	R = 0.172; *P* = 9.10E-03
Digestive system	110	sediment	60	R = 0.3444; *P* = 1.00E-04	R = 0.2388; *P* = 1.00E-04	R = 0.5289; *P* = 1.00E-04	R = 0.3364; *P* = 1.00E-04	R = 0.2382; *P* = 1.00E-04	R = 0.5609; *P* = 1.00E-04

Digestive system	110	skin	15	R = 0.6042; *P* = 1.00E-04	R = 0.4522; *P* = 1.00E-04	R = 0.7468; *P* = 1.00E-04	R = 0.569; *P* = 1.00E-04	R = 0.4293; *P* = 1.00E-04	R = 0.7691; *P* = 1.00E-04
Digestive system	110	Soil	72	R = 0.7914; *P* = 1.00E-04	R = 0.6825; *P* = 1.00E-04	R = 0.8714; *P* = 1.00E-04	R = 0.7716; *P* = 1.00E-04	R = 0.697; *P* = 1.00E-04	R = 0.8714; *P* = 1.00E-04
Digestive system	110	wastewater	45	R = 0.3053; *P* = 1.00E-04	R = 0.2144; *P* = 1.00E-04	R = 0.4899; *P* = 1.00E-04	R = 0.2871; *P* = 1.00E-04	R = 0.1945; *P* = 2.00E-04	R = 0.4936; *P* = 1.00E-04
Digestive system	110	water	55	R = 0.4443; *P* = 1.00E-04	R = 0.4108; *P* = 1.00E-04	R = 0.3577; *P* = 1.00E-04	R = 0.4401; *P* = 1.00E-04	R = 0.3923; *P* = 1.00E-04	R = 0.389; *P* = 1.00E-04
Oral	26	sediment	60	R = 0.3342; *P* = 1.00E-04	R = 0.3333; *P* = 1.00E-04	R = 0.3153; *P* = 1.00E-04	R = 0.3339; *P* = 1.00E-04	R = 0.3251; *P* = 1.00E-04	R = 0.3533; *P* = 1.00E-04
Oral	26	skin	15	R = 0.4039; *P* = 1.00E-04	R = 0.3075; *P* = 1.00E-04	R = 0.5381; *P* = 1.00E-04	R = 0.3667; *P* = 1.00E-04	R = 0.2753; *P* = 2.00E-04	R = 0.5542; *P* = 1.00E-04
Oral	26	Soil	72	R = 0.7558; *P* = 1.00E-04	R = 0.6973; *P* = 1.00E-04	R = 0.7851; *P* = 1.00E-04	R = 0.7566; *P* = 1.00E-04	R = 0.7134; *P* = 1.00E-04	R = 0.7826; *P* = 1.00E-04
Oral	26	wastewater	45	R = 0.2557; *P* = 1.00E-04	R = 0.2541; *P* = 1.00E-04	R = 0.2084; *P* = 1.00E-04	R = 0.2444; *P* = 3.00E-04	R = 0.2466; *P* = 1.00E-04	R = 0.2193; *P* = 3.00E-04
Oral	26	water	55	R = 0.0586; *P* = 6.39E-02	R = 0.0713; *P* = 3.58E-02	R = 0.0075; *P* = 3.87E-01	R = 0.0692; *P* = 3.55E-02	R = 0.0591; *P* = 5.32E-02	R = 0.0215; *P* = 2.68E-01

sediment	60	skin	15	R = 0.3929; *P* = 2.00E-04	R = 0.3367; *P* = 2.00E-04	R = 0.3653; *P* = 1.00E-04	R = 0.3615; *P* = 3.00E-04	R = 0.2888; *P* = 5.00E-04	R = 0.3825; *P* = 1.00E-04
sediment	60	Soil	72	R = 0.3277; *P* = 1.00E-04	R = 0.3052; *P* = 1.00E-04	R = 0.2955; *P* = 1.00E-04	R = 0.3274; *P* = 1.00E-04	R = 0.3156; *P* = 1.00E-04	R = 0.269; *P* = 1.00E-04
sediment	60	wastewa ter	45	R = 0.0827; *P* = 1.20E-03	R = 0.0717; *P* = 4.80E-03	R = 0.0826; *P* = 1.50E-03	R = 0.0738; *P* = 3.10E-03	R = 0.0624; *P* = 6.90E-03	R = 0.0788; *P* = 1.70E-03
sediment	60	water	55	R = 0.4049; *P* = 1.00E-04	R = 0.4201; *P* = 1.00E-04	R = 0.2965; *P* = 1.00E-04	R = 0.409; *P* = 1.00E-04	R = 0.4129; *P* = 1.00E-04	R = 0.2944; *P* = 1.00E-04
skin	15	Soil	72	R = 0.5101; *P* = 1.00E-04	R = 0.4363; *P* = 1.00E-04	R = 0.416; *P* = 1.00E-04	R = 0.5064; *P* = 1.00E-04	R = 0.4362; *P* = 1.00E-04	R = 0.4332; *P* = 1.00E-04
skin	15	wastewa ter	45	R = 0.3341; *P* = 1.00E-04	R = 0.3117; *P* = 4.00E-04	R = 0.2792; *P* = 1.10E-03	R = 0.3093; *P* = 3.00E-04	R = 0.2612; *P* = 1.20E-03	R = 0.3034; *P* = 8.00E-04
skin	15	water	55	R = 0.3749; *P* = 1.00E-04	R = 0.3314; *P* = 1.00E-04	R = 0.3626; *P* = 1.00E-04	R = 0.346; *P* = 1.00E-04	R = 0.3057; *P* = 1.00E-04	R = 0.3659; *P* = 1.00E-04
Soil	72	wastewa ter	45	R = 0.4462; *P* = 1.00E-04	R = 0.4185; *P* = 1.00E-04	R = 0.3727; *P* = 1.00E-04	R = 0.4479; *P* = 1.00E-04	R = 0.428; *P* = 1.00E-04	R = 0.3614; *P* = 1.00E-04
Soil	72	water	55	R = 0.6964; *P* = 1.00E-04	R = 0.6929; *P* = 1.00E-04	R = 0.633; *P* = 1.00E-04	R = 0.701; *P* = 1.00E-04	R = 0.6949; *P* = 1.00E-04	R = 0.617; *P* = 1.00E-04

wastewate r	45	water	55	R = 0.3269; P = 1.00E-04	R = 0.3385; P = 1.00E-04	R = 0.2093; P = 1.00E-04	R = 0.3235; P = 1.00E-04	R = 0.331; P = 1.00E-04	R = 0.189; P = 1.00E-04

Supplementary Table 8. ANOSIM results for codon/amino acid usage distances considering samples sequenced by the non-Illumina method. Here we compared codon and amino acid usage distances among the test samples from three ecological niches using ANOSIM test. All the samples used in this analysis were sequenced by non-Illumina method. Codon usage frequencies were calculated by two approaches absCUFs and synCUFs method (see main text). ANOSIM test was carried out considering the codon or amino acid frequencies of samples from two habitats at a time using Euclidean distance matrix and Bray-Curtis dissimilarity measure separately with 10,000 permutation values. Therefore, there are two columns for the test results (one for Euclidean distance another for Bray-Curtis dissimilarity) for codon (absCUFs or synCUFs) and amino acid usage frequencies. Here N1 and N2 represent the number of samples in the first and second habitat, respectively. ANOSIM test statistics are shown with R-values and significance levels are shown with P-values.

**Sample 1**	**Sample _count 1**	**Sample 2**	**Sample _count 2**	**Anosim R values for absCUFs frequencies based on Euclidean distance**	**Anosim R values for synCUFs frequencies based on Euclidean distance**	**Anosim R values for amino acid frequencies based on Euclidean distance**	**Anosim R values for absCUFs frequencies based on Bray-Curtis dissimilarity**	**Anosim R values for synCUFs frequencies based on Bray-Curtis dissimilarity**	**Anosim R values for amino acid frequencies based on Bray-Curtis dissimilarity**

Digestive system	7	Soil	11	R = 0.9686; P = 1.00E-04	R = 0.8411; P = 1.00E-04	R = 1; P = 2.00E-04	R = 0.96; P = 1.00E-04	R = 0.8281; P = 1.00E-04	R = 1; P = 1.00E-04
Digestive system	7	water	13	R = 0.6925; P = 1.00E-04	R = 0.7693; P = 1.00E-04	R = 0.2896; P = 1.10E-02	R = 0.6865; P = 1.00E-04	R = 0.701; P = 1.00E-04	R = 0.3238; P = 6.60E-03
Soil	11	water	13	R = 0.9237; P = 1.00E-04	R = 0.9412; P = 1.00E-04	R = 0.6906; P = 1.00E-04	R = 0.926; P = 1.00E-04	R = 0.9356; P = 1.00E-04	R = 0.6852; P = 1.00E-04

Supplementary Table 9. ANOSIM results for codon usage distances of probable ribosomal protein coding genes in the test metagenomic samples. Here we compared codon usage distances (absCUFs and synCUFs frequencies) of probable ribosomal protein coding genes in the test metagenomic samples using ANOSIM test. ANOSIM test was carried out considering 10,000 permutation values and two matrices separately (i) Euclidean, and (ii) Bray-Curtis dissimilarity matrices. Therefore, there are two columns (one for Euclidean distance and another for Bray-Curtis dissimilarity) for absCUFs or synCUFs. N1 and N2 represent the number of samples in the first and second habitat, respectively. ANOSIM test statistics are shown with R-values and significance levels are shown with *P*-values.

Habitat1	N1	Habitat2	N2	ANOSIM R-values for absCUFs (Euclidean distance)	ANOSIM R-values for absCUFs (Bray-Curtis dissimilarity)	ANOSIM R-values for synCUFs (Euclidean distance)	ANOSIM R-values for synCUFs (Bray-Curtis dissimilarity)
Digestive system	104	Oral	27	R = 0.3912; *P* = 0.0001	R = 0.3968; *P* = 0.0001	R = 0.4359; *P* = 0.0001	R = 0.3933; *P* = 0.0001
Digestive system	104	Sediment	53	R = 0.3736; *P* = 0.0001	R = 0.3905; *P* = 0.0001	R = 0.3061; *P* = 0.0001	R = 0.2969; *P* = 0.0001
Digestive system	104	Skin	11	R = 0.4767; *P* = 0.0002	R = 0.4874; *P* = 0.0001	R = 0.4839; *P* = 0.0001	R = 0.447; *P* = 0.0002
Digestive system	104	Soil	85	R = 0.8217; *P* = 0.0001	R = 0.8203; *P* = 0.0001	R = 0.7449; *P* = 0.0001	R = 0.7551; *P* = 0.0001

Digestive system	104	wastewater	40	R = 0.3334; P = 0.0001	R = 0.3352; P = 0.0001	R = 0.3245; P = 0.0001	R = 0.2809; P = 0.0001
Digestive system	104	Water	59	R = 0.5764; P = 0.0001	R = 0.5841; P = 0.0001	R = 0.5918; P = 0.0001	R = 0.5657; P = 0.0001
Oral	27	Sediment	53	R = 0.4242; P = 0.0001	R = 0.4267; P = 0.0001	R = 0.4208; P = 0.0001	R = 0.4096; P = 0.0001
Oral	27	Skin	11	R = 0.5123; P = 0.0001	R = 0.5016; P = 0.0001	R = 0.5222; P = 0.0001	R = 0.5103; P = 0.0001
Oral	27	Soil	85	R = 0.8631; P = 0.0001	R = 0.8702; P = 0.0001	R = 0.8401; P = 0.0001	R = 0.8522; P = 0.0001
Oral	27	wastewater	40	R = 0.2381; P = 0.0001	R = 0.2357; P = 0.0001	R = 0.2448; P = 0.0001	R = 0.2353; P = 0.0001

Oral	27	Water	59	R = 0.0952; *P* = 0.0115	R = 0.1027; *P* = 0.009	R = 0.1157; *P* = 0.0037	R = 0.0984; *P* = 0.0096
sediment	53	Skin	11	R = 0.3509; *P* = 0.0004	R = 0.3347; *P* = 0.0003	R = 0.3659; *P* = 0.0002	R = 0.3179; *P* = 0.0007
sediment	53	Soil	85	R = 0.4564; *P* = 0.0001	R = 0.4635; *P* = 0.0001	R = 0.4452; *P* = 0.0001	R = 0.4548; *P* = 0.0001
sediment	53	wastewater	40	R = 0.1332; *P* = 0.0003	R = 0.124; *P* = 0.0003	R = 0.1267; *P* = 0.0004	R = 0.1108; *P* = 0.0005
Sediment	53	Water	59	R = 0.4284; *P* = 0.0001	R = 0.4449; *P* = 0.0001	R = 0.4522; *P* = 0.0001	R = 0.4478; *P* = 0.0001
Skin	11	Soil	85	R = 0.6354; *P* = 0.0001	R = 0.645; *P* = 0.0001	R = 0.5597; *P* = 0.0001	R = 0.569; *P* = 0.0001

Skin	11	wastewater	40	R = 0.172; *P* = 0.0234	R = 0.151; *P* = 0.0372	R = 0.2008; *P* = 0.0134	R = 0.1626; *P* = 0.0298
Skin	11	Water	59	R = 0.3407; *P* = 0.0001	R = 0.357; *P* = 0.0001	R = 0.3918; *P* = 0.0001	R = 0.3714; *P* = 0.0001
Soil	85	wastewater	40	R = 0.6045; *P* = 0.0001	R = 0.614; *P* = 0.0001	R = 0.5842; *P* = 0.0001	R = 0.5987; *P* = 0.0001
Soil	85	Water	59	R = 0.807; *P* = 0.0001	R = 0.8135; *P* = 0.0001	R = 0.805; *P* = 0.0001	R = 0.8073; *P* = 0.0001
Wastewater	40	Water	59	R = 0.2938; *P* = 0.0001	R = 0.3115; *P* = 0.0001	R = 0.3246; *P* = 0.0001	R = 0.3198; *P* = 0.0001

Supplementary Table 10. Correlations between distances in average GC content with distances in codon usage frequencies. For each sample, codon usage frequencies were calculated by two approaches absCUFs and synCUFs method (see main text). Distances in codon usage

frequencies (absCUFs or synCUFs) among the samples were calculated by the Euclidean distance and Bray-Curtis dissimilarity method. Distances between GC content among the samples were calculated as absolute distances in their average GC content. Correlation values were calculated considering distances (in codon usage frequencies and GC content) among all samples in our dataset and also considering distances among the samples from each seven selected habitats separately. To access the strength of correlation, we calculated Spearman Rank correlation co-efficient (ρ) where significance levels were shown with *P*-values.

**Sample's biome**	**Number of samples**	**Correlation between distances in GC content and absCUFs distances (absCUFs distances calculated by Euclidean distance)**	**Correlation between distances in GC content and absCUFs distances (absCUFs distances calculated by Bray-Curtis dissimilarity)**	**Correlation between distances in GC content and synCUFs distances (synCUFs distances calculated by Euclidean distance)**	**Correlation between distances in GC content and synCUFs distances (synCUFs distances calculated by Bray-Curtis dissimilarity)**
All samples	422	ρ = 0.904; *P* = 0	ρ = 0.912; *P* = 0	ρ = 0.900; *P* = 0	ρ = 0.918; *P* = 0
Digestive system	122	ρ = 0.828; *P* = 0	ρ = 0.8315; *P* = 0	ρ = 0.812; *P* = 0	ρ = 0.8415; *P* = 0

Oral	26	ρ = 0.963; P = 1.05E-200	ρ = 0.9619; P = 1.049764E-198	ρ = 0.953; P = 2.88E-183	ρ = 0.9603; P = 1.672031E-195
Sediment	56	ρ = 0.774; P = 0	ρ = 0.7874; P = 0	ρ = 0.812; P = 0	ρ = 0.8389; P = 0
Skin	15	ρ = 0.695; P = 1.85E-16	ρ = 0.8295; P = 8.382611E-28	ρ = 0.923; P = 1.84E-44	ρ = 0.9369; P = 8.215296E-49
Soil	78	ρ = 0.967; P = 0	ρ = 0.9711; P = 0	ρ = 0.948; P = 0	ρ = 0.9592; P = 0
Waste water	43	ρ = 0.851; P = 3.35E-279	ρ = 0.8515; P = 2.748464E-279	ρ = 0.885; P = 0	ρ = 0.9046; P = 0
Water	67	ρ = 0.975; P = 0	ρ = 0.9737; P = 0	ρ = 0.974; P = 0	ρ = 0.977; P = 0

Supplementary Table 11. Correlations between distances in codon/amino acid usage frequencies with k-mer usage frequencies. For each sample, codon usage frequencies were calculated by two approaches absCUFs and synCUFs method (see main text) and k-mer frequencies were calculated for k=2 to k=10 (except for k=3). All the distances in the usage frequencies (codon, amino acid or k-mers) among the samples were

calculated by the Euclidean distance and Bray-Curtis dissimilarity methods and correlated separately. To access the strength of correlation, we calculated Spearman Rank correlation co-efficient (ρ) where significance levels were shown with *P*-values.

k-mers	Correlation between distances in k-mer frequencies and amino acid distances (distances calculated by Euclidean distance method)	Correlation between distances in k-mer frequencies and amino acid distances (distances calculated by Bray-Curtis dissimilarity method)	Correlation between distances in k-mer frequencies and absCUFs distances (distances calculated by Euclidean distance method)	Correlation between distances in k-mer frequencies and absCUFs distances (distances calculated by Bray-Curtis dissimilarity method)	Correlation between distances in k-mer frequencies and synCUFs distances (distances calculated by Euclidean distance method)	Correlation between distances in k-mer frequencies and synCUFs distances (distances calculated by Bray-Curtis dissimilarity method)
k2 frequencies	ρ = 0.4828; *P* = 1 < 10-6	ρ = 0.6939; *P* = 1 < 10-6	ρ = 0.5439; *P* = 1 < 10-6	ρ = 0.7863; *P* = 1 < 10-6	ρ = 0.5392; *P* = 1 < 10-6	ρ = 0.7757; *P* = 1 < 10-6
k4 frequencies	ρ = 0.9033; *P* = 1 < 10-6	ρ = 0.9008; *P* = 1 < 10-6	ρ = 0.9611; *P* = 1 < 10-6	ρ = 0.9759; *P* = 1 < 10-6	ρ = 0.9175; *P* = 1 < 10-6	ρ = 0.9437; *P* = 1 < 10-6
k5 frequencies	ρ = 0.9221; *P* = 1 < 10-6	ρ = 0.9102; *P* = 1 < 10-6	ρ = 0.9627; *P* = 1 < 10-6	ρ = 0.9705; *P* = 1 < 10-6	ρ = 0.9101; *P* = 1 < 10-6	ρ = 0.9315; *P* = 1 < 10-6
selected k10 frequencies	ρ = 0.6329; *P* = 1 < 10-6	ρ = 0.7725; *P* = 1 < 10-6	ρ = 0.6757; *P* = 1 < 10-6	ρ = 0.7789; *P* = 1 < 10-6	ρ = 0.6432; *P* = 1 < 10-6	ρ = 0.7202; *P* = 1 < 10-6
selected k6 frequencies	ρ = 0.9156; *P* = 1 < 10-6	ρ = 0.9074; *P* = 1 < 10-6	ρ = 0.9445; *P* = 1 < 10-6	ρ = 0.9651; *P* = 1 < 10-6	ρ = 0.8866; *P* = 1 < 10-6	ρ = 0.9225; *P* = 1 < 10-6

selected k7 frequencies	ρ = 0.896; *P* = 1 < 10-6	ρ = 0.9123; *P* = 1 < 10-6	ρ = 0.9061; *P* = 1 < 10-6	ρ = 0.9453; *P* = 1 < 10-6	ρ = 0.8438; *P* = 1 < 10-6	ρ = 0.8939; *P* = 1 < 10-6
selected k8 frequencies	ρ = 0.852; *P* = 1 < 10-6	ρ = 0.8979; *P* = 1 < 10-6	ρ = 0.8585; *P* = 1 < 10-6	ρ = 0.9219; *P* = 1 < 10-6	ρ = 0.7996; *P* = 1 < 10-6	ρ = 0.8655; *P* = 1 < 10-6
selected k9 frequencies	ρ = 0.6798; *P* = 1 < 10-6	ρ = 0.8274; *P* = 1 < 10-6	ρ = 0.7222; *P* = 1 < 10-6	ρ = 0.8493; *P* = 1 < 10-6	ρ = 0.6865; *P* = 1 < 10-6	ρ = 0.7987; *P* = 1 < 10-6

Supplementary Table 12. Correlations between distances in codon usage frequencies and that of GO terms' abundance. Correlation values were calculated considering distances (in codon usage frequencies or GO terms' abundance) among all samples in our dataset and also considering distances among the samples from each of seven selected habitats separately. Codon usage distances were calculated based on codon usage frequencies calculated by two approaches absCUFs and synCUFs method (see main text). Distances in Gene Ontology (GO) terms' frequencies among the samples were calculated by considering the relative abundance of 500 GO biological process, 500 GO molecular function, and 100 GO cellular component terms separately (see main text). All the distances (either in codon usage or GO terms' abundance) among the samples were calculated by (i) Euclidean distance and (ii) Bray-Curtis dissimilarity methods. Correlation values were calculated considering the distances calculated by these two methods separately. To access the strength of correlation, we calculated Spearman's rank correlation co-efficient (ρ) where significance levels were shown with *P*-values.

GO TYPE	Habitat	Number of samples	Spearman's correlation (ρ) with distances in absCUFs (distances calculated by Euclidean method)	Spearman's correlation (ρ) with distances in absCUFs (distances calculated by Bray-Curtis method)	Spearman's correlation (ρ) with distances in synCUFs (distances calculated by Euclidean method)	Spearman's correlation (ρ) with distances in synCUFs distances calculated by (Bray-Curtis method)
BP	Digestive system	122	ρ = 0.672; *P* = 0	ρ = 0.6916*;* *P* = 0.000E+0	ρ = 0.639; *P* = 0	ρ = 0.6322*;* *P* = 0.000E+0
BP	Oral	26	ρ = 0.726; *P* = 1.13E-58	ρ = 0.6992*;* *P* = 8.286E-53	ρ = 0.724; *P* = 3.29E-58	ρ = 0.6856*;* *P* = 4.738E-50
BP	Sediment	56	ρ = 0.470; *P* = 4.48E-98	ρ = 0.4191*;* *P* = 3.243E-76	ρ = 0.403; *P* = 2.99E-70	ρ = 0.3343*;* *P* = 1.801E-47
BP	Skin	15	ρ = 0.462; *P* = 6.95E-07	ρ = 0.4862*;* *P* = 1.460E-7	ρ = 0.583; *P* = 6.56E-11	ρ = 0.5704*;* *P* = 2.117E-10
BP	Soil	78	ρ = 0.487; *P* = 0	ρ = 0.5582*;* *P* = 1.203E-291	ρ = 0.481; *P* = 0	ρ = 0.542*;* *P* = 9.018E-272
BP	Wastewater	43	ρ = 0.558; *P* = 3.88E-82	ρ = 0.554*;* *P* = 9.961E-81	ρ = 0.461; *P* = 3.72E-53	ρ = 0.4371*;* *P* = 1.881E-47
BP	Water	67	ρ = 0.390; *P* = 1.29E-83	ρ = 0.4798*;* *P* = 1.708E-131	ρ = 0.373; *P* = 3.13E-76	ρ = 0.4458*;* *P* = 1.248E-111
CC	Digestive system	122	ρ = 0.464; *P* = 0	ρ = 0.5776*;* *P* = 0.000E+0	ρ = 0.449; *P* = 0	ρ = 0.5307*;* *P* = 0.000E+0

CC	Oral	26	ρ = 0.528; P = 1.30E-26	ρ = 0.5536; P = 1.424E-29	ρ = 0.534; P = 3.26E-27	ρ = 0.5461; P = 1.140E-28
CC	Sediment	56	ρ = 0.370; P = 1.17E-58	ρ = 0.2762; P = 3.268E-2	ρ = 0.309; P = 1.96E-40	-----------
CC	Skin	15	ρ = 0.232; P = 0.01707	ρ = 0.336; P = 4.575E-4	ρ = 0.326; P = 0.0007	ρ = 0.4105; P = 1.368E-5
CC	Soil	78	ρ = 0.345; P = 0	ρ = 0.4472; P = 3.645E-175	ρ = 0.320; P = 1.39E-85	ρ = 0.4277; P = 8.836E-159
CC	Wastewater	43	ρ = 0.305; P = 8.17E-23	ρ = 0.3538; P = 1.479E-30	ρ = 0.238; P = 2.90E-14	ρ = 0.2478; P = 2.544E-15
CC	Water	67	ρ = 0.191; P = 4.18E-20	ρ = 0.2592; P = 2.724E-36	ρ = 0.172; P = 1.44E-16	ρ = 0.2268; P = 5.755E-28
MF	Digestive system	122	ρ = 0.675; P = 0	ρ = 0.6987; P = 0.000E+0	ρ = 0.651; P = 0	ρ = 0.6385; P = 0.000E+0
MF	Oral	26	ρ = 0.451; P = 5.78E-19	ρ = 0.6149; P = 6.961E-38	ρ = 0.449; P = 7.61E-19	ρ = 0.5968; P = 3.039E-35
MF	Sediment	56	ρ = 0.393; P = 1.88E-66	ρ = 0.3937; P = 1.086E-66	ρ = 0.319; P = 2.89E-43	ρ = 0.3046; P = 2.520E-39
MF	Skin	15	ρ = 0.421; P = 7.93E-06	ρ = 0.5054; P = 3.825E-8	ρ = 0.532; P = 5.38E-09	ρ = 0.5698; P = 2.245E-10
MF	Soil	78	ρ = 0.361; P = 0	ρ = 0.5106; P = 2.533E-236	ρ = 0.339; P = 8.47E-97	ρ = 0.484; P = 4.575E-209
MF	Wastewater	43	ρ = 0.564; P = 3.44E-84	ρ = 0.5746; P = 4.659E-88	ρ = 0.484; P = 3.68E-59	ρ = 0.4606; P = 3.893E-53

MF	Water	67	ρ = 0.299; *P* = 2.04E-48	ρ = 0.4227*;* *P* = 1.998E-99	ρ = 0.281; *P* = 1.20E-42	ρ = 0.388*;* *P* = 9.429E-83

Supplementary Table 13. ANOSIM results for different COG functional categories. Here we compared codon usage distances (absCUFs and synCUFs) of sequences within different COG functional categories for samples from 7 environmental biomes. ANOSIM test was carried out considering 10,000 permutation values and two matrices separately (i) Euclidean, and (ii) Bray-Curtis dissimilarity matrices. Therefore, there are two columns (one for Euclidean distance and another for Bray-Curtis dissimilarity) for absCUFs or synCUFs. N1 and N2 represent the number of samples in the first and second habitat, respectively. ANOSIM test statistics are shown with R-values and significance levels are shown with *P*-values.

**Functional categories**	**Description**	**Habitat1**	**N1**	**Habitat2**	**N2**	**ANOSIM results for absCUFs frequency based on Euclidean distance**	**ANOSIM results for synCUFs frequency based on Euclidean distance**	**ANOSIM results for absCUFs frequency based on Bray-Curtis dissimilarity**	**ANOSIM resulst for synCUFs frequency based on Bray-Curtis dissimilarity**

C	Energy production and conversion	Digestive system	122	Oral	27	R = 0.1713; *P* = 0.0064	R = 0.2304; *P* = 0.0011	R = 0.1774; *P* = 0.0038	R = 0.1869; *P* = 0.004
C	Energy production and conversion	Digestive system	122	sediment	53	R = 0.2267; *P* = 0.0001	R = 0.1533; *P* = 0.0005	R = 0.2334; *P* = 0.0001	R = 0.1575; *P* = 0.0003
C	Energy production and conversion	Digestive system	122	skin	15	R = 0.3515; *P* = 0.0002	R = 0.3155; *P* = 0.0013	R = 0.3673; *P* = 0.0001	R = 0.3099; *P* = 0.0013
C	Energy production and conversion	Digestive system	122	Soil	82	R = 0.6735; *P* = 0.0001	R = 0.5736; *P* = 0.0001	R = 0.6682; *P* = 0.0001	R = 0.5954; *P* = 0.0001
C	Energy production and conversion	Digestive system	122	wastewater	45	R = 0.2481; *P* = 0.0001	R = 0.2008; *P* = 0.0001	R = 0.2494; *P* = 0.0001	R = 0.1899; *P* = 0.0004
C	Energy production and conversion	Digestive system	122	water	67	R = 0.4446; *P* = 0.0001	R = 0.4541; *P* = 0.0001	R = 0.4497; *P* = 0.0001	R = 0.4351; *P* = 0.0001

C	Energy production and conversion	Oral	27	sediment	53	R = 0.4189; *P* = 0.0001	R = 0.4238; *P* = 0.0001	R = 0.4248; *P* = 0.0001	R = 0.4139; *P* = 0.0001
C	Energy production and conversion	Oral	27	skin	15	R = 0.4087; *P* = 0.0001	R = 0.3697; *P* = 0.0001	R = 0.3916; *P* = 0.0001	R = 0.3603; *P* = 0.0001
C	Energy production and conversion	Oral	27	Soil	82	R = 0.8463; *P* = 0.0001	R = 0.8039; *P* = 0.0001	R = 0.8521; *P* = 0.0001	R = 0.8213; *P* = 0.0001
C	Energy production and conversion	Oral	27	wastewater	45	R = 0.3389; *P* = 0.0001	R = 0.2863; *P* = 0.0001	R = 0.3146; *P* = 0.0001	R = 0.279; *P* = 0.0001
C	Energy production and conversion	Oral	27	water	67	R = 0.1171; *P* = 0.004	R = 0.145; *P* = 0.0006	R = 0.119; *P* = 0.0024	R = 0.1223; *P* = 0.0029
C	Energy production and conversion	Sediment	53	skin	15	R = 0.3414; *P* = 0.0003	R = 0.355; *P* = 0.0001	R = 0.3447; *P* = 0.0002	R = 0.3234; *P* = 0.0004

C	Energy production and conversion	sediment	53	Soil	82	R = 0.4116; *P* = 0.0001	R = 0.3703; *P* = 0.0001	R = 0.4187; *P* = 0.0001	R = 0.3829; *P* = 0.0001
C	Energy production and conversion	Sediment	53	wastewater	45	R = 0.115; *P* = 0.0001	R = 0.1054; *P* = 0.0003	R = 0.107; *P* = 0.0001	R = 0.0948; *P* = 0.0004
C	Energy production and conversion	Sediment	53	water	67	R = 0.4255; *P* = 0.0001	R = 0.439; *P* = 0.0001	R = 0.4304; *P* = 0.0001	R = 0.4341; *P* = 0.0001
C	Energy production and conversion	Skin	15	Soil	82	R = 0.6378; *P* = 0.0001	R = 0.5387; *P* = 0.0001	R = 0.6369; *P* = 0.0001	R = 0.5565; *P* = 0.0001
C	Energy production and conversion	Skin	15	wastewater	45	R = 0.1856; *P* = 0.0109	R = 0.1917; *P* = 0.0084	R = 0.1714; *P* = 0.0168	R = 0.1636; *P* = 0.0195
C	Energy production and conversion	Skin	15	water	67	R = 0.3711; *P* = 0.0001	R = 0.4037; *P* = 0.0001	R = 0.3663; *P* = 0.0001	R = 0.3813; *P* = 0.0001

C	Energy production and conversion	Soil	82	wastewater	45	R = 0.4975; P = 0.0001	R = 0.4475; P = 0.0001	R = 0.4843; P = 0.0001	R = 0.4559; P = 0.0001
C	Energy production and conversion	Soil	82	water	67	R = 0.7378; P = 0.0001	R = 0.7423; P = 0.0001	R = 0.7408; P = 0.0001	R = 0.7394; P = 0.0001
C	Energy production and conversion	wastewater	45	water	67	R = 0.3759; P = 0.0001	R = 0.3762; P = 0.0001	R = 0.3718; P = 0.0001	R = 0.3714; P = 0.0001
D	Cell cycle control, cell division, chromosome partitioning	Digestive system	115	Oral	27	R = 0.2195; P = 0.0012	R = 0.2426; P = 0.0003	R = 0.2294; P = 0.0006	R = 0.2102; P = 0.0009
D	Cell cycle control, cell division, chromosome partitioning	Digestive system	115	sediment	50	R = 0.2586; P = 0.0001	R = 0.1958; P = 0.0001	R = 0.2682; P = 0.0001	R = 0.194; P = 0.0001
D	Cell cycle control,	Digestive	115	skin	13	R = 0.3869;	R = 0.3287;	R = 0.3952;	R = 0.3034;

	cell division, chromosome partitioning	system				*P* = 0.0003	*P* = 0.0005	*P* = 0.0001	*P* = 0.0021
D	Cell cycle control, cell division, chromosome partitioning	Digestive system	115	Soil	82	R = 0.7388; *P* = 0.0001	R = 0.6871; *P* = 0.0001	R = 0.7413; *P* = 0.0001	R = 0.6997; *P* = 0.0001
D	Cell cycle control, cell division, chromosome partitioning	Digestive system	115	wastewater	40	R = 0.2122; *P* = 0.0002	R = 0.1738; *P* = 0.0004	R = 0.2146; *P* = 0.0001	R = 0.1539; *P* = 0.0013
D	Cell cycle control, cell division, chromosome partitioning	Digestive system	115	water	67	R = 0.4573; *P* = 0.0001	R = 0.4629; *P* = 0.0001	R = 0.4683; *P* = 0.0001	R = 0.4396; *P* = 0.0001
D	Cell cycle control, cell division, chromosome	Oral	27	sediment	50	R = 0.4022; *P* = 0.0001	R = 0.3745; *P* = 0.0001	R = 0.3902; *P* = 0.0001	R = 0.3646; *P* = 0.0001

	partitioning								
D	Cell cycle control, cell division, chromosome partitioning	Oral	27	skin	13	R = 0.3364; $P = 0.0001$	R = 0.2707; $P = 0.0006$	R = 0.302; $P = 0.0003$	R = 0.2507; $P = 0.0019$
D	Cell cycle control, cell division, chromosome partitioning	Oral	27	Soil	82	R = 0.8407; $P = 0.0001$	R = 0.8034; $P = 0.0001$	R = 0.8427; $P = 0.0001$	R = 0.815; $P = 0.0001$
D	Cell cycle control, cell division, chromosome partitioning	Oral	27	wastewater	40	R = 0.2735; $P = 0.0001$	R = 0.2491; $P = 0.0001$	R = 0.2536; $P = 0.0001$	R = 0.2352; $P = 0.0001$
D	Cell cycle control, cell division, chromosome partitioning	Oral	27	water	67	R = 0.0525; $P = 0.0988$	R = 0.0862; $P = 0.0217$	R = 0.0619; $P = 0.0549$	R = 0.0721; $P = 0.0348$
D	Cell cycle control,	sediment	50	skin	13	R = 0.279;	R = 0.234;	R = 0.2736;	R = 0.2201;

	cell division, chromosome partitioning					*P* = 0.0014	*P* = 0.0046	*P* = 0.001	*P* = 0.0064
D	Cell cycle control, cell division, chromosome partitioning	Sediment	50	Soil	82	R = 0.3941; *P* = 0.0001	R = 0.3667; *P* = 0.0001	R = 0.4013; *P* = 0.0001	R = 0.3782; *P* = 0.0001
D	Cell cycle control, cell division, chromosome partitioning	Sediment	50	wastewater	40	R = 0.1074; *P* = 0.0011	R = 0.0886; *P* = 0.0027	R = 0.0997; *P* = 0.0011	R = 0.0856; *P* = 0.0024
D	Cell cycle control, cell division, chromosome partitioning	Sediment	50	water	67	R = 0.4062; *P* = 0.0001	R = 0.4253; *P* = 0.0001	R = 0.4113; *P* = 0.0001	R = 0.4184; *P* = 0.0001
D	Cell cycle control, cell division, chromosome	Skin	13	Soil	82	R = 0.6245; *P* = 0.0001	R = 0.5782; *P* = 0.0001	R = 0.6453; *P* = 0.0001	R = 0.5972; *P* = 0.0001

	partitioning								
D	Cell cycle control, cell division, chromosome partitioning	Skin	13	wastewater	40	R = 0.1946; *P* = 0.0127	R = 0.1954; *P* = 0.0109	R = 0.1849; *P* = 0.0143	R = 0.1619; *P* = 0.0252
D	Cell cycle control, cell division, chromosome partitioning	skin	13	water	67	R = 0.2769; *P* = 0.0001	R = 0.2994; *P* = 0.0001	R = 0.2814; *P* = 0.0002	R = 0.2828; *P* = 0.0002
D	Cell cycle control, cell division, chromosome partitioning	Soil	82	wastewater	40	R = 0.561; *P* = 0.0001	R = 0.5378; *P* = 0.0001	R = 0.5652; *P* = 0.0001	R = 0.5503; *P* = 0.0001
D	Cell cycle control, cell division, chromosome partitioning	Soil	82	water	67	R = 0.7744; *P* = 0.0001	R = 0.7798; *P* = 0.0001	R = 0.7785; *P* = 0.0001	R = 0.7775; *P* = 0.0001
D	Cell cycle control,	wastewater	40	water	67	R = 0.2865;	R = 0.2998;	R = 0.2947;	R = 0.2992;

	cell division, chromosome partitioning					*P* = 0.0001	*P* = 0.0001	*P* = 0.0001	*P* = 0.0001
E	Amino acid transport and metabolism	Digestive system	122	Oral	27	R = 0.2203; *P* = 0.001	R = 0.2595; *P* = 0.0001	R = 0.223; *P* = 0.0017	R = 0.2195; *P* = 0.0009
E	Amino acid transport and metabolism	Digestive system	122	sediment	53	R = 0.2191; *P* = 0.0001	R = 0.1419; *P* = 0.0004	R = 0.2198; *P* = 0.0001	R = 0.1425; *P* = 0.0006
E	Amino acid transport and metabolism	Digestive system	122	skin	15	R = 0.3902; *P* = 0.0002	R = 0.3217; *P* = 0.001	R = 0.3878; *P* = 0.0001	R = 0.3069; *P* = 0.0015
E	Amino acid transport and metabolism	Digestive system	122	Soil	82	R = 0.6582; *P* = 0.0001	R = 0.5706; *P* = 0.0001	R = 0.6485; *P* = 0.0001	R = 0.5868; *P* = 0.0001
E	Amino acid transport and metabolism	Digestive system	122	wastewater	44	R = 0.2542; *P* = 0.0001	R = 0.1965; *P* = 0.0002	R = 0.2412; *P* = 0.0001	R = 0.1792; *P* = 0.0001

E	Amino acid transport and metabolism	Digestive system	122	water	68	R = 0.4544; *P* = 0.0001	R = 0.4615; *P* = 0.0001	R = 0.4592; *P* = 0.0001	R = 0.443; *P* = 0.0001
E	Amino acid transport and metabolism	Oral	27	sediment	53	R = 0.4222; *P* = 0.0001	R = 0.4311; *P* = 0.0001	R = 0.4248; *P* = 0.0001	R = 0.4183; *P* = 0.0001
E	Amino acid transport and metabolism	Oral	27	skin	15	R = 0.406; *P* = 0.0001	R = 0.3459; *P* = 0.0001	R = 0.3754; *P* = 0.0001	R = 0.3294; *P* = 0.0002
E	Amino acid transport and metabolism	Oral	27	Soil	82	R = 0.8365; *P* = 0.0001	R = 0.8066; *P* = 0.0001	R = 0.8446; *P* = 0.0001	R = 0.8203; *P* = 0.0001
E	Amino acid transport and metabolism	Oral	27	wastewater	44	R = 0.3736; *P* = 0.0001	R = 0.3302; *P* = 0.0001	R = 0.3535; *P* = 0.0001	R = 0.3278; *P* = 0.0001
E	Amino acid transport and metabolism	Oral	27	water	68	R = 0.0701; *P* = 0.0452	R = 0.0974; *P* = 0.0106	R = 0.0824; *P* = 0.0217	R = 0.0844; *P* = 0.0197

E	Amino acid transport and metabolism	sediment	53	skin	15	R = 0.2919; *P* = 0.0003	R = 0.2874; *P* = 0.0007	R = 0.2842; *P* = 0.0006	R = 0.2603; *P* = 0.0013
E	Amino acid transport and metabolism	Sediment	53	Soil	82	R = 0.3873; *P* = 0.0001	R = 0.3582; *P* = 0.0001	R = 0.398; *P* = 0.0001	R = 0.3722; *P* = 0.0001
E	Amino acid transport and metabolism	Sediment	53	wastewater	44	R = 0.121; *P* = 0.0001	R = 0.1042; *P* = 0.0005	R = 0.1036; *P* = 0.0002	R = 0.0941; *P* = 0.0004
E	Amino acid transport and metabolism	Sediment	53	water	68	R = 0.4121; *P* = 0.0001	R = 0.4407; *P* = 0.0001	R = 0.4224; *P* = 0.0001	R = 0.434; *P* = 0.0001
E	Amino acid transport and metabolism	Skin	15	Soil	82	R = 0.5786; *P* = 0.0001	R = 0.5186; *P* = 0.0001	R = 0.5923; *P* = 0.0001	R = 0.5389; *P* = 0.0001
E	Amino acid transport and metabolism	Skin	15	wastewater	44	R = 0.1644; *P* = 0.014	R = 0.1695; *P* = 0.0156	R = 0.155; *P* = 0.0189	R = 0.1501; *P* = 0.0232

E	Amino acid transport and metabolism	Skin	15	water	68	R = 0.3409; *P* = 0.0001	R = 0.3673; *P* = 0.0001	R = 0.3409; *P* = 0.0001	R = 0.353; *P* = 0.0001
E	Amino acid transport and metabolism	Soil	82	wastewater	44	R = 0.478; *P* = 0.0001	R = 0.4472; *P* = 0.0001	R = 0.4719; *P* = 0.0001	R = 0.4549; *P* = 0.0001
E	Amino acid transport and metabolism	Soil	82	water	68	R = 0.728; *P* = 0.0001	R = 0.7458; *P* = 0.0001	R = 0.7364; *P* = 0.0001	R = 0.7411; *P* = 0.0001
E	Amino acid transport and metabolism	wastewater	44	water	68	R = 0.3701; *P* = 0.0001	R = 0.3811; *P* = 0.0001	R = 0.3739; *P* = 0.0001	R = 0.3783; *P* = 0.0001
F	Nucleotide transport and metabolism	Digestive system	122	Oral	27	R = 0.2032; *P* = 0.0013	R = 0.2296; *P* = 0.0004	R = 0.2029; *P* = 0.0012	R = 0.1943; *P* = 0.0016
F	Nucleotide transport and metabolism	Digestive system	122	sediment	52	R = 0.1989; *P* = 0.0001	R = 0.1111; *P* = 0.003	R = 0.1976; *P* = 0.0001	R = 0.1102; *P* = 0.0032

F	Nucleotide transport and metabolism	Digestive system	122	skin	15	R = 0.3452; *P* = 0.0007	R = 0.2804; *P* = 0.003	R = 0.3427; *P* = 0.0006	R = 0.2592; *P* = 0.0053
F	Nucleotide transport and metabolism	Digestive system	122	Soil	82	R = 0.6679; *P* = 0.0001	R = 0.5521; *P* = 0.0001	R = 0.6601; *P* = 0.0001	R = 0.5714; *P* = 0.0001
F	Nucleotide transport and metabolism	Digestive system	122	wastewater	41	R = 0.198; *P* = 0.0001	R = 0.1402; *P* = 0.0032	R = 0.187; *P* = 0.0003	R = 0.114; *P* = 0.0127
F	Nucleotide transport and metabolism	Digestive system	122	water	68	R = 0.4439; *P* = 0.0001	R = 0.4474; *P* = 0.0001	R = 0.4515; *P* = 0.0001	R = 0.4284; *P* = 0.0001
F	Nucleotide transport and metabolism	Oral	27	sediment	52	R = 0.3517; *P* = 0.0001	R = 0.3614; *P* = 0.0001	R = 0.3497; *P* = 0.0001	R = 0.3443; *P* = 0.0001
F	Nucleotide transport and metabolism	Oral	27	skin	15	R = 0.3796; *P* = 0.0001	R = 0.334; *P* = 0.0001	R = 0.3525; *P* = 0.0001	R = 0.3212; *P* = 0.0001

F	Nucleotide transport and metabolism	Oral	27	Soil	82	R = 0.8145; *P* = 0.0001	R = 0.7802; *P* = 0.0001	R = 0.8263; *P* = 0.0001	R = 0.7979; *P* = 0.0001
F	Nucleotide transport and metabolism	Oral	27	wastewater	41	R = 0.3075; *P* = 0.0001	R = 0.2882; *P* = 0.0001	R = 0.2817; *P* = 0.0001	R = 0.267; *P* = 0.0001
F	Nucleotide transport and metabolism	Oral	27	water	68	R = 0.0909; *P* = 0.0146	R = 0.1286; *P* = 0.0017	R = 0.1103; *P* = 0.0046	R = 0.1155; *P* = 0.0025
F	Nucleotide transport and metabolism	sediment	52	skin	15	R = 0.2802; *P* = 0.001	R = 0.294; *P* = 0.0011	R = 0.2753; *P* = 0.001	R = 0.2624; *P* = 0.0018
F	Nucleotide transport and metabolism	sediment	52	Soil	82	R = 0.4139; *P* = 0.0001	R = 0.3828; *P* = 0.0001	R = 0.4256; *P* = 0.0001	R = 0.398; *P* = 0.0001
F	Nucleotide transport and metabolism	sediment	52	wastewater	41	R = 0.1132; *P* = 0.0005	R = 0.1079; *P* = 0.0006	R = 0.0998; *P* = 0.001	R = 0.0947; *P* = 0.0017

F	Nucleotide transport and metabolism	sediment	52	water	68	R = 0.4143; *P* = 0.0001	R = 0.4498; *P* = 0.0001	R = 0.4302; *P* = 0.0001	R = 0.4434; *P* = 0.0001
F	Nucleotide transport and metabolism	skin	15	Soil	82	R = 0.593; *P* = 0.0001	R = 0.5315; *P* = 0.0001	R = 0.6142; *P* = 0.0001	R = 0.5537; *P* = 0.0001
F	Nucleotide transport and metabolism	skin	15	wastewater	41	R = 0.2187; *P* = 0.0029	R = 0.2631; *P* = 0.0006	R = 0.2058; *P* = 0.005	R = 0.2257; *P* = 0.0024
F	Nucleotide transport and metabolism	skin	15	water	68	R = 0.3686; *P* = 0.0001	R = 0.4139; *P* = 0.0001	R = 0.3755; *P* = 0.0001	R = 0.3976; *P* = 0.0001
F	Nucleotide transport and metabolism	Soil	82	wastewater	41	R = 0.5469; *P* = 0.0001	R = 0.5263; *P* = 0.0001	R = 0.5528; *P* = 0.0001	R = 0.5395; *P* = 0.0001
F	Nucleotide transport and metabolism	Soil	82	water	68	R = 0.7667; *P* = 0.0001	R = 0.7811; *P* = 0.0001	R = 0.7774; *P* = 0.0001	R = 0.7801; *P* = 0.0001

F	Nucleotide transport and metabolism	wastewater	41	water	68	R = 0.3344; P = 0.0001	R = 0.3542; P = 0.0001	R = 0.3457; P = 0.0001	R = 0.3531; P = 0.0001
G	Carbohydrate transport and metabolism	Digestive system	122	Oral	27	R = 0.2185; P = 0.001	R = 0.219; P = 0.0009	R = 0.2151; P = 0.001	R = 0.186; P = 0.0038
G	Carbohydrate transport and metabolism	Digestive system	122	sediment	53	R = 0.2603; P = 0.0001	R = 0.1428; P = 0.0005	R = 0.2554; P = 0.0001	R = 0.1455; P = 0.0002
G	Carbohydrate transport and metabolism	Digestive system	122	skin	15	R = 0.4213; P = 0.0001	R = 0.3059; P = 0.0012	R = 0.4073; P = 0.0001	R = 0.2972; P = 0.0019
G	Carbohydrate transport and metabolism	Digestive system	122	Soil	82	R = 0.6857; P = 0.0001	R = 0.5498; P = 0.0001	R = 0.6777; P = 0.0001	R = 0.5781; P = 0.0001
G	Carbohydrate transport and metabolism	Digestive system	122	wastewater	42	R = 0.2171; P = 0.0001	R = 0.115; P = 0.0079	R = 0.2024; P = 0.0002	R = 0.1044; P = 0.0135

G	Carbohydrate transport and metabolism	Digestive system	122	water	68	R = 0.43; *P* = 0.0001	R = 0.4193; *P* = 0.0001	R = 0.4327; *P* = 0.0001	R = 0.3948; *P* = 0.0001
G	Carbohydrate transport and metabolism	Oral	27	sediment	53	R = 0.3455; *P* = 0.0001	R = 0.3281; *P* = 0.0001	R = 0.3367; *P* = 0.0001	R = 0.3155; *P* = 0.0001
G	Carbohydrate transport and metabolism	Oral	27	skin	15	R = 0.311; *P* = 0.0003	R = 0.257; *P* = 0.0005	R = 0.2826; *P* = 0.0003	R = 0.2435; *P* = 0.0006
G	Carbohydrate transport and metabolism	Oral	27	Soil	82	R = 0.7749; *P* = 0.0001	R = 0.7171; *P* = 0.0001	R = 0.7811; *P* = 0.0001	R = 0.738; *P* = 0.0001
G	Carbohydrate transport and metabolism	Oral	27	wastewater	42	R = 0.3049; *P* = 0.0001	R = 0.2975; *P* = 0.0001	R = 0.2798; *P* = 0.0001	R = 0.2704; *P* = 0.0001
G	Carbohydrate transport and metabolism	Oral	27	water	68	R = 0.0858; *P* = 0.0197	R = 0.1237; *P* = 0.0021	R = 0.0926; *P* = 0.0121	R = 0.1; *P* = 0.0091

G	Carbohydrate transport and metabolism	sediment	53	skin	15	R = 0.3068; *P* = 0.0004	R = 0.2922; *P* = 0.0007	R = 0.2812; *P* = 0.001	R = 0.2612; *P* = 0.0019
G	Carbohydrate transport and metabolism	sediment	53	Soil	82	R = 0.3782; *P* = 0.0001	R = 0.3416; *P* = 0.0001	R = 0.3847; *P* = 0.0001	R = 0.3561; *P* = 0.0001
G	Carbohydrate transport and metabolism	sediment	53	wastewater	42	R = 0.1112; *P* = 0.0006	R = 0.0894; *P* = 0.0021	R = 0.0945; *P* = 0.0012	R = 0.083; *P* = 0.0026
G	Carbohydrate transport and metabolism	sediment	53	water	68	R = 0.3672; *P* = 0.0001	R = 0.3952; *P* = 0.0001	R = 0.3775; *P* = 0.0001	R = 0.3877; *P* = 0.0001
G	Carbohydrate transport and metabolism	skin	15	Soil	82	R = 0.555; *P* = 0.0001	R = 0.464; *P* = 0.0001	R = 0.5674; *P* = 0.0001	R = 0.4918; *P* = 0.0001
G	Carbohydrate transport and metabolism	skin	15	wastewater	42	R = 0.226; *P* = 0.0016	R = 0.2702; *P* = 0.0007	R = 0.2134; *P* = 0.0016	R = 0.2293; *P* = 0.0006

G	Carbohydrate transport and metabolism	skin	15	water	68	R = 0.3462; *P* = 0.0001	R = 0.3887; *P* = 0.0001	R = 0.3451; *P* = 0.0001	R = 0.3685; *P* = 0.0001
G	Carbohydrate transport and metabolism	Soil	82	wastewater	42	R = 0.4952; *P* = 0.0001	R = 0.4508; *P* = 0.0001	R = 0.4907; *P* = 0.0001	R = 0.4647; *P* = 0.0001
G	Carbohydrate transport and metabolism	Soil	82	water	68	R = 0.7215; *P* = 0.0001	R = 0.7344; *P* = 0.0001	R = 0.7319; *P* = 0.0001	R = 0.7322; *P* = 0.0001
G	Carbohydrate transport and metabolism	wastewater	42	water	68	R = 0.3105; *P* = 0.0001	R = 0.3308; *P* = 0.0001	R = 0.316; *P* = 0.0001	R = 0.3244; *P* = 0.0001
H	Coenzyme transport and metabolism	Digestive system	120	Oral	27	R = 0.1733; *P* = 0.0075	R = 0.2165; *P* = 0.0014	R = 0.1804; *P* = 0.0049	R = 0.1808; *P* = 0.0049
H	Coenzyme transport and metabolism	Digestive system	120	sediment	53	R = 0.2075; *P* = 0.0001	R = 0.1538; *P* = 0.0003	R = 0.213; *P* = 0.0001	R = 0.1519; *P* = 0.0002

H	Coenzyme transport and metabolism	Digestive system	120	skin	15	R = 0.4491; *P* = 0.0001	R = 0.3564; *P* = 0.0004	R = 0.4426; *P* = 0.0001	R = 0.3428; *P* = 0.0008
H	Coenzyme transport and metabolism	Digestive system	120	Soil	82	R = 0.6545; *P* = 0.0001	R = 0.589; *P* = 0.0001	R = 0.6497; *P* = 0.0001	R = 0.601; *P* = 0.0001
H	Coenzyme transport and metabolism	Digestive system	120	wastewater	41	R = 0.2402; *P* = 0.0001	R = 0.19; *P* = 0.0004	R = 0.2328; *P* = 0.0001	R = 0.1728; *P* = 0.0008
H	Coenzyme transport and metabolism	Digestive system	120	water	68	R = 0.4372; *P* = 0.0001	R = 0.4463; *P* = 0.0001	R = 0.4475; *P* = 0.0001	R = 0.4289; *P* = 0.0001
H	Coenzyme transport and metabolism	Oral	27	sediment	53	R = 0.3448; *P* = 0.0001	R = 0.3672; *P* = 0.0001	R = 0.342; *P* = 0.0001	R = 0.3492; *P* = 0.0001
H	Coenzyme transport and metabolism	Oral	27	skin	15	R = 0.4595; *P* = 0.0001	R = 0.3855; *P* = 0.0001	R = 0.4294; *P* = 0.0001	R = 0.3619; *P* = 0.0001

H	Coenzyme transport and metabolism	Oral	27	Soil	82	R = 0.7873; *P* = 0.0001	R = 0.7694; *P* = 0.0001	R = 0.8039; *P* = 0.0001	R = 0.7831; *P* = 0.0001
H	Coenzyme transport and metabolism	Oral	27	wastewater	41	R = 0.3153; *P* = 0.0001	R = 0.2795; *P* = 0.0001	R = 0.298; *P* = 0.0001	R = 0.2716; *P* = 0.0001
H	Coenzyme transport and metabolism	Oral	27	water	68	R = 0.0491; *P* = 0.1117	R = 0.0768; *P* = 0.0323	R = 0.0711; *P* = 0.04	R = 0.0732; *P* = 0.0356
H	Coenzyme transport and metabolism	sediment	53	skin	15	R = 0.3009; *P* = 0.0003	R = 0.2555; *P* = 0.002	R = 0.2921; *P* = 0.0008	R = 0.2276; *P* = 0.0037
H	Coenzyme transport and metabolism	sediment	53	Soil	82	R = 0.3686; *P* = 0.0001	R = 0.338; *P* = 0.0001	R = 0.3823; *P* = 0.0001	R = 0.3527; *P* = 0.0001
H	Coenzyme transport and metabolism	sediment	53	wastewater	41	R = 0.123; *P* = 0.0003	R = 0.1022; *P* = 0.0006	R = 0.1047; *P* = 0.0005	R = 0.0899; *P* = 0.0015

H	Coenzyme transport and metabolism	sediment	53	water	68	R = 0.3757; *P* = 0.0001	R = 0.4156; *P* = 0.0001	R = 0.3937; *P* = 0.0001	R = 0.4083; *P* = 0.0001
H	Coenzyme transport and metabolism	skin	15	Soil	82	R = 0.492; *P* = 0.0001	R = 0.4587; *P* = 0.0001	R = 0.5194; *P* = 0.0001	R = 0.4744; *P* = 0.0001
H	Coenzyme transport and metabolism	skin	15	wastewater	41	R = 0.2139; *P* = 0.0034	R = 0.215; *P* = 0.005	R = 0.2003; *P* = 0.0058	R = 0.1873; *P* = 0.0087
H	Coenzyme transport and metabolism	skin	15	water	68	R = 0.3365; *P* = 0.0001	R = 0.3603; *P* = 0.0001	R = 0.3445; *P* = 0.0001	R = 0.3494; *P* = 0.0001
H	Coenzyme transport and metabolism	Soil	82	wastewater	41	R = 0.4745; *P* = 0.0001	R = 0.4642; *P* = 0.0001	R = 0.4797; *P* = 0.0001	R = 0.4735; *P* = 0.0001
H	Coenzyme transport and metabolism	Soil	82	water	68	R = 0.7025; *P* = 0.0001	R = 0.7303; *P* = 0.0001	R = 0.7182; *P* = 0.0001	R = 0.7271; *P* = 0.0001

H	Coenzyme transport and metabolism	wastewater	41	water	68	R = 0.3077; *P* = 0.0001	R = 0.3274; *P* = 0.0001	R = 0.3197; *P* = 0.0001	R = 0.3262; *P* = 0.0001
I	Lipid transport and metabolism	Digestive system	121	Oral	27	R = 0.1758; *P* = 0.0052	R = 0.1968; *P* = 0.0027	R = 0.1801; *P* = 0.0041	R = 0.1613; *P* = 0.0069
I	Lipid transport and metabolism	Digestive system	121	sediment	53	R = 0.3306; *P* = 0.0001	R = 0.2389; *P* = 0.0001	R = 0.32; *P* = 0.0001	R = 0.2508; *P* = 0.0001
I	Lipid transport and metabolism	Digestive system	121	skin	15	R = 0.5131; *P* = 0.0001	R = 0.4273; *P* = 0.0002	R = 0.4999; *P* = 0.0001	R = 0.4244; *P* = 0.0001
I	Lipid transport and metabolism	Digestive system	121	Soil	82	R = 0.7492; *P* = 0.0001	R = 0.676; *P* = 0.0001	R = 0.7391; *P* = 0.0001	R = 0.696; *P* = 0.0001
I	Lipid transport and metabolism	Digestive system	121	wastewater	43	R = 0.3635; *P* = 0.0001	R = 0.2716; *P* = 0.0001	R = 0.3342; *P* = 0.0001	R = 0.2686; *P* = 0.0001
I	Lipid transport and metabolism	Digestive system	121	water	67	R = 0.4278; *P* = 0.0001	R = 0.4215; *P* = 0.0001	R = 0.4281; *P* = 0.0001	R = 0.401; *P* = 0.0001
I	Lipid transport and metabolism	Oral	27	sediment	53	R = 0.5012; *P* = 0.0001	R = 0.5112; *P* = 0.0001	R = 0.5056; *P* = 0.0001	R = 0.5007; *P* = 0.0001
I	Lipid transport	Oral	27	skin	15	R = 0.5122;	R = 0.4708;	R = 0.4799;	R = 0.4481;

	and metabolism					*P* = 0.0001	*P* = 0.0001	*P* = 0.0001	*P* = 0.0001
I	Lipid transport and metabolism	Oral	27	Soil	82	R = 0.8662; *P* = 0.0001	R = 0.8456; *P* = 0.0001	R = 0.8716; *P* = 0.0001	R = 0.8547; *P* = 0.0001
I	Lipid transport and metabolism	Oral	27	wastewater	43	R = 0.4482; *P* = 0.0001	R = 0.4038; *P* = 0.0001	R = 0.4295; *P* = 0.0001	R = 0.4002; *P* = 0.0001
I	Lipid transport and metabolism	Oral	27	water	67	R = 0.0363; *P* = 0.1609	R = 0.0501; *P* = 0.1026	R = 0.0439; *P* = 0.1174	R = 0.0404; *P* = 0.1355
I	Lipid transport and metabolism	sediment	53	skin	15	R = 0.3048; *P* = 0.0007	R = 0.2956; *P* = 0.0012	R = 0.3022; *P* = 0.0009	R = 0.2733; *P* = 0.0018
I	Lipid transport and metabolism	sediment	53	Soil	82	R = 0.3579; *P* = 0.0001	R = 0.3399; *P* = 0.0001	R = 0.3692; *P* = 0.0001	R = 0.3495; *P* = 0.0001
I	Lipid transport and metabolism	sediment	53	wastewater	43	R = 0.1567; *P* = 0.0001	R = 0.1268; *P* = 0.0001	R = 0.1355; *P* = 0.0001	R = 0.1167; *P* = 0.0001
I	Lipid transport and metabolism	sediment	53	water	67	R = 0.3911; *P* = 0.0001	R = 0.4149; *P* = 0.0001	R = 0.3997; *P* = 0.0001	R = 0.4089; *P* = 0.0001
I	Lipid transport and metabolism	skin	15	Soil	82	R = 0.5451; *P* = 0.0001	R = 0.4789; *P* = 0.0001	R = 0.5562; *P* = 0.0001	R = 0.4935; *P* = 0.0001
I	Lipid transport	skin	15	wastewater	43	R = 0.1711;	R = 0.1809;	R = 0.162;	R = 0.1568;

	and metabolism					$P = 0.0118$	$P = 0.0087$	$P = 0.016$	$P = 0.0168$
I	Lipid transport and metabolism	skin	15	water	67	R = 0.3362; $P = 0.0001$	R = 0.3648; $P = 0.0001$	R = 0.3366; $P = 0.0001$	R = 0.3469; $P = 0.0001$
I	Lipid transport and metabolism	Soil	82	wastewater	43	R = 0.4839; $P = 0.0001$	R = 0.4407; $P = 0.0001$	R = 0.4711; $P = 0.0001$	R = 0.4458; $P = 0.0001$
I	Lipid transport and metabolism	Soil	82	water	67	R = 0.6967; $P = 0.0001$	R = 0.715; $P = 0.0001$	R = 0.7037; $P = 0.0001$	R = 0.7093; $P = 0.0001$
I	Lipid transport and metabolism	wastewater	43	water	67	R = 0.3568; $P = 0.0001$	R = 0.3636; $P = 0.0001$	R = 0.3558; $P = 0.0001$	R = 0.3587; $P = 0.0001$
J	Translation, ribosomal structure and biogenesis	Digestive system	122	Oral	27	R = 0.2098; $P = 0.0011$	R = 0.2361; $P = 0.0005$	R = 0.2064; $P = 0.0016$	R = 0.1975; $P = 0.002$
J	Translation, ribosomal structure and biogenesis	Digestive system	122	sediment	53	R = 0.2172; $P = 0.0001$	R = 0.1272; $P = 0.0016$	R = 0.2191; $P = 0.0001$	R = 0.1285; $P = 0.0013$
J	Translation,	Digestive	122	skin	15	R = 0.3501;	R = 0.2873;	R = 0.3486;	R = 0.2703;

	ribosomal structure and biogenesis	system				*P* = 0.0007	*P* = 0.002	*P* = 0.0005	*P* = 0.0036
J	Translation, ribosomal structure and biogenesis	Digestive system	122	Soil	82	R = 0.6634*;* *P* = 0.0001	R = 0.5651*;* *P* = 0.0001	R = 0.6635*;* *P* = 0.0001	R = 0.5859*;* *P* = 0.0001
J	Translation, ribosomal structure and biogenesis	Digestive system	122	wastewater	44	R = 0.227*;* *P* = 0.0002	R = 0.1628*;* *P* = 0.0005	R = 0.2149*;* *P* = 0.0001	R = 0.1434*;* *P* = 0.0019
J	Translation, ribosomal structure and biogenesis	Digestive system	122	water	68	R = 0.4235*;* *P* = 0.0001	R = 0.4244*;* *P* = 0.0001	R = 0.431*;* *P* = 0.0001	R = 0.4054*;* *P* = 0.0001
J	Translation, ribosomal structure and	Oral	27	sediment	53	R = 0.4214*;* *P* = 0.0001	R = 0.4284*;* *P* = 0.0001	R = 0.4177*;* *P* = 0.0001	R = 0.4116*;* *P* = 0.0001

	biogenesis								
J	Translation, ribosomal structure and biogenesis	Oral	27	skin	15	R = 0.4523; P = 0.0001	R = 0.4034; P = 0.0001	R = 0.4248; P = 0.0001	R = 0.3933; P = 0.0001
J	Translation, ribosomal structure and biogenesis	Oral	27	Soil	82	R = 0.8292; P = 0.0001	R = 0.8066; P = 0.0001	R = 0.8429; P = 0.0001	R = 0.822; P = 0.0001
J	Translation, ribosomal structure and biogenesis	Oral	27	wastewater	44	R = 0.2801; P = 0.0001	R = 0.2744; P = 0.0001	R = 0.266; P = 0.0001	R = 0.2623; P = 0.0001
J	Translation, ribosomal structure and biogenesis	Oral	27	water	68	R = 0.0776; P = 0.0325	R = 0.1018; P = 0.009	R = 0.0884; P = 0.0177	R = 0.0854; P = 0.0205
J	Translation,	sediment	53	skin	15	R = 0.3266;	R = 0.3225;	R = 0.31;	R = 0.2857;

	ribosomal structure and biogenesis					*P* = 0.0002	*P* = 0.0002	*P* = 0.0002	*P* = 0.0007
J	Translation, ribosomal structure and biogenesis	sediment	53	Soil	82	R = 0.4029; *P* = 0.0001	R = 0.3836; *P* = 0.0001	R = 0.4153; *P* = 0.0001	R = 0.3956; *P* = 0.0001
J	Translation, ribosomal structure and biogenesis	sediment	53	wastewater	44	R = 0.1109; *P* = 0.0004	R = 0.1012; *P* = 0.0006	R = 0.0978; *P* = 0.0003	R = 0.0898; *P* = 0.001
J	Translation, ribosomal structure and biogenesis	sediment	53	water	68	R = 0.4192; *P* = 0.0001	R = 0.4534; *P* = 0.0001	R = 0.4356; *P* = 0.0001	R = 0.4475; *P* = 0.0001
J	Translation, ribosomal structure and	skin	15	Soil	82	R = 0.5793; *P* = 0.0001	R = 0.5405; *P* = 0.0001	R = 0.604; *P* = 0.0001	R = 0.558; *P* = 0.0001

	biogenesis								
J	Translation, ribosomal structure and biogenesis	skin	15	wastewater	44	R = 0.1748; *P* = 0.0138	R = 0.1933; *P* = 0.0107	R = 0.1567; *P* = 0.0218	R = 0.1656; *P* = 0.0199
J	Translation, ribosomal structure and biogenesis	skin	15	water	68	R = 0.3696; *P* = 0.0001	R = 0.4046; *P* = 0.0001	R = 0.3767; *P* = 0.0001	R = 0.3895; *P* = 0.0001
J	Translation, ribosomal structure and biogenesis	Soil	82	wastewater	44	R = 0.5015; *P* = 0.0001	R = 0.4831; *P* = 0.0001	R = 0.5136; *P* = 0.0001	R = 0.4965; *P* = 0.0001
J	Translation, ribosomal structure and biogenesis	Soil	82	water	68	R = 0.7704; *P* = 0.0001	R = 0.7843; *P* = 0.0001	R = 0.7822; *P* = 0.0001	R = 0.7841; *P* = 0.0001
J	Translation,	wastewater	44	water	68	R = 0.3263;	R = 0.3533;	R = 0.3392;	R = 0.3511;

	ribosomal structure and biogenesis					*P* = 0.0001	*P* = 0.0001	*P* = 0.0001	*P* = 0.0001
K	Transcription	Digestive system	121	Oral	27	R = 0.202*;* *P* = 0.0022	R = 0.1904*;* *P* = 0.0029	R = 0.1942*;* *P* = 0.0023	R = 0.1644*;* *P* = 0.0069
K	Transcription	Digestive system	121	sediment	53	R = 0.2771*;* *P* = 0.0001	R = 0.186*;* *P* = 0.0001	R = 0.2773*;* *P* = 0.0001	R = 0.1894*;* *P* = 0.0001
K	Transcription	Digestive system	121	skin	14	R = 0.5024*;* *P* = 0.0001	R = 0.367*;* *P* = 0.0004	R = 0.4839*;* *P* = 0.0001	R = 0.3578*;* *P* = 0.0001
K	Transcription	Digestive system	121	Soil	82	R = 0.7458*;* *P* = 0.0001	R = 0.6681*;* *P* = 0.0001	R = 0.7403*;* *P* = 0.0001	R = 0.686*;* *P* = 0.0001
K	Transcription	Digestive system	121	wastewater	43	R = 0.2985*;* *P* = 0.0001	R = 0.1847*;* *P* = 0.0003	R = 0.2796*;* *P* = 0.0001	R = 0.1768*;* *P* = 0.0003
K	Transcription	Digestive system	121	water	68	R = 0.4183*;* *P* = 0.0001	R = 0.3997*;* *P* = 0.0001	R = 0.4237*;* *P* = 0.0001	R = 0.3842*;* *P* = 0.0001
K	Transcription	Oral	27	sediment	53	R = 0.4057*;* *P* = 0.0001	R = 0.3894*;* *P* = 0.0001	R = 0.4013*;* *P* = 0.0001	R = 0.383*;* *P* = 0.0001
K	Transcription	Oral	27	skin	14	R = 0.4183*;*	R = 0.3277*;*	R = 0.3813*;*	R = 0.3143*;*

						P = 0.0001	*P* = 0.0001	*P* = 0.0001	*P* = 0.0003
K	Transcription	Oral	27	Soil	82	R = 0.8237; *P* = 0.0001	R = 0.7961; *P* = 0.0001	R = 0.8353; *P* = 0.0001	R = 0.8112; *P* = 0.0001
K	Transcription	Oral	27	wastewater	43	R = 0.3387; *P* = 0.0001	R = 0.315; *P* = 0.0001	R = 0.3183; *P* = 0.0001	R = 0.307; *P* = 0.0001
K	Transcription	Oral	27	water	68	R = 0.0422; *P* = 0.1367	R = 0.0503; *P* = 0.1009	R = 0.0495; *P* = 0.1001	R = 0.0435; *P* = 0.1235
K	Transcription	sediment	53	skin	14	R = 0.3096; *P* = 0.0003	R = 0.2398; *P* = 0.0039	R = 0.2889; *P* = 0.0009	R = 0.2264; *P* = 0.0066
K	Transcription	sediment	53	Soil	82	R = 0.4142; *P* = 0.0001	R = 0.3691; *P* = 0.0001	R = 0.4214; *P* = 0.0001	R = 0.3849; *P* = 0.0001
K	Transcription	sediment	53	wastewater	43	R = 0.1164; *P* = 0.0004	R = 0.0794; *P* = 0.0035	R = 0.0952; *P* = 0.0013	R = 0.0734; *P* = 0.0053
K	Transcription	sediment	53	water	68	R = 0.3876; *P* = 0.0001	R = 0.4088; *P* = 0.0001	R = 0.3967; *P* = 0.0001	R = 0.4047; *P* = 0.0001
K	Transcription	skin	14	Soil	82	R = 0.4913; *P* = 0.0001	R = 0.4773; *P* = 0.0001	R = 0.5213; *P* = 0.0001	R = 0.4981; *P* = 0.0001
K	Transcription	skin	14	wastewater	43	R = 0.1761;	R = 0.1928;	R = 0.1661;	R = 0.1749;

						P = 0.0118	*P* = 0.0085	*P* = 0.0136	*P* = 0.0131
K	Transcription	skin	14	water	68	R = 0.3526; *P* = 0.0001	R = 0.3491; *P* = 0.0001	R = 0.3449; *P* = 0.0001	R = 0.3394; *P* = 0.0001
K	Transcription	Soil	82	wastewater	43	R = 0.4798; *P* = 0.0001	R = 0.4786; *P* = 0.0001	R = 0.4874; *P* = 0.0001	R = 0.486; *P* = 0.0001
K	Transcription	Soil	82	water	68	R = 0.7357; *P* = 0.0001	R = 0.7467; *P* = 0.0001	R = 0.7434; *P* = 0.0001	R = 0.7451; *P* = 0.0001
K	Transcription	wastewater	43	water	68	R = 0.3257; *P* = 0.0001	R = 0.3358; *P* = 0.0001	R = 0.3266; *P* = 0.0001	R = 0.3327; *P* = 0.0001
L	Replication, recombination and repair	Digestive system	122	Oral	27	R = 0.1717; *P* = 0.0058	R = 0.1767; *P* = 0.0055	R = 0.1722; *P* = 0.0056	R = 0.1483; *P* = 0.0116
L	Replication, recombination and repair	Digestive system	122	sediment	53	R = 0.248; *P* = 0.0001	R = 0.1565; *P* = 0.0002	R = 0.2433; *P* = 0.0001	R = 0.1524; *P* = 0.0001
L	Replication, recombination and repair	Digestive system	122	skin	15	R = 0.4159; *P* = 0.0001	R = 0.3132; *P* = 0.0009	R = 0.4065; *P* = 0.0001	R = 0.2918; *P* = 0.0019

L	Replication, recombination and repair	Digestive system	122	Soil	82	R = 0.7075; *P* = 0.0001	R = 0.6297; *P* = 0.0001	R = 0.6964; *P* = 0.0001	R = 0.6359; *P* = 0.0001
L	Replication, recombination and repair	Digestive system	122	wastewater	43	R = 0.2486; *P* = 0.0001	R = 0.1646; *P* = 0.0008	R = 0.2357; *P* = 0.0001	R = 0.1435; *P* = 0.002
L	Replication, recombination and repair	Digestive system	122	water	68	R = 0.3903; *P* = 0.0001	R = 0.3738; *P* = 0.0001	R = 0.397; *P* = 0.0001	R = 0.3526; *P* = 0.0001
L	Replication, recombination and repair	Oral	27	sediment	53	R = 0.3895; *P* = 0.0001	R = 0.3953; *P* = 0.0001	R = 0.38; *P* = 0.0001	R = 0.3757; *P* = 0.0001
L	Replication, recombination and repair	Oral	27	skin	15	R = 0.4109; *P* = 0.0001	R = 0.3442; *P* = 0.0001	R = 0.3813; *P* = 0.0001	R = 0.3276; *P* = 0.0001
L	Replication, recombination and repair	Oral	27	Soil	82	R = 0.8156; *P* = 0.0001	R = 0.794; *P* = 0.0001	R = 0.8264; *P* = 0.0001	R = 0.8055; *P* = 0.0001

L	Replication, recombination and repair	Oral	27	wastewater	43	R = 0.2843; *P* = 0.0001	R = 0.2679; *P* = 0.0001	R = 0.2714; *P* = 0.0001	R = 0.2589; *P* = 0.0001
L	Replication, recombination and repair	Oral	27	water	68	R = 0.0471; *P* = 0.1076	R = 0.0641; *P* = 0.0578	R = 0.0631; *P* = 0.0589	R = 0.0557; *P* = 0.0755
L	Replication, recombination and repair	sediment	53	skin	15	R = 0.2536; *P* = 0.0015	R = 0.2265; *P* = 0.0036	R = 0.239; *P* = 0.0032	R = 0.2037; *P* = 0.009
L	Replication, recombination and repair	sediment	53	Soil	82	R = 0.3811; *P* = 0.0001	R = 0.3567; *P* = 0.0001	R = 0.389; *P* = 0.0001	R = 0.369; *P* = 0.0001
L	Replication, recombination and repair	sediment	53	wastewater	43	R = 0.0976; *P* = 0.0014	R = 0.0818; *P* = 0.0017	R = 0.083; *P* = 0.0028	R = 0.0727; *P* = 0.0038
L	Replication, recombination and repair	sediment	53	water	68	R = 0.3998; *P* = 0.0001	R = 0.4296; *P* = 0.0001	R = 0.4136; *P* = 0.0001	R = 0.4227; *P* = 0.0001

L	Replication, recombination and repair	skin	15	Soil	82	R = 0.5202; *P* = 0.0001	R = 0.49; *P* = 0.0001	R = 0.5431; *P* = 0.0001	R = 0.5043; *P* = 0.0001
L	Replication, recombination and repair	skin	15	wastewater	43	R = 0.1701; *P* = 0.0121	R = 0.1787; *P* = 0.0104	R = 0.1603; *P* = 0.0148	R = 0.1563; *P* = 0.0198
L	Replication, recombination and repair	skin	15	water	68	R = 0.343; *P* = 0.0001	R = 0.361; *P* = 0.0001	R = 0.3519; *P* = 0.0001	R = 0.3527; *P* = 0.0001
L	Replication, recombination and repair	Soil	82	wastewater	43	R = 0.4889; *P* = 0.0001	R = 0.4755; *P* = 0.0001	R = 0.4991; *P* = 0.0001	R = 0.4847; *P* = 0.0001
L	Replication, recombination and repair	Soil	82	water	68	R = 0.7623; *P* = 0.0001	R = 0.778; *P* = 0.0001	R = 0.7738; *P* = 0.0001	R = 0.7762; *P* = 0.0001
L	Replication, recombination and repair	wastewater	43	water	68	R = 0.3054; *P* = 0.0001	R = 0.3236; *P* = 0.0001	R = 0.3176; *P* = 0.0001	R = 0.3223; *P* = 0.0001

M	Cell wall/membrane/envelope biogenesis	Digestive system	116	Oral	27	R = 0.2149; *P* = 0.0012	R = 0.2435; *P* = 0.0004	R = 0.2137; *P* = 0.0009	R = 0.2057; *P* = 0.0019
M	Cell wall/membrane/envelope biogenesis	Digestive system	116	sediment	53	R = 0.2776; *P* = 0.0001	R = 0.1712; *P* = 0.0002	R = 0.2653; *P* = 0.0001	R = 0.1668; *P* = 0.0001
M	Cell wall/membrane/envelope biogenesis	Digestive system	116	skin	15	R = 0.4679; *P* = 0.0001	R = 0.3843; *P* = 0.0002	R = 0.4562; *P* = 0.0001	R = 0.3683; *P* = 0.0006
M	Cell wall/membrane/envelope biogenesis	Digestive system	116	Soil	82	R = 0.6869; *P* = 0.0001	R = 0.5917; *P* = 0.0001	R = 0.6721; *P* = 0.0001	R = 0.6041; *P* = 0.0001
M	Cell wall/membrane/envelope biogenesis	Digestive system	116	wastewater	42	R = 0.2577; *P* = 0.0001	R = 0.1848; *P* = 0.0003	R = 0.2439; *P* = 0.0001	R = 0.1594; *P* = 0.0006
M	Cell wall/membrane/envelope biogenesis	Digestive system	116	water	68	R = 0.4504; *P* = 0.0001	R = 0.4412; *P* = 0.0001	R = 0.4544; *P* = 0.0001	R = 0.4208; *P* = 0.0001

M	Cell wall/membrane/envelope biogenesis	Oral	27	sediment	53	R = 0.358; P = 0.0001	R = 0.3329; P = 0.0001	R = 0.3449; P = 0.0001	R = 0.3184; P = 0.0001
M	Cell wall/membrane/envelope biogenesis	Oral	27	skin	15	R = 0.3962; P = 0.0002	R = 0.3239; P = 0.0001	R = 0.3615; P = 0.0001	R = 0.3039; P = 0.0004
M	Cell wall/membrane/envelope biogenesis	Oral	27	Soil	82	R = 0.7763; P = 0.0001	R = 0.7168; P = 0.0001	R = 0.7771; P = 0.0001	R = 0.7348; P = 0.0001
M	Cell wall/membrane/envelope biogenesis	Oral	27	wastewater	42	R = 0.2509; P = 0.0001	R = 0.2194; P = 0.0002	R = 0.2284; P = 0.0001	R = 0.2073; P = 0.0003
M	Cell wall/membrane/envelope biogenesis	Oral	27	water	68	R = 0.0631; P = 0.0555	R = 0.1085; P = 0.0056	R = 0.0887; P = 0.0147	R = 0.0955; P = 0.012
M	Cell wall/membrane/envelope biogenesis	sediment	53	skin	15	R = 0.3078; P = 0.0004	R = 0.2879; P = 0.0004	R = 0.2967; P = 0.0008	R = 0.2625; P = 0.001

M	Cell wall/membrane/envelope biogenesis	sediment	53	Soil	82	R = 0.3649; *P* = 0.0001	R = 0.336; *P* = 0.0001	R = 0.3693; *P* = 0.0001	R = 0.3485; *P* = 0.0001
M	Cell wall/membrane/envelope biogenesis	sediment	53	wastewater	42	R = 0.1185; *P* = 0.0001	R = 0.1001; *P* = 0.0008	R = 0.1019; *P* = 0.0004	R = 0.0932; *P* = 0.0014
M	Cell wall/membrane/envelope biogenesis	sediment	53	water	68	R = 0.3844; *P* = 0.0001	R = 0.427; *P* = 0.0001	R = 0.4041; *P* = 0.0001	R = 0.4199; *P* = 0.0001
M	Cell wall/membrane/envelope biogenesis	skin	15	Soil	82	R = 0.506; *P* = 0.0001	R = 0.4377; *P* = 0.0001	R = 0.5135; *P* = 0.0001	R = 0.4557; *P* = 0.0001
M	Cell wall/membrane/envelope biogenesis	skin	15	wastewater	42	R = 0.2253; *P* = 0.0016	R = 0.2548; *P* = 0.001	R = 0.2139; *P* = 0.0036	R = 0.2268; *P* = 0.0021
M	Cell wall/membrane/envelope biogenesis	skin	15	water	68	R = 0.3565; *P* = 0.0001	R = 0.4147; *P* = 0.0001	R = 0.3755; *P* = 0.0001	R = 0.3966; *P* = 0.0001

M	Cell wall/membrane/envelope biogenesis	Soil	82	wastewater	42	R = 0.5049; *P* = 0.0001	R = 0.4851; *P* = 0.0001	R = 0.5088; *P* = 0.0001	R = 0.497; *P* = 0.0001
M	Cell wall/membrane/envelope biogenesis	Soil	82	water	68	R = 0.7262; *P* = 0.0001	R = 0.7498; *P* = 0.0001	R = 0.7434; *P* = 0.0001	R = 0.7483; *P* = 0.0001
M	Cell wall/membrane/envelope biogenesis	wastewater	42	water	68	R = 0.2839; *P* = 0.0001	R = 0.3132; *P* = 0.0001	R = 0.2995; *P* = 0.0001	R = 0.3106; *P* = 0.0001
N	Cell motility	Digestive system	106	Oral	27	R = 0.112; *P* = 0.0294	R = 0.0989; *P* = 0.044	R = 0.1242; *P* = 0.0193	R = 0.1062; *P* = 0.0325
N	Cell motility	Digestive system	106	sediment	49	R = 0.4002; *P* = 0.0001	R = 0.3118; *P* = 0.0001	R = 0.4035; *P* = 0.0001	R = 0.3326; *P* = 0.0001
N	Cell motility	Digestive system	106	skin	9	R = 0.709; *P* = 0.0001	R = 0.5991; *P* = 0.0001	R = 0.6925; *P* = 0.0001	R = 0.609; *P* = 0.0001
N	Cell motility	Digestive system	106	Soil	82	R = 0.8351; *P* = 0.0001	R = 0.7897; *P* = 0.0001	R = 0.8315; *P* = 0.0001	R = 0.7988; *P* = 0.0001
N	Cell motility	Digestive	106	wastewater	40	R = 0.3893;	R = 0.2732;	R = 0.374;	R = 0.2833;

		system				*P* = 0.0001	*P* = 0.0001	*P* = 0.0001	*P* = 0.0001
N	Cell motility	Digestive system	106	water	62	R = 0.3639; *P* = 0.0001	R = 0.3449; *P* = 0.0001	R = 0.3671; *P* = 0.0001	R = 0.3122; *P* = 0.0001
N	Cell motility	Oral	27	sediment	49	R = 0.3334; *P* = 0.0001	R = 0.2973; *P* = 0.0001	R = 0.3009; *P* = 0.0001	R = 0.2646; *P* = 0.0001
N	Cell motility	Oral	27	skin	9	R = 0.6581; *P* = 0.0001	R = 0.5529; *P* = 0.0001	R = 0.5884; *P* = 0.0001	R = 0.5059; *P* = 0.0001
N	Cell motility	Oral	27	Soil	82	R = 0.8281; *P* = 0.0001	R = 0.7753; *P* = 0.0001	R = 0.8251; *P* = 0.0001	R = 0.7847; *P* = 0.0001
N	Cell motility	Oral	27	wastewater	40	R = 0.341; *P* = 0.0001	R = 0.2915; *P* = 0.0001	R = 0.3021; *P* = 0.0001	R = 0.2604; *P* = 0.0001
N	Cell motility	Oral	27	water	62	R = 0.1908; *P* = 0.0001	R = 0.1631; *P* = 0.0004	R = 0.1894; *P* = 0.0002	R = 0.1406; *P* = 0.0006
N	Cell motility	sediment	49	skin	9	R = 0.2982; *P* = 0.0007	R = 0.2226; *P* = 0.0091	R = 0.2451; *P* = 0.0046	R = 0.1872; *P* = 0.0216
N	Cell motility	sediment	49	Soil	82	R = 0.4328; *P* = 0.0001	R = 0.4177; *P* = 0.0001	R = 0.4413; *P* = 0.0001	R = 0.4324; *P* = 0.0001
N	Cell motility	sediment	49	wastewater	40	R = 0.0603;	R = 0.0504;	R = 0.0479;	R = 0.0443;

						P = 0.0101	*P* = 0.0208	*P* = 0.0236	*P* = 0.0291
N	Cell motility	sediment	49	water	62	R = 0.3757; *P* = 0.0001	R = 0.392; *P* = 0.0001	R = 0.38; *P* = 0.0001	R = 0.3842; *P* = 0.0001
N	Cell motility	skin	9	Soil	82	R = 0.3624; *P* = 0.0021	R = 0.3032; *P* = 0.0066	R = 0.3019; *P* = 0.0061	R = 0.2868; *P* = 0.0069
N	Cell motility	skin	9	wastewater	40	R = 0.2344; *P* = 0.0077	R = 0.2577; *P* = 0.0023	R = 0.2295; *P* = 0.0071	R = 0.2411; *P* = 0.0032
N	Cell motility	skin	9	water	62	R = 0.5304; *P* = 0.0001	R = 0.5098; *P* = 0.0001	R = 0.5127; *P* = 0.0001	R = 0.4947; *P* = 0.0001
N	Cell motility	Soil	82	wastewater	40	R = 0.5182; *P* = 0.0001	R = 0.5284; *P* = 0.0001	R = 0.5323; *P* = 0.0001	R = 0.5434; *P* = 0.0001
N	Cell motility	Soil	82	water	62	R = 0.7784; *P* = 0.0001	R = 0.7845; *P* = 0.0001	R = 0.784; *P* = 0.0001	R = 0.7839; *P* = 0.0001
N	Cell motility	wastewater	40	water	62	R = 0.3128; *P* = 0.0001	R = 0.3174; *P* = 0.0001	R = 0.312; *P* = 0.0001	R = 0.3127; *P* = 0.0001
O	Posttranslational modification, protein turnover,	Digestive system	122	Oral	27	R = 0.1934; *P* = 0.0022	R = 0.2205; *P* = 0.001	R = 0.1844; *P* = 0.0036	R = 0.176; *P* = 0.0057

	chaperones								
O	Posttranslational modification, protein turnover, chaperones	Digestive system	122	sediment	53	R = 0.2535; *P* = 0.0001	R = 0.1671; *P* = 0.0001	R = 0.2511; *P* = 0.0001	R = 0.1695; *P* = 0.0001
O	Posttranslational modification, protein turnover, chaperones	Digestive system	122	skin	15	R = 0.4304; *P* = 0.0002	R = 0.3528; *P* = 0.0007	R = 0.4186; *P* = 0.0001	R = 0.3413; *P* = 0.0008
O	Posttranslational modification, protein turnover, chaperones	Digestive system	122	Soil	82	R = 0.6969; *P* = 0.0001	R = 0.6189; *P* = 0.0001	R = 0.6929; *P* = 0.0001	R = 0.638; *P* = 0.0001
O	Posttranslational modification, protein turnover, chaperones	Digestive system	122	wastewater	41	R = 0.2319; *P* = 0.0002	R = 0.163; *P* = 0.0006	R = 0.2145; *P* = 0.0002	R = 0.1423; *P* = 0.0028
O	Posttranslational	Digestive	122	water	68	R = 0.4489;	R = 0.4427;	R = 0.4483;	R = 0.4229;

	modification, protein turnover, chaperones	system				*P* = 0.0001	*P* = 0.0001	*P* = 0.0001	*P* = 0.0001
O	Posttranslational modification, protein turnover, chaperones	Oral	27	sediment	53	R = 0.4054; *P* = 0.0001	R = 0.3985; *P* = 0.0001	R = 0.3992; *P* = 0.0001	R = 0.3836; *P* = 0.0001
O	Posttranslational modification, protein turnover, chaperones	Oral	27	skin	15	R = 0.458; *P* = 0.0001	R = 0.404; *P* = 0.0001	R = 0.4274; *P* = 0.0001	R = 0.3901; *P* = 0.0001
O	Posttranslational modification, protein turnover, chaperones	Oral	27	Soil	82	R = 0.814; *P* = 0.0001	R = 0.7852; *P* = 0.0001	R = 0.8266; *P* = 0.0001	R = 0.7991; *P* = 0.0001
O	Posttranslational modification, protein turnover,	Oral	27	wastewater	41	R = 0.2927; *P* = 0.0001	R = 0.2687; *P* = 0.0001	R = 0.2745; *P* = 0.0001	R = 0.2541; *P* = 0.0001

	chaperones								
O	Posttranslational modification, protein turnover, chaperones	Oral	27	water	68	R = 0.0819; *P* = 0.023	R = 0.1007; *P* = 0.0091	R = 0.0883; *P* = 0.0167	R = 0.0888; *P* = 0.0152
O	Posttranslational modification, protein turnover, chaperones	sediment	53	skin	15	R = 0.2873; *P* = 0.0007	R = 0.2644; *P* = 0.0015	R = 0.2705; *P* = 0.0011	R = 0.239; *P* = 0.0026
O	Posttranslational modification, protein turnover, chaperones	sediment	53	Soil	82	R = 0.3751; *P* = 0.0001	R = 0.3546; *P* = 0.0001	R = 0.3863; *P* = 0.0001	R = 0.3657; *P* = 0.0001
O	Posttranslational modification, protein turnover, chaperones	sediment	53	wastewater	41	R = 0.1168; *P* = 0.0004	R = 0.1051; *P* = 0.0009	R = 0.1013; *P* = 0.0006	R = 0.0939; *P* = 0.0014
O	Posttranslational	sediment	53	water	68	R = 0.4217;	R = 0.4418;	R = 0.4267;	R = 0.4342;

	modification, protein turnover, chaperones					*P* = 0.0001	*P* = 0.0001	*P* = 0.0001	*P* = 0.0001
O	Posttranslational modification, protein turnover, chaperones	skin	15	Soil	82	R = 0.5252; *P* = 0.0001	R = 0.4785; *P* = 0.0001	R = 0.5492; *P* = 0.0001	R = 0.4955; *P* = 0.0001
O	Posttranslational modification, protein turnover, chaperones	skin	15	wastewater	41	R = 0.2282; *P* = 0.002	R = 0.2487; *P* = 0.002	R = 0.208; *P* = 0.0036	R = 0.2146; *P* = 0.0036
O	Posttranslational modification, protein turnover, chaperones	skin	15	water	68	R = 0.3994; *P* = 0.0001	R = 0.419; *P* = 0.0001	R = 0.3932; *P* = 0.0001	R = 0.4034; *P* = 0.0001
O	Posttranslational modification, protein turnover,	Soil	82	wastewater	41	R = 0.5248; *P* = 0.0001	R = 0.511; *P* = 0.0001	R = 0.5355; *P* = 0.0001	R = 0.5219; *P* = 0.0001

	chaperones								
O	Posttranslational modification, protein turnover, chaperones	Soil	82	water	68	R = 0.745; *P* = 0.0001	R = 0.7571; *P* = 0.0001	R = 0.7527; *P* = 0.0001	R = 0.7536; *P* = 0.0001
O	Posttranslational modification, protein turnover, chaperones	wastewater	41	water	68	R = 0.3226; *P* = 0.0001	R = 0.3268; *P* = 0.0001	R = 0.3227; *P* = 0.0001	R = 0.3237; *P* = 0.0001
P	Inorganic ion transport and metabolism	Digestive system	122	Oral	27	R = 0.155; *P* = 0.0104	R = 0.2; *P* = 0.0023	R = 0.1513; *P* = 0.0126	R = 0.1591; *P* = 0.0081
P	Inorganic ion transport and metabolism	Digestive system	122	sediment	53	R = 0.2349; *P* = 0.0001	R = 0.165; *P* = 0.0002	R = 0.2427; *P* = 0.0001	R = 0.1671; *P* = 0.0001
P	Inorganic ion transport and metabolism	Digestive system	122	skin	15	R = 0.3806; *P* = 0.0002	R = 0.3091; *P* = 0.0013	R = 0.3775; *P* = 0.0002	R = 0.2948; *P* = 0.0018

P	Inorganic ion transport and metabolism	Digestive system	122	Soil	82	R = 0.6748; *P* = 0.0001	R = 0.5968; *P* = 0.0001	R = 0.672; *P* = 0.0001	R = 0.6142; *P* = 0.0001
P	Inorganic ion transport and metabolism	Digestive system	122	wastewater	41	R = 0.2163; *P* = 0.0002	R = 0.1534; *P* = 0.002	R = 0.2086; *P* = 0.0002	R = 0.1368; *P* = 0.0038
P	Inorganic ion transport and metabolism	Digestive system	122	water	67	R = 0.3833; *P* = 0.0001	R = 0.3787; *P* = 0.0001	R = 0.385; *P* = 0.0001	R = 0.3545; *P* = 0.0001
P	Inorganic ion transport and metabolism	Oral	27	sediment	53	R = 0.3859; *P* = 0.0001	R = 0.3708; *P* = 0.0001	R = 0.3783; *P* = 0.0001	R = 0.3512; *P* = 0.0001
P	Inorganic ion transport and metabolism	Oral	27	skin	15	R = 0.3427; *P* = 0.0001	R = 0.2737; *P* = 0.0002	R = 0.3168; *P* = 0.0003	R = 0.2568; *P* = 0.0007
P	Inorganic ion transport and metabolism	Oral	27	Soil	82	R = 0.8148; *P* = 0.0001	R = 0.7659; *P* = 0.0001	R = 0.819; *P* = 0.0001	R = 0.7769; *P* = 0.0001

P	Inorganic ion transport and metabolism	Oral	27	wastewater	41	R = 0.3051; *P* = 0.0001	R = 0.2474; *P* = 0.0001	R = 0.2815; *P* = 0.0001	R = 0.2364; *P* = 0.0001
P	Inorganic ion transport and metabolism	Oral	27	water	67	R = 0.0394; *P* = 0.1441	R = 0.0451; *P* = 0.124	R = 0.0441; *P* = 0.1198	R = 0.0346; *P* = 0.1709
P	Inorganic ion transport and metabolism	sediment	53	skin	15	R = 0.2781; *P* = 0.0007	R = 0.2682; *P* = 0.0011	R = 0.2654; *P* = 0.0012	R = 0.2477; *P* = 0.0023
P	Inorganic ion transport and metabolism	sediment	53	Soil	82	R = 0.3557; *P* = 0.0001	R = 0.3203; *P* = 0.0001	R = 0.3612; *P* = 0.0001	R = 0.3307; *P* = 0.0001
P	Inorganic ion transport and metabolism	sediment	53	wastewater	41	R = 0.1122; *P* = 0.0006	R = 0.0975; *P* = 0.0009	R = 0.0966; *P* = 0.0009	R = 0.0897; *P* = 0.0016
P	Inorganic ion transport and metabolism	sediment	53	water	67	R = 0.372; *P* = 0.0001	R = 0.3857; *P* = 0.0001	R = 0.3694; *P* = 0.0001	R = 0.3768; *P* = 0.0001

P	Inorganic ion transport and metabolism	skin	15	Soil	82	R = 0.5999; *P* = 0.0001	R = 0.5297; *P* = 0.0001	R = 0.6022; *P* = 0.0001	R = 0.5444; *P* = 0.0001
P	Inorganic ion transport and metabolism	skin	15	wastewater	41	R = 0.1809; *P* = 0.009	R = 0.1978; *P* = 0.0071	R = 0.1738; *P* = 0.0099	R = 0.1813; *P* = 0.0102
P	Inorganic ion transport and metabolism	skin	15	water	67	R = 0.2906; *P* = 0.0001	R = 0.3002; *P* = 0.0001	R = 0.2792; *P* = 0.0001	R = 0.2843; *P* = 0.0001
P	Inorganic ion transport and metabolism	Soil	82	wastewater	41	R = 0.5136; *P* = 0.0001	R = 0.4809; *P* = 0.0001	R = 0.5101; *P* = 0.0001	R = 0.4898; *P* = 0.0001
P	Inorganic ion transport and metabolism	Soil	82	water	67	R = 0.7127; *P* = 0.0001	R = 0.7238; *P* = 0.0001	R = 0.7159; *P* = 0.0001	R = 0.7186; *P* = 0.0001
P	Inorganic ion transport and metabolism	wastewater	41	water	67	R = 0.2878; *P* = 0.0001	R = 0.2722; *P* = 0.0001	R = 0.2732; *P* = 0.0001	R = 0.2681; *P* = 0.0001

Q	Secondary metabolites biosynthesis, transport and catabolism	Digestive system	108	Oral	27	R = 0.2126; *P* = 0.0006	R = 0.183; *P* = 0.0018	R = 0.2201; *P* = 0.0006	R = 0.162; *P* = 0.005
Q	Secondary metabolites biosynthesis, transport and catabolism	Digestive system	108	sediment	50	R = 0.4261; *P* = 0.0001	R = 0.3348; *P* = 0.0001	R = 0.4225; *P* = 0.0001	R = 0.3353; *P* = 0.0001
Q	Secondary metabolites biosynthesis, transport and catabolism	Digestive system	108	skin	13	R = 0.6112; *P* = 0.0001	R = 0.534; *P* = 0.0001	R = 0.6039; *P* = 0.0001	R = 0.5185; *P* = 0.0001
Q	Secondary metabolites biosynthesis,	Digestive system	108	Soil	82	R = 0.7661; *P* = 0.0001	R = 0.7067; *P* = 0.0001	R = 0.761; *P* = 0.0001	R = 0.7138; *P* = 0.0001

	transport and catabolism								
Q	Secondary metabolites biosynthesis, transport and catabolism	Digestive system	108	wastewater	40	R = 0.4576; *P* = 0.0001	R = 0.3418; *P* = 0.0001	R = 0.4392; *P* = 0.0001	R = 0.3311; *P* = 0.0001
Q	Secondary metabolites biosynthesis, transport and catabolism	Digestive system	108	water	61	R = 0.483; *P* = 0.0001	R = 0.4601; *P* = 0.0001	R = 0.4836; *P* = 0.0001	R = 0.431; *P* = 0.0001
Q	Secondary metabolites biosynthesis, transport and catabolism	Oral	27	sediment	50	R = 0.3839; *P* = 0.0001	R = 0.3461; *P* = 0.0001	R = 0.384; *P* = 0.0001	R = 0.349; *P* = 0.0001
Q	Secondary	Oral	27	skin	13	R = 0.4067;	R = 0.3801;	R = 0.3813;	R = 0.3527;

	metabolites biosynthesis, transport and catabolism					*P* = 0.0001	*P* = 0.0002	*P* = 0.0001	*P* = 0.0002
Q	Secondary metabolites biosynthesis, transport and catabolism	Oral	27	Soil	82	R = 0.8014*;* *P* = 0.0001	R = 0.7608*;* *P* = 0.0001	R = 0.8046*;* *P* = 0.0001	R = 0.7718*;* *P* = 0.0001
Q	Secondary metabolites biosynthesis, transport and catabolism	Oral	27	wastewater	40	R = 0.3974*;* *P* = 0.0001	R = 0.3445*;* *P* = 0.0001	R = 0.3771*;* *P* = 0.0001	R = 0.3357*;* *P* = 0.0001
Q	Secondary metabolites biosynthesis, transport and	Oral	27	water	61	R = 0.073*;* *P* = 0.0361	R = 0.0575*;* *P* = 0.068	R = 0.0745*;* *P* = 0.0339	R = 0.0487*;* *P* = 0.0963

	catabolism								
Q	Secondary metabolites biosynthesis, transport and catabolism	sediment	50	skin	13	R = 0.3139; *P* = 0.0008	R = 0.2747; *P* = 0.0023	R = 0.2981; *P* = 0.0011	R = 0.262; *P* = 0.0033
Q	Secondary metabolites biosynthesis, transport and catabolism	sediment	50	Soil	82	R = 0.2942; *P* = 0.0001	R = 0.2814; *P* = 0.0001	R = 0.3008; *P* = 0.0001	R = 0.2875; *P* = 0.0001
Q	Secondary metabolites biosynthesis, transport and catabolism	sediment	50	wastewater	40	R = 0.1348; *P* = 0.0004	R = 0.0962; *P* = 0.0018	R = 0.114; *P* = 0.0005	R = 0.0917; *P* = 0.0028
Q	Secondary metabolites	sediment	50	water	61	R = 0.3813; *P* = 0.0001	R = 0.3951; *P* = 0.0001	R = 0.3869; *P* = 0.0001	R = 0.3881; *P* = 0.0001

	biosynthesis, transport and catabolism								
Q	Secondary metabolites biosynthesis, transport and catabolism	skin	13	Soil	82	R = 0.4971; *P* = 0.0001	R = 0.4401; *P* = 0.0001	R = 0.4922; *P* = 0.0001	R = 0.4491; *P* = 0.0001
Q	Secondary metabolites biosynthesis, transport and catabolism	skin	13	wastewater	40	R = 0.2434; *P* = 0.0037	R = 0.2655; *P* = 0.0025	R = 0.2249; *P* = 0.0078	R = 0.2395; *P* = 0.0045
Q	Secondary metabolites biosynthesis, transport and catabolism	skin	13	water	61	R = 0.3175; *P* = 0.0001	R = 0.3314; *P* = 0.0002	R = 0.3086; *P* = 0.0001	R = 0.3107; *P* = 0.0002

Q	Secondary metabolites biosynthesis, transport and catabolism	Soil	82	wastewater	40	R = 0.4624; P = 0.0001	R = 0.4307; P = 0.0001	R = 0.446; P = 0.0001	R = 0.4308; P = 0.0001
Q	Secondary metabolites biosynthesis, transport and catabolism	Soil	82	water	61	R = 0.6754; P = 0.0001	R = 0.6933; P = 0.0001	R = 0.6799; P = 0.0001	R = 0.6859; P = 0.0001
Q	Secondary metabolites biosynthesis, transport and catabolism	wastewater	40	water	61	R = 0.3493; P = 0.0001	R = 0.3515; P = 0.0001	R = 0.3423; P = 0.0001	R = 0.3443; P = 0.0001
R	General function prediction only	Digestive system	122	Oral	27	R = 0.1947; P = 0.0025	R = 0.2166; P = 0.0011	R = 0.1919; P = 0.0032	R = 0.1817; P = 0.0038
R	General function	Digestive	122	sediment	53	R = 0.2555;	R = 0.1715;	R = 0.2547;	R = 0.1687;

	prediction only	system				*P* = 0.0001	*P* = 0.0002	*P* = 0.0001	*P* = 0.0001
R	General function prediction only	Digestive system	122	skin	15	R = 0.4744; *P* = 0.0001	R = 0.3803; *P* = 0.0002	R = 0.4634; *P* = 0.0003	R = 0.3645; *P* = 0.0003
R	General function prediction only	Digestive system	122	Soil	82	R = 0.6991; *P* = 0.0001	R = 0.6162; *P* = 0.0001	R = 0.6915; *P* = 0.0001	R = 0.6302; *P* = 0.0001
R	General function prediction only	Digestive system	122	wastewater	44	R = 0.2723; *P* = 0.0001	R = 0.1966; *P* = 0.0002	R = 0.26; *P* = 0.0001	R = 0.1747; *P* = 0.0001
R	General function prediction only	Digestive system	122	water	68	R = 0.4241; *P* = 0.0001	R = 0.4151; *P* = 0.0001	R = 0.4279; *P* = 0.0001	R = 0.3932; *P* = 0.0001
R	General function prediction only	Oral	27	sediment	53	R = 0.3664; *P* = 0.0001	R = 0.3547; *P* = 0.0001	R = 0.3637; *P* = 0.0001	R = 0.3452; *P* = 0.0001
R	General function prediction only	Oral	27	skin	15	R = 0.4409; *P* = 0.0001	R = 0.3686; *P* = 0.0001	R = 0.4098; *P* = 0.0001	R = 0.3501; *P* = 0.0001
R	General function prediction only	Oral	27	Soil	82	R = 0.7971; *P* = 0.0001	R = 0.7612; *P* = 0.0001	R = 0.8051; *P* = 0.0001	R = 0.7759; *P* = 0.0001
R	General function prediction only	Oral	27	wastewater	44	R = 0.3288; *P* = 0.0001	R = 0.3021; *P* = 0.0001	R = 0.3192; *P* = 0.0001	R = 0.298; *P* = 0.0001
R	General function	Oral	27	water	68	R = 0.0187;	R = 0.0277;	R = 0.0344;	R = 0.0217;

	prediction only					*P* = 0.2853	*P* = 0.2183	*P* = 0.1756	*P* = 0.2557
R	General function prediction only	sediment	53	skin	15	R = 0.2732; *P* = 0.001	R = 0.2512; *P* = 0.0012	R = 0.2621; *P* = 0.0005	R = 0.2269; *P* = 0.003
R	General function prediction only	sediment	53	Soil	82	R = 0.3637; *P* = 0.0001	R = 0.3312; *P* = 0.0001	R = 0.3701; *P* = 0.0001	R = 0.3442; *P* = 0.0001
R	General function prediction only	sediment	53	wastewater	44	R = 0.0969; *P* = 0.0004	R = 0.0766; *P* = 0.0031	R = 0.0799; *P* = 0.0021	R = 0.0678; *P* = 0.0068
R	General function prediction only	sediment	53	water	68	R = 0.3396; *P* = 0.0001	R = 0.3646; *P* = 0.0001	R = 0.349; *P* = 0.0001	R = 0.358; *P* = 0.0001
R	General function prediction only	skin	15	Soil	82	R = 0.4846; *P* = 0.0001	R = 0.4559; *P* = 0.0001	R = 0.5041; *P* = 0.0001	R = 0.4694; *P* = 0.0001
R	General function prediction only	skin	15	wastewater	44	R = 0.1794; *P* = 0.0094	R = 0.2033; *P* = 0.0048	R = 0.1748; *P* = 0.013	R = 0.1819; *P* = 0.01
R	General function prediction only	skin	15	water	68	R = 0.3271; *P* = 0.0002	R = 0.3443; *P* = 0.0001	R = 0.3269; *P* = 0.0001	R = 0.3299; *P* = 0.0001
R	General function prediction only	Soil	82	wastewater	44	R = 0.4658; *P* = 0.0001	R = 0.4473; *P* = 0.0001	R = 0.4654; *P* = 0.0001	R = 0.4568; *P* = 0.0001
R	General function	Soil	82	water	68	R = 0.685;	R = 0.7026;	R = 0.6943;	R = 0.699;

	prediction only					*P* = 0.0001	*P* = 0.0001	*P* = 0.0001	*P* = 0.0001
R	General function prediction only	wastewater	44	water	68	R = 0.2912; *P* = 0.0001	R = 0.3047; *P* = 0.0001	R = 0.2934; *P* = 0.0001	R = 0.3006; *P* = 0.0001
S	Function unknown	Digestive system	111	Oral	27	R = 0.3013; *P* = 0.0001	R = 0.3357; *P* = 0.0001	R = 0.3032; *P* = 0.0001	R = 0.2929; *P* = 0.0001
S	Function unknown	Digestive system	111	sediment	50	R = 0.3002; *P* = 0.0001	R = 0.2118; *P* = 0.0001	R = 0.3064; *P* = 0.0001	R = 0.207; *P* = 0.0001
S	Function unknown	Digestive system	111	skin	14	R = 0.5474; *P* = 0.0001	R = 0.4748; *P* = 0.0001	R = 0.5388; *P* = 0.0001	R = 0.453; *P* = 0.0001
S	Function unknown	Digestive system	111	Soil	82	R = 0.7489; *P* = 0.0001	R = 0.6676; *P* = 0.0001	R = 0.7428; *P* = 0.0001	R = 0.6778; *P* = 0.0001
S	Function unknown	Digestive system	111	wastewater	45	R = 0.3284; *P* = 0.0001	R = 0.2489; *P* = 0.0001	R = 0.3194; *P* = 0.0001	R = 0.2217; *P* = 0.0001
S	Function unknown	Digestive system	111	water	67	R = 0.4873; *P* = 0.0001	R = 0.4696; *P* = 0.0001	R = 0.4941; *P* = 0.0001	R = 0.446; *P* = 0.0001
S	Function unknown	Oral	27	sediment	50	R = 0.3942; *P* = 0.0001	R = 0.3692; *P* = 0.0001	R = 0.3836; *P* = 0.0001	R = 0.3581; *P* = 0.0001
S	Function	Oral	27	skin	14	R = 0.4833;	R = 0.4179;	R = 0.4487;	R = 0.3993;

	unknown					P = 0.0001	P = 0.0001	P = 0.0001	P = 0.0001
S	Function unknown	Oral	27	Soil	82	R = 0.849; P = 0.0001	R = 0.8141; P = 0.0001	R = 0.8509; P = 0.0001	R = 0.8248; P = 0.0001
S	Function unknown	Oral	27	wastewater	45	R = 0.3254; P = 0.0001	R = 0.3089; P = 0.0001	R = 0.3189; P = 0.0001	R = 0.3057; P = 0.0001
S	Function unknown	Oral	27	water	67	R = 0.0205; P = 0.277	R = 0.0128; P = 0.3296	R = 0.0332; P = 0.1762	R = 0.0068; P = 0.3941
S	Function unknown	sediment	50	skin	14	R = 0.2792; P = 0.0006	R = 0.2473; P = 0.0011	R = 0.2619; P = 0.0007	R = 0.2277; P = 0.002
S	Function unknown	sediment	50	Soil	82	R = 0.3872; P = 0.0001	R = 0.3524; P = 0.0001	R = 0.3926; P = 0.0001	R = 0.3665; P = 0.0001
S	Function unknown	sediment	50	wastewater	45	R = 0.086; P = 0.0022	R = 0.0637; P = 0.0064	R = 0.0731; P = 0.003	R = 0.0545; P = 0.011
S	Function unknown	sediment	50	water	67	R = 0.3282; P = 0.0001	R = 0.3545; P = 0.0001	R = 0.3376; P = 0.0001	R = 0.3466; P = 0.0001
S	Function unknown	skin	14	Soil	82	R = 0.5254; P = 0.0001	R = 0.4707; P = 0.0001	R = 0.5341; P = 0.0001	R = 0.4847; P = 0.0001
S	Function	skin	14	wastewater	45	R = 0.1691;	R = 0.2054;	R = 0.172;	R = 0.1915;

	unknown					*P* = 0.0202	*P* = 0.0067	*P* = 0.0166	*P* = 0.0093
S	Function unknown	skin	14	water	67	R = 0.3329; *P* = 0.0001	R = 0.3583; *P* = 0.0001	R = 0.3362; *P* = 0.0001	R = 0.3445; *P* = 0.0001
S	Function unknown	Soil	82	wastewater	45	R = 0.5156; *P* = 0.0001	R = 0.4982; *P* = 0.0001	R = 0.5163; *P* = 0.0001	R = 0.5068; *P* = 0.0001
S	Function unknown	Soil	82	water	67	R = 0.6997; *P* = 0.0001	R = 0.7209; *P* = 0.0001	R = 0.709; *P* = 0.0001	R = 0.7165; *P* = 0.0001
S	Function unknown	wastewater	45	water	67	R = 0.2874; *P* = 0.0001	R = 0.3064; *P* = 0.0001	R = 0.2932; *P* = 0.0001	R = 0.3012; *P* = 0.0001
T	Signal transduction mechanisms	Digestive system	121	Oral	27	R = 0.2082; *P* = 0.001	R = 0.1779; *P* = 0.005	R = 0.2015; *P* = 0.0014	R = 0.158; *P* = 0.0075
T	Signal transduction mechanisms	Digestive system	121	sediment	53	R = 0.2945; *P* = 0.0001	R = 0.1938; *P* = 0.0001	R = 0.2934; *P* = 0.0001	R = 0.1952; *P* = 0.0001
T	Signal transduction mechanisms	Digestive system	121	skin	14	R = 0.5588; *P* = 0.0001	R = 0.3941; *P* = 0.0002	R = 0.5356; *P* = 0.0001	R = 0.3874; *P* = 0.0001

T	Signal transduction mechanisms	Digestive system	121	Soil	82	R = 0.728; P = 0.0001	R = 0.6282; P = 0.0001	R = 0.7176; P = 0.0001	R = 0.6442; P = 0.0001
T	Signal transduction mechanisms	Digestive system	121	wastewater	43	R = 0.3606; P = 0.0001	R = 0.2222; P = 0.0001	R = 0.3322; P = 0.0001	R = 0.2145; P = 0.0001
T	Signal transduction mechanisms	Digestive system	121	water	68	R = 0.3909; P = 0.0001	R = 0.3711; P = 0.0001	R = 0.3897; P = 0.0001	R = 0.3459; P = 0.0001
T	Signal transduction mechanisms	Oral	27	sediment	53	R = 0.3034; P = 0.0001	R = 0.2938; P = 0.0001	R = 0.3048; P = 0.0001	R = 0.2873; P = 0.0001
T	Signal transduction mechanisms	Oral	27	skin	14	R = 0.3591; P = 0.0003	R = 0.2859; P = 0.0004	R = 0.3264; P = 0.0002	R = 0.2626; P = 0.0014
T	Signal transduction mechanisms	Oral	27	Soil	82	R = 0.7108; P = 0.0001	R = 0.6832; P = 0.0001	R = 0.7236; P = 0.0001	R = 0.693; P = 0.0001

T	Signal transduction mechanisms	Oral	27	wastewater	43	R = 0.3303; *P* = 0.0001	R = 0.3053; *P* = 0.0001	R = 0.3252; *P* = 0.0001	R = 0.3061; *P* = 0.0001
T	Signal transduction mechanisms	Oral	27	water	68	R = 0.0224; *P* = 0.261	R = 0.0357; *P* = 0.169	R = 0.0358; *P* = 0.1584	R = 0.0326; *P* = 0.1809
T	Signal transduction mechanisms	sediment	53	skin	14	R = 0.3408; *P* = 0.0001	R = 0.2603; *P* = 0.0011	R = 0.3175; *P* = 0.0004	R = 0.2439; *P* = 0.0023
T	Signal transduction mechanisms	sediment	53	Soil	82	R = 0.3409; *P* = 0.0001	R = 0.3091; *P* = 0.0001	R = 0.342; *P* = 0.0001	R = 0.3178; *P* = 0.0001
T	Signal transduction mechanisms	sediment	53	wastewater	43	R = 0.1143; *P* = 0.0002	R = 0.0614; *P* = 0.0089	R = 0.0942; *P* = 0.0008	R = 0.0519; *P* = 0.0166
T	Signal transduction mechanisms	sediment	53	water	68	R = 0.3316; *P* = 0.0001	R = 0.3484; *P* = 0.0001	R = 0.3399; *P* = 0.0001	R = 0.3443; *P* = 0.0001

T	Signal transduction mechanisms	skin	14	Soil	82	R = 0.4534; *P* = 0.0001	R = 0.4263; *P* = 0.0001	R = 0.4641; *P* = 0.0001	R = 0.4328; *P* = 0.0001
T	Signal transduction mechanisms	skin	14	wastewater	43	R = 0.1899; *P* = 0.0127	R = 0.2197; *P* = 0.0059	R = 0.1936; *P* = 0.0114	R = 0.2063; *P* = 0.0063
T	Signal transduction mechanisms	skin	14	water	68	R = 0.3578; *P* = 0.0001	R = 0.3474; *P* = 0.0001	R = 0.3464; *P* = 0.0001	R = 0.3356; *P* = 0.0001
T	Signal transduction mechanisms	Soil	82	wastewater	43	R = 0.4034; *P* = 0.0001	R = 0.3931; *P* = 0.0001	R = 0.4052; *P* = 0.0001	R = 0.3948; *P* = 0.0001
T	Signal transduction mechanisms	Soil	82	water	68	R = 0.6717; *P* = 0.0001	R = 0.6788; *P* = 0.0001	R = 0.6791; *P* = 0.0001	R = 0.679; *P* = 0.0001
T	Signal transduction mechanisms	wastewater	43	water	68	R = 0.3203; *P* = 0.0001	R = 0.3192; *P* = 0.0001	R = 0.3137; *P* = 0.0001	R = 0.3175; *P* = 0.0001

U	Intracellular trafficking, secretion, and vesicular transport	Digestive system	109	Oral	27	R = 0.3593; P = 0.0001	R = 0.3945; P = 0.0001	R = 0.3648; P = 0.0001	R = 0.3564; P = 0.0001
U	Intracellular trafficking, secretion, and vesicular transport	Digestive system	109	sediment	50	R = 0.4132; P = 0.0001	R = 0.2622; P = 0.0001	R = 0.413; P = 0.0001	R = 0.2566; P = 0.0001
U	Intracellular trafficking, secretion, and vesicular transport	Digestive system	109	skin	13	R = 0.5213; P = 0.0001	R = 0.3608; P = 0.0004	R = 0.5035; P = 0.0001	R = 0.3462; P = 0.0003
U	Intracellular trafficking, secretion, and vesicular transport	Digestive system	109	Soil	82	R = 0.8071; P = 0.0001	R = 0.6969; P = 0.0001	R = 0.8037; P = 0.0001	R = 0.709; P = 0.0001
U	Intracellular trafficking,	Digestive system	109	wastewater	40	R = 0.3776; P = 0.0001	R = 0.245; P = 0.0001	R = 0.3719; P = 0.0001	R = 0.2225; P = 0.0001

	secretion, and vesicular transport								
U	Intracellular trafficking, secretion, and vesicular transport	Digestive system	109	water	62	R = 0.5738; *P* = 0.0001	R = 0.5406; *P* = 0.0001	R = 0.5721; *P* = 0.0001	R = 0.5163; *P* = 0.0001
U	Intracellular trafficking, secretion, and vesicular transport	Oral	27	sediment	50	R = 0.5475; *P* = 0.0001	R = 0.5153; *P* = 0.0001	R = 0.5388; *P* = 0.0001	R = 0.509; *P* = 0.0001
U	Intracellular trafficking, secretion, and vesicular transport	Oral	27	skin	13	R = 0.4716; *P* = 0.0001	R = 0.3845; *P* = 0.0001	R = 0.4308; *P* = 0.0001	R = 0.3662; *P* = 0.0001
U	Intracellular trafficking, secretion, and vesicular transport	Oral	27	Soil	82	R = 0.876; *P* = 0.0001	R = 0.8463; *P* = 0.0001	R = 0.8788; *P* = 0.0001	R = 0.8564; *P* = 0.0001

U	Intracellular trafficking, secretion, and vesicular transport	Oral	27	wastewater	40	R = 0.3998; *P* = 0.0001	R = 0.358; *P* = 0.0001	R = 0.3817; *P* = 0.0001	R = 0.3488; *P* = 0.0001
U	Intracellular trafficking, secretion, and vesicular transport	Oral	27	water	62	R = 0.0611; *P* = 0.0596	R = 0.0555; *P* = 0.0733	R = 0.0585; *P* = 0.0651	R = 0.0398; *P* = 0.1337
U	Intracellular trafficking, secretion, and vesicular transport	sediment	50	skin	13	R = 0.2661; *P* = 0.0021	R = 0.2375; *P* = 0.006	R = 0.2488; *P* = 0.004	R = 0.2236; *P* = 0.0083
U	Intracellular trafficking, secretion, and vesicular transport	sediment	50	Soil	82	R = 0.3685; *P* = 0.0001	R = 0.3349; *P* = 0.0001	R = 0.3692; *P* = 0.0001	R = 0.3466; *P* = 0.0001
U	Intracellular trafficking,	sediment	50	wastewater	40	R = 0.1091; *P* = 0.0004	R = 0.0934; *P* = 0.0012	R = 0.099; *P* = 0.0018	R = 0.089; *P* = 0.0021

	secretion, and vesicular transport								
U	Intracellular trafficking, secretion, and vesicular transport	sediment	50	water	62	R = 0.4301; *P* = 0.0001	R = 0.4479; *P* = 0.0001	R = 0.4336; *P* = 0.0001	R = 0.4392; *P* = 0.0001
U	Intracellular trafficking, secretion, and vesicular transport	skin	13	Soil	82	R = 0.607; *P* = 0.0001	R = 0.5606; *P* = 0.0001	R = 0.6223; *P* = 0.0001	R = 0.5809; *P* = 0.0001
U	Intracellular trafficking, secretion, and vesicular transport	skin	13	wastewater	40	R = 0.1452; *P* = 0.0356	R = 0.1643; *P* = 0.022	R = 0.1337; *P* = 0.0427	R = 0.1411; *P* = 0.0378
U	Intracellular trafficking, secretion, and vesicular transport	skin	13	water	62	R = 0.2993; *P* = 0.0001	R = 0.305; *P* = 0.0001	R = 0.291; *P* = 0.0001	R = 0.2883; *P* = 0.0001

U	Intracellular trafficking, secretion, and vesicular transport	Soil	82	wastewater	40	R = 0.5229; *P* = 0.0001	R = 0.5058; *P* = 0.0001	R = 0.5291; *P* = 0.0001	R = 0.5184; *P* = 0.0001
U	Intracellular trafficking, secretion, and vesicular transport	Soil	82	water	62	R = 0.7571; *P* = 0.0001	R = 0.7671; *P* = 0.0001	R = 0.7611; *P* = 0.0001	R = 0.7622; *P* = 0.0001
U	Intracellular trafficking, secretion, and vesicular transport	wastewater	40	water	62	R = 0.3159; *P* = 0.0001	R = 0.3176; *P* = 0.0001	R = 0.3151; *P* = 0.0001	R = 0.3129; *P* = 0.0001
V	Defense mechanisms	Digestive system	120	Oral	27	R = 0.2197; *P* = 0.0007	R = 0.227; *P* = 0.0008	R = 0.2156; *P* = 0.0011	R = 0.195; *P* = 0.0014
V	Defense mechanisms	Digestive system	120	sediment	51	R = 0.287; *P* = 0.0001	R = 0.2237; *P* = 0.0001	R = 0.2957; *P* = 0.0001	R = 0.2196; *P* = 0.0001
V	Defense mechanisms	Digestive system	120	skin	14	R = 0.426; *P* = 0.0001	R = 0.3527; *P* = 0.0002	R = 0.4261; *P* = 0.0001	R = 0.3321; *P* = 0.0004

V	Defense mechanisms	Digestive system	120	Soil	82	R = 0.6988; *P* = 0.0001	R = 0.6422; *P* = 0.0001	R = 0.6979; *P* = 0.0001	R = 0.6533; *P* = 0.0001
V	Defense mechanisms	Digestive system	120	wastewater	43	R = 0.2221; *P* = 0.0001	R = 0.184; *P* = 0.0001	R = 0.2169; *P* = 0.0001	R = 0.153; *P* = 0.001
V	Defense mechanisms	Digestive system	120	water	66	R = 0.4113; *P* = 0.0001	R = 0.4005; *P* = 0.0001	R = 0.4128; *P* = 0.0001	R = 0.3717; *P* = 0.0001
V	Defense mechanisms	Oral	27	sediment	51	R = 0.4364; *P* = 0.0001	R = 0.4144; *P* = 0.0001	R = 0.4263; *P* = 0.0001	R = 0.4044; *P* = 0.0001
V	Defense mechanisms	Oral	27	skin	14	R = 0.4601; *P* = 0.0001	R = 0.4154; *P* = 0.0001	R = 0.4299; *P* = 0.0001	R = 0.3886; *P* = 0.0001
V	Defense mechanisms	Oral	27	Soil	82	R = 0.8186; *P* = 0.0001	R = 0.7959; *P* = 0.0001	R = 0.8247; *P* = 0.0001	R = 0.8042; *P* = 0.0001
V	Defense mechanisms	Oral	27	wastewater	43	R = 0.2916; *P* = 0.0001	R = 0.2943; *P* = 0.0001	R = 0.2772; *P* = 0.0001	R = 0.2833; *P* = 0.0001
V	Defense mechanisms	Oral	27	water	66	R = -0.0128; *P* = 0.587	R = 0.0102; *P* = 0.364	R = -0.0009; *P* = 0.4608	R = 0.0013; *P* = 0.4312
V	Defense mechanisms	sediment	51	skin	14	R = 0.2055; *P* = 0.007	R = 0.1749; *P* = 0.0116	R = 0.1926; *P* = 0.0075	R = 0.1549; *P* = 0.0196

V	Defense mechanisms	sediment	51	Soil	82	R = 0.3089; *P* = 0.0001	R = 0.2818; *P* = 0.0001	R = 0.3092; *P* = 0.0001	R = 0.292; *P* = 0.0001
V	Defense mechanisms	sediment	51	wastewater	43	R = 0.1186; *P* = 0.0005	R = 0.1043; *P* = 0.0007	R = 0.1043; *P* = 0.001	R = 0.0955; *P* = 0.0021
V	Defense mechanisms	sediment	51	water	66	R = 0.3617; *P* = 0.0001	R = 0.3841; *P* = 0.0001	R = 0.3676; *P* = 0.0001	R = 0.377; *P* = 0.0001
V	Defense mechanisms	skin	14	Soil	82	R = 0.5055; *P* = 0.0001	R = 0.4714; *P* = 0.0001	R = 0.5144; *P* = 0.0001	R = 0.4779; *P* = 0.0001
V	Defense mechanisms	skin	14	wastewater	43	R = 0.1637; *P* = 0.0194	R = 0.1837; *P* = 0.0106	R = 0.1534; *P* = 0.0211	R = 0.161; *P* = 0.018
V	Defense mechanisms	skin	14	water	66	R = 0.3009; *P* = 0.0001	R = 0.3346; *P* = 0.0001	R = 0.2996; *P* = 0.0003	R = 0.3199; *P* = 0.0001
V	Defense mechanisms	Soil	82	wastewater	43	R = 0.5344; *P* = 0.0001	R = 0.5105; *P* = 0.0001	R = 0.5274; *P* = 0.0001	R = 0.5199; *P* = 0.0001
V	Defense mechanisms	Soil	82	water	66	R = 0.7084; *P* = 0.0001	R = 0.722; *P* = 0.0001	R = 0.7135; *P* = 0.0001	R = 0.7186; *P* = 0.0001
V	Defense mechanisms	wastewater	43	water	66	R = 0.2381; *P* = 0.0001	R = 0.2587; *P* = 0.0001	R = 0.2407; *P* = 0.0001	R = 0.2522; *P* = 0.0001

X	Mobilome: prophages, transposons	Digestive system	114	Oral	27	R = 0.2341; *P* = 0.0007	R = 0.2535; *P* = 0.0002	R = 0.2259; *P* = 0.0004	R = 0.2171; *P* = 0.0005
X	Mobilome: prophages, transposons	Digestive system	114	sediment	49	R = 0.3805; *P* = 0.0001	R = 0.2515; *P* = 0.0001	R = 0.3896; *P* = 0.0001	R = 0.2557; *P* = 0.0001
X	Mobilome: prophages, transposons	Digestive system	114	skin	13	R = 0.4638; *P* = 0.0001	R = 0.3537; *P* = 0.0001	R = 0.4509; *P* = 0.0001	R = 0.3282; *P* = 0.0008
X	Mobilome: prophages, transposons	Digestive system	114	Soil	82	R = 0.8289; *P* = 0.0001	R = 0.7481; *P* = 0.0001	R = 0.8212; *P* = 0.0001	R = 0.7622; *P* = 0.0001
X	Mobilome: prophages, transposons	Digestive system	114	wastewater	41	R = 0.3139; *P* = 0.0001	R = 0.2349; *P* = 0.0001	R = 0.3099; *P* = 0.0001	R = 0.2197; *P* = 0.0002
X	Mobilome: prophages, transposons	Digestive system	114	water	66	R = 0.382; *P* = 0.0001	R = 0.3097; *P* = 0.0001	R = 0.3815; *P* = 0.0001	R = 0.2874; *P* = 0.0001

X	Mobilome: prophages, transposons	Oral	27	sediment	49	R = 0.4682; *P* = 0.0001	R = 0.4488; *P* = 0.0001	R = 0.458; *P* = 0.0001	R = 0.4287; *P* = 0.0001
X	Mobilome: prophages, transposons	Oral	27	skin	13	R = 0.3263; *P* = 0.0001	R = 0.243; *P* = 0.0021	R = 0.3002; *P* = 0.0003	R = 0.2185; *P* = 0.0034
X	Mobilome: prophages, transposons	Oral	27	Soil	82	R = 0.8988; *P* = 0.0001	R = 0.8705; *P* = 0.0001	R = 0.8976; *P* = 0.0001	R = 0.8719; *P* = 0.0001
X	Mobilome: prophages, transposons	Oral	27	wastewater	41	R = 0.2756; *P* = 0.0001	R = 0.2532; *P* = 0.0001	R = 0.264; *P* = 0.0001	R = 0.2477; *P* = 0.0001
X	Mobilome: prophages, transposons	Oral	27	water	66	R = 0.0769; *P* = 0.038	R = 0.0077; *P* = 0.3773	R = 0.0367; *P* = 0.1686	R = -0.0062; *P* = 0.5332
X	Mobilome: prophages, transposons	sediment	49	skin	13	R = 0.2418; *P* = 0.0013	R = 0.2455; *P* = 0.0011	R = 0.2401; *P* = 0.0014	R = 0.2191; *P* = 0.0021

X	Mobilome: prophages, transposons	sediment	49	Soil	82	R = 0.4798; *P* = 0.0001	R = 0.4806; *P* = 0.0001	R = 0.478; *P* = 0.0001	R = 0.4772; *P* = 0.0001
X	Mobilome: prophages, transposons	sediment	49	wastewater	41	R = 0.0901; *P* = 0.0022	R = 0.0813; *P* = 0.0039	R = 0.0853; *P* = 0.0051	R = 0.0731; *P* = 0.0082
X	Mobilome: prophages, transposons	sediment	49	water	66	R = 0.2262; *P* = 0.0001	R = 0.2217; *P* = 0.0001	R = 0.2228; *P* = 0.0001	R = 0.2212; *P* = 0.0001
X	Mobilome: prophages, transposons	skin	13	Soil	82	R = 0.7078; *P* = 0.0001	R = 0.6672; *P* = 0.0001	R = 0.7193; *P* = 0.0001	R = 0.6739; *P* = 0.0001
X	Mobilome: prophages, transposons	skin	13	wastewater	41	R = 0.0948; *P* = 0.0703	R = 0.1063; *P* = 0.0546	R = 0.0981; *P* = 0.0686	R = 0.0942; *P* = 0.0716
X	Mobilome: prophages, transposons	skin	13	water	66	R = 0.1003; *P* = 0.0701	R = 0.0918; *P* = 0.083	R = 0.0774; *P* = 0.1137	R = 0.079; *P* = 0.106

X	Mobilome: prophages, transposons	Soil	82	wastewater	41	R = 0.5186; *P* = 0.0001	R = 0.5128; *P* = 0.0001	R = 0.5255; *P* = 0.0001	R = 0.5126; *P* = 0.0001
X	Mobilome: prophages, transposons	Soil	82	water	66	R = 0.7421; *P* = 0.0001	R = 0.7363; *P* = 0.0001	R = 0.7428; *P* = 0.0001	R = 0.7428; *P* = 0.0001
X	Mobilome: prophages, transposons	wastewater	41	water	66	R = 0.1486; *P* = 0.0001	R = 0.1384; *P* = 0.0003	R = 0.1375; *P* = 0.0004	R = 0.1369; *P* = 0.0002

Supplementary Table 14. Correlations between distances in the relative abundance of different taxonomic ranks and that of codon usage. Correlation values were calculated considering distances (in codon usage frequencies or relative abundance of different taxonomic ranks) among the test samples. Codon usage distances were calculated based on codon usage frequencies calculated by two approaches absCUFs and synCUFs method (see main text). Distances in the taxonomic abundance were calculated based on the relative abundance of different taxons grouped according to their taxonomic ranks. Here we considered taxons from four ranks namely phylum, order, family, and genus, and calculated distances considering frequencies of highly abundant taxons under each of these ranks separately. All the distances (either in codon usage or taxonomic

abundance) among the samples were calculated by the Euclidean distance and the Bray-Curtis dissimilarity methods and correlated separately. To access the strength of correlation, we calculated Spearman's rank correlation co-efficient (ρ) where significance levels were shown with *P*-values.

	**Spearman's correlation (ρ) with distances in absCUFs (distances calculated by Euclidean distance method)**	**Spearman's correlation (ρ) with distances in absCUFs (distances calculated by Bray-Curtis dissimilarity method)**	**Spearman's correlation (ρ) with distances in synCUFs (distances calculated by Euclidean distance method)**	**Spearman's correlation (ρ) with distances in synCUFs (distances calculated by Bray-Curtis dissimilarity method)**	**Spearman's correlation (ρ) with distances in amino acid frequencies (distances calculated by Euclidean distance method))**	**Spearman's correlation (ρ) with distances in amino acid frequencies (distances calculated by Bray-Curtis dissimilarity method)**
Family	ρ = 0.2485; *P* = 1 < 10-6	ρ = 0.4214; *P* = 1 < 10-6	ρ = 0.2415; *P* = 1 < 10-6	ρ = 0.3753; *P* = 1 < 10-6	ρ = 0.1948; *P* = 1 < 10-6	ρ = 0.4689; *P* = 1 < 10-6
Genus	ρ = 0.2574; *P* = 1 < 10-6	ρ = 0.4006; *P* = 1 < 10-6	ρ = 0.2526; *P* = 1 < 10-6	ρ = 0.3562; *P* = 1 < 10-6	ρ = 0.1928; *P* = 1 < 10-6	ρ = 0.4384; *P* = 1 < 10-6
Order	ρ = 0.3134;	ρ = 0.4546;	ρ = 0.2891;	ρ = 0.4032;	ρ = 0.3013;	ρ = 0.5218;

	P = 1 < 10-6	*P* = 1 < 10-6	*P* = 1 < 10-6	*P* = 1 < 10-6	*P* = 1 < 10-6	*P* = 1 < 10-6
Phylum	ρ = 0.3465; *P* = 1 < 10-6	ρ = 0.3581; *P* = 1 < 10-6	ρ = 0.3035; *P* = 1 < 10-6	ρ = 0.3015; *P* = 1 < 10-6	ρ = 0.4253; *P* = 1 < 10-6	ρ = 0.5021; *P* = 1 < 10-6

Supplementary Table 15. Partial correlations between the distances in amino acid frequencies and distances of all other variables. Partial correlations were conducted between two variables controlling the effects of all other variables where one variable is distances in amino acid usage frequencies and the other variable is either distances in GC content or distances in GO terms' abundance or distances in taxonomic abundance or k-mer frequencies. Distances in Gene Ontology (GO) terms' frequencies among the samples were calculated by considering the relative abundance of 500 GO biological process, 500 GO molecular function, and 100 GO cellular component terms separately. Taxonomic distances among the samples were calculated considering the relative abundance of taxons under four taxonomic ranks (order, phylum, family, and genus) separately. All the distances were calculated using Euclidean distance and Bray-Curtis dissimilarity methods and partial correlation analysis were done separately. Here ρ stands for correlation coefficient and significance levels were shown with *P*-values.

**Test variable**	**Control variables**	**Partial correlation with distances in amino acid frequency (distances calculated by Euclidean distance method)**	**Partial correlation with distances in amino acid frequency (distances calculated by Bray-Curtis dissimilarity**

			**method)**
absCUFs frequency	GO-CC & GO-MF & family & genus & order & phylum & GC-distance & k2-mer & k4-mer & k5-mer & k6-mer & k7-mer & k8-mer & k9-mer & k10-mer & GO-BP	ρ = -0.3; *P* = 0.00E+00	ρ =-0.394; *P* = 0.00E+00
GO-BP	absCUFs & GO-CC & GO-MF & family & genus & order & phylum & GC-distance & k2-mer & k4-mer & k5-mer & k6-mer & k7-mer & k8-mer & k9-mer & k10-mer	ρ = -0.099; *P* = 6.23E-191	ρ =-0.048; *P* = 1.31E-46
GO-CC	GO-MF & family & genus & order & phylum & GC-distance & k2-mer & k4-mer & k5-mer & k6-mer & k7-mer & k8-mer & k9 & k10 & GO-BP & absCUFs	ρ = 0.019; *P* = 7.02E-09	-----
GO-MF	family & genus & order & phylum & GC-distance & k2-mer & k4-mer & k5-mer & k6-mer & k7-mer & k8-mer & k9-mer & k10-mer & GO-BP & absCUFs & GO-CC	ρ = 0.258; *P* = 0.00E+00	ρ =0.049; *P* = 3.26E-49
family	genus & order & phylum & GC-distance & k2-mer & k4-mer & k5-mer & k6-mer & k7-mer & k8-mer & k9-mer & k10-mer & GO-BP & absCUFs & GO-CC & GO-MF	ρ = -0.102; *P* = 3.33E-204	ρ =-0.041; *P* = 6.68E-35
genus	order & phylum & GC-distance & k2-mer & k4-mer	ρ = 0.102;	ρ =0.048;

	& k5-mer & k6-mer & k7-mer & k8-mer & k9-mer & k10-mer & GO-BP & absCUFs & GO-CC & GO-MF & family	*P* = 5.97E-205	*P* = 2.40E-47
order	phylum & GC-distance & k2-mer & k4-mer & k5-mer & k6-mer & k7-mer & k8-mer & k9 & k10 & GO-BP & absCUFs & GO-CC & GO-MF & family & genus	ρ = 0.062*;* *P* = 2.43E-76	ρ =0.021*;* *P* = 1.64E-10
phylum	GC-distance & k2-mer & k4-mer & k5-mer & k6-mer & k7-mer & k8-mer & k9-mer & k10-mer & GO-BP & absCUFs & GO-CC & GO-MF & family & genus & order	ρ = 0.167*;* *P* = 0.00E+00	ρ =0.193*;* *P* = 0.00E+00
GC-distance	k2-mer & k4-mer & k5-mer & k6-mer & k7-mer & k8-mer & k9-mer & k10-mer & GO-BP & absCUFs & GO-CC & GO-MF & family & genus & order & phylum	ρ = 0.256*;* *P* = 0.00E+00	ρ =0.077*;* *P* = 5.68E-116
k2-mer	k4-mer & k5-mer & k6-mer & k7-mer & k8-mer & k9-mer & k10-mer & GO-BP & absCUFs & GO-CC & GO-MF & family & genus & order & phylum & GC-distance	ρ = 0.01*;* *P* = 3.68E-03	ρ =0.022*;* *P* = 2.02E-11
k4-mer	k5-mer & k6-mer & k7-mer & k8-mer & k9-mer & k10-mer & GO-BP & absCUFs & GO-CC & GO-MF & family & genus & order & phylum & GC-distance & k2-mer	ρ = -0.009*;* *P* = 9.07E-03	ρ =0.146*;* *P* = 0.00E+00
k5-mer	k6-mer & k7-mer & k8-mer & k9-mer & k10-mer & GO-BP & absCUFs & GO-CC & GO-MF & family &	ρ = 0.298*;* *P* = 0.00E+00	ρ =0.122*;* *P* = 5.18E-290

	genus & order & phylum & GC-distance & k2-mer & k4-mer		
k6-mer	k7-mer & k8-mer & k9-mer & k10-mer & GO-BP & absCUFs & GO-CC & GO-MF & family & genus & order & phylum & GC-distance & k2-mer & k4-mer & k5-mer	ρ = -0.149*;* *P* = 0.00E+00	ρ =-0.23*;* *P* = 0.00E+00
k7-mer	k8-mer & k9-mer & k10-mer & GO-BP & absCUFs & GO-CC & GO-MF & family & genus & order & phylum & GC-distance & k2-mer & k4-mer & k5-mer & k6-mer	ρ = -0.002*;* *P* = 5.70E-01	ρ =0.085*;* *P* = 7.05E-143
k8-mer	k9-mer & k10-mer & GO-BP & absCUFs & GO-CC & GO-MF & family & genus & order & phylum & GC-distance & k2-mer & k4-mer & k5-mer & k6-mer & k7-mer	ρ = 0.124*;* *P* = 2.72E-300	ρ =0.196*;* *P* = 0.00E+00
k9-mer	k10-mer & GO-BP & absCUFs & GO-CC & GO-MF & family & genus & order & phylum & GC-distance & k2-mer & k4-mer & k5-mer & k6-mer & k7-mer & k8-mer	ρ = -0.022*;* *P* = 7.99E-11	ρ =-0.015*;* *P* = 5.47E-06
k10-mer	GO-BP & absCUFs & GO-CC & GO-MF & family & genus & order & phylum & GC-distance & k2-mer & k4-mer & k5-mer & k6-mer & k7-mer & k8-mer & k9-mer	ρ = -0.152*;* *P* = 0.00E+00	ρ =0.013*;* *P* = 5.96E-05

Supplementary Table 16. Linear regression analysis to test the impact of other variables on codon usage distances. Distances were calculated by two methods (i) Euclidean distance and (ii) Bray-Curtis dissimilarity method and linear regression was conducted considering distances calculated by each method separately.

Test variable	Standardized β-coefficient for absCUFs based on Euclidean distance	Standardized β-coefficient for absCUFs based on Bray-Curtis dissimilarity
Amino Acid	β = -0.148; *P* = 0.00E+00	β = -0.181; *P* = 0.00E+00
GO-BP	β = 0.033; *P* = 1.73E-153	β = 0.318; *P* = 0.00E+00
GO-CC	β = 0.041; *P* = 0.00E+00	--------
GO-MF	β = 0.014; *P* = 7.36E-28	β = -0.314; *P* = 0.00E+00
Family	β = 0.012; *P* = 2.44E-07	β = 0.033; *P* = 1.06E-17

Genus	β = 0.046; *P* = 4.12E-200	β = -0.001; *P* = 8.10E-01
Order	β = 0.051; *P* = 1.64E-198	β = 0.023; *P* = 5.10E-28
Phylum	β = -0.04; *P* = 0.00E+00	β = -0.033; *P* = 1.57E-281
GC-distance	β = 0.494; *P* = 0.00E+00	β = 0.233; *P* = 0.00E+00
k2-mer	β = 0.018; *P* = 3.27E-158	β = 0.01; *P* = 1.71E-30
k4-mer	β = 0.159; *P* = 1.36E-196	β = 0.668; *P* = 0.00E+00
k5-mer	β = 0.916; *P* = 0.00E+00	β = 0.026; *P* = 1.03E-01
k6-mer	β = -0.543; *P* = 0.00E+00	β = 0.3; *P* = 7.38E-184
k7-mer	β = -0.003; *P* = 8.20E-04	β = -0.232; *P* = 0.00E+00

k8-mer	β = 0.109; *P* = 2.76E-126	β = 0.123; *P* = 2.55E-214
k9-mer	β = 0.02; *P* = 2.73E-10	β = 0.037; *P* = 1.18E-48
k10-mer	β = -0.06; *P* = 2.90E-108	β = -0.032; *P* = 4.94E-50

Supplementary Table 17. Linear regression analysis to test the impact of other variables on amino acid usage distances. Distances were calculated by two methods (i) Euclidean distance and (ii) Bray-Curtis dissimilarity method and linear regression was conducted considering distances calculated by each method separately.

(**Test variable**	**Standardized β-coefficient for Amino acid distances based on Euclidean distance**	**Standardized β-coefficient for Amino acid distances based on Bray-Curtis dissimilarity**
absCUFs	β = -0.609; *P* = 0.00E+00	β = -0.854; *P* = 0.00E+00
GO-BP	β = -0.075; *P* = 6.23E-191	β = -0.094; *P* = 1.31E-46

GO-CC	β = 0.009; *P* = 7.02E-09	β = 0; *P* = 0.00E+00
GO-MF	β = 0.194; *P* = 0.00E+00	β = 0.098; *P* = 3.26E-49
Family	β = -0.138; *P* = 3.33E-204	β = -0.104; *P* = 6.68E-35
Genus	β = 0.094; *P* = 5.97E-205	β = 0.09; *P* = 2.40E-47
Order	β = 0.064; *P* = 2.43E-76	β = 0.029; *P* = 1.64E-10
Phylum	β = 0.093; *P* = 0.00E+00	β = 0.114; *P* = 0.00E+00
GC-distance	β = 0.419; *P* = 0.00E+00	β = 0.127; *P* = 5.68E-116
k2-mer	β = 0.004; *P* = 3.68E-03	β = 0.013; *P* = 2.02E-11
k4-mer	β = -0.028; *P* = 9.07E-03	β = 1.093; *P* = 0.00E+00
k5-mer	β = 1.803; *P* = 0.00E+00	β = 1.257; *P* = 5.18E-290
k6-mer	β = -0.834; *P* = 0.00E+00	β = -1.549; *P* = 0.00E+00
k7-mer	β = -0.001; *P* = 5.70E-01	β = 0.3; *P* = 7.05E-143
k8-mer	β = 0.341; *P* = 2.72E-300	β = 0.499; *P* = 0.00E+00
k9-mer	β = -0.042; *P* = 7.99E-11	β = -0.025; *P* = 5.47E-06
k10-mer	β = -0.251; *P* = 0.00E+00	β = 0.019; *P* = 5.96E-05

Supplementary Table 18. ANOSIM results for comparison of distances in codon usage frequencies among the samples in GC bins. Samples were grouped into GC bins according to their average GC content of all coding sequences. GC bins were defined in the range of 5% GC variation starting from the sample with the lowest average GC content (35%). In each GC bin, codon usage frequencies were compared among seven selected habitats which are represented by more than 10 samples in the bin (to have sufficient numbers of samples from the test habitats for statistical test). Codon usage frequencies of the samples were calculated by two approaches absCUFs and synCUFs method (see main text) and in each GC bin, ANOSIM test was carried out considering samples from two habitats at a time using Euclidean distance method and 10,000 permutation values. Here N_1 and N_2 represent the number of samples in first and second habitat, respectively. ANOSIM test statistics are shown with R-values and significance level are shown with *P*-values.

GC bin	Habitat 1	N_1	Habitat 2	N_2	ANOSIM R-values for absCUFs	ANOSIM R-values for synCUFs
Gr-2	Digestive system	21	Water	14	R = 0.3758; *P* = 1.00E-04	R = 0.265; *P* = 6.00E-04
Gr-3	Digestive system	45	Water	17	R = 0.6019; *P* = 1.00E-04	R = 0.6151; *P* = 1.00E-04
Gr-4	Digestive system	52	Sediment	17	R = 0.5156; *P* = 1.00E-04	R = 0.4156; *P* = 1.00E-04
Gr-4	Digestive system	52	Wastewater	21	R = 0.6155; *P* = 1.00E-04	R = 0.6140; *P* = 1.00E-04
Gr-4	Digestive system	52	Water	12	R = 0.8036; *P* = 1.00E-04	R = 0.6912; *P* = 1.00E-04
Gr-4	Sediment	17	Wastewater	21	R = 0.2052; *P* = 5.00E-04	R = 0.2208; *P* = 5.00E-04
Gr-4	Sediment	17	Water	12	R = 0.2828; *P* = 6.00E-04	R = 0.2271; *P* = 3.50E-03

Gr-4	Wastewater	21	Water	12	R = 0.3733; P = 1.00E-04	R = 0.3387; P = 7.00E-04
Gr-5	Sediment	31	Soil	29	R = 0.1088; P = 3.10E-03	R = 0.09879; P = 2.80E-03
Gr-5	Sediment	31	Wastewater	16	R = 0.1917; P = 6.20E-03	R = 0.1803; P = 9.10E-03
Gr-5	Soil	29	Wastewater	16	R = 0.248; P = 5.00E-04	R = 0.2255; P = 1.10E-03

Supplementary Table 19. ANOSIM test results for comparison of ANOSIM R-values between real and random datasets. From the CDS sequences of each test sample, 20 sets of random coding sequences were generated using SPARCS algorithm that preserves the encoded protein sequence and the dinucleotide frequencies of each real sequence (see main text). Codon usage frequencies of real and random datasets of each sample were calculated by absCUFs and synCUFs methods and the codon usage distances among the samples were calculated by the Euclidean distance method (see main text). ANOSIM test was done for samples from any two-habitats at a time considering the codon usage frequencies of real and that of corresponding random datasets separately using 10,000 permutation values. For real dataset, ANOSIM test statistics are shown with R-values and significance levels are shown with *P*-values. To compare ANOSIM values obtained for codon frequencies of real and random datasets we considered Z-score approach. For samples from any two-habitats, Z-score is defined as Z-score= (ANOSIM R value obtained based on

codon frequencies of real dataset - Average of ANOSIM R-values obtained for corresponding random datasets)/standard deviation of ANOSIM R-values of obtained for corresponding random datasets. Here a positive Z-score indicates that ANOSIM R-values is higher for real dataset than that obtained for corresponding random datasets while negative Z-score indicates the opposite.

**HABITAT 1**	**HABITAT 2**	**ANOSIM R-values for REAL dataset**	**Z-SCORE for absCUFs**	**Z-SCORE for synCUFs**
Digestive system	Oral	R = 0.1883<br>*P* = 1.20E-03	5.075002	266.3145075
Digestive system	Sediment	R = 0.2231<br>*P* = 1.00E-04	0.593416	3.129408936
Digestive system	Skin	R = 0.4556<br>*P* = 1.00E-04	6.963037	34.52126944
Digestive system	Soil	R = 0.6608<br>*P* = 1.00E-04	-4.23446	-0.149390738
Digestive system	Wastewater	R = 0.2087<br>*P* = 1.00E-04	-1.79484	5.96914648

Digestive system	Water	R = 0.3842<br>*P* = 1.00E-04	4.426339	8.064834675
Oral	Sediment	R = 0.3368<br>*P* = 1.00E-04	1.647871	2.326829854
Oral	Skin	R = 0.3237<br>*P* = 2.00E-04	20.11179	24.64311474
Oral	Soil	R = 0.6935<br>*P* = 1.00E-04	-2.02874	-1.690277545
Oral	Wastewater	R = 0.253<br>*P* = 1.00E-04	0.742552	2.890503144
Oral	Water	R = 0.0395<br>*P* = 1.43E-01	3.738273	3.818954086
Sediment	Skin	R = 0.3367<br>*P* = 3.00E-04	5.664842	9.262796269
Sediment	Soil	R = 0.2941<br>*P* = 1.00E-04	-1.0328	-0.909796442
Sediment	Wastewater	R = 0.0717	2.044415	1.65606749

		P = 4.10E-03		
Sediment	Water	R = 0.3862<br>P = 1.00E-04	1.726538	1.939966087
Skin	Soil	R = 0.4419<br>P = 1.00E-04	-1.30809	-2.016145996
Skin	Wastewater	R = 0.3117<br>P = 3.00E-04	1.801429	3.776813021
Skin	Water	R = 0.3516<br>P = 1.00E-04	2.963094	3.748803889
Soil	Wastewater	R = 0.4127<br>P = 1.00E-04	0.722069	-0.347105295
Soil	Water	R = 0.6997<br>P = 1.00E-04	1.276294	2.78315967
Wastewater	Water	R = 0.2971<br>P = 1.00E-04	2.376525	2.081062163

Supplementary Table 20. List of COG and InterPro ids related to ribosomal proteins. Here is the list of COG and InterPro ids used for detection of probable ribosomal protein coding genes in the test metagenomic samples.

COG	Functional category	Information	Information	Information	Information
		COG entries			
COG0048	J	Ribosomal protein S12	RpsL	Ribosome 30S subunit	4V4H
COG0049	J	Ribosomal protein S7	RpsG	Ribosome 30S subunit	4V4H
COG0051	J	Ribosomal protein S10	RpsJ	Ribosome 30S subunit	4V4H
COG0052	J	Ribosomal protein S2	RpsB	Ribosome 30S subunit	4V4H
COG0080	J	Ribosomal protein L11	RplK	Ribosome 50S subunit	2.00E+34
COG0081	J	Ribosomal protein L1	RplA	Ribosome 50S subunit	3J65
COG0087	J	Ribosomal protein L3	RplC	Ribosome 50S subunit	1VS6
COG0088	J	Ribosomal protein L4	RplD	Ribosome 50S subunit	1VS6
COG0089	J	Ribosomal protein L23	RplW	Ribosome 50S subunit	1VS6
COG0090	J	Ribosomal protein L2	RplB	Ribosome 50S subunit	1VS6
COG0091	J	Ribosomal protein L22	RplV	Ribosome 50S subunit	1VS6
COG0092	J	Ribosomal protein S3	RpsC	Ribosome 30S subunit	1VS6
COG0093	J	Ribosomal protein L14	RplN	Ribosome 50S subunit	4V4H

COG0094	J	Ribosomal protein L5	RplE	Ribosome 50S subunit	1VS6
COG0096	J	Ribosomal protein S8	RpsH	Ribosome 30S subunit	4V4H
COG0097	J	Ribosomal protein L6P/L9E	RplF	Ribosome 50S subunit	1VS6
COG0098	J	Ribosomal protein S5	RpsE	Ribosome 30S subunit	4V4H
COG0099	J	Ribosomal protein S13	RpsM	Ribosome 30S subunit	4V4H
COG0100	J	Ribosomal protein S11	RpsK	Ribosome 30S subunit	4V4H
COG0102	J	Ribosomal protein L13	RplM	Ribosome 50S subunit	1VS6
COG0103	J	Ribosomal protein S9	RpsI	Ribosome 30S subunit	4V4H
COG0184	J	Ribosomal protein S15P/S13E	RpsO	Ribosome 30S subunit	4V4H
COG0185	J	Ribosomal protein S19	RpsS	Ribosome 30S subunit	4V4H
COG0186	J	Ribosomal protein S17	RpsQ	Ribosome 30S subunit	4V4H
COG0197	J	Ribosomal protein L16/L10AE	RplP	Ribosome 50S subunit	1WKI
COG0198	J	Ribosomal protein L24	RplX	Ribosome 50S subunit	1VS6
COG0199	J	Ribosomal protein S14	RpsN	Ribosome 30S subunit	4V4H
COG0200	J	Ribosomal protein L15	RplO	Ribosome 50S subunit	1VS6

COG0203	J	Ribosomal protein L17	RplQ	Ribosome 50S subunit	1GD8
COG0211	J	Ribosomal protein L27	RpmA	Ribosome 50S subunit	1VS6
COG0222	J	Ribosomal protein L7/L12	RplL	Ribosome 50S subunit	1CTF
COG0227	J	Ribosomal protein L28	RpmB	Ribosome 50S subunit	2JZ6
COG0228	J	Ribosomal protein S16	RpsP	Ribosome 30S subunit	4V4H
COG0230	J	Ribosomal protein L34	RpmH	Ribosome 50S subunit	6SPB
COG0238	J	Ribosomal protein S18	RpsR	Ribosome 30S subunit	4V4H
COG0244	J	Ribosomal protein L10	RplJ	Ribosome 50S subunit	5IT8
COG0254	J	Ribosomal protein L31	RpmE	Ribosome 50S subunit	1VS6
COG0255	J	Ribosomal protein L29	RpmC	Ribosome 50S subunit	1VS6
COG0256	J	Ribosomal protein L18	RplR	Ribosome 50S subunit	1VS6
COG0257	J	Ribosomal protein L36	RpmJ	Ribosome 50S subunit	1DFE
COG0261	J	Ribosomal protein L21	RplU	Ribosome 50S subunit	1VS6
COG0267	J	Ribosomal protein L33	RpmG	Ribosome 50S subunit	1VS6
COG0268	J	Ribosomal protein S20	RpsT	Ribosome 30S subunit	4V4H

COG0291	J	Ribosomal protein L35	RpmI	Ribosome 50S subunit	1VS6
COG0292	J	Ribosomal protein L20	RplT	Ribosome 50S subunit	1VS6
COG0333	J	Ribosomal protein L32	RpmF	Ribosome 50S subunit	1VS6
COG0335	J	Ribosomal protein L19	RplS	Ribosome 50S subunit	1VS6
COG0359	J	Ribosomal protein L9	RplI	Ribosome 50S subunit	1VS6
COG0360	J	Ribosomal protein S6	RpsF	Ribosome 30S subunit	4V4H
COG0456	J	Ribosomal protein S18 acetylase RimI and related acetyltransfer ases	RimI		1GHE
COG0522	J	Ribosomal protein S4 or related protein	RpsD	Ribosome 30S subunit	4V4H
COG0539	J	Ribosomal protein S1	RpsA	Ribosome 30S subunit	4NNH
COG0828	J	Ribosomal protein S21	RpsU	Ribosome 30S subunit	4V4H
COG1098	R	Predicted RNA-binding protein, contains ribosomal protein S1 (RPS1)	YabR		2K4K

		domain			
COG1358	J	Ribosomal protein L7Ae or related RNA K-turn-binding protein	Rpl7Ae	Ribosome 50S subunit	4LCK
COG1383	J	Ribosomal protein S17E	RPS17A	Archaeal ribosomal proteins	1RQ6
COG1471	J	Ribosomal protein S4E	RPS4A	Archaeal ribosomal proteins	
COG1552	J	Ribosomal protein L40E	RPL40A	Archaeal ribosomal proteins	2AYJ
COG1631	J	Ribosomal protein L44E	RPL42A	Archaeal ribosomal proteins	
COG1632	J	Ribosomal protein L15E	RPL15A	Archaeal ribosomal proteins	
COG1717	J	Ribosomal protein L32E	Rpl32e	Archaeal ribosomal proteins	
COG1727	J	Ribosomal protein L18E	RPL18A	Archaeal ribosomal proteins	
COG1825	J	Ribosomal protein L25 (general stress protein Ctc)	RplY	Ribosome 50S subunit	1B75

COG1841	J	Ribosomal protein L30/L7E	RpmD	Ribosome 50S subunit	1VS6
COG1890	J	Ribosomal protein S3AE	RPS3A	Archaeal ribosomal proteins	4CUY
COG1911	J	Ribosomal protein L30E	RPL30E	Archaeal ribosomal proteins	1CK2
COG1944	J	Ribosomal protein S12 methylthiotra nsferase accessory factor YcaO	YcaO		4BS9
COG1997	J	Ribosomal protein L37AE/L43A	RPL43A	Archaeal ribosomal proteins	1JJ2
COG1998	J	Ribosomal protein S27AE	RPS27ae	Archaeal ribosomal proteins	2K4X
COG2004	J	Ribosomal protein S24E	RPS24A	Archaeal ribosomal proteins	1XN9
COG2007	J	Ribosomal protein S8E	RPS8A	Archaeal ribosomal proteins	2KCO
COG2051	J	Ribosomal protein S27E	RPS27A	Archaeal ribosomal proteins	1QXF
COG2053	J	Ribosomal protein S28E/S33	RPS28A	Archaeal ribosomal proteins	1NE3
COG2058	J	Ribosomal protein L12E/L44/L4	RPP1A	Archaeal ribosomal proteins	

		5/RPP1/RPP2			
COG2075	J	Ribosomal protein L24E	RPL24A	Archaeal ribosomal proteins	
COG2097	J	Ribosomal protein L31E	RPL31A	Archaeal ribosomal proteins	
COG2125	J	Ribosomal protein S6E (S10)	RPS6A	Archaeal ribosomal proteins	4UER
COG2126	J	Ribosomal protein L37E	RPL37A	Archaeal ribosomal proteins	
COG2139	J	Ribosomal protein L21E	RPL21A	Archaeal ribosomal proteins	4BYN
COG2147	J	Ribosomal protein L19E	RPL19A	Archaeal ribosomal proteins	4BYN
COG2157	J	Ribosomal protein L20A (L18A)	RPL20A	Archaeal ribosomal proteins	2JXT
COG2163	J	Ribosomal protein L14E/L6E/L27E	RPL14A	Ribosome 50S subunit	3IZS
COG2167	J	Ribosomal protein L39E	RPL39	Archaeal ribosomal proteins	
COG2174	J	Ribosomal protein L34E	RPL34A	Archaeal ribosomal proteins	4BYN
COG2238	J	Ribosomal protein S19E (S16A)	RPS19A	Archaeal ribosomal proteins	2V7F

COG2264	J	Ribosomal protein L11 methylase PrmA	PrmA		3GRZ
COG2451	J	Ribosomal protein L35AE/L33A	Rpl35A	Archaeal ribosomal proteins	1SQR
COG2850	J	Ribosomal protein L16 Arg81 hydroxylase, contains JmjC domain	RoxA		1VRB
COG4352	J	Ribosomal protein L13E	RPL13	Archaeal ribosomal proteins	
COG4830	J	Ribosomal protein S26e	RPS26B	Archaeal ribosomal proteins	3JAP
COG4901	J	Ribosomal protein S25e	RPS25	Archaeal ribosomal proteins	4UJF
COG4919	J	Ribosomal protein S30	RPS30	Archaeal ribosomal proteins	
COG5459	J	Ribosomal protein RSM22 (predicted mitochondria l rRNA methylase)	Rsm22	Archaeal ribosomal proteins	
		InterPro entries			
IPR001014	Conserved_site	Ribosomal protein L23/L25, conserved site			

IPR001196	Conserved_site	Ribosomal protein L15, conserved site
IPR002358	Conserved_site	Ribosomal protein L6, conserved site
IPR002359	Conserved_site	Ribosomal protein L6, conserved site-2
IPR002363	Conserved_site	Ribosomal protein L10, eubacterial, conserved site
IPR004037	Conserved_site	Ribosomal protein L7Ae conserved site
IPR005825	Conserved_site	Ribosomal protein L24/L26, conserved site
IPR013000	Conserved_site	Ribosomal protein L4/L1e, eukaryotic/archaeal, conserved site
IPR018038	Conserved_site	Ribosomal protein L30, conserved site
IPR018065	Conserved_site	Ribosomal protein L34e, conserved site
IPR018079	Conserved_site	Ribosomal protein S4, conserved site
IPR018130	Conserved_site	Ribosomal protein S2, conserved site
IPR018192	Conserved_site	Ribosomal protein S5, N-terminal, conserved site
IPR018199	Conserved_site	Ribosomal protein S4e, N-terminal, conserved site
IPR018254	Conserved_site	Ribosomal protein L29, conserved site
IPR018255	Conserved_site	Ribosomal protein L10e, conserved site

IPR018256	Conserved_site	Ribosomal protein L13e, conserved site
IPR018257	Conserved_site	Ribosomal protein L19, conserved site
IPR018258	Conserved_site	Ribosomal protein L21, conserved site
IPR018259	Conserved_site	Ribosomal protein L21e, conserved site
IPR018260	Conserved_site	Ribosomal protein L22/L17, conserved site
IPR018261	Conserved_site	Ribosomal protein L27, conserved site
IPR018262	Conserved_site	Ribosomal protein L27e, conserved site
IPR018263	Conserved_site	Ribosomal protein L32e, conserved site
IPR018264	Conserved_site	Ribosomal protein L33, conserved site
IPR018265	Conserved_site	Ribosomal protein L35, conserved site
IPR018266	Conserved_site	Ribosomal protein L35Ae, conserved site
IPR018267	Conserved_site	Ribosomal protein L37e, conserved site
IPR018268	Conserved_site	Ribosomal protein S10, conserved site
IPR018269	Conserved_site	Ribosomal protein S13, conserved site
IPR018271	Conserved_site	Ribosomal protein S14, conserved site

IPR018273	Conserved_site	Ribosomal protein S17e, conserved site
IPR018275	Conserved_site	Ribosomal protein S18, conserved site
IPR018277	Conserved_site	Ribosomal protein S19e, conserved site
IPR018278	Conserved_site	Ribosomal protein S21, conserved site
IPR018279	Conserved_site	Ribosomal protein S21e, conserved site
IPR018280	Conserved_site	Ribosomal protein S3, conserved site
IPR018281	Conserved_site	Ribosomal protein S3Ae, conserved site
IPR018282	Conserved_site	Ribosomal protein S6/S6e/A/B/2, conserved site
IPR018283	Conserved_site	Ribosomal protein S8e, conserved site
IPR019926	Conserved_site	Ribosomal protein L3, conserved site
IPR019972	Conserved_site	Ribosomal protein L14P, conserved site
IPR019979	Conserved_site	Ribosomal protein S17, conserved site
IPR020052	Conserved_site	Ribosomal protein L31e, conserved site
IPR020083	Conserved_site	Ribosomal protein L39e, conserved site
IPR020574	Conserved_site	Ribosomal protein S9, conserved site

IPR020592	Conserved_site	Ribosomal protein S16, conserved site
IPR020606	Conserved_site	Ribosomal protein S7, conserved site
IPR020785	Conserved_site	Ribosomal protein L11, conserved site
IPR020798	Conserved_site	Ribosomal protein L16, conserved site
IPR020815	Conserved_site	Ribosomal protein S6, conserved site
IPR020925	Conserved_site	Ribosomal protein L15e, conserved site
IPR020929	Conserved_site	Ribosomal protein L5, conserved site
IPR020934	Conserved_site	Ribosomal protein S15/S19, conserved site
IPR020939	Conserved_site	Ribosomal protein L34, conserved site
IPR021132	Conserved_site	Ribosomal protein L18/L18-A/B/e, conserved site
IPR022671	Conserved_site	Ribosomal protein L2, conserved site
IPR022991	Conserved_site	Ribosomal protein L30e, conserved site
IPR023442	Conserved_site	Ribosomal protein L24e, conserved site
IPR023563	Conserved_site	Ribosomal protein L13, conserved site
IPR023638	Conserved_site	Ribosomal protein L19/L19e conserved site

IPR023673	Conserved_site	Ribosomal protein L1, conserved site
IPR028626	Conserved_site	Ribosomal protein S28e conserved site
IPR000196	Domain	Ribosomal protein L19/L19e
IPR001351	Domain	Ribosomal protein S3, C-terminal
IPR001912	Domain	Ribosomal protein S4/S9, N-terminal
IPR001975	Domain	Ribosomal protein L40e
IPR002784	Domain	Ribosomal protein L14e domain
IPR002906	Domain	Ribosomal protein S27a
IPR004038	Domain	Ribosomal protein L7Ae/L30e/S12e/Gadd45
IPR005324	Domain	Ribosomal protein S5, C-terminal
IPR005568	Domain	Ribosomal protein L6, N-terminal
IPR005633	Domain	Ribosomal protein L23/L25, N-terminal
IPR007741	Domain	Ribosomal protein/NADH dehydrogenase domain
IPR008932	Domain	Ribosomal protein L7/L12, oligomerisation
IPR012606	Domain	Ribosomal protein S13/S15, N-terminal

IPR012988	Domain	Ribosomal protein L30, N-terminal
IPR013810	Domain	Ribosomal protein S5, N-terminal
IPR013823	Domain	Ribosomal protein L7/L12, C-terminal
IPR013843	Domain	Ribosomal protein S4e, N-terminal
IPR013845	Domain	Ribosomal protein S4e, central region
IPR016082	Domain	Ribosomal protein L30, ferredoxin-like fold domain
IPR016180	Domain	Ribosomal protein L10e/L16
IPR019349	Domain	Ribosomal protein S24/S35, mitochondrial, conserved domain
IPR020040	Domain	Ribosomal protein L6, alpha-beta domain
IPR020057	Domain	Ribosomal protein L25, beta domain
IPR020069	Domain	Ribosomal protein L9, C-terminal
IPR020070	Domain	Ribosomal protein L9, N-terminal
IPR020783	Domain	Ribosomal protein L11, C-terminal
IPR020784	Domain	Ribosomal protein L11, N-terminal
IPR021131	Domain	Ribosomal protein L18e/L15P

IPR021757	Domain	Ribosomal protein L46, N-terminal
IPR022666	Domain	Ribosomal Proteins L2, RNA binding domain
IPR022669	Domain	Ribosomal protein L2, C-terminal
IPR023573	Domain	Ribosomal protein 50S-L18Ae/60S-L20/60S-L18A
IPR023611	Domain	Ribosomal protein S23/S25, mitochondrial
IPR023798	Domain	Ribosomal protein S7 domain
IPR025607	Domain	Ribosomal protein L5 eukaryotic, C-terminal
IPR025755	Domain	60S ribosomal protein L4, C-terminal domain
IPR027486	Domain	Ribosomal protein S10 domain
IPR031309	Domain	Ribosomal protein L5, C-terminal
IPR031310	Domain	Ribosomal protein L5, N-terminal
IPR032277	Domain	40S ribosomal protein S4, C-terminal domain
IPR032281	Domain	40S ribosomal protein SA, C-terminal domain
IPR032440	Domain	40S ribosomal protein S11, N-terminal
IPR032528	Domain	Sigma 54 modulation/S30EA ribosomal protein, C-terminal

IPR033650	Domain	Mitochondrial ribosomal protein L46 NUDIX
IPR033935	Domain	Ribosomal protein L19, eukaryotic
IPR033936	Domain	Ribosomal protein L19e, archaeal
IPR035808	Domain	Ribosomal protein L7, eukaryotic/archaeal
IPR040637	Domain	60S ribosomal protein L10P, insertion domain
IPR040894	Domain	54S ribosomal protein L8, C-terminal
IPR041905	Domain	Ribosomal protein S6 kinase alpha-3, C-terminal catalytic domain
IPR041982	Domain	Ribosomal protein S4, KOW domain
IPR041985	Domain	Ribosomal protein L14, KOW motif
IPR041988	Domain	Ribosomal protein L26/L24, KOW domain
IPR041991	Domain	Eukaryotic Ribosomal Protein L27, KOW domain
IPR041997	Domain	Ribosomal Protein L6, KOW domain
IPR042132	Domain	Ribosomal protein S6 kinase delta-1, PX domain
IPR044443	Domain	54S ribosomal protein L3, double-stranded RNA binding domain
IPR000039	Family	Ribosomal protein L18e

IPR000054	Family	Ribosomal protein L31e
IPR000077	Family	Ribosomal protein L39e
IPR000110	Family	Ribosomal protein S1
IPR000114	Family	Ribosomal protein L16
IPR000206	Family	Ribosomal protein L7/L12
IPR000218	Family	Ribosomal protein L14P
IPR000231	Family	Ribosomal protein L30e
IPR000235	Family	Ribosomal protein S5/S7
IPR000244	Family	Ribosomal protein L9
IPR000266	Family	Ribosomal protein S17/S11
IPR000271	Family	Ribosomal protein L34
IPR000289	Family	Ribosomal protein S28e
IPR000307	Family	Ribosomal protein S16
IPR000439	Family	Ribosomal protein L15e
IPR000456	Family	Ribosomal protein L17

IPR000473	Family	Ribosomal protein L36
IPR000509	Family	Ribosomal protein L36e
IPR000529	Family	Ribosomal protein S6
IPR000530	Family	Ribosomal protein S12e
IPR000552	Family	Ribosomal protein L44e
IPR000554	Family	Ribosomal protein S7e
IPR000589	Family	Ribosomal protein S15
IPR000592	Family	Ribosomal protein S27
IPR000597	Family	Ribosomal protein L3
IPR000630	Family	Ribosomal protein S8
IPR000702	Family	Ribosomal protein L6
IPR000754	Family	Ribosomal protein S9
IPR000851	Family	Ribosomal protein S5
IPR000876	Family	Ribosomal protein S4e
IPR000892	Family	Ribosomal protein S26e

IPR000911	Family	Ribosomal protein L11/L12
IPR000915	Family	60S ribosomal protein L6E
IPR000948	Family	Ribosomal protein L7Ae, prokaryotes
IPR000988	Family	Ribosomal protein L24e-related
IPR001021	Family	Ribosomal protein L25, long-form
IPR001047	Family	Ribosomal protein S8e
IPR001063	Family	Ribosomal protein L22/L17
IPR001141	Family	Ribosomal protein L27e
IPR001147	Family	Ribosomal protein L21e
IPR001197	Family	Ribosomal protein L10e
IPR001209	Family	Ribosomal protein S14
IPR001210	Family	Ribosomal protein S17e
IPR001266	Family	Ribosomal protein S19e
IPR001377	Family	Ribosomal protein S6e
IPR001380	Family	Ribosomal protein L13e

IPR001383	Family	Ribosomal protein L28
IPR001515	Family	Ribosomal protein L32e
IPR001569	Family	Ribosomal protein L37e
IPR001593	Family	Ribosomal protein S3Ae
IPR001648	Family	Ribosomal protein S18
IPR001684	Family	Ribosomal protein L27
IPR001705	Family	Ribosomal protein L33
IPR001706	Family	Ribosomal protein L35, non-mitochondrial
IPR001780	Family	Ribosomal protein L35A
IPR001787	Family	Ribosomal protein L21
IPR001790	Family	Ribosomal protein L10P
IPR001848	Family	Ribosomal protein S10
IPR001854	Family	Ribosomal protein L29/L35
IPR001857	Family	Ribosomal protein L19
IPR001859	Family	Trypanosoma cruzi ribosomal protein P2-like

IPR001865	Family	Ribosomal protein S2
IPR001892	Family	Ribosomal protein S13
IPR001911	Family	Ribosomal protein S21
IPR001921	Family	Ribosomal protein L7A/L8
IPR001931	Family	Ribosomal protein S21e
IPR001971	Family	Ribosomal protein S11
IPR001976	Family	Ribosomal protein S24e
IPR002132	Family	Ribosomal protein L5
IPR002136	Family	Ribosomal protein L4/L1e
IPR002143	Family	Ribosomal protein L1
IPR002150	Family	Ribosomal protein L31
IPR002171	Family	Ribosomal protein L2
IPR002222	Family	Ribosomal protein S19/S15
IPR002583	Family	Ribosomal protein S20
IPR002671	Family	Ribosomal protein L22e

IPR002672	Family	Ribosomal protein L28e
IPR002673	Family	Ribosomal protein L29e
IPR002674	Family	Ribosomal protein L37ae
IPR002675	Family	Ribosomal protein L38e
IPR002677	Family	Ribosomal protein L32p
IPR003256	Family	Ribosomal protein L24
IPR004389	Family	Ribosomal protein L18, bacterial-type
IPR004498	Family	Ribosomal protein L11 methyltransferase
IPR004977	Family	Ribosomal protein S25
IPR005290	Family	Ribosomal protein S15, bacterial-type
IPR005484	Family	Ribosomal protein L18
IPR005485	Family	Ribosomal protein L5 eukaryotic/L18 archaeal
IPR005679	Family	Ribosomal protein S12, bacterial-type
IPR005680	Family	Ribosomal protein S23, eukaryotic/archaeal
IPR005703	Family	Ribosomal protein S3, eukaryotic/archaeal

IPR005704	Family	Ribosomal protein S3, bacterial-type
IPR005706	Family	Ribosomal protein S2, bacteria/mitochondria/plastid
IPR005707	Family	Ribosomal protein S2, eukaryotic/archaeal
IPR005709	Family	Ribosomal protein S4, bacterial-type
IPR005710	Family	Ribosomal protein S4/S9, eukaryotic/archaeal
IPR005711	Family	Ribosomal protein S5, eukaryotic/archaeal
IPR005712	Family	Ribosomal protein S5, bacterial-type
IPR005713	Family	Ribosomal protein S19A/S15e
IPR005716	Family	Ribosomal protein S5/S7, eukaryotic/archaeal
IPR005717	Family	Ribosomal protein S7, bacterial/organellar-type
IPR005721	Family	Ribosomal protein L22/L17, eukaryotic/archaeal
IPR005727	Family	Ribosomal protein L22, bacterial/chloroplast-type
IPR005729	Family	Ribosomal protein S10, eukaryotic/archaeal
IPR005732	Family	Ribosomal protein S19, bacterial-type
IPR005745	Family	Ribosomal protein L14P, bacterial-type

IPR005749	Family	Ribosomal protein L15, bacterial-type
IPR005755	Family	Ribosomal protein L13, eukaryotic/archaeal
IPR005756	Family	Ribosomal protein L26/L24, eukaryotic/archaeal
IPR005813	Family	Ribosomal protein L20
IPR005822	Family	Ribosomal protein L13
IPR005823	Family	Ribosomal protein L13, bacterial-type
IPR005840	Family	Ribosomal protein S12 methylthiotransferase RimO
IPR005878	Family	Ribosomal protein L1, bacterial-type
IPR005879	Family	Ribosomal protein L1, mitochondrial
IPR005880	Family	Ribosomal protein L2, bacterial/organellar-type
IPR005996	Family	Ribosomal protein L30, bacterial-type
IPR005997	Family	Ribosomal protein L30, archaeal
IPR005998	Family	Ribosomal protein L7, eukaryotic
IPR006032	Family	Ribosomal protein S12/S23
IPR006519	Family	Ribosomal protein L11, bacterial-type

IPR006846	Family	Ribosomal protein S30
IPR006924	Family	Ribosomal protein PSRP-3/Ycf65
IPR007740	Family	Ribosomal protein L49/IMG2
IPR007836	Family	Ribosomal protein L41
IPR007980	Family	Ribosomal protein VAR1
IPR008092	Family	Ribosomal protein S29, mitochondrial
IPR008195	Family	Ribosomal protein L34Ae
IPR010729	Family	Ribosomal protein L47, mitochondrial
IPR010793	Family	Ribosomal protein L37/S30
IPR012607	Family	Ribosomal protein S22
IPR013005	Family	50S ribosomal protein uL4
IPR013025	Family	Ribosomal protein L25/L23
IPR013219	Family	Ribosomal protein S27/S33, mitochondrial
IPR013870	Family	Ribosomal protein L37, mitochondrial
IPR014401	Family	Ribosomal protein S6, eukaryotic

IPR015324	Family	Ribosomal protein Rsm22-like
IPR015608	Family	Putative ribosomal protein L7/L12, plants
IPR016238	Family	Ribosomal protein S6 kinase
IPR016239	Family	Ribosomal protein S6 kinase II
IPR016340	Family	Ribosomal protein L31, mitochondrial
IPR016522	Family	Ribosomal protein S22, mitochondrial, budding yeast
IPR016576	Family	Ribosomal protein 63, mitochondrial
IPR016712	Family	Mitochondrial ribosomal protein MRP51, fungi
IPR016939	Family	Mitochondrial ribosomal protein S25
IPR017081	Family	Ribosomal protein S24, mitochondrial
IPR017082	Family	Ribosomal protein S23, mitochondrial, fungi
IPR017127	Family	Ribosomal protein L3-specific, glutamine-N5-methyltransferase
IPR017264	Family	Ribosomal protein MRP10, mitochondrial
IPR018305	Family	Ribosomal protein L50, mitochondria
IPR018492	Family	Ribosomal protein L7Ae/L8/Nhp2 family

IPR018615	Family	Ribosomal protein L55, mitochondrial
IPR019189	Family	Ribosomal protein L27/L41, mitochondrial
IPR019192	Family	Ribosomal protein L28/L40, mitochondrial
IPR019266	Family	28S ribosomal protein S27, mitochondrial
IPR019338	Family	Ribosomal protein L35, mitochondrial
IPR019346	Family	39S ribosomal protein L42, mitochondrial
IPR019368	Family	Ribosomal protein S23/S29, mitochondrial
IPR019373	Family	Ribosomal protein L51, mitochondrial
IPR019374	Family	Ribosomal protein S22, mitochondrial
IPR019375	Family	Ribosomal protein S28, mitochondrial
IPR019520	Family	Ribosomal protein S23, mitochondrial
IPR019716	Family	Ribosomal protein L53, mitochondrial
IPR019906	Family	Ribosomal protein L6, bacterial-type
IPR019907	Family	Ribosomal protein L6P, archaea
IPR019927	Family	Ribosomal protein L3, bacterial/organelle-type

IPR019928	Family	Ribosomal protein L3, archaeal
IPR019958	Family	Ribosomal protein S9, archaeal
IPR019961	Family	Ribosomal protein S11, archaeal
IPR019970	Family	Ribosomal protein L4, archaea
IPR019971	Family	Ribosomal protein L14P, archaeal
IPR019977	Family	Ribosomal protein S13, archaeal
IPR019978	Family	Ribosomal protein S17, archaeal
IPR019980	Family	Ribosomal protein S13, bacterial-type
IPR019981	Family	Ribosomal protein S11, bacterial-type
IPR019984	Family	30S ribosomal protein S17
IPR019985	Family	Ribosomal protein L23
IPR020055	Family	Ribosomal protein L25, short-form
IPR020526	Family	Ribosomal protein L6, chloroplast
IPR020594	Family	Ribosomal protein L9, bacteria/chloroplast
IPR020814	Family	Ribosomal protein S6, plastid/chloroplast

IPR020919	Family	Ribosomal protein S8e, archaeal
IPR020924	Family	Ribosomal protein S6e, archaeal
IPR020926	Family	Ribosomal protein L15e, archaeal
IPR020930	Family	Ribosomal protein L5, bacterial-type
IPR021036	Family	Ribosomal protein S35, mitochondrial
IPR021137	Family	Ribosomal protein L35
IPR021138	Family	60S ribosomal protein L18a/ L20, eukaryotes
IPR022295	Family	Ribosomal protein L12, archaea
IPR022309	Family	Ribosomal protein S8e/ribosomal biogenesis NSA2
IPR022481	Family	Ribosomal protein L7Ae, archaea
IPR022801	Family	Ribosomal protein S4/S9
IPR022802	Family	Ribosomal protein S4, archaeal
IPR022804	Family	Ribosomal protein L5, archaeal
IPR022845	Family	Ribosomal protein S27ae
IPR022856	Family	Ribosomal protein L21e, archaeal

IPR022863	Family	Ribosomal protein S12, archaea
IPR022909	Family	50S ribosomal protein L10, archaea
IPR022947	Family	Ribosomal protein L18e, archaea
IPR022973	Family	Ribosomal protein L10
IPR022981	Family	Ribosomal protein L10e, archaea
IPR023029	Family	Ribosomal protein S15P
IPR023035	Family	Ribosomal protein S9, bacterial/plastid
IPR023036	Family	Ribosomal protein S14, bacterial/plastid
IPR023053	Family	Ribosomal protein S14, type Z
IPR023438	Family	Ribosomal protein L24e
IPR023454	Family	Ribosomal protein S2, archaeal
IPR023460	Family	Ribosomal protein L7Ae, putative
IPR023533	Family	Ribosomal protein S6--L-glutamate ligase RimK
IPR023651	Family	Ribosomal protein L14e
IPR023654	Family	Ribosomal protein L32e, archaeal

IPR023657	Family	Ribosomal protein L40e, archaeal
IPR023669	Family	Ribosomal protein L1, archaea
IPR023672	Family	Ribosomal protein L2, archaeal-type
IPR023676	Family	Ribosomal protein S14, type Z, archaeal
IPR024388	Family	Ribosomal protein L20, mitochondrial
IPR026018	Family	Ribosomal protein S7, archaeal
IPR026140	Family	28S ribosomal protein S26
IPR026146	Family	28S ribosomal protein S24, mitochondrial
IPR026299	Family	Mitochondrial 28S ribosomal protein S31
IPR026569	Family	Ribosomal protein L28/L24
IPR027487	Family	Mitochondrial ribosomal protein L48
IPR027488	Family	Ribosomal protein S3, archaeal
IPR027491	Family	Ribosomal protein L31 type A
IPR027493	Family	Ribosomal protein L31 type B
IPR027498	Family	Ribosomal protein S2, eukaryotic

IPR027500	Family	40S ribosomal protein S1/3, eukaryotes
IPR027504	Family	40S ribosomal protein SA
IPR027534	Family	Ribosomal protein L12/P1/P2 family
IPR027548	Family	Ribosomal protein S19e, archaeal
IPR028333	Family	Ribosomal protein S17, archaeal/eukaryotic
IPR028364	Family	Ribosomal protein L1/ribosomal biogenesis protein
IPR028370	Family	60S ribosomal protein L22-like 1
IPR028877	Family	50S ribosomal protein L18Ae/60S ribosomal protein L20 and L18a
IPR028909	Family	Ribosomal protein L21-like
IPR029751	Family	Ribosomal protein L25
IPR030670	Family	60S acidic ribosomal protein P0
IPR030826	Family	30S ribosomal protein
IPR030838	Family	30S ribosomal protein S3Ae
IPR030878	Family	Ribosomal protein L15
IPR031414	Family	30S ribosomal protein Thx

IPR032053	Family	Mitochondrial 28S ribosomal protein S34
IPR034596	Family	39S ribosomal protein L52, mitochondrial
IPR034600	Family	54S ribosomal protein L36, yeast
IPR034643	Family	Mitochondrial ribosomal protein S7, plants
IPR035050	Family	Ribosomal protein S6 kinase-like 1
IPR035104	Family	Ribosomal protein S1-like
IPR035306	Family	Ribosomal protein S12
IPR037447	Family	40S Ribosomal protein S10
IPR037507	Family	54S ribosomal protein L25
IPR039109	Family	Ribosomal protein L30/YlxQ
IPR039145	Family	39S ribosomal protein L40, mitochondrial
IPR039193	Family	28S ribosomal protein S17, mitochondrial
IPR039547	Family	60S ribosomal protein L19
IPR039660	Family	Ribosomal protein L14
IPR039699	Family	Ribosomal protein L7/L30

IPR039744	Family	40S ribosomal protein S29/30S ribosomal protein S14 type Z
IPR039848	Family	Ribosomal protein S24/S35, mitochondrial
IPR039927	Family	39S ribosomal protein L43/54S ribosomal protein L51
IPR039982	Family	Ribosomal protein S30, mitochondrial
IPR040008	Family	39S ribosomal protein L46, mitochondrial
IPR040030	Family	54S ribosomal protein L15, mitochondrial
IPR040049	Family	28S ribosomal protein S25, mitochondrial
IPR040054	Family	28S ribosomal protein S18b, mitochondrial
IPR040055	Family	28S ribosomal protein S10, mitochondrial
IPR040307	Family	50S ribosomal protein 5, chloroplastic
IPR042776	Family	54S ribosomal protein L44, mitochondrial
IPR042831	Family	54S ribosomal protein L28, mitochondrial
IPR044076	Family	Ribosomal protein P2
IPR044252	Family	60S acidic ribosomal protein P3-like
IPR044695	Family	30S ribosomal protein S31, plant

IPR044954	Family	Ribosomal protein S3, mitochondrial, plant
IPR044957	Family	Ribosomal protein L32p, bacterial type
IPR044958	Family	Ribosomal protein L32p, plant/cyanobacteria type
IPR045059	Family	60S ribosomal protein L35
IPR045077	Family	Ribosomal protein L3, archaeal/eukaryotic type
IPR045240	Family	Ribosomal protein L4, eukaryotic and archaeal type
IPR009027	Homologous_superfamily	Ribosomal protein L9/RNase H1, N-terminal
IPR010979	Homologous_superfamily	Ribosomal protein S13-like, H2TH
IPR011035	Homologous_superfamily	Ribosomal protein L25/Gln-tRNA synthetase, anti-codon-binding domain superfamily
IPR011331	Homologous_superfamily	Ribosomal protein L37ae/L37e
IPR011332	Homologous_superfamily	Zinc-binding ribosomal protein
IPR012678	Homologous_superfamily	Ribosomal protein L23/L15e core domain superfamily
IPR014717	Homologous_superfamily	Translation elongation factor EF1B/ribosomal protein S6
IPR014719	Homologous_superfamily	Ribosomal protein L7/L12, C-terminal/adaptor protein ClpS-like
IPR014721	Homologous_superfamily	Ribosomal protein S5 domain 2-type fold, subgroup

IPR014722	Homologous_superfamily	Ribosomal protein L2, domain 2
IPR014726	Homologous_superfamily	Ribosomal protein L2, domain 3
IPR015972	Homologous_superfamily	Ribosomal protein L19/L19e, domain 1
IPR015973	Homologous_superfamily	Ribosomal protein L19e, domain 2
IPR015974	Homologous_superfamily	Ribosomal protein L19e, domain 3
IPR016095	Homologous_superfamily	Ribosomal protein L1, 3-layer alpha/beta-sandwich
IPR020056	Homologous_superfamily	Ribosomal protein L25/Gln-tRNA synthetase, N-terminal
IPR020568	Homologous_superfamily	Ribosomal protein S5 domain 2-type fold
IPR022803	Homologous_superfamily	Ribosomal protein L5 domain superfamily
IPR023407	Homologous_superfamily	Ribosomal protein S27, zinc-binding domain superfamily
IPR023574	Homologous_superfamily	Ribosomal protein L4 domain superfamily
IPR023575	Homologous_superfamily	Ribosomal protein S19/S15, superfamily
IPR023591	Homologous_superfamily	Ribosomal protein S2, flavodoxin-like domain superfamily
IPR023621	Homologous_superfamily	Ribosomal protein L31e domain superfamily
IPR023626	Homologous_superfamily	Ribosomal protein L39e domain superfamily

IPR023674	Homologous_superfamily	Ribosomal protein L1-like
IPR023803	Homologous_superfamily	Ribosomal protein S16 domain superfamily
IPR024794	Homologous_superfamily	Ribosomal protein L15e core domain superfamily
IPR027437	Homologous_superfamily	30s ribosomal protein S13, C-terminal
IPR029064	Homologous_superfamily	50S ribosomal protein L30e-like
IPR035566	Homologous_superfamily	Ribosomal protein L20, C-terminal
IPR035970	Homologous_superfamily	Ribosomal protein L19/L19e superfamily
IPR035977	Homologous_superfamily	Ribosomal protein L36 superfamily
IPR035980	Homologous_superfamily	Ribosomal protein S6 superfamily
IPR035987	Homologous_superfamily	Ribosomal protein S8 superfamily
IPR036049	Homologous_superfamily	Ribosomal protein L29/L35 superfamily
IPR036235	Homologous_superfamily	Ribosomal protein L7/L12, oligomerisation domain superfamily
IPR036351	Homologous_superfamily	Ribosomal protein L32e superfamily
IPR036373	Homologous_superfamily	Ribosomal protein L17 superfamily
IPR036394	Homologous_superfamily	Ribosomal protein L22/L17 superfamily

IPR036401	Homologous_superfamily	Ribosomal protein S17e-like superfamily
IPR036419	Homologous_superfamily	Ribosomal protein S3, C-terminal domain superfamily
IPR036510	Homologous_superfamily	Ribosomal protein S20 superfamily
IPR036769	Homologous_superfamily	Ribosomal protein L11, C-terminal domain superfamily
IPR036789	Homologous_superfamily	Ribosomal protein L6, alpha-beta domain superfamily
IPR036791	Homologous_superfamily	Ribosomal protein L9, C-terminal domain superfamily
IPR036796	Homologous_superfamily	Ribosomal protein L11/L12, N-terminal domain superfamily
IPR036823	Homologous_superfamily	Ribosomal protein S7 domain superfamily
IPR036838	Homologous_superfamily	Ribosomal protein S10 domain superfamily
IPR036853	Homologous_superfamily	Ribosomal protein L14 superfamily
IPR036870	Homologous_superfamily	Ribosomal protein S18 superfamily
IPR036899	Homologous_superfamily	Ribosomal protein L13 superfamily
IPR036919	Homologous_superfamily	Ribosomal protein L30, ferredoxin-like fold domain superfamily
IPR036920	Homologous_superfamily	Ribosomal protein L10e/L16 superfamily
IPR036935	Homologous_superfamily	Ribosomal protein L9, N-terminal domain superfamily

IPR036948	Homologous_superfamily	Ribosomal protein L21 superfamily
IPR036967	Homologous_superfamily	Ribosomal protein S11 superfamily
IPR037121	Homologous_superfamily	Ribosomal protein L25, C-terminal
IPR037147	Homologous_superfamily	Ribosomal protein L28/L24 superfamily
IPR037229	Homologous_superfamily	Ribosomal protein L35 superfamily
IPR038097	Homologous_superfamily	Ribosomal protein L36e domain superfamily
IPR038237	Homologous_superfamily	Ribosomal protein S4e, central domain superfamily
IPR038380	Homologous_superfamily	Ribosomal protein S21 superfamily
IPR038416	Homologous_superfamily	Sigma 54 modulation/S30EA ribosomal protein, C-terminal domain superfamily
IPR038464	Homologous_superfamily	Ribosomal protein L38e superfamily
IPR038526	Homologous_superfamily	Ribosomal protein L22e superfamily
IPR038551	Homologous_superfamily	Ribosomal protein S26e superfamily
IPR038579	Homologous_superfamily	Ribosomal protein S21e superfamily
IPR038584	Homologous_superfamily	Ribosomal protein L33 superfamily
IPR038587	Homologous_superfamily	Ribosomal protein L40e superfamily

IPR038630	Homologous_superfamily	Ribosomal protein L24e/L24 superfamily
IPR038655	Homologous_superfamily	Ribosomal protein L27e superfamily
IPR038657	Homologous_superfamily	Ribosomal protein L19 superfamily
IPR038661	Homologous_superfamily	Ribosomal protein L35A superfamily
IPR038716	Homologous_superfamily	Ribosomal protein P1/P2, N-terminal domain
IPR042105	Homologous_superfamily	Ribosomal protein L31 superfamily
IPR042563	Homologous_superfamily	Ribosomal protein S8e subdomain, eukaryotes
IPR043140	Homologous_superfamily	Ribosomal protein S14/S29
IPR043141	Homologous_superfamily	Ribosomal protein L10-like domain superfamily
IPR043164	Homologous_superfamily	50S ribosomal protein L10, insertion domain superfamily
IPR044884	Homologous_superfamily	Mitochondrial ribosomal protein L55 superfamily
IPR044892	Homologous_superfamily	Ribosomal protein L3, domain 3, archaeal type superfamily

Supplementary References:

1. Leinonen, R., Sugawara, H., Shumway, M. & Collaboration, I.N.S.D. The sequence read archive. *Nucleic Acids Res.* **39**, D19-21 (2011).
2. Bolger, A.M., Lohse, M. & Usadel, B. Trimmomatic: a flexible trimmer for Illumina sequence data. *Bioinformatics* **30**, 2114-2120 (2014).
3. Langmead, B. & Salzberg, S.L. Fast gapped-read alignment with Bowtie 2. *Nat. Methods* **9**, 357-359 (2012).
4. Li, D., Liu, C.M., Luo, R., Sadakane, K. & Lam, T.W. MEGAHIT: an ultra-fast single-node solution for large and complex metagenomics assembly via succinct de Bruijn graph. *Bioinformatics* **31**, 1674-1676 (2015).
5. Hyatt, D., LoCascio, P.F., Hauser, L.J. & Uberbacher, E.C. Gene and translation initiation site prediction in metagenomic sequences. *Bioinformatics* **28**, 2223-2230 (2012).
6. Hyatt, D. *et al.* Prodigal: prokaryotic gene recognition and translation initiation site identification. *BMC Bioinform.* **11**, 119 (2010).
