# Supplementary material for: Determinants of associations between codon and amino acid usage patterns of microbial communities and the environment inferred based on a cross-biome metagenomic analysis
Source: NPJ Biofilms Microbiomes. 2023 Jan 24;9:5. doi: 10.1038/s41522-023-00372-w (PMC9873608; doi:10.1038/s41522-023-00372-w)
Supplement: Supplementary file 2 — Reporting Summary [file 41522_2023_372_MOESM2_ESM.pdf]

nature portfolio

Corresponding author(s): Tamir Tuller

Last updated by author(s): 2022/12/29

# Reporting Summary

Nature Portfolio wishes to improve the reproducibility of the work that we publish. This form provides structure for consistency and transparency in reporting. For further information on Nature Portfolio policies, see our Editorial Policies and the Editorial Policy Checklist.

## Statistics

For all statistical analyses, confirm that the following items are present in the figure legend, table legend, main text, or Methods section.

| n/a | Confirmed | |
|---|---|---|
| ☐ | ☒ | The exact sample size (*n*) for each experimental group/condition, given as a discrete number and unit of measurement |
| ☐ | ☒ | A statement on whether measurements were taken from distinct samples or whether the same sample was measured repeatedly |
| ☐ | ☒ | The statistical test(s) used AND whether they are one- or two-sided<br>*Only common tests should be described solely by name; describe more complex techniques in the Methods section.* |
| ☐ | ☒ | A description of all covariates tested |
| ☐ | ☒ | A description of any assumptions or corrections, such as tests of normality and adjustment for multiple comparisons |
| ☐ | ☒ | A full description of the statistical parameters including central tendency (e.g. means) or other basic estimates (e.g. regression coefficient) AND variation (e.g. standard deviation) or associated estimates of uncertainty (e.g. confidence intervals) |
| ☐ | ☒ | For null hypothesis testing, the test statistic (e.g. *F*, *t*, *r*) with confidence intervals, effect sizes, degrees of freedom and *P* value noted<br>*Give P values as exact values whenever suitable.* |
| ☒ | ☐ | For Bayesian analysis, information on the choice of priors and Markov chain Monte Carlo settings |
| ☐ | ☒ | For hierarchical and complex designs, identification of the appropriate level for tests and full reporting of outcomes |
| ☐ | ☒ | Estimates of effect sizes (e.g. Cohen's *d*, Pearson's *r*), indicating how they were calculated |

*Our web collection on statistics for biologists contains articles on many of the points above.*

## Software and code

Policy information about availability of computer code

| | |
|---|---|
| Data collection | Metagenomic project and sample information was collected from the MGnify metagenomic database. Raw sequence read files of each test sample were retrieved from the Sequence Read Archive database using fastq-dump tool. For comparison of results, predicted CDS and protein sequences of each sample were also collected from the MGnify metagenomic database. Protein and CDS sequences of microorganisms selected from the fusionDB database were downloaded from the NCBI GenBank database. |
| Data analysis | Sequence collection: fastq-dump algorithm (version 2.10.8)<br>Sequence quality filtering: BBDuk algorithm (version 38.57) and Trimmomatic algorithm (version 0.36)<br>Read alignment: Bowtie 2 algorithm (version 2.2.6)<br>Sequence quality check: FastQC algorithm (version 0.10.1)<br>Read assembly: MEGAHIT (version 1.2.9)<br>CDS/protein sequence prediction from contigs: Prodigal (version 2.6.3)<br>Taxonomic annotations of the sequences: Kraken2 (version 2.0.9-beta)<br>Functional annotation of the sequences: InterProScan (version 5.47-82.0), RPS-BLAST (version 2.2.18) and rpsbproc algorithms (version 0.5.0, Package blast 2.10.1)<br>Statistical analysis: R software (version 3.5.1) |

For manuscripts utilizing custom algorithms or software that are central to the research but not yet described in published literature, software must be made available to editors and reviewers. We strongly encourage code deposition in a community repository (e.g. GitHub). See the Nature Portfolio guidelines for submitting code & software for further information.

## Data

Policy information about availability of data

All manuscripts must include a data availability statement. This statement should provide the following information, where applicable:

- Accession codes, unique identifiers, or web links for publicly available datasets
- A description of any restrictions on data availability
- For clinical datasets or third party data, please ensure that the statement adheres to our policy

Quantitative data underpinning the results reported in this article are deposited in the online scientific data repository Zenodo (https://doi.org/10.5281/zenodo.7455261) and all supplementary tables and figures can be found in supplementary information supplied with this manuscript. All data related to this paper can also be requested from the corresponding author. All the codes used to generate results that are reported in this paper and central to its main claims are provided in supplementary files associated with this manuscript.

## Human research participants

Policy information about studies involving human research participants and Sex and Gender in Research.

| | |
|---|---|
| Reporting on sex and gender | Not relevant to our study |
| Population characteristics | Not relevant to our study |
| Recruitment | Not relevant to our study |
| Ethics oversight | Not relevant to our study |

Note that full information on the approval of the study protocol must also be provided in the manuscript.

# Field-specific reporting

Please select the one below that is the best fit for your research. If you are not sure, read the appropriate sections before making your selection.

☐ Life sciences ☐ Behavioural & social sciences ☒ Ecological, evolutionary & environmental sciences

For a reference copy of the document with all sections, see nature.com/documents/nr-reporting-summary-flat.pdf

# Ecological, evolutionary & environmental sciences study design

All studies must disclose on these points even when the disclosure is negative.

| | |
|---|---|
| Study description | In this study, we analyzed codon and amino acid usage frequencies of 422 metagenomic samples collected from 7 different environmental biomes. Further, we analyzed the influence of several other factors such as their GC content, taxonomic and functional abundance, k-mer frequencies on the codon/amino acid usage distances among the samples. Our study suggested that samples from similar environmental biomes tend to show comparable codon and amino acid usage frequencies as opposed to the samples from different environmental biomes. Our study suggested that this similarity in codon or amino acid usage cannot be fully explained considering the potential effect of GC content or functional or taxonomic similarities. |
| Research sample | For this study we considered 422 samples from collected from 7 different environmental biomes. We choose samples specifically from these 7 biomes because for each of these biomes we found at least 15 samples (helpful for reliable statistical tests) with at least 10,000 predicted CDS sequences. Raw sequence read files of each test sample were retrieved from the Sequence Read Archive database. Further, we also considered 925 microorganisms from fusionDB database to test the codon and amino acid choice of microorganisms at species level. Protein and CDS sequences of these microorganisms were downloaded from the NCBI GenBank database. |
| Sampling strategy | Here we considered samples from 7 different environmental biomes that have been studied by different groups of investigator at different times and locations. Therefore, multiple projects were deposited for most of the microbial habitats. Here we mainly considered the projects for which at least 5 samples are deposited (for reliable statistical tests) and for any habitat type if we found multiple projects were deposited by the same group of investigators we considered one project randomly. |
| Data collection | Metagenomic project and sample information was collected from the MGnify metagenomic database. Raw sequence read files of each test sample were retrieved from the Sequence Read Archive database using fastq-dump tool. For comparison of results, predicted CDS and protein sequences of each sample were also collected from the MGnify metagenomic database. Protein and CDS sequences of microorganisms selected from the fusionDB database were downloaded from the NCBI GenBank database. |
| Timing and spatial scale | The samples considered for this study were collected at different time interval starting from the year 2007 up to the year 2017. Samples were collected across the globe including the countries such as: Argentina, Australia, Austria, Belgium, Brazil, Canada, china, |

Denmark, Finland, France, Germany, Hungary, India, Iran, Israel, Italy, Kenya, Netherlands, New Zealand, Norway, Pakistan, Papua New Guinea, Philippines, Saudi Arabia, Slovakia, South Korea, Spain, Sweden, Switzerland, United Kingdom, and USA, etc.

| | |
|---|---|
| Data exclusions | No data were excluded during analysis. |
| Reproducibility | We predicted CDS sequences considering different subsets of reads from the total collection of all reads in each sample. In addition, we collected predicted CDS and protein sequences from MAGnify metagenomic database for each sample. In each case we found similar trends in codon and amino acid usage frequencies as we fund for our main dataset. |
| Randomization | For a metagenomic project if more than 5 samples were documented we considered 5 samples randomly. |
| Blinding | Our study is mainly analysis of samples that were collected by others. Further the samples were processed in batch mode without giving special attention to any particular sample. Thus we do not think blinding test is applicable here. |

Did the study involve field work? ☐ Yes ☒ No

# Reporting for specific materials, systems and methods

We require information from authors about some types of materials, experimental systems and methods used in many studies. Here, indicate whether each material, system or method listed is relevant to your study. If you are not sure if a list item applies to your research, read the appropriate section before selecting a response.

## Materials & experimental systems

| n/a | Involved in the study |
|---|---|
| ☒ | ☐ Antibodies |
| ☒ | ☐ Eukaryotic cell lines |
| ☒ | ☐ Palaeontology and archaeology |
| ☒ | ☐ Animals and other organisms |
| ☒ | ☐ Clinical data |
| ☒ | ☐ Dual use research of concern |

## Methods

| n/a | Involved in the study |
|---|---|
| ☒ | ☐ ChIP-seq |
| ☒ | ☐ Flow cytometry |
| ☒ | ☐ MRI-based neuroimaging |
