# Supplementary material for: Determinants of associations between codon and amino acid usage patterns of microbial communities and the environment inferred based on a cross-biome metagenomic analysis
Source: NPJ Biofilms Microbiomes. 2023 Jan 24;9:5. doi: 10.1038/s41522-023-00372-w (PMC9873608; doi:10.1038/s41522-023-00372-w)
Supplement: Supplementary file 3 — Supplementary software [file 41522_2023_372_MOESM3_ESM.zip › Supplementary_softwares/Description_of_codes.docx]

**Description of supplementary codes:**

**Determinants of associations between codon and amino acid usage patterns of microbial communities and the environment inferred based on a cross-biome metagenomic analysis**

Arup Panda^1^, Tamir Tuller^1^*

^1^Department of Biomedical Engineering, Tel Aviv University, Tel Aviv 69978, Israel

* Corresponding author

e-mail: tamirtul@post.tau.ac.il (Tamir Tuller)

**Contents:**

| Serial number | Content | Content type |
| --- | --- | --- |
| 1 | Metagenomic read processing example.pdf | PDF file |
| 2 | Bray-Curtis_bycombination.pl | Perl code |
| 3 | cal_aminoacid_frequency.pl | Perl code |
| 4 | cal_cai_values.pl | Perl code |
| 5 | cal_codon_freq.pl | Perl code |
| 6 | cal_dcbs_values.pl | Perl code |
| 7 | cal_enc_values.pl | Perl code |
| 8 | cal_GC_content.pl | Perl code |
| 9 | cal_synonymous_codonfreq_forsynCUFS.pl | Perl code |
| 10 | corrected_spearman_rho_withPvalues_Fast.pl | Perl code |
| 11 | euclidian_distance_bycombination.pl | Perl code |
| 12 | find_distance_between_clusters.pl | Perl code |
| 13 | GC_content_distance_bycombination.pl | Perl code |
| 14 | Pairwise_Anosim_NMDA_AminoAcid.pl | Perl code |
| 15 | pca_code_R.txt | R code |
| 16 | Rdensity_plot_fromPerl_forSynCUF.pl | Perl code |
| 17 | run_cdd_search.pl | Perl code |
| 18 | run_dishuffle.pl | Perl code |
| 19 | run_metagenomic_pipeline.pl | Perl code |
| 20 | synCUFS_from_codon_freq_table.pl | Perl code |
| 21 | Z_score_metagenome_Anosim.pl | Perl code |
