# Supplementary material for: Determinants of associations between codon and amino acid usage patterns of microbial communities and the environment inferred based on a cross-biome metagenomic analysis
Source: NPJ Biofilms Microbiomes. 2023 Jan 24;9:5. doi: 10.1038/s41522-023-00372-w (PMC9873608; doi:10.1038/s41522-023-00372-w)
Supplement: Supplementary file 3 — Supplementary software [file 41522_2023_372_MOESM3_ESM.zip › Supplementary_softwares/Metagenomic read processing example.pdf]

Metagenomic read processing example:

Determinants of associations between codon and amino acid usage patterns of microbial communities and the environment inferred based on a cross-biome metagenomic analysis

Arup Panda[1], Tamir Tuller[1]*

[1]Department of Biomedical Engineering, Tel Aviv University, Tel Aviv 69978, Israel

* Corresponding author

e-mail: tamirtul@post.tau.ac.il (Tamir Tuller)

Processing of metagenomic raw sequence reads

Raw sequences read of each sample in our analysis were retrieved from the Sequence Read Archive (SRA) database [1]. These reads are then processed with a rigorous quality check pipeline. As input, this pipeline accepts the raw sequence read files (either single-end or paired-end) of each sample in fastq format, processes the reads with different quality check algorithms, and provides high-quality filtered reads in the final step. Here we described the detailed parameters used in each step of this pipeline. The exact commands that we used in each step are given in the lines starting with "$" character and usage of the command is explained in the lines starting with "#" character. Here we considered sample corresponding to run id "ERR1035442" (sample id: ERS612889; project PRJEB7840) to explain the commands. Reads are paired-end so the commands (where applicable) were run in paired-end mode.

Retrieval of fastq reads using fastq-damp tool

In this step raw sequence read files of each sample were downloaded from the SRA database [1] using "fastq-dump" tool.

Command:

$esearch -db sra -query ERR1035442 | efetch -format runinfo

This command provides several useful information about the searched id such as read type (paired-end/single-end); collection date, etc.

$prefetch ERR1035442 --max-size 80G

This option allows fast transfer of files and saves the files in a newly-created directory. The "max-size" option specifies the size limit of the downloadable files.

$vdb-validate ERR1035442

#This option checks the integrity of the downloaded files to ensure successful download.

$fastq-dump --split-3 --skip-technical --readids --clip --read-filter pass --dumpbase --origfmt ERR1035442/ERR1035442.sra

This command converts downloaded .sra files to .fastq files. The "split-3" option separates the forward and reverse reads into separate files; any unpaired read without matching pair is saved in a separate file. The "skip-technical" flag dumps only biological reads. The "readids" option appends separate identifiers to the sequences in forward and reverse read files. The "clip" option removes sequences with unwanted tags such as tags used for whole genome amplification. The "read-filter" flag filters out reads that are all N's or otherwise completely useless. The option "dumpbase" ensures that the read files have nucleotide bases (A, T, G, C) instead of being put into color space.

Running sequence quality check by "BBDuk" algorithm

In this step, sequences containing bar code, adopter, or bad quality nucleotides are trimmed as well as low-quality sequences, and short sequences are removed using "BBDuk" algorithm of BBTools. BBTools is a suite of bioinformatics tools designed for the analysis of DNA and RNA next-generation sequence data developed by the Joint Genome Institute and are available for download at https://jgi.doe.gov/data-and-tools/bbtools/.

Command:

$bbduk.sh in1=ERR1035442_1.fastq in2=ERR1035442_2.fastq qin=auto out1=ERR1035442_1.bbdukclean.fastq out2=ERR1035442_2.bbdukclean.fastq outs=ERR1035442_bdduk_singleton.fastq ref=adapters.fa, truseq.fa.gz, truseq_rna.fa.gz, nextera.fa.gz,

phix_adapters.fa, phix174_ill.ref.fa, sequencing_artifacts.fa ktrim=r trimq=10 qtrim=rl minlength=50 k=18 mink=10 hdist=1 ordered stats=bbduk_quality_stats_ERR1035442.txt tbo t=1 -Xmx10g

#in1 and in2 flags specify the names of the input fastq files (forward and reverse); out1 and out2 are the names of the output files in fastq format (forward and reverse); "outs" stores the filtered reads in a separate file for which no matching paired is found; "qin=auto" option sets the input quality offset to be detect automatically; "ref" flag refers to the names of reference files against which the reads are aligned; "ktrim=r" option sets the trimming to be done at right side (3' adapters); "trimq" option specifies the minimum quality (in Phread score) of the reads below which reads will be trimmed; "qtrim" option decides the sides of quality trimming; "minlen" option specifies the minimum length of the reads below which they will be discarded; "k" option specifies the size of the kmer to be used for alignment; "mink" flag specifies the length of shorter kmers to be used to align the ends of the reads; "hdist" stands for hamming distance that allows one mismatch; "tbo" option is recommended option which allows to trim adapters based on pair overlap detection using BBMerge that does not require known adapter sequences; "ordered" option ensures the reads in output files will be in the same order as in the input files; "stats" option saves the reports produced by the algorithm in a (text) file; "t" option specifies the number threads to be used, and "-Xmx1" set the maximum limit of memory usage.

Re-checking sequence quality by Trimmomatic [2] algorithm

Command:

$java -jar Trimmomatic-0.36/trimmomatic-0.36.jar PE -threads 1 ERR1035442_1.bbdukclean.fastq ERR1035442_2.bbdukclean.fastq -baseout ERR1035442_trimmomatic.fastq ILLUMINACLIP:Trimmomatic-0.36/adapters/TruSeq3-PE.fa:2:30:10 LEADING:3 TRAILING:3 SLIDINGWINDOW:4:15 MINLEN:50

"PE" is selected to suggest paired-end reads; "baseout" flag sets the base names of output files; threads option specifies the number of treads to be used; ILLUMINACLIP option specifies trimming parameters; LEADING and TRAILING options check the quality of the read at the start and of the reads respectively and cut if below a threshold quality; MINLENTH option drops all reads below the specified length.

Checking presence of sequence contamination with Bowtie 2 [3] algorithm

In this step, we checked the presence of human sequences as contamination by aligning the reads against the human reference genome using the Bowtie 2 [3] algorithm.

Command:

$bowtie2 -x Bowtie2Index/humangenome -1 ERR1035442_trimmomatic_1P.fastq -2 ERR1035442_trimmomatic_2P.fastq -S ERR1035442_aligned.sam

This command maps the filtered reads against the reference database (here human reference genome) and keeps both mapped and unmapped reads.

$samtools view -bS ERR1035442_aligned.sam > ERR1035442_aligned.bam

#converts .sam file to .bam file

$samtools view -b -f 12 -F 256 ERR1035442_aligned.bam > ERR1035442_unmapped.bam

This command extracts only alignments with both reads unmapped.

$samtools sort -n ERR1035442_unmapped.bam ERR1035442_unmapped_sorted

#To sort the out file

$bedtools bamtofastq -i ERR1035442_unmapped_sorted.bam -fq ERR1035442_R1_filetred.fastq -fq2 ERR1035442_R2_filetred.fastq

This command splits paired-end reads into separated fastq files.

Running FastQC to check sequence quality after filtering

FastQC was downloaded from https://www.bioinformatics.babraham.ac.uk/projects/fastqc/.

Command:

$fastqc -t 1 ERR1035442_R1_filetred.fastq

$fastqc -t 1 ERR1035442_R2_filetred.fastq

#“t” flags sets number of threads to use.

Running MEGAHIT [4] assembly in paired end mode

In this step filtered raw reads were assembled into contigs using the MEGAHIT [4] algorithm. For this, we considered the quality-checked paired-end reads and also unpaired reads that were downloaded from the

SRA database [1] (reads without any matching pair) or generated during the quality filtering of pair-end reads. Unpaired reads were filtered following similar steps as we described for paired-end reads and were concatenated to a single file. Therefore there are three input files containing the reads.

Command:

$megahit -1 ERR1035442_R1_filetred.fastq -2 ERR1035442_R2_filetred.fastq -r ERR1035442_allsingleton_unpaired_filetred.fastq --min-contig-len 60 -t 1 --presets meta-sensitive -o ERR1035442.megahit_asm

“min-contig-len” flag specifies the minimum length of the contigs to be reported; “t” option specifies the number of threads to be used; “presets” flag is used to set the command parameters to different pre-adjusted options which control the sensitivity of the algorithm (here we considered sensitive option); “o” option sets the name of the output directory in which the results files will be stored.

Gene prediction by MetaProdigal [5, 6] algorithm

In this step, we predicted potential protein-coding sequences from the predicted contig file(s) of each sample.

Command:

$prodigal -i ERR1035442_contigs.fa -o ERR1035442_gff.gff -a ERR1035442_protein.faa -f gff -d ERR1035442_genes.faa -p meta –q

“-i” option specifies the name of input file; “-o”, “-a”,”-f” options set the names of the output files. “–p” flag indicates that the reads are metagenomics reads and adjust algorithm parameters accordingly.

References:

1. Leinonen, R., Sugawara, H., Shumway, M. & Collaboration, I.N.S.D. The sequence read archive. *Nucleic Acids Res.* **39**, D19-21 (2011).
2. Bolger, A.M., Lohse, M. & Usadel, B. Trimmomatic: a flexible trimmer for Illumina sequence data. *Bioinformatics* **30**, 2114-2120 (2014).

3. Langmead, B. & Salzberg, S.L. Fast gapped-read alignment with Bowtie 2. *Nat Methods* **9**, 357-359 (2012).
4. Li, D., Liu, C.M., Luo, R., Sadakane, K. & Lam, T.W. MEGAHIT: an ultra-fast single-node solution for large and complex metagenomics assembly via succinct de Bruijn graph. *Bioinformatics* **31**, 1674-1676 (2015).
5. Hyatt, D., LoCascio, P.F., Hauser, L.J. & Uberbacher, E.C. Gene and translation initiation site prediction in metagenomic sequences. *Bioinformatics* **28**, 2223-2230 (2012).
6. Hyatt, D. *et al.* Prodigal: prokaryotic gene recognition and translation initiation site identification. *BMC Bioinform.* **11**, 119 (2010).
